# Alzheimer’s Is a Multiform Disease of Sustained Neuronal Integrated Stress Response Driven by the C99 Fragment Generated Independently of AβPP; Proteolytic Production of Aβ Is Suppressed in AD-Affected Neurons: Evolution of a Theory

**DOI:** 10.3390/ijms26094252

**Published:** 2025-04-29

**Authors:** Vladimir Volloch, Sophia Rits-Volloch

**Affiliations:** 1 Department of Developmental Biology, Harvard School of Dental Medicine, Boston, MA 02115, USA; 2 Division of Molecular Medicine, Children’s Hospital, Boston, MA 02115, USA; 3 Department of Biological Chemistry and Molecular Pharmacology, Harvard Medical School, Boston, MA 02115, USA

**Keywords:** Amyloid Cascade Hypothesis 2.0 (ACH2.0), conventional and unconventional Alzheimer’s disease (AD), AβPP-independent generation of the C99 fragment, neuronal integrated stress response (ISR), AD as the disease of the neuronal ISR, ISR-mediated suppression of the AβPP proteolytic pathway in AD-affected neurons, C99 as the driver of AD, inhibition of the neuronal ISR as AD therapy, concurrent inhibition of the neuronal ISR and activation of BACE1 and BACE2 as composite AD therapy, RNA-dependent amplification of human AβPP mRNA, RNA-based AD therapies

## Abstract

The present *Perspective* analyzes the remarkable evolution of the Amyloid Cascade Hypothesis 2.0 (ACH2.0) theory of Alzheimer’s disease (AD) since its inception a few years ago, as reflected in the diminishing role of amyloid-beta (Aβ) in the disease. In the initial iteration of the ACH2.0, Aβ-protein-precursor (AβPP)-derived intraneuronal Aβ (iAβ), accumulated to neuronal integrated stress response (ISR)-eliciting levels, triggers AD. The neuronal ISR, in turn, activates the AβPP-independent production of its C99 fragment that is processed into iAβ, which drives the disease. The second iteration of the ACH2.0 stemmed from the realization that AD is, in fact, a disease of the sustained neuronal ISR. It introduced two categories of AD—conventional and unconventional—differing mainly in the manner of their causation. The former is caused by the neuronal ISR triggered by AβPP-derived iAβ, whereas in the latter, the neuronal ISR is elicited by stressors distinct from AβPP-derived iAβ and arising from brain trauma, viral and bacterial infections, and various types of inflammation. Moreover, conventional AD always contains an unconventional component, and in both forms, the disease is driven by iAβ generated independently of AβPP. In its third, the current, iteration, the ACH2.0 posits that proteolytic production of Aβ is suppressed in AD-affected neurons and that the disease is driven by C99 generated independently of AβPP. Suppression of Aβ production in AD seems an oxymoron: Aβ is equated with AD, and the later is inconceivable without the former in an ingrained Amyloid Cascade Hypothesis (ACH)-based notion. But suppression of Aβ production in AD-affected neurons is where the logic leads, and to follow it we only need to overcome the inertia of the preexisting assumptions. Moreover, not only is the generation of Aβ suppressed, so is the production of all components of the AβPP proteolytic pathway. This assertion is not a quantum leap (unless overcoming the inertia counts as such): the global cellular protein synthesis is severely suppressed under the neuronal ISR conditions, and there is no reason for constituents of the AβPP proteolytic pathway to be exempted, and they, apparently, are not, as indicated by the empirical data. In contrast, tau protein translation persists in AD-affected neurons under ISR conditions because the human tau mRNA contains an internal ribosomal entry site in its 5′UTR. In current mouse models, iAβ derived from AβPP expressed exogenously from human transgenes elicits the neuronal ISR and thus suppresses its own production. Its levels cannot principally reach AD pathology-causing levels regardless of the number of transgenes or the types of FAD mutations that they (or additional transgenes) carry. Since the AβPP-independent C99 production pathway is inoperative in mice, the current transgenic models have no potential for developing the full spectrum of AD pathology. What they display are only effects of the AβPP-derived iAβ-elicited neuronal ISR. The paper describes strategies to construct adequate transgenic AD models. It also details the utilization of human neuronal cells as the only adequate model system currently available for conventional and unconventional AD. The final alteration of the ACH2.0, introduced in the present *Perspective*, is that AβPP, which supports neuronal functionality and viability, is, after all, potentially produced in AD-affected neurons, albeit not conventionally but in an ISR-driven and -compatible process. Thus, the present narrative begins with the “omnipotent” Aβ capable of both triggering and driving the disease and ends up with this peptide largely dislodged from its pedestal and retaining its central role in triggering the disease in only one, although prevalent (conventional), category of AD (and driving it in none). Among interesting inferences of the present *Perspective* is the determination that “sporadic AD” is not sporadic at all (“non-familial” would be a much better designation). The term has fatalistic connotations, implying that the disease can strike at random. This is patently not the case: The conventional disease affects a distinct subpopulation, and the basis for unconventional AD is well understood. Another conclusion is that, unless prevented, the occurrence of conventional AD is inevitable given a sufficiently long lifespan. This *Perspective* also defines therapeutic directions not to be taken as well as auspicious ways forward. The former category includes ACH-based drugs (those interfering with the proteolytic production of Aβ and/or depleting extracellular Aβ). They are legitimate (albeit inefficient) preventive agents for conventional AD. There is, however, a proverbial snowball’s chance in hell of them being effective in symptomatic AD, lecanemab, donanemab, and any other “…mab” or “…stat” notwithstanding. They comprise Aβ-specific antibodies, inhibitors of beta- and gamma-secretase, and modulators of the latter. In the latter category, among ways to go are the following: (1) Depletion of iAβ, which, if sufficiently “deep”, opens up a tantalizing possibility of once-in-a-lifetime preventive transient treatment for conventional AD and aging-associated cognitive decline, AACD. (2) Composite therapy comprising the degradation of C99/iAβ and concurrent inhibition of the neuronal ISR. A single transient treatment could be sufficient to arrest the progression of conventional AD and prevent its recurrence for life. Multiple recurrent treatments would achieve the same outcome in unconventional AD. Alternatively, the sustained reduction/removal of unconventional neuronal ISR-eliciting stressors through the elimination of their source would convert unconventional AD into conventional one, preventable/treatable by a single transient administration of the composite C99/iAβ depletion/ISR suppression therapy. Efficient and suitable ISR inhibitors are available, and it is explicitly clear where to look for C99/iAβ-specific targeted degradation agents—activators of BACE1 and, especially, BACE2. Directly acting C99/iAβ-specific degradation agents such as proteolysis-targeting chimeras (PROTACs) and molecular-glue degraders (MGDs) are also viable options. (3) A circumscribed shift (either upstream or downstream) of the position of transcription start site (TSS) of the human AβPP gene, or, alternatively, a gene editing-mediated excision or replacement of a small, defined segment of its portion encoding 5′-untranslated region of AβPP mRNA; targeting AβPP RNA with anti-antisense oligonucleotides is another possibility. If properly executed, these RNA-based strategies would not interfere with the protein-coding potential of AβPP mRNA, and each would be capable of both preventing and stopping the AβPP-independent generation of C99 and thus of either preventing AD or arresting the progression of the disease in its conventional and unconventional forms. The paper is interspersed with “validation” sections: every conceptually significant notion is either validated by the existing data or an experimental procedure validating it is proposed.

## 1. Introduction

The present *Perspective* outlines and analyzes the remarkable evolution of a theory of Alzheimer’s disease (AD), designated the Amyloid Cascade Hypothesis 2.0 (ACH2.0), since its formulation a few years ago [1,2,3,4,5,6,7,8,9,10]. The ACH2.0 shares a portion of its name with the preceding theory of AD, the Amyloid Cascade Hypothesis (ACH). This overlap is intentional. In the ACH, amyloid-beta (Aβ) is both the causative agent and the driver of the disease. At the time of the inception of the ACH2.0, it was assumed that the same is conceptually true for this theory of AD as well. With time, our understanding of AD evolved, and the ACH2.0 evolved accordingly. If anything, the evolution of the ACH2.0 is a story of the diminishing role of Aβ in the disease. In the initial iteration of the ACH2.0, Aβ-protein-precursor (AβPP)-derived intraneuronal Aβ (iAβ) accumulated to neuronal integrated stress response (ISR)-eliciting levels triggers AD, and iAβ produced independently of AβPP drives the disease. The realization that AD is actually a disease of the sustained neuronal ISR (which activates AβPP-independent production of C99 and, potentially, iAβ) prompted the second iteration of the ACH2.0. It introduced two categories of AD, conventional and unconventional, differing mainly in the manner of their causation. The former is caused by the neuronal ISR triggered by AβPP-derived iAβ, whereas in the latter, stressors that are distinct from AβPP-derived iAβ and arise from traumatic brain injury, bacterial and viral infections, and various forms of inflammation elicit the neuronal ISR. In its both forms, the disease is driven by iAβ generated independently of AβPP. The third iteration of the ACH2.0 posits that proteolytic production of Aβ is suppressed in AD-affected neurons and that the C99 fragment of AβPP generated independently of AβPP drives the disease. Moreover, not only is the production of Aβ suppressed, so is the production of all components of the AβPP proteolytic pathway, including AβPP and beta- and gamma-secretases. The final modification of the ACH2.0, introduced in the present *Perspective*, is that AβPP, which is required for the neuronal functionality and viability, is, after all, produced in AD-affected neurons, albeit not conventionally but in an ISR-driven and -compatible process. Thus, the story we are about to relay begins with an “omnipotent” Aβ capable of both triggering and driving the disease and ends with this peptide largely dislodged from its pedestal and retaining its central role in merely triggering the disease in only one, albeit prevalent, (conventional) category of AD (and driving it in none; thus, “central but not causative” in George Perry’s words). To present the complete picture, we begin the story with the birth of AD as a distinct research field.

## 2. A Brief Historical Background

The field of Alzheimer’s disease has a defined birthday: 3 November 1906. On that day, Dr. Alois Alzheimer presented a case study of his patient, Auguste D., at the 37th annual meeting of the German regional psychiatric association in Tubingen. The disorder that he described was yet to be called “Alzheimer’s disease” but was referred to as “a peculiar severe disease of the cerebral cortex” marked by distinctive neuritic plaques and neurofibrillary tangles as its main histological attributes. The presentation elicited little enthusiasm or even interest; no post-presentation discussion, customary at that time, followed. In fact, not even questions were asked, and the session progressed promptly to more fashionable psychoanalytical cases. Nevertheless, the abstract of this presentation was included in the proceedings of the conference published in 1906 [11], and one year later, in 1907, Dr. Alzheimer succeeded in publishing his complete lecture [12]. The disease acquired its by now familiar name only in 1910, when Dr. Kraepelin, a colleague and mentor of Dr. Alzheimer, described it in the eighth edition of his textbook “Psychiatrie” [13,14] and referred to it as “Alzheimer’s disease”. Initially, the field developed quite slowly, in part due to the rarity of the disease (the life expectancy in Germany at the time was 48 years, insufficient for sporadic AD to develop; Dr. Alzheimer himself, for example, died in 1915 aged only 51); in his remaining lifetime, Dr. Alzheimer described only four additional clinical cases [15,16]. A noticeable milestone occurred in 1968, when Blessed and co-workers described what they interpreted as a quantitative association between cerebral plaques in brains of elderly subjects and senile changes in their behavior [17]. The next milestone took place in 1984, almost 80 years after the introduction of AD. This is the year when Glenner and Wong identified and sequenced the main component of neuritic plaques (Dr. Alzheimer referred to plaques as “miliary foci”, which he described as dystrophic neuronal processes around a “special substance in the cortex”; what Glenner and Wong isolated and characterized was this “special substance”); they designated it as amyloid-beta (Aβ) peptide and speculated that it was derived from a larger precursor [18]. Since this seminal event, the development of the field rapidly accelerated. In 1987 three research groups, using the amino acid sequence of Aβ elucidated in [17], confirmed the prediction of Glenner and Wong [18]: they identified, independently and nearly simultaneously, the gene encoding the protein precursor of human Aβ (AβPP) and obtained a complete sequence of its cDNA [19,20,21]. Finally, in 1991, Goate and co-workers detected the first (of many to follow) AD-causing AβPP mutation [22]. This burst of developments in the field culminated, in 1992, in the formulation, by Hardy and Higgins, of a major theory of AD called the Amyloid Cascade Hypothesis, ACH [23]. This theory was destined to guide the field for the following three decades.

## 3. The Amyloid Cascade Hypothesis: From Domination to Unsustainability

The essence of the ACH is best summarized in the words of its inventors, Hardy and Higgins: “Our hypothesis is that the extracellular deposition of amyloid β protein, the main component of the plaques, is the causative agent of Alzheimer’s pathology and that the neurofibrillary tangles, cell loss, vascular damage, and dementia follow as the direct result of this deposition” [23]. It should be emphasized that in 1992, the time of formulation of the ACH, cerebral plaques ostensibly associated with AD were known for decades; it was, in fact Dr. Alzheimer who established their occurrence in the disease [11,12]. The identity of the main component of the plaques was also known since 1984 [18], for a better part of a decade. This knowledge, however, was insufficient to anoint Aβ as the causative agent of AD. What allowed it was the discovery, in 1991, of an AβPP mutation affecting the production of Aβ in the AβPP proteolytic pathway and segregating with the early-onset disease (familiar AD, FAD) [22].

At the time of its introduction, the ACH was consistent with the accumulated body of empirical data. It also defined the path forward and was, therefore, enthusiastically accepted by the scientific community. The ACH guided the design and construction of transgenic AD models and the development of AD drugs. The logic of these endeavors was straightforward: overexpress human AβPP in mice and the disease will follow; deplete extracellular Aβ and reduce the amyloid plaques load and the disease would be stopped and possibly cured. Under this guidance numerous AD drug candidates were developed that either deplete extracellular Aβ (e.g., various Aβ-specific monoclonal antibodies) or suppress the production of Aβ in the AβPP proteolytic pathway (e.g., inhibitors of the beta-site AβPP cleaving enzyme, BACE1, also known as beta-secretase). These types of AD drugs are referred to henceforth as “ACH-based drugs”. Many ACH-based drugs were spectacularly successful not only in reducing but also in reversing the symptoms in transgenic mouse models overexpressing Aβ from numerous human AβPP transgenes [24,25,26].

But this is where the first cracks in the ACH appeared. Transgenic, human Aβ-overexpressing mice indeed deposit excessive amyloid plaques and exhibit some neurodegeneration and certain cognitive impairments, such as defects of the neuronal plasticity, learning, and memory formation. However, these mouse models appear unable to develop the full spectrum of AD pathology. Tellingly, none of the ubiquitous current transgenic mouse models develops neurofibrillary tangles (NFT), a major hallmark of AD. Therefore, it can be argued, and, indeed, it was argued by us [7,10] that transgenic mice overexpressing human AβPP are not AD models (further discussed in Section 19 below). Whatever symptoms and pathology these models develop are apparently due to the overexpression of human Aβ. It is, therefore, no surprise that ACH-based drugs targeting overexpressed Aβ provide relief from those symptoms and pathology.

The cracks in the ACH theory became chasms and crevasses when numerous human trials of candidate ACH-based drugs in symptomatic AD were conducted. In all such trials ACH-based drugs failed as spectacularly as they succeeded in transgenic mouse models; no efficacy whatsoever was seen, with some trials terminating prematurely [27,28] (the observed marginal effects of lecanemab and donanemab in very early AD [29,30,31,32,33] do not contradict this statement; see Section 39 below). Importantly, it became apparent that the drugs failed not because they underperformed mechanistically in humans (versus mice). To the contrary, the results of the trials indicated that they performed remarkably well in humans. Thus, verubecestat, an inhibitor of BACE1, substantially suppressed the production of Aβ in the AβPP proteolytic pathway and cleared extracellular Aβ, resulting in 80% drop of levels of Aβ in CSF of AD patients [27,28]. The findings that the drugs accomplished their mechanistic mission but did not provide any relief to the patients suggested that the underlying theory is incorrect.

Furthermore, as PET scan methods were developed that allow the evaluation of the amyloid plaques load in the living brain, it became apparent that there is no good correlation between the amount of amyloid deposition and the occurrence of AD. In a large fraction, about one third, of the aged general population the degree of extracellular Aβ deposition is comparable with or even exceeds that seen in AD patients. These individuals, however, develop neither cognitive impairment nor AD pathology in their lifetimes [34,35,36,37,38,39,40]. The reverse is also true: some AD patients exhibit no excessive levels of extracellular Aβ deposition [41]. Considered cumulatively, the observations described above provide a convincing indication that extracellular Aβ neither causes nor drives AD pathology and, consequently, that the ACH is unsustainable.

## 4. The Central Role of Aβ in Conventional Alzheimer’s Disease Is Incontestable

The conclusion of the preceding section notwithstanding, the centrality of Aβ in conventional Alzheimer’s disease is indisputable. The basis for this assertion is the nature of mutations that either cause familiar AD or protect from the disease. As mentioned above, Goate and co-workers discovered the first FAD-causing mutation in 1991 [22]. Since then, numerous mutations within either AβPP of presenilins (PSENs, components of the gamma-secretase complex) were discovered [42]. With one exception, all these mutations cause early-onset AD. The exception is the Icelandic mutation within the Aβ segment of AβPP, which confers on its carriers protection not only from AD but also from aging-associated cognitive decline, AACD [43,44]. All these mutations have one common attribute: they affect only the structure, production, and cleavages of Aβ. Hardy and Higgins deemed a single AβPP AD-causing mutation sufficient to assign to Aβ the central role in the disease [23], a notion readily accepted by the AD research field. The discovery of dozens of such mutations affecting, in one way or another, solely Aβ strongly affirm the centrality of Aβ in AD. Below, in Section 32, Section 33, Section 34 and Section 35, we describe the mechanistic underlying of these mutations. The mechanisms involved not only explain how these mutations exert their effect (i.e., the causation of or protection from AD and AACD) but also suggest effective approaches to intervene with the disease. Total cerebral Aβ population consists of two subclasses: extracellular Aβ and intraneuronal Aβ (iAβ), both accumulating during the lifetime (physiologically occurring processes underlying accumulation of iAβ are described in Section 24 below). Since Aβ is apparently central in Alzheimer’s disease and because extracellular Aβ can be ruled out as the causative and driving agent of AD, it logically follows that this distinction belongs to intraneuronal Aβ. This conclusion is supported by numerous observations that AD symptoms correlate much better with the levels of iAβ than with those of extracellular Aβ [45,46,47,48,49,50,51,52,53,54,55,56,57]. Co-incidentally, as described in the following section, the same conclusion is derived from the results of clinical trials of ACH-based candidate drugs in symptomatic AD.

## 5. The ACH2.0, Initial Version: Conventional Alzheimer’s Disease Is Triggered by AβPP-Derived Intraneuronal Aβ (iAβ) and Driven by iAβ Generated Independently of AβPP

The major principles of the initial (Variant One) formulation of the ACH2.0, an AD theory replacing the ACH, stem from two sources. One is the certitude, articulated above, that conventional AD is triggered and driven by Aβ. Another source is the results of multiple clinical trials of candidate ACH-based drugs in symptomatic AD. Of those, especially important are trials of verubecestat, an effective inhibitor of BACE1 [27,28]. This is because whereas drugs like Aβ-specific antibodies deplete extracellular Aβ, verubecestat (and other BACE1 inhibitors) both clears extracellular Aβ and suppresses the production of Aβ in the AβPP proteolytic pathway.

The logic applied to the analysis of the outcomes of clinical trials is straightforward. (1) A substantial removal of extracellular Aβ has no positive effect whatsoever. Therefore, it is intraneuronal Aβ, iAβ, which triggers and drives AD. (2) A substantial suppression of the production of Aβ in the AβPP proteolytic pathway has no positive effect whatsoever. Therefore, the intraneuronal Aβ that drives AD is generated independently of AβPP and retained within the disease-affected neurons. These two notions constitute the principal attributes of the ACH2.0 in its initial iteration [1], which posits the following: Alzheimer’s disease is triggered by AβPP-derived intraneuronal Aβ, iAβ, accumulated over certain critical threshold, and is driven by Aβ produced independently of Aβ-protein-precursor (AβPP) and retained intraneuronally as iAβ [1,2,3,4,5,6,7,8,9,10]. Whereas the notion of intraneuronal Aβ, iAβ, is, as described below, rather conventional and its origins are well understood, the concept of the generation of Aβ independently of its genome-encoded precursor, AβPP, is anything but (this is the reason why one of our initial publications on the subject is entitled “News from Mars” [58]). It may appear outlandish and even farfetched. It is neither. In fact, this concept, which defines the active core, the “engine” of AD in the ACH2.0 paradigm, has been already formulated, twice, a couple of decades previously but was deemed unnecessary and unfounded, and was “waiting in the wings” for a considerable time. Presently, it is strongly supported by the empirical data, and its time has apparently arrived. Familiarity with it is necessary in order to continue the present narrative. The following is its brief history (note that the present narrative resumes in Section 24 below).

## 6. Singular Attributes of the AUG Codon Encoding Methionine 671 of Human AβPP

The present section picks up the AD story in 1987. This is the year when several laboratories, independently and nearly simultaneously, cloned and sequenced cDNA encoding human AβPP [19,20,21]. As a reminder, it is 770 amino acid residues long (albeit there are shorter and, actually, some longer versions) and Aβ is embedded within its C-terminal segment and released by two proteolytic cleavages. One is enacted by beta-secretase, known also as BACE (Beta-site AβPP-Cleaving Enzyme). The “beta-site” of the above designation is localized between amino acid residues 671 and 672 and the cleavage by BACE at this site produces the 99 amino acid residues long C-terminal fragment (CTF) of AβPP designated rather imaginatively C99. C99 contains Aβ as its N-terminal segment and Asp672 of AβPP forms the N-end of both C99 and the future Aβ. This is the immediate precursor of Aβ. The release of Aβ is completed by gamma-secretase that cleaves C99 within a narrow spatial window and generates peptides 39–43 (Aβ39-Aβ43) amino acid residues long (with prevailing species Aβ40 and Aβ42).

The same year, 1987, subsequent to publication of the nucleotide sequence of human AβPP cDNA, two researchers, Breimer and Denny, noticed that the amino acid residue in position 671 of AβPP (i.e., the position contiguously preceding the C99 segment of AβPP) is methionine [59], referred to henceforth as Met671. They also noted that the AUG codon encoding Met671 of human AβPP is positioned within the optimal translation initiation nucleotide context (known as Kozak motif or Kozak consensus sequence, after Merilyn Kozak who discovered it). In its own right, such localization is remarkable due to its rarity. Even more remarkable is the fact, also noticed by Breimer and Denny [59], that the AUG encoding Met671 of human AβPP is the only AUG codon in AβPP mRNA that is situated within the optimal translation initiation nucleotide context. Human AβPP mRNA contains twenty in-frame, methionine-encoding AUG codons, and not even the AUG encoding translation-initiating methionine is embedded within the optimal translation initiation nucleotide context. Breimer and Denny’s paper [59] happened to be published on April 1st. However, it was anything but a joke. The singularity of the location of the AUG encoding Met671 of human AβPP prompted Breimer and Denny to posit that such a propitious positioning is not random but potentially underlies a physiological function, namely the internal initiation of translation of intact AβPP mRNA, a process, they proposed, possibly induced in Alzheimer’s disease [59]. This would result in C99 (presuming the translation-initiating methionine 671 is cleaved off co-translationally by N-terminal methionine aminopeptidase, MAP, as typically occurs in translation; further discussed in Section 23 below) as the primary translation product. Thus, the C99 fragment of AβPP would be generated independently of AβPP and could, upon gamma-secretase cleavage, yield Aβ, also produced in the AβPP-independent manner [59].

## 7. Ruling out the Internal Initiation of Translation of the Intact Human AβPP mRNA

Shortly after Breimer and Denny published their proposal [59], two research groups attempted to test it [60,61]. Both employed the same rationale. If the coding region of AβPP mRNA is altered well upstream of the AUG encoding Met671, the alteration would affect the conventional translation but would not interfere with translation initiated internally from the AUG codon in question. To implement this approach, one research group introduced frame-shifting mutations upstream from the AUG codon encoding Met671 of human AβPP [60]. Another research group inserted a translation stop codon, also well upstream of the AUG encoding Met671 of human AβPP [61]. In both approaches, alteration of the human AβPP gene was expected to stop the conventional production of AβPP and, consequently, of C99 and Aβ, but not to interfere with the production of C99 (and Aβ) initiated from the AUG encoding Met671, provided it occurs. In both approaches, therefore, the production of C99 and Aβ from altered AβPP mRNA would report on the occurrence of the unconventional internal initiation of translation at the AUG encoding Met671 of AβPP. In both approaches no production of C99 and/or Aβ from the altered AβPP expression vectors was observed, and both groups declared the possibility of the internal initiation of translation from the AUG codon in question as “ruled out” [60,61] (further discussed below; see Section 22).

## 8. Initiation of Translation from the AUG Encoding Met671 of Human AβPP Remains a Viable Possibility

Conceptually, however, the occurrence of the in-frame AUG codon singularly positioned within the optimal translation initiation nucleotide context and contiguously preceding the C99-encoding segment of human AβPP mRNA opens up another possibility of generating C99 (and, subsequently, Aβ) independently of AβPP. The solution is rather simple: generate a 5′-truncated AβPP mRNA where the AUG codon encoding Met671 of human AβPP is the first, 5′-most translation initiation codon, and translation of C99 independently of AβPP would occur conventionally. The key question in this regard is: Does such a process occur in Alzheimer’s disease? The answer is, apparently, affirmative, and the discovery of a mechanism capable of accomplishing this constitutes a part of our story. This mechanism has been described in detail elsewhere [58] and is briefly summarized in Section 10, Section 11, Section 12, Section 13 and Section 14 below. However, to keep the narrative in chronological order, we first describe, in the following section, a puzzling observation that was not understood at the time it was made, but later played a pivotal role in the presently narrated story.

## 9. 3′-Extended Human AβPP cDNA: An Artifact or a Clue?

The next event in our narrative occurs in 1988. This is the year when a paper by Mita and co-workers was published [62] describing human AβPP cDNA that differed in its nucleotide sequence from human AβPP cDNAs described initially by other three laboratories and referred to henceforth as “conventional” human AβPP cDNA [19,20,21]. It is, in fact, identical to the conventional cDNAs except it contains a substantial extension at its 3′ end [62]. At the time of its introduction the genomic nucleotide sequence upstream from the conventionally defined human AβPP gene was not yet known, and the authors of [62] suggested that the 3′-extended human AβPP cDNA originated as a complement of corresponding human AβPP mRNA initiated from a transcription start site (TSS) positioned well upstream from the “conventional” TSS of the human AβPP gene [62]. Later the same year, however, the nucleotide sequence upstream of the conventional TSS of the human AβPP gene was determined and published [63], and it ruled out the proposed origin of the observed 3′-extended cDNA as a transcript of human AβPP mRNA initiated upstream from the conventional TSS; no such mRNA species occurs. Lacking a plausible explanation of their results, the authors of [62] declared their observation an artifact and corrected their paper accordingly [62]. Several years later, however, when a phenomenon of physiologically occurring mammalian RNA-dependent mRNA was discovered and characterized (see the following section), this observation provided a clue and, potentially, a proof of concept, for the cellular mechanism capable of producing 5′-truncated human AβPP mRNA where the AUG codon encoding Met671 of AβPP is the first, 5′-most translation initiation codon (see Section 13 below). The general outlines of mammalian RNA-dependent mRNA amplification are sketched out in the following section.

## 10. Mammalian RNA-Dependent mRNA Amplification: General Outline

The next development pertinent to our story takes place in 1996. This is the year when the physiological occurrence of the RNA-dependent amplification of mammalian mRNA was posited [64] and further developed in subsequent publications [58,65,66,67,68,69,70,71,72,73]. As follows from its designation, this process is capable of amplifying mRNA molecules, i.e., of utilizing existing mRNA molecules as templates to generate new ones. It is of the utmost relevance to AD because, potentially, it is capable of generating a version of the original gene-transcribed mRNA molecule (referred to henceforth as mRNA progenitor) 5′-truncated within its coding region and thus encoding only a CTF of the original polypeptide; in other words, this mechanism is conceptually capable of generating a variant of AβPP mRNA that, given the availability of in-frame AUG contiguously preceding the C99-encoding segment of AβPP mRNA and provided this AUG is the first translation initiation codon, can be translated into C99 thus producing this polypeptide independently of AβPP.

Mammalian RNA-dependent mRNA amplification was first discovered in differentiating erythropoietic cells where it is responsible for the large-scale production of globin polypeptides [65,66]. Subsequently, this process was shown to underlie the generation of vast amounts of matrix proteins during their extracellular deposition [67]. In this process every eligible gene-transcribed mRNA molecule (“mRNA progenitor”) is utilized repeatedly as a template for generation of additional mRNA molecules. Since mRNA progenitors can number hundreds, in some cases thousands of molecules per cell, RNA-dependent mRNA amplification is equivalent to a massive gene amplification; its utilization in production of specific proteins could be orders of magnitude more efficient than conventional cellular protein synthesis. This process was shown to occur in two distinct phases. The first phase, referred to as “chimeric” for the reasons articulated below, is of especial interest in terms of its relevance for Alzheimer disease because it can result in a severely 5′-truncated mRNA end product encoding only a CTF portion of the original protein. This phase is outlined in the present and subsequent sections. Upon completion of the first phase of mRNA amplification, one of the two resulting end products can, if properly modified, serve as a progenitor in a polymerase chain reaction (PCR) process that always produces mRNA molecules retaining the entire coding capacity of the mRNA progenitor and thus encoding the intact original polypeptide. This phase of RNA-dependent mRNA amplification is described, and its relevance to Alzheimer’s disease explained, in Section 79 below.

**Figure 1:**
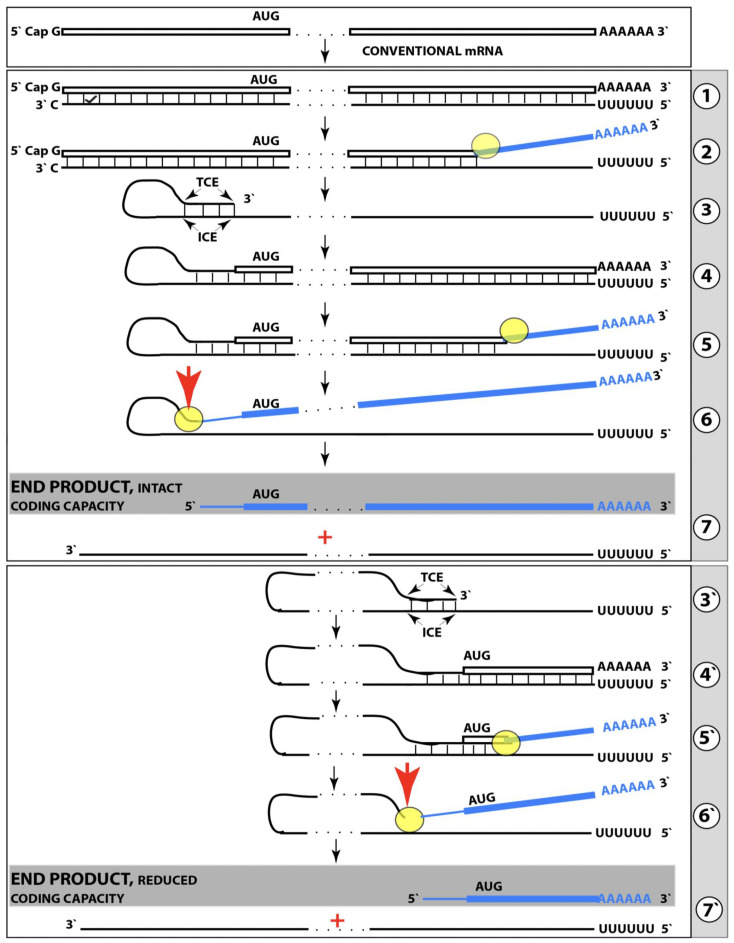
**Mammalian RNA-dependent mRNA amplification: Principal stages.** *Single lines*: Antisense RNA. *Boxed lines*: Sense RNA. *Blue boxed lines*: Single-stranded RNA separated from its complementary RNA strand by helicase. *Yellow circles*: Helicase complex containing helicase strand-separating activity, nucleotide modifying activity and RNA cleaving activity. *Red arrows*: positions of the cleavage of the intermediate generating RNA end products of the amplification process. *RdRp*: RNA-dependent RNA polymerase. *AUG*: Translation initiation codon. *TCE*: 3′ Terminal Complementary Element of the antisense RNA strands. *ICE*: Internal Complementary Element of the antisense RNA strands. *Top panel*: Conventionally transcribed mRNA molecule referred to as progenitor mRNA. *Middle panel*: Stages of the chimeric pathway of the RNA-dependent mRNA amplification; the ICE is located within a portion of antisense RNA corresponding to the 5′ UTR of the progenitor mRNA molecule. (**1**): The progenitor mRNA is transcribed by RdRp into antisense RNA. (**2**): Complementary RNA strands are separated by the helicase activity. Helicase complex mounts the 3′ poly(A) segment and moves along the sense RNA strand modifying on average ever fifth nucleotide. (**3**): Folding of the antisense RNA into self-priming configuration is guided by the interaction of its TCE and ICE elements. (**4**): RdRp extends the 3′ terminus of self-primed antisense RNA. This produces a hairpin-like molecule containing both sense and antisense RNA components and referred to as the chimeric RNA intermediate. (**5**): Complementary strands of the chimeric RNA intermediate are separated by the helicase activity. Nucleotide modifications introduced during the separation prevent the re-annealing of the sense and antisense RNA strands. (**6**): When the helicase reaches the single-stranded portion of he chimeric intermediate (either the 5′ end of the TCE or a TCE/ICE mismatch) it cleaves the RNA molecule. (**7**): End products of RNA-dependent mRNA amplification. Antisense RNA is truncated at its 3′ end and sense portion of the chimeric RNA is truncated at the 5′ end and acquires the cleaved-off antisense RNA fragment; its translation would result in the complete original polypeptide. *Bottom panel*: (**3′**–**7′**) correspond to (**3**–**7**) of the middle panel. The ICE element is situated within a segment of antisense RNA corresponding to the coding region of the progenitor mRNA. The chimeric RNA end product is 5′-truncated within its coding region. Potential outcome of its translation are described in the main text. Please note that this figure was shown in [10].

Stages of the chimeric phase of mammalian RNA-dependent mRNA amplification are outlined in Figure 1. For the purposes of orientation, the progenitor mRNA (also referred to as conventional mRNA) is shown in the top panel; its elements of importance are its termini and the translation-initiating AUG codon (marked “AUG”). In the first stage of the chimeric phase of mRNA amplification (marked “1” in the middle panel of Figure 1) RNA-dependent RNA polymerase (RdRp) initiates transcription of the progenitor mRNA molecule within its 3′-terminal poly(A) segment and completes it with transcribing the 5′-terminal cap-G into “C”, which is not encoded in the genome.

In the second stage of the chimeric phase of mRNA amplification (marked “2” in the middle panel of Figure 1) a helicase complex separates the sense and antisense RNA strands. It mounts the 3′-terminal poly(A) segment in double-stranded configuration with its 5″-terminal poly(U) complement and proceeds in the 5′ direction separating the strands and modifying on average every fifth nucleotide of the sense strand thus preventing the re-annealing of the strands. When separation is completed the sense strand (the progenitor mRNA molecule) can be utilized again, repeatedly, in additional amplification rounds.

The feasibility of the third stage of the chimeric phase of mRNA amplification (marked “3” in the middle panel of Figure 1) depends vitally on two requirements. The first requirement is the presence within the antisense RNA strand of two complementary segments. One is the 3′-terminal complementary element, designated TCE; it is always, by definition, 3′-terminal. The second element can be positioned anywhere within the antisense RNA strand, it is designated the internal complementary element, ICE. The complementation of the TCE and ICE does not have to be perfect, just sufficient to form a stable structure. It is important to mention that in the scenario depicted in the middle panel of Figure 1 the ICE element is located within the portion of antisense RNA corresponding to the 5′ untranslated region (5′UTR) of the mRNA progenitor molecule. The second requirement is that the TCE and ICE are topographically compatible, in other words mutually accessible within the folded configuration of the antisense RNA strand. If both requirements are satisfied, the folding of the antisense RNA strand results in an RNA molecule in self-primed conformation.

In the fourth stage of the chimeric phase of mRNA amplification (marked “4” in the middle panel of Figure 1), RdRp extends the 3′ terminus of the self-primed antisense RNA strand. During this extension, RdRp transcribes the 5′-terminal portion of the antisense RNA strand, thus generating the sense RNA strand. The extension concludes with synthesis of 3′-terminal poly(A) and results in a 5′-truncated portion of the progenitor mRNA covalently attached to the 3′ terminus of the antisense RNA strand in a hairpin-like configuration. Since this molecule consists of both sense and antisense components, it is chimeric, which explains the designation of this phase as the chimeric phase of mRNA amplification. Since this molecule is an intermediate in the amplification process, it is referred to as “the chimeric RNA intermediate” and the site of the initiation of the extension (i.e., of the transition of the antisense into sense orientation) is designated “the chimeric junction”. Importantly, since in the depicted scenario the ICE element of the antisense RNA strand is positioned within its portion corresponding to the 5′UTR of the mRNA progenitor molecule, the sense orientation component of the chimeric RNA intermediate retains the entire coding region of the progenitor, including the translation-initiating AUG codon.

In the fifth stage of the chimeric phase of mRNA amplification (marked “5” in the middle panel of Figure 1), the same helicase complex that was invoked above mounts the 3′-terminal poly(A) segment of the sense-orientation component of the chimeric RNA intermediate and moves along it, separating RNA strands and making frequent nucleotide modifications thus preventing re-annealing of the strands. In the sixth stage (marked “6” in the middle panel of Figure 1) the helicase complex reaches the single-stranded portion of a hairpin structure. At this point there are two possibilities. First, the TCE/ICE structure has no mismatches. In this case, a component of the helicase complex or an associated activity cleaves at the 5′ end of the TCE. In the second possibility there is a mismatch or multiple mismatches in the TCE/ICE structure. In this case, the helicase complex cleaves at the mismatch. Here, again, there are two possibilities. If the relationship of the remaining 5′ portion of the TCE with the corresponding segment of the ICE is unstable, this is the end of the process. If, however, following the cleavage, the self-primed conformation of the folded antisense RNA remains stable, the extension could reoccur, and the process repeated. The chimeric junction in this case would shift 5′ from the initial chimeric junction, a process designated “chimeric junction shift” [58]; with multiple TCE/ICE mismatches this can take place more than once until the extension can no longer occur.

The seventh stage (marked “7” in the middle panel of Figure 1) depicts two RNA end products of the chimeric phase of mRNA amplification. One end product is the antisense RNA or rather what remains of it because it is truncated in its 3′ portion. More precisely, it has lost its entire TCE element (if the TCE and ICE had no mismatches) or a part thereof (if the cleavage occurred at the TCE/ICE mismatch). It cannot any longer form a self-primed structure. Its further processing amounts to an intracellular polymerase chain reaction and is described in Section 79 below.

Another end product is mRNA. It is distinct from the progenitor mRNA molecule in two ways. First, it is chimeric. It comprises the sense-orientation portion, which acquires at its 5′ end a portion of the antisense RNA molecule, more precisely the same portion that was cleaved off antisense RNA in the sixth stage. The second difference is that its sense-orientation portion is 5′-truncated (in comparison with the progenitor mRNA). However, because (in this scenario) the ICE is located within the portion of the antisense strand corresponding to the 5′UTR of the progenitor mRNA, the truncation removes only a terminal part of the 5′UTR. Thus, the chimeric RNA end product retains the intact coding region and can be translated into the polypeptide identical to that encoded by the progenitor mRNA.

## 11. Chimeric mRNA Amplification Can Occur Asymmetrically and Result in mRNA Encoding Only a CTF of the Progenitor mRNA-Encoded Protein

In the scenario presented in the preceding section the ICE element of the antisense RNA strand is positioned within its portion corresponding to the 5′UTR of the progenitor mRNA molecule. Consequently, the resulting chimeric mRNA end product retains the entire coding region of and is translated into a polypeptide identical to that encoded by the progenitor mRNA. But this is only one option of many. Whereas the position of the TCE element of the antisense RNA strand is fixed (it is always 3′-terminal by definition), the position of the ICE element is highly variable; potentially it can be localized anywhere within the antisense RNA strand. The positioning of the ICE defines the potential of the chimeric RNA end product to produce, upon translation, a polypeptide, and can lead to several interesting translational outcomes (discussed in detail in [58]).

The present section addresses the scenario where the ICE element of the antisense RNA strand is positioned within its portion corresponding to the coding region of the progenitor mRNA molecule. This scenario is illustrated in the bottom panel of Figure 1. In this scenario stages 3′ through 7′ correspond and are conceptually identical to stages 3 through 7 described in the preceding section. The final outcome, however, is substantially different. Due to the position of the ICE element, only a 3′-terminal portion of the coding region (and the rest of mRNA) would be transcribed from the antisense RNA template. As depicted in stage 7′, following separation of strands and the cleavage, one of the end products, namely the antisense RNA, would be very similar in its structure to that depicted in stage 7. It would be missing at its 3′ end only the TCE element or a part thereof. The chimeric RNA end product, however, would be very different from its counterpart shown in stage 7 in that its coding region would be 5′-truncated. The translation outcome for this chimeric RNA end product would depend on the position of the first functional translation initiation codon (marked “AUG”, not to be confused with the “AUG” in the top and middle panels of Figure 1). If it is located within the truncated coding region and, if it is in-frame with the coding nucleotide sequence, translation of the chimeric RNA end product would yield a CTF of the progenitor mRNA-encoded polypeptide. Since only the 3′-terminal portion of the progenitor mRNA is amplified, which encodes only the C-terminal portion of the original protein, this process is referred to as “asymmetric” amplification.

## 12. Evaluating the Eligibility of an mRNA Species for the RNA-Dependent Amplification Process

Implications of the asymmetric RNA-dependent mRNA amplification and their potentially considerable relevance to Alzheimer’s disease are apparent. If human AβPP mRNA can be amplified asymmetrically and if in the resulting chimeric RNA end product the first translation initiation codon happens to be the AUG encoding Met671 of AβPP, the C99 fragment would be produced, and produced at a high rate, independently of AβPP. However, the probability that human AβPP mRNA is eligible for such an asymmetric RNA-dependent amplification process is, ostensibly, rather small if not negligible. Indeed, the AUG encoding Met671 of human AβPP is located over two thousand nucleotides downstream from the 5′ terminus of AβPP mRNA. Since the segment of mRNA encoding the ICE of the antisense strand has to be in the proximity of the AUG encoding Met671, the TCE and ICE elements would also be separated by about two thousand nucleotides. First, however these elements have to be present in the AβPP antisense RNA, and this is not a given. Second, crucially, these elements (if they occur in the first place) must be mutually accessible in the folded conformation of the antisense RNA strand, a tall order considering the extent of their separation and the complexity of RNA folding. Fortunately, the occurrence of the TCE and ICE elements and their mutual accessibility in the folded antisense RNA strand can be assessed (for any mRNA species) experimentally.

In such an experiment a specific mRNA is used as the initial template, and RdRp is modeled by RNA-dependent DNA polymerase, RdDp (also referred to as reverse transcriptase, RT). Oligo(dT) is used as a primer to initiate transcription of the antisense strand (cDNA) from the 3′-terminal poly (A) segment of mRNA. It is essential for this experiment that RNAse H is included in the reaction. It models the helicase activity of the mRNA amplification process and removes mRNA strand following the completion of reverse transcription thus enabling the folding of cDNA. There are two ways to include RNase H activity into the reaction. One, the simplest, is to use a viral preparation of RdDp, which always contains RNase H activity. Another way is to use cloned RdDp, which lacks RNase H activity, and add the latter to the reaction mix. When the reverse transcription and the removal of RNA template are completed, the newly generated antisense strand (cDNA) is folded. If it contains the TCE and ICE elements and if these elements are mutually accessible, a self-primed structure will be formed, and its 3′ end will be extended by RdDp generating a 3′-terminal segment of the sense strand. Size analysis of the resulting cDNA would determine (by comparison with the size of its mRNA template) whether the extension occurred, and, if affirmative, the nucleotide sequencing of cDNA would identify the TCE and ICE elements and define the position of the ICE.

## 13. Validation (1): Human AβPP mRNA Is a Legitimate Template for the Asymmetric RNA-Dependent Amplification; The Chimeric RNA End Product Encodes the C99 Fragment of AβPP

The scenario presented in the preceding section describes precisely the outcome of the experiment by Mita and co-workers [62] described in Section 9 above. As the reader might remember, for the lack of an explanation, Mita et al. defined their results (a substantially 3′-extended human AβPP cDNA) as an artifact [62]. However, upon further analysis of the structure and nucleotide sequence of the observed 3′-extended AβPP cDNA it transpired that the extension seen in [62] is not random but, in fact, a 3′-terminal portion of the sense-orientation AβPP DNA strand and that it could be generated solely by the antisense self-priming mechanism described above [74]. The question arises: why this extension occurred in Mita et al. experiment [62] but not in studies of three laboratories [19,20,21] who initially determined the nucleotide sequence of human AβPP cDNA? The answer is trivial and technical: Mita et al. used viral preparation of RdDp, which contained RNase H activity required for the removal of the mRNA template (i.e., for strands separation). In contrast, other groups utilized more “advanced” (and expensive) cloned preparations of RdDp lacking RNase H activity; without the removal of the mRNA template no cDNA folding could occur.

**Figure 2:**
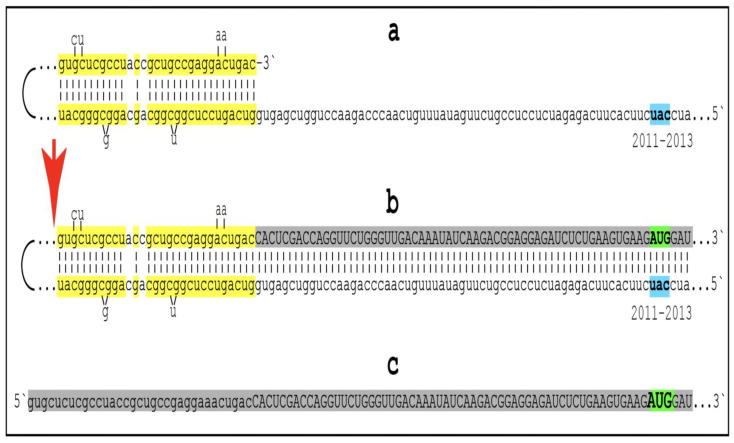
**Human AβPP mRNA Is a Legitimate Template for the Asymmetric RNA-dependent Amplification.** *Small letters*: nucleotide sequences of the relevant segments of the human antisense AβPP RNA molecule. *Large letters*: nucleotide sequence of a portion of human AβPP mRNA produced by the extension of the 3′ end of self-primed antisense RNA. *Highlighted in yellow*: the TCE and ICE components of the human antisense AβPP RNA. *2011–2013*: Positions counted in numbers of nucleotides from the 3′ terminus of the human AβPP antisense RNA. Nucleotides at these positions form the “uac” (*shown in blue*) corresponding to the “AUG” (*shown in green*) encoding Met671 of human AβPP mRNA. Segments (**a**) to (**c**) correspond to stages 3′ to 7′ of Figure 1. (**a**): Folding of human antisense AβPP RNA into self-primed structure is guided by the interaction of its TCE and ICE components. (**b**): The extension of the 3′ end of self-primed human antisense AβPP RNA produces 3′-terminal portion of AβPP RNA mRNA (*shown in gray*). Following the separation of strands by the helicase complex the hairpin-like intermediate is cleaved. (**c**) The cleavage (denoted by red arrow) produces the chimeric RNA end product (*shown in gray*). Its sense orientation portion is truncated deep within the coding region of AβPP mRNA; its translation would start from the AUG encoding Met 671 of AβPP and produce the C99 fragment independently of AβPP. Please note that this figure was shown in [10].

As discussed above, the determination of the nucleotide sequence of the 3′-extended human AβPP cDNA identified the TCE and ICE elements of the antisense strand, defined the position of the ICE element, and established the location of the site of initiation of the extension (“the chimeric junction site” in the terminology utilized above). Importantly, it also established the identity and location of the first translation initiation codon within the extended segment: It occurs 58 nucleotides into the extension portion, and it is the AUG encoding Met671 of AβPP! Figure 2 depicts the projected pivotal stages of the chimeric pathway of asymmetric RNA-dependent amplification of human AβPP mRNA with panels (a) to (c) paralleling stages 3′ to 7′ of Figure 1. Panel (a) of Figure 2 depicts folded self-primed conformation of the antisense human AβPP RNA following its separation from the progenitor AβPP mRNA by a helicase activity. The TCE and ICE elements, separated by about two thousand nucleotides, are highlighted in yellow. Panel (b) of Figure 2 depicts the extension of the 3′ terminus of self-primed antisense RNA resulting in formation of the chimeric RNA intermediate. The extension portion is highlighted in gray. The first translation initiation codon within the extension portion is highlighted in green; it happens to be the AUG encoding Met 671 of human AβPP mRNA. Following strands separation by a helicase activity, cleavage within the chimeric RNA intermediate (denoted by red arrow) can occur either at one of the TCE/ICE nucleotide mismatches or, as shown in Panel (b), at the 5′ end of the TCE element. Panel (c) of Figure 2 shows the resulting chimeric RNA end product. Its 5′-terminal antisense portion is shown in small letters. Its sense-orientation portion is severely 5′-truncated within the coding region of the progenitor AβPP mRNA. Its first translation initiation codon is the AUG encoding Met671 of human AβPP; conventional initiation of translation from this codon would yield the C99 fragment generated independently of AβPP. This analysis was performed and published in 1996 [74], before being subsequently expanded upon in further publications [75,76,77].

## 14. Validation (2): The Major Prediction Relevant to AβPP-Independent Production of C99 in Alzheimer’s Disease Has Been Confirmed in a “Dream Experiment”

How to validate that AβPP-independent production of C99 indeed occurs in and, moreover, drives Alzheimer’s disease? Through testing key predictions of this notion. The quantity of C99 produced via the amplification of AβPP mRNA in a process analogous to a massive gene amplification would greatly outbalance that of C99 generated in the AβPP proteolytic pathway. Therefore, with the AβPP mRNA amplification process operational, inhibition of beta-site cleavage by beta-secretase would be completely inconsequential and futile as a therapeutic strategy in AD. This is the major prediction. Testing it at the time when the concept was formulated, in 1996 [74], would require a major “dream experiment”. A “dream” not only because the identity of beta-secretase was not yet established but also because it would require the development and testing of a drug, a financial impossibility for an academic laboratory, and experimentation on human patients (clinical trials).

Enter Big Pharma. In 1996, following the publication of [74], Merck invited one of the authors (V.V.) to make a presentation at their headquarters in King of Prussia (a town in Pennsylvania, USA). At the end of the presentation, they asked what would be a key one-line advice regarding the therapeutic strategies for AD. The answer was: do not pursue the inhibition of beta-secretase; it would be futile. Merck did not heed this advice. By 1999 the identity of beta-secretase (BACE1) was established [78,79,80] and the race to develop BACE1 inhibitors was on. It took over a decade and Merck won this race with the development of verubecestat, a potent, efficient, and brain (and neurons)-penetrating BACE1 inhibitor. The first promising results in animal models were obtained around 2007 [81,82,83,84,85,86,87,88,89], and in 2012 Merck singled on the BACE1 inhibitor designated “compound 16” [90]. Further improvements of “compound 16” resulted in verubecestat [91]. Verubecestat performed brilliantly in animal models and in early human safety trials [91], and the hopes associated with its potential use as a drug were considerable. The final test came in the form of two massive stage-III human clinical trials initiated in 2016: one for mild-to-moderate AD and another for prodromal AD [27,28]. By 2018 both trials were terminated, both prematurely, due to the complete lack of efficacy (the lack of efficacy was solely in therapeutic outcomes, mechanistically verubecestat performed in humans as effectively, if not more so, as in animal models) [27,28]. The development of verubecestat and its trials, at a combined cost of over billion dollars, constituted the “dream experiment”, referred to above, and validated the notion of AβPP-independent generation of C99 in Alzheimer’s disease.

## 15. Validation (3): Identifying the Chimeric RNA Intermediates of RNA-Dependent Human AβPP mRNA Amplification

The aim of the present section is to define precisely the nucleotide sequences to search for in order to validate the occurrence of chimeric AβPP RNA molecules in AD-affected neurons. The chimeric nature of these RNA molecules is their unique feature, and it is epitomized in the occurrence of the chimeric junction, which is a site where the antisense nucleotide sequence transits into the sense orientation. Therefore, the way to validate the occurrence of chimeric AβPP RNA molecules is to identify and sequence their regions containing both the sense and antisense components. Since the chimeric RNA end product of human AβPP mRNA amplification, containing sense/antisense junction, has to be, by definition, highly abundant, it would appear that detecting and sequencing it is simple. This, however, is not the case. When the helicase complex separates complementary segments of the chimeric RNA intermediate, as shown in Figure 1 above, it mounts the 3′-terminal poly(A) and moves along the molecule separating it from its complement. It also modifies on average every fifth nucleotide; when the newly generated chimeric RNA end product is cleaved off the chimeric RNA intermediate, it is modified along its entire length. The purpose of these modifications is, apparently, to prevent the re-annealing of separated strands and, possibly, to enable the translation of the resulting cap-less mRNA (further discussed below). Since these modifications prevent hybridization of the modified RNA with its complement, it cannot be analyzed by techniques involving nucleic acid hybridization and cDNA-based sequencing. These molecules may be ubiquitous, but they are “invisible” to current methods of detection [58]. What can be detected, however, are chimeric junction-containing regions of the chimeric RNA intermediate. This is because when the junction is generated by the extension of self-primed antisense AβPP RNA, it remains unmodified for the duration of the extension process plus the time it takes the helicase complex to traverse the length of the sense-orientation RNA strand.

**Figure 3:**
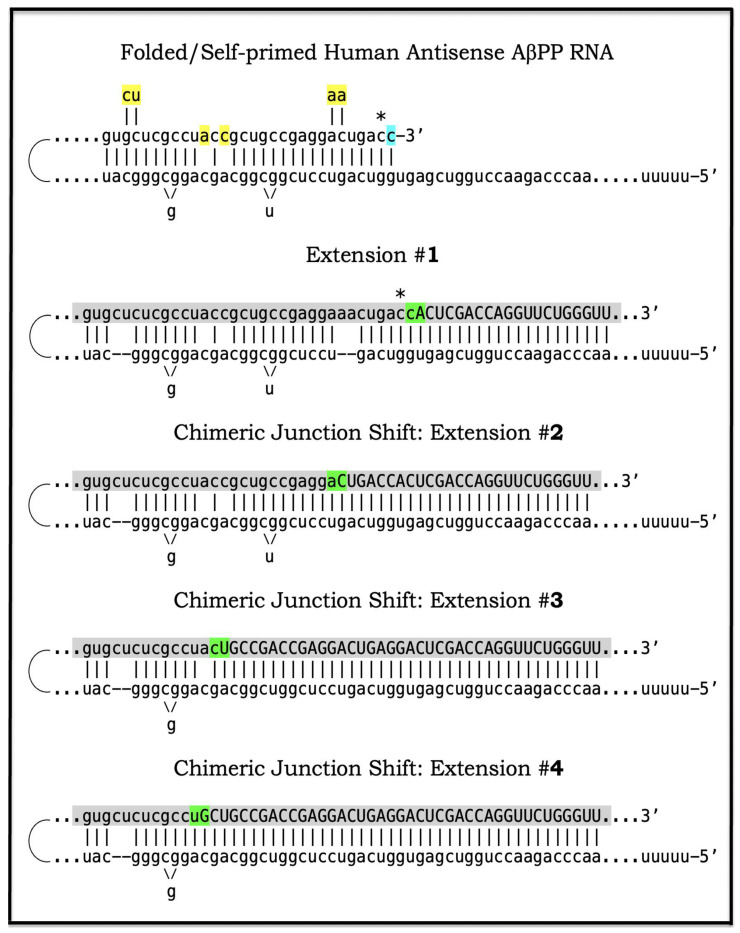
**Anticipated nucleotide sequences of chimeric junction regions of chimeric human AβPP mRNA amplification intermediates**. *Small letters*: Nucleotide sequences of the relevant portions of self-primed antisense AβPP RNA. *Large letters*: Nucleotide sequences of sense RNA generated by the extension of self-primed human AβPP RNA. *Yellow boxes*: Mismatches within the TCE/ICE double-stranded complex. *Green boxes*: Chimeric junctions consisting of the 3′-terminal nucleotide of antisense RNA and 5′-terminal nucleotide of the sense RNA strand. *Asterisk*: The nucleotide of the antisense RNA molecule corresponding to the transcription start site positioned 149 nucleotides upstream from the translation-initiating AUG codon. *Shown in blue*: “c” transcribed from the 5′-terminal cap “G” of AβPP mRNA; it is accommodated within the self primed antisense RNA. *Shown in gray*: anticipated nucleotide sequences of the regions containing chimeric junctions. *Extension #1* of the self-primed RNA generates chimeric RNA intermediate containing the full-size TCE element of antisense RNA. *Extension #2*: Cleavage of the chimeric RNA intermediate occurs at the first TCE/ICE mismatch. Self-primed antisense RNA configuration remains stable and is extended; the chimeric junction shifts upstream from the original one. *Extension #3*: Cleavage of the chimeric RNA intermediate occurs at the second TCE/ICE mismatch. Self-primed antisense RNA configuration remains stable and is extended again; the chimeric junction shifts upstream. *Extension #4*: Cleavage of the chimeric RNA intermediate occurs at the third TCE/ICE mismatch. Self-primed antisense RNA conformation remains stable and is extended once more; the chimeric junction shifts as shown.

The nucleotide sequence of one validation target is easy to define. It is shown in Figure 3 as a segment of its “Extension #1” portion highlighted in gray. It contains the 3′ terminus of self-primed AβPP antisense RNA extended into the sense-orientation molecule. But there could be additional sense/antisense junctions. This is because the helicase complex cleaves the chimeric RNA intermediate when it reaches its single-stranded portion, and, therefore, the cleavage can occur at a mismatch within the TCE/ICE complex. If, following such cleavage, the self-primed antisense RNA structure (i.e., the remaining portion of the TCE/ICE complex) remains stable, it can be extended again, as shown in the “Extension #2” portion of Figure 3. In such a case, a new chimeric junction is generated but it is shifted upstream from the initial one. Such a process is designated “chimeric junction shift” [58] and it can be repeated several times until, in fact, the remaining portion of the TCE/ICE complex is no longer stable. As shown in Figure 1, in case of human AβPP RNA it can occur possibly four times, every time generating new chimeric junction and defining new junction-containing regions. Nucleotide sequences of these regions (validation targets) are highlighted in gray in the “Extension #1” through “Extension #4” portions of Figure 3. These validation targets can be searched for in human neuronal cell-based AD models (discussed below).

## 16. Mammalian RNA-Dependent mRNA Amplification Is Enabled and Sustained by the Integrated Stress Response

**Figure 4:**
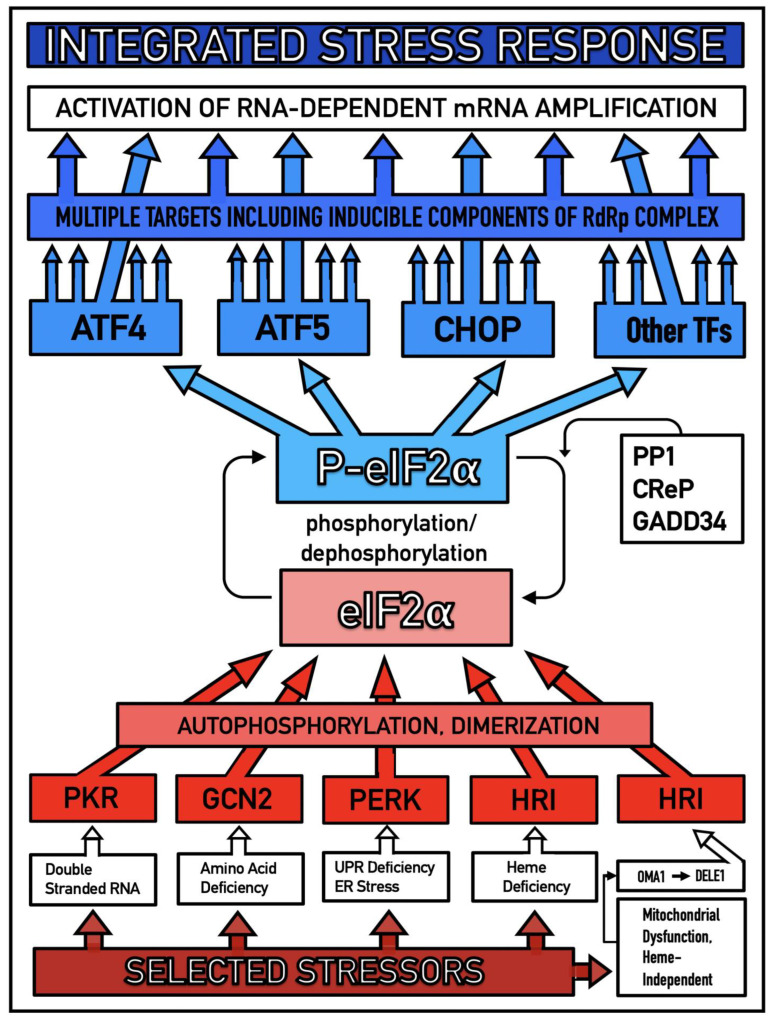
**Mammalian RNA-dependent mRNA amplification is enabled by the integrated stress response.** Distinct stressors selectively named in the Figure activate, in a stressor-specific manner, a subset (one or more) of eIF2α kinases: PKR, GCN2, PERK, and HRI; the activation process entails autophosphorylation and dimerization of the kinases. When activated, kinases phosphorylate eIF2α at its Ser51 residue; this is the event, which integrates a multitude of signaling pathways into a single outcome; it is the essence of the integrated stress response pathway. When elicited, the ISR radically rearranges the transcriptional and translational landscapes of the cell. Global cellular protein production is severely inhibited, primarily through suppression of cap-dependent initiation of translation. Concurrently, the ISR activates cap-independent translation of a small set of selected mRNA species, including those that encode various transcription factors. Among the proteins whose production was enabled by the ISR are essential components of RdRp, not present under regular, non-ISR, conditions. Thus, ISR enables the operation of the mammalian RNA-dependent RNA amplification pathway.

Previous investigations of mammalian RNA-dependent mRNA amplification suggested that this process is enabled and sustained by the integrated stress response (ISR) [58]. This pathway is activated in response to multiple stresses, and it is designated “integrated” because the effects of a multitude of stimuli/stressors, both physiological and pathological as well as environmental, capable of eliciting the ISR, converge on a single (“integrating”) event, namely the phosphorylation of eukaryotic translation initiation factor 2 alpha (eIF2α) at its residue Ser51 [92,93,94,95,96,97,98,99,100,101]. This event is enacted by one of four constituent members of the family of eIF2α kinases: protein kinase double-stranded RNA-dependent (PKR), PKR-like endoplasmic reticulum kinase (PERK), general control non-derepressible-2 kinase (GCN2), and heme-regulated inhibitor kinase (HRI). These four kinases are extensively homological in their catalytic domains but possess distinct regulatory domains. They all operate in a similar manner involving autophosphorylation and dimerization to acquire their full activity. Each kinase, however, responds to a defined (albeit somewhat overlapping) set of stressors. Thus, PERK, which is localized at the endoplasmic reticulum (ER), is activated by ER stresses but also by ATP depletion, deprivation of glucose, and activation of various oncogenes. GCN2 responds to amino acid deprivation but also to UV and other stimuli. PKR responds to double-stranded RNA (viral infections) but also to oxidative stress, growth factor depletion, and caspase activity among other stimuli. HRI responds to heme deficiency but also to heat shock, proteasome inhibition, nitric oxide, osmotic stress, and mitochondrial dysfunction. When elicited, the ISR radically rearranges the transcriptional and translational landscapes of the cell. Global cellular protein production is severely inhibited, primarily through suppression of cap-dependent initiation of translation. Concurrently, the ISR activates cap-independent translation of a small set of selected mRNA species, including those that encode various transcription factors. It was suggested [58] that among the proteins whose production is enabled by the ISR are essential components of RdRp, not present under regular, non-ISR, conditions. This process is illustrated in Figure 4.

In studied cases the operation of the RNA-dependent mRNA amplification pathway propagates ISR conditions and thus sustains its own activity. For example, during erythroid differentiation, the increased production of globin chains results in sequestration of heme in tetrameric hemoglobin complexes. This depletes cellular heme, activates HRI, and elicits the ISR. Under the ISR conditions RdRp is activated, and globin mRNA is amplified [66]. The resulting “turbocharged” production of globin chains further depletes heme, maintains the ISR conditions, and sustains, via the activity of RNA-dependent globin mRNA amplification pathway, its own operation. Similarly, in cells producing and depositing extracellular matrix (ECM) proteins, their elevated conventional production induces the ER stress and thus activates PERK. PERK, in turn, phosphorylates eIF2α and elicits the ISR. Under ISR conditions, RdRp is activated and mRNA species encoding ECM proteins are amplified [67]. This results in substantially increased production of ECM proteins. Consequently, PERK activity is maintained, ISR conditions propagated, and the operation of RNA-dependent amplification of mRNA encoding ECM proteins perpetualized. As described in more details in Section 26 below, in conventional Alzheimer’s disease the neuronal ISR is elicited and AβPP-independent pathway of C99 production, presumably via asymmetric RNA-dependent amplification of AβPP mRNA, is activated by AβPP-derived iAβ accumulated over a certain critical threshold. This pathway, in turn, propagates ISR conditions and thus perpetuates its own operation.

## 17. Alzheimer’s Is a Species-Specific, Possibly Uniquely Human Disease

**Figure 5:**
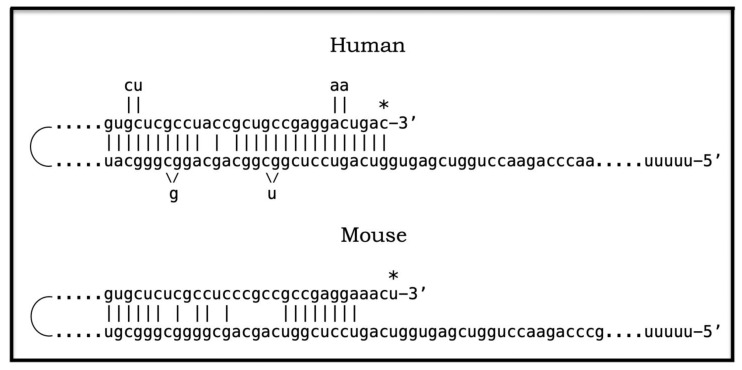
**Mouse AβPP mRNA is not a legitimate template for RNA-dependent mRNA amplification** *“Human”*: human antisense AβPP RNA folded into the self-primed configuration. *“Mouse”*: The relationship between segments of the mouse antisense AβPP RNA molecule analogous to that of the human TCE and ICE elements. *Asterisk*: The nucleotide of the antisense RNA molecule corresponding to the transcription-initiating nucleotide of either human or mouse AβPP mRNA; in both human and mouse transcription of 149 upstream from the AUG translation initiation codon. There is no better than random complementarity between segments of mouse antisense AβPP RNA corresponding to the TCE and ICE elements of its human counterpart, and the 3′ overhang would effectively prevent the priming/extension. The possibility that the ICE of mouse antisense AβPP RNA is positioned somewhere else in the molecule was excluded by the blast analysis of its 3′-terminal portion, with the rest of the molecule showing no significant complementarity anywhere. Mouse AβPP mRNA is, therefore, not a legitimate template for RNA-dependent amplification.

To date, Alzheimer’s disease has been detected solely in humans. It could be argued that in other species the limited lifespan does not allow AD to develop. This, apparently, is not the reason: the disease was not seen, for example, in elephants despite their longevity (over 80 years). The potential involvement of RNA-dependent amplification of AβPP mRNA explains why: in most, if not all, non-human species AβPP mRNA is ineligible for RNA-dependent amplification described above. Since mice are often used in attempts to model AD, it is prudent to analyze them in this respect as an example. As discussed above, for an mRNA species to be an eligible template for the RNA-dependent mRNA amplification process, its antisense complement must satisfy two requirements: (1) it should possess the TCE and ICE elements and (2) these elements should be mutually accessible in the folded antisense RNA molecule. However, as illustrated in Figure 5 (shown in comparison with human antisense AβPP RNA), even the first requirement is not satisfied in mouse antisense AβPP RNA. There is no better than random complementarity between segments of mouse antisense AβPP RNA corresponding to the TCE and ICE elements of its human counterpart, and the 3′ overhang would effectively prevent the priming/extension. As for the possibility that the ICE of mouse antisense AβPP RNA is positioned somewhere else in the molecule, the blast analysis of the 3′-terminal portion with the rest of the molecule showed no significant complementarity anywhere. Mouse AβPP mRNA is, therefore, not a legitimate template for RNA-dependent amplification. For the same reasons, this conclusion extends to other non-human mammalian AβPP mRNA species. It appears that Alzheimer’s is species-specific if not uniquely human disease.

## 18. AβPP mRNA Transcribed from Human Transgenes in Animal Models Is Ineligible for RNA-Dependent Amplification

The preceding section established that in mice, as in the majority and possibly all non-human mammalian species, AβPP mRNAs cannot be amplified because their antisense counterparts lack the mutually accessible TCE and ICE elements. But human AβPP mRNA, expressed in animal models from human transgenes, is not amplified either; this is the reason why transgenic mouse models are incapable of developing the full spectrum of AD pathology. What is the reason for this failure? It could be argued that mice do not possess RNA-dependent mRNA amplification machinery, but this is not the case: they do employ a robust RNA-dependent mRNA amplification as a basic physiological tool [66,67]. This is exemplified by amplification of globin-encoding mRNAs in erythroid differentiation and of ECM proteins-encoding mRNAs during ECM deposition. Moreover, mutations, affecting (weakening) the TCE/ICE interaction in globin antisense RNA, were shown to result in various types of thalassemia [58,102]. It could also be argued that, possibly, no ISR can be elicited in mouse neurons and therefore no RNA-dependent mRNA amplification pathway can be activated. As discussed in more details in the following section, this is also not the case: iAβ, accumulated to sufficient levels, triggers the elicitation of the neuronal ISR in mice. The remaining, apparently paradoxical, answer is that somehow human AβPP mRNA is ineligible for RNA-dependent amplification in mouse neurons. This is the right answer, but it has nothing to do with mice and has everything to do with the structure of human AβPP mRNA expressed in transgenic mouse models. During the construction of human AβPP transgenes-carrying vectors the 5′ terminus of human AβPP cDNA is substantially modified for its insertion into the vector. This alters or altogether eliminates the segment encoding the TCE element of AβPP antisense RNA rendering it ineligible for RNA-dependent amplification. In this respect exogenous AβPP mRNA expressed from human transgenes is similar to endogenous mouse AβPP mRNA: both are incapable (for the same reason) of supporting their RNA-dependent amplification.

## 19. Transgenic Animals Overexpressing Human AβPP Model Not Alzheimer’s Disease but Solely Effects of the Neuronal ISR: How to Generate an Adequate Mouse Model of AD

As mentioned above and discussed in detail below (see Section 25), in Alzheimer’s disease AβPP-derived iAβ, accumulated over the critical threshold, elicits the neuronal ISR and thus activates the AβPP-independent production of C99, and, as presumed in this iteration of the ACH2.0, of iAβ that drives the disease. In transgenic mice a fraction of the vast output of human Aβ accumulates intraneuronally and triggers the elicitation of the neuronal ISR [103]. This is the integral part of the development of AD and occurs also in AD patients [104]. But in transgenic mice overexpressing human AβPP the development of AD stops at this stage because the pathway capable of generating C99 independently of AβPP is inoperative. Yet these mice display an extent of neurodegeneration and of cognitive impairment that were, until now, interpreted as symptoms of AD. It is apparent now, however, that both neurodegeneration and cognitive impairment observed in these models can be ascribed to the neuronal ISR, more specifically to the global suppression of cellular protein synthesis, which is the manifestation of the ISR. It is inevitable that the persistent neuronal ISR would, via suppression of protein production, result in cellular damage; this effect was, in fact, observed not only in neurons but in other cell types as well [105,106,107,108,109,110,111,112,113,114,115,116,117,118]. The very same neuronal ISR-caused suppression of global cellular protein production explains also cognitive impairment seen in transgenic mouse models overexpressing human AβPP. The observed impairments include defects in learning, memory formation and neuronal plasticity, i.e., in functions shown to require new neuronal protein production; it is not a surprise that the suppression of the latter causes the former [105,106,107,108,109,110,111,112,113,114,115,116,117,118]. The causal relationship between cognitive impairments in mice overexpressing human AβPP and the neuronal ISR is illustrated by observations that prevention of the neuronal ISR in these mice prevents cognitive impairment and that inhibition of the neuronal ISR (and consequent restoration of cellular protein synthesis) abrogates impairment if it already occurred [119,120,121,122,123,124,125,126,127]. The above considerations make it apparent that mice overexpressing human AβPP model not AD but rather solely the effects of the exogenous iAβ-elicited neuronal ISR.

Thus, the current mouse transgenic models overexpressing human AβPP are, patently, NOT AD models. The question arises, therefore, how does one generate adequate mouse AD models? This question is addressed in detail in our previous studies [7,10]. In short, the expression, at sufficient levels, of human AβPP mRNA eligible for the RNA-dependent mRNA amplification process would be both necessary and sufficient to achieve this goal (this is the core; appending FAD mutations in both AβPP and PSENs and expressing ApoE4 would significantly improve a model). The simplest approach to accomplish this is to utilize the current transgenic models overexpressing human AβPP: they are, after all, well on their way to becoming AD models in that they exhibit the neuronal ISR elicited by AβPP-derived iAβ; all that is needed is to enable operation of the AβPP-independent C99 production pathway. Modifying, via gene editing, human AβPP transgenes in existing transgenic models in such a way as to restore their 5′ termini would attain the latter. Another approach is to start from scratch by constructing new transgenic vectors expressing human AβPP mRNA identical in all respects to that produced endogenously in human neurons (or at least containing the intact 5′ terminus).

## 20. An Adequate Human Neuronal Cell-Based AD Model Has Been Constructed but Could Be Significantly Improved upon

Transgenic animal models of AD are essential for the advancement of the field. However, many aspects of AD can be studied and therapeutic strategies for the disease evaluated in the neuronal cell-based models. Conceivably, such a model could be the most authentic because it can utilize human neuronal cells. The advantage of employing human neuronal cells is not only in that they are from the species affected by the disease and therefore reflect more faithfully cellular AD pathology but also because they possess a mechanism capable of the AβPP-independent production of C99 (presumably the RNA-dependent AβPP mRNA amplification pathway but in unlikely case other mechanisms, discussed in Section 22 below, are involved, they are also in place). In light of considerations discussed in the preceding sections, to construct a human neuronal cell-based AD model is straightforward: Elicit sustainably the integrated stress response and the endogenous AβPP-independent C99 production pathway (presumably the RNA-dependent amplification of endogenously produced AβPP mRNA) would be activated, and cellular AD pathology would ensue. Approaches to elicit the ISR in human neuronal cells are described in detail in [7]. Briefly, the ISR could be elicited either by exogenously expressed iAβ or by any other suitable ISR-eliciting stressor. Utilization of the former would yield a cellular model of conventional AD whereas employment of the latter would result in a cellular model representing unconventional AD (for detailed discussion of unconventional AD see Section 50 below). The question is whether such a human neuronal cell-based model is feasible. The answer is affirmative: not only feasible but highly plausible. The basis for this assertion is a 2014 study by Choi and co-workers [128].

The proof of concept provided in [128] is rather inadvertent. Choi and co-workers designed their model with the aim to maximize the extent of extracellular deposition of exogenously produced Aβ in hope that this would, in accordance with the ACH, maximize the extent of cellular AD pathology. To achieve their goal, they expressed exogenously AβPP containing two FAD mutations as well as an FAD-causing mutant of PSEN1 [128]. The essential innovation of this study was that human neuronal cells were cultured in a semi-solid matrigel medium in order to prevent the diffusion of secreted Aβ. This experiment resulted in an unmitigated success, actually a triumph: for the first time an experimental model system exhibited the formation of neurofibrillary tangles. Choi and co-workers interpreted this outcome as the affirmation of the principles of the ACH (see the introductory sentence of Section 3 above) [128].

In the framework of the ACH2.0, however, the results of Choi and co-workers are interpreted very differently. Due to utilization of semi-solid matrigel medium, secreted Aβ did not diffuse but was retained in the proximity of cultured neurons; this facilitated their cellular uptake. Two mutations utilized in [128], London mutation of AβPP (V717I) and ΔE9 mutation of PSEN1 substantially increase the production and, subsequently, secretion of the Aβ42 isoform. As discussed in Section 24 below, Aβ42 is taken up by neurons twice as effectively as other isoforms of Aβ. Second AβPP mutation employed in [128] is Swedish mutation (K670N/M671L). This mutation was shown to substantially increase a fraction of C99 undergoing gamma-cleavage on the intracellular rather than on plasma membranes, a process culminating in the retention (rather than secretion) of resulting iAβ. The most important attribute of this model in the ACH2.0 perspective is that it maximizes the influx of iAβ derived from massively overproduced exogenous AβPP. When exogenous iAβ accumulates to sufficient levels (see Section 25 below), it triggers the elicitation of the ISR. This, in turn, activates the endogenous AβPP-independent production of C99. Consequently, the progression of cellular AD pathology commences and results in formation of neurofibrillary tangles. If the objective is to assure a sufficient influx of iAβ, the approaches employed in [128] are rather cumbersome; as mentioned above, they could be significantly improved upon. Nevertheless, the study by Choi and co-workers [128], interpreted in terms of the ACH2.0, serves as proof of principle for the plausibility of human neuronal cell-based models of AD.

## 21. Validation (4): Expression of iAβ in Human Neuronal Cells Should Trigger Cellular AD Pathology Including Formation of NFTs

In the human neuronal cell-based AD model developed by Choi and co-workers [128] and discussed in the preceding section the accumulation of iAβ occurs in an indirect and rather convoluted way. Indeed, it occurs inefficiently via the cellular uptake of secreted extracellular AβPP-derived Aβ and through the retention of iAβ resulting from gamma-cleavages occurring on intraneuronal rather than on plasma membranes. The reason for this is that this model was constructed with the purpose of maximizing the accumulation of extracellular, not intraneuronal Aβ. The goal of maximizing the accumulation of iAβ could be easily accomplished by exogenously expressing in human neuronal cells not AβPP and not C99 (because C99 contains trans-membrane domain at the C-end of its Aβ segment, and Aβ produced by gamma cleavage of C99 would be secreted) but rather Aβ, more specifically Aβ42. This can be accomplished by utilizing the AUG encoding Met671 in AβPP as translation initiation codon in the expression vector. Its primary translation product would be Met-Aβ42 (see Section 23 below); the N-terminal Met would be removed by one of multiple aminopeptidases with a broad specificity, yielding Aβ42 (discussed in Section 23 below). It can be anticipated that iAβ produced in such a manner would efficiently accumulate and rapidly reach the ISR-eliciting threshold. Consequently, the neuronal integrated stress response would be elicited (see Section 25 below), the endogenous AβPP-independent C99 generation pathway would be activated, AD cellular AD pathology would commence and progress, and neurofibrillary tangles would manifest. In a control experiment, the same procedure can be carried out in mouse neuronal cells. Since in mice the AβPP-independent C99 production pathway is inoperative (discussed above), no extensive degeneration would occur (except that caused by the exogenous iAβ-elicited neuronal ISR; see Section 19) and no NFTs would form. If the outcome obtained in human neuronal cells is as anticipated, such an experimental system would constitute an adequate cellular model system for conventional AD.

## 22. Alternative Modes of Initiation of Translation from the AUG Codon Encoding Met671 of AβPP

In the authors’ viewpoint, in fact, a conviction, RNA-dependent amplification of human AβPP mRNA is by far the most plausible candidate for the role of a mechanism enacting the AβPP-independent production of C99. It has “built-in” human specificity observed in the field and is strongly supported by the empirical data. Its chimeric end RNA end product is cap-less. But cap-less mRNA species are translated under the ISR conditions. Moreover, its nucleotide modifications potentially ensure its translatability under the ISR (even a single m(6)A modification was shown to promote cap-independent translation [129]). Nevertheless, there are three other, albeit much less likely, possibilities of utilization of the AUG encoding Met671 of AβPP as a translation initiation codon that should be mentioned here for the reader’s consideration. One such mode is the internal initiation of translation of intact AβPP mRNA from the AUG codon under discussion. This possibility was discussed in Section 7 above. It was proposed by Breimer and Denny [59] and evaluated and “ruled out” by two research groups [60,61]. However, this “ruling out”, in both studies [60,61], is grossly unfounded. Breimer and Denny posited that the initiation of translation of the intact human AβPP mRNA from the AUG encoding Met671 might occur as an inducible process in neuronal cells under the AD conditions [59]. Both studies, however, utilized non-neuronal cells and were carried out certainly not under the AD conditions [60,61]. Their conclusions, therefore, should be discounted and the initiation of translation of the intact human AβPP mRNA from the AUG encoding Met671 should be considered a viable option until proven otherwise.

The remaining two possibilities of the initiation of translation of human AβPP mRNA from the AUG encoding Met671 are conceptually similar to the outcome of the RNA-dependent AβPP mRNA amplification pathway in that the translated mRNA molecules are 5′-truncated to the extent that the AUG encoding Met671 of AβPP becomes the first functional translation initiation codon. What differ are mechanisms that produce 5′-truncated AβPP mRNA. In one case, this is the internal initiation of transcription of AβPP RNA from a position within the AβPP gene proximal to but upstream of the ATG encoding Met671. This would require expression of specialized transcription factor(s) presumably induced by the neuronal ISR conditions.

In another case, the truncation is enacted by a cleavage of AβPP mRNA in proximity of but upstream from the AUG encoding Met671. Such a process would require a specialized nuclease produced under the neuronal ISR conditions. Whereas the internal initiation of transcription would produce capped 5′-truncated AβPP mRNA (unlikely to be translated under the ISR conditions), cleavage-generated 5′-truncated AβPP mRNA would be uncapped. It should be emphasized that we consider all three possibilities interesting but implausible; they do neither reflect human specificity, nor are they supported by the empirical data.

## 23. Validation (5): The Primary Product of Translation Initiated from the AUG Encoding Met671 of Human AβPP Is Distinguishable from Its Counterpart Generated by AβPP Proteolysis and Can Serve as a Reporter

When Breimer and Denny proposed that C99 could be generated independently of AβPP via the internal initiation of translation of AβPP mRNA at the AUG encoding Met671 [59], they presumed that it would be indistinguishable from C99 produced by the proteolytic cleavage of AβPP at the beta-site, between residues 671 and 672. The reason for this is that, at that time (1987), it was assumed that the translation-initiating methionine is always removed co-translationally by N-terminal methionine aminopeptidase (MAP). If this were the case, the primary product of translation initiated at the AUG encoding Met671 would terminate at its N-end with Asp672, and would, indeed, be indistinguishable from C99 produced by the cleavage of AβPP at the Met671/Asp672 junction. Subsequent research, however, demonstrated that this is not always the case. Interestingly, the erroneous notion that N-terminal Met is always removed co-translationally by MAP still persists in a significant fraction of the scientific community despite evidence to the contrary described below; authors are indebted to Alexander Varshavsky (Caltech) for conveying this instrumental information.

For the N-terminal methionine to be cleaved-off co-translationally, it should be accommodated, together with the following amino acid residue, within the active site of MAP. This is not always possible. More precisely, this is possible only in defined combinations, namely methionine followed by any one of the seven smallest amino acid residues. In order of increasing size, they are as follows: Gly, Ser, Cys, Thr, Pro, and Val. In cases where translation-initiating methionine is followed by any amino acid residue larger than valine (in fact, by any residue other than the above listed seven), N-terminal Met would not be removed by MAP co-translationally but would be retained in the primary translation product [130,131,132,133,134,135]. Met671 of human AβPP is followed by aspartate, which is larger than valine. Therefore, if translation were to initiate at the AUG encoding Met671 of AβPP (regardless of a mechanism enabling such an initiation of translation), the primary translation product would be not C99 but rather C100, i.e., N-terminal Met-C99. C100 would not, however, retain its N-terminal methionine for long. It was shown that in cases like this, when translation-initiating methionine is retained in the primary translation product, it is eventually removed by one of the numerous aminopeptidases with broad specificity [134,135]. Importantly, however, this would occur post-translationally; only MAP is capable of the co-translational cleavage of the N-terminal methionine. It follows that if AβPP-independent production of C100 indeed occurs in AD-affected neurons and is followed by its conversion to C99, a pool of C100 would, nevertheless, persist. This is of considerable practical significance since the occurrence of C100 would report the activity of the AβPP-independent pathway of its production. A question might be asked: why was C100 not seen in postmortem samples from AD patients? The answer is because in dead tissues protein synthesis ceases well before the activity of proteolytic enzymes stops. Consequently, without a new influx of C100, its pool would be, in its entirety, converted into C99 and thus lose its identifying N-terminal Met. On the other hand, C100 pools would exist and should be detectable in adequate models of AD, described in Section 19 and Section 20 above and elsewhere [7].

## 24. Accumulation of Intraneuronal Aβ (iAβ) Occurs Physiologically via Two Distinct Processes

The present section picks-up the narrative from Section 5 (the intervening Sections were introduced to establish the plausibility of the AβPP-independent production of C99 in AD-affected neurons). In that section, we formulated the principles of the ACH2.0. The first of these principles is that conventional AD is triggered by iAβ accumulated over a certain critical threshold. Intraneuronal accumulation of Aβ is well established; it occurs (albeit, as argued below, at different rates) in healthy individuals, i.e., those who will not develop AD, as well as in future AD patients. Intraneuronal accumulation of Aβ takes place via two well-understood physiological mechanisms. One such mechanism is the retention of newly produced Aβ within the neuron. The immediate precursor of Aβ in the AβPP proteolytic pathway is the C99 fragment. Aβ is generated by the gamma-cleavage of C99. Usually this occurs on plasma membranes in conjunction with the secretion of newly formed Aβ. In some cases, however, the cleavage occurs on intraneuronal membranes within various organelles [136,137,138,139,140,141,142,143,144]. Aβ generated in this way is not secreted to the extracellular space but is retained intraneuronally as iAβ. As discussed below, certain mutations of AβPP and of presenilins facilitate the cleavage of C99 on the intraneuronal membranes; this increases the fraction of AβPP-derived Aβ retained as iAβ and elevates the rate of its influx.

Another physiologically operating mechanism resulting in accumulation of AβPP-derived iAβ is the cellular uptake of secreted Aβ [145,146,147,148,149,150]. Apparently, the oligomerization of extracellular Aβ is a prerequisite for its effective internalization [149,150]. For this reason, due to its demonstrated propensity to aggregate, the Aβ42 variant is taken up by cells at the rate twice that of other Aβ isoforms [150]. The cellular uptake of extracellular Aβ is mediated by numerous receptors [151,152,153,154,155,156,157,158,159]. The ligands of these receptors play an important role in and regulate the internalization of extracellular Aβ. One of such ligands is ApoE [149]. Of its several variants, ApoE4 is substantially more effective in mediating the internalization of extracellular Aβ than other ApoE variants; its occurrence, therefore, increases the rate of cellular uptake of extracellular Aβ and thus elevates the rate of influx of AβPP-derived iAβ.

## 25. iAβ Accumulated over the Critical Level Activates PKR and HRI Kinases and Thus Triggers the Elicitation of the Neuronal ISR

The elicitation of the integrated stress response is enacted, via phosphorylation of eIF2α, by one or more of eIF2α kinases. iAβ was shown to be capable of mediating the activation of two of those: PKR and HRI. The activation of PKR was observed, alongside with phosphorylation of eIF2α, in cell models and animal models that overexpress Aβ [160,161,162]. Moreover, both activated PKR and eIF2α phosphorylated at its Ser51 residue were detected in neuronal cells of AD patients [163,164]. iAβ, at sufficient cellular levels, can apparently cause phosphorylation and consequent activation of PKR in two ways. One is through PKR-activator (PACT). This conclusion is supported by the observation that PACT and activated PKR are co-localized in AD-affected neurons [165]. Another way for iAβ to cause phosphorylation and activation of PKR is via tumor necrosis factor alpha (TNFα), as was observed in transgenic mouse models that overexpress human Aβ [166].

The activation of HRI in Alzheimer’s disease, on the other hand, is a corollary of the iAβ-triggered mitochondrial dysfunction in AD-affected neurons. AD-related mitochondrial distress is one of the earliest symptoms of the disease and as such was studied extensively [167,168,169,170,171,172,173,174,175,176,177,178,179,180,181,182,183,184]. The connection between iAβ-caused mitochondrial dysfunction and the activation of HRI in AD-affected neurons is as follows. One of the early events in dysfunctional mitochondria is the activation of mitochondrial protease OMA1 [185,186]. Upon its activation, OMA1 cleaves another mitochondrial protein, DELE1, in a site-specific manner [185,186]. One of the resulting fragments of DELE1 is transported across mitochondrial membranes into the cytosol [185,186]. This fragment has an affinity to HRI; upon binding, it activates the kinase. Thus, iAβ, accumulated to sufficient levels, triggers the activation of either PKR (via PACT and/or TNFα) or HRI (through OMA1 to DELE1 to HRI pathway). When PKR or HRI, or both, are activated, eIF2α is phosphorylated at its Ser51 residue and the elicitation of the neuronal ISR ensues.

## 26. The Neuronal ISR Enables Operation of the Self-Sustainable AβPP-Independent C99 Production Pathway; AD Commences Only When the Latter Is Activated

The rate of accumulation of AβPP-derived iAβ via its intraneuronal retention or through the cellular uptake of secreted extracellular Aβ, both occurring physiologically, could be (albeit not always; see below) sufficient to activate PKR and/or HRI and thus trigger phosphorylation of eIF2α and elicitation of the neuronal integrated stress response but it is patently insufficient to support the accretion of AβPP-derived iAβ to the AD pathology-causing levels. The AD pathology-causing levels of iAβ are attained via the activity of the AβPP-independent C99 generation pathway (discussed in the preceding sections). As described above, under the conditions of the neuronal ISR, translation of the global cellular cap-dependent mRNAs to proteins is severely suppressed. Concurrently, an array of new transcription factors is expressed, new mRNA species are transcribed, and cap-independent production of a small subset of cellular proteins is activated. Among those are essential components of the AβPP-independent C99 production pathway that are not present under the non-ISR conditions; when they are available, the pathway is activated. In the version of ACH2.0 under discussion C99 generated independently of AβPP is processed into iAβ. Its entire output is retained within neurons. Consequently, its levels rapidly increase and reach AD pathology-causing range. Not only is it toxic in its own right, it was also shown to suppress the ubiquitin-proteasome system and thus promote tau accumulation [187,188,189,190].

**Figure 6:**
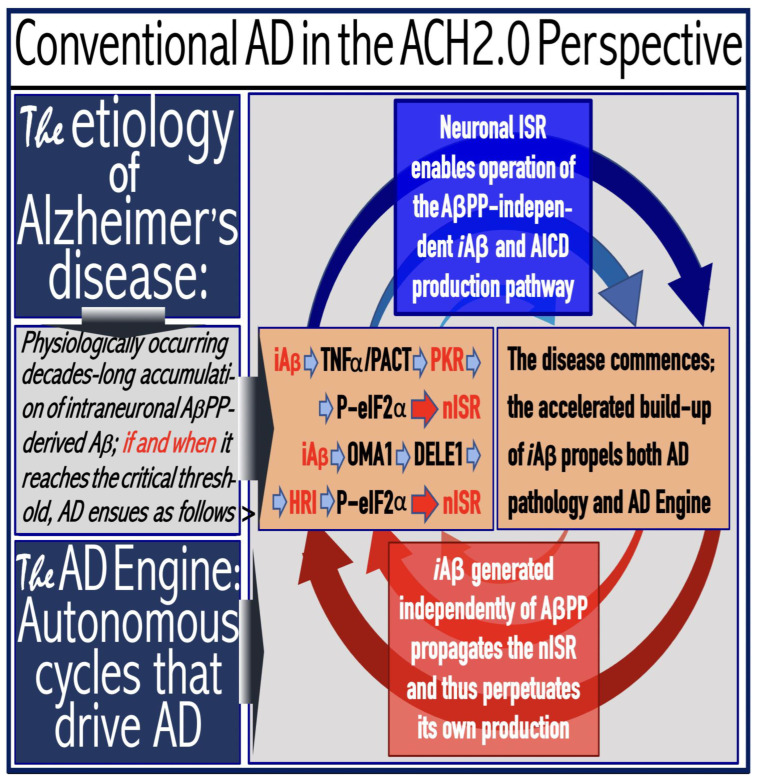
**AD is driven by the self-sustainable AβPP-independent C99 production pathway enabled by the neuronal ISR**. *eIF2α*: eukaryotic translation initiation factor 2*α*. *PKR and HRI*: kinases that phosphorylate eIF2α at its Ser51 residue. *PACT*: PKR activator. *TNFα*: tumor necrosis factor *α*. *OMA1*: mitochondrial protease activated during mitochondrial dysfunction. *DELE1*: substrate of OMA1 protease. Cleavage of DELE1 leads to the activation of HRI. Phosphorylation of eIF2α elicits the integrated stress response, which, in turn, enables the generation of components essential for the activity of the AβPP-independent pathway of C99 and, subsequently, iAβ production. iAβ: intraneuronal Aβ. AβPP-derived iAβ accumulates physiologically via the importation of extracellular Aβ and the retention of iAβ produced by gamma-cleavages on intracellular membranes in an exceedingly slow process. In most individuals it does not reach the PKR- and/or HRI-activating, the neuronal ISR-eliciting threshold within their lifetimes and no conventional AD occurs. When this threshold is crossed, the neuronal ISR is elicited and the AβPP-independent C99 (and, subsequently, iAβ) production pathway activated. The iAβ product of the latter drives AD pathology. It also propagates the neuronal ISR and thus perpetuates its own production in the AβPP-independent pathway. It should be noted that the accumulation of iAβ produced in both the AβPP proteolytic and AβPP-independent pathways is accompanied by a proportional accumulation of AICD (AβPP Intracellular Domain). AICD was shown to be capable of interfering with numerous processes involved in AD [191,192,193,194,195,196,197,198,199,200,201,202,203,204,205,206] but its contribution to the disease, if any, remains to be elucidated.

Thus, in this version of the ACH2.0, AD is triggered, via the elicitation of the neuronal integrated stress response, by AβPP-derived iAβ accumulated to sufficient levels and is driven by differentially derived iAβ generated by gamma-cleavage of C99 produced in the AβPP-independent pathway. Since the accumulation of AβPP-derived iAβ, regardless of its rate, is a physiological process, AD commences only with the elicitation of the neuronal ISR and consequent activation of the AβPP-independent C99 production pathway. Importantly, driving AD pathology is only one function of iAβ produced independently of AβPP. Its other major function is ascertaining the sustainability of its own production. Indeed, when the disease commences, levels of iAβ (derived from AβPP) are, by definition, already above those required for the elicitation of the neuronal ISR. Operation of the AβPP-independent C99 production pathway supplies, at the rate orders of magnitude greater than the rate of influx of AβPP-derived iAβ, additional, differentially derived iAβ. This maintains PKR and/or HRI in the activated states, sustains phosphorylation of eIF2α, and thus propagates the neuronal ISR and, consequently, perpetuates the operation of the AβPP-independent C99 (and, in this ACH2.0 version, iAβ) production pathway. This feedback loop ensures the uninterrupted production of C99 and iAβ independently of AβPP and constitutes the Engine that drives the disease. The sequence of events described above is illustrated in Figure 6.

## 27. The Decisive Role of the Rate of Accumulation of AβPP-Derived iAβ in the Occurrence and Timing of Alzheimer’s Disease

**Figure 7:**
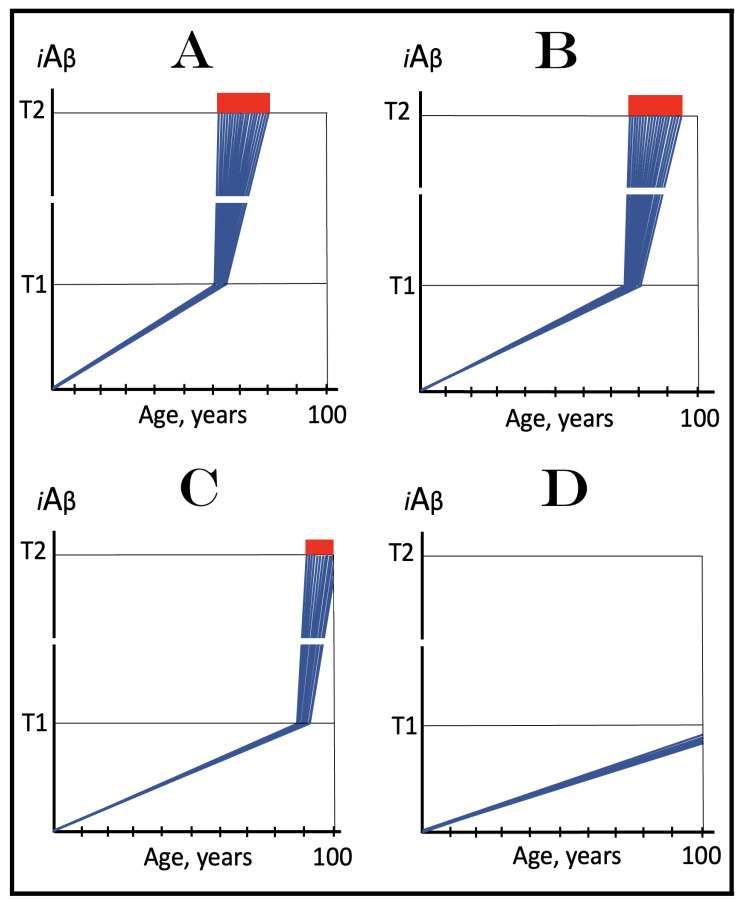
**The occurrence and timing of conventional AD is determined by the dynamics of accumulation of AβPP-derived iAβ**. *Blue lines*: Intraneuronal Aβ, iAβ. ***T1***: Threshold of cellular concentration of iAβ triggering the activation of PKR and/or HRI kinases, phosphorylation of eIF2α, the elicitation of the neuronal ISR, the initiation of the AβPP-independent C99 production pathway, and the commencement of AD. ***T2***: Cellular concentration of iAβ triggering neuronal death via apoptosis or necroptosis [207]. *Red box*: Apoptotic zone, defined as a range of cellular concentrations of iAβ within which cells neuronal cells committed apoptosis or necroptosis or are dead. The only variable parameter in the present figure is the rate of accumulation of AβPP-derived iAβ. *Panel* (**A**): The rate of accumulation of AβPP-derived iAβ is relatively rapid. It crosses the T1 threshold at mid-sixties. PKR and/or HRI are activated, eIF2α phosphorylated, the neuronal ISR elicited, AβPP-independent C99 generation pathway initiated, and AD commences. *Panel* (**B**): The rate of accumulation of AβPP-derived iAβ is reduced. The T1 threshold is reached and crossed, and the disease commences at around the age of eighty. *Panel* (**C**): The rate of accumulation of AβPP-derived iAβ is reduced further. The T1 threshold is reached, and the disease commences only in the nineties. *Panel* (**D**): AβPP-derived iAβ does not reach the T1 threshold within the lifespan of the individual; the neuronal ISR is not elicited, the AβPP-independent C99 generation pathway is not activated, and conventional AD does not occur. Such an outcome is typical for the majority of the human population.

In Figure 7 below, the T1 threshold defines levels of AβPP-derived iAβ that mediate the activation of PKR and/or HRI and thus trigger elicitation of the neuronal integrated stress response, consequent activation of the AβPP-independent C99 production pathway, and commencement of AD. The figure makes it apparent that the occurrence of AD crucially depends on the rate of accrual of AβPP-derived iAβ (the only variable in Figure 7). It is a simple logic. If no T1 crossing occurs within the lifetime of an individual, there is no AD. This, actually, describes the majority of the human population: most individuals do not develop the disease. If, however, the T1 threshold is crossed, the timing of the crossing, and, consequently, of the commencement of AD is reversely proportional to the rate of accrual of AβPP-derived iAβ: greater the latter, shorter the former. Four panels of Figure 7 depict four possibilities. First three (Panels A–C) vary only quantitatively and illustrate the proportionality invoked above. The fourth possibility (Panel D) differs qualitatively from others: the T1 threshold is not crossed, and AD does not occur. The T1 threshold appears to constitute a proverbial thin line that separates health and disease.

The concept of connectivity between the rate of accumulation of AβPP-derived iAβ and AD is validated by the analysis of mechanisms of action of all known FAD mutations, both causative and protective, which is presented in Section 32, Section 33 and Section 34 below. This connectivity, moreover, includes not only FAD mutations, but also all factors predisposing to the disease. A good example is ApoE4. As was mentioned above, all members of the ApoE family of ligands facilitate the cellular uptake of secreted extracellular Aβ; their efficiency, therefore, influence the rate of influx of AβPP-derived iAβ and, consequently, that of its accumulation. ApoE4 is substantially more efficient in facilitating the internalization of extracellular Aβ than other ApoE variants [149]. In carriers of ApoE4 allele(s), therefore, the rate of accumulation of AβPP-derived iAβ is greater than in the general population. Therefore, in these individuals the T1 threshold is crossed and, consequently, AD occurs sooner (or more often) than in the general population. They are “predisposed” to the disease and this predisposition is dose-dependent: carriers with only one ApoE4 allele are predisposed to the disease less than those carrying two ApoE4 alleles.

## 28. On the Conditionality of the First Stage of Conventional AD

As depicted in Figure 7 above, the kinetics of accumulation of iAβ in individuals who do not develop AD within their lifetimes is single-phased. It is, however, double-phased in those who eventually become AD patients. Phase One is the accrual of AβPP-derived iAβ until it crosses the T1 threshold. At this point the neuronal ISR is elicited, the AβPP-independent C99 (and, in this version of ACH2.0, iAβ) production pathway is activated, and the second (rapid) phase of iAβ accumulation and the disease (driven by iAβ produced in second phase) commence. Phase One of iAβ accumulation is, therefore, essential for phase Two and the disease to occur.

It is reasonable, even natural, therefore, to associate phases of iAβ accumulation with stages of AD. In these terms, phase One of iAβ accumulation is equated with stage one of AD. But this stage is a physiological process and no disease occurs at this stage. Thus, as an example, according to the above logic we must append the definition “stage one of AD” to AβPP-derived iAβ accumulation depicted in Panel D of Figure 7, which is an oxymoron: the disease not only does not occur but is not going to occur. A solution to this conundrum is simple: stage one of AD is conditional, it is rendered such only post-factum, if and when the T1 threshold was crossed and the disease actually commenced; short of this, there is no “stage one” of AD, only normal, physiologically occurring process of accumulation of AβPP-derived iAβ.

## 29. On the Inevitability of Conventional AD in a Sufficiently Long Lifespan

In terms of the preceding section, the incidence of AD depends on the conversion of accumulation of AβPP-derived iAβ from a physiologically occurring process into the fist stage of the disease, and this conversion is demarcated by the crossing of the T1 crossing. If the T1 threshold were not crossed within a defined lifespan, no AD would occur. But the defined lifespan is a subjective, ever changing, more precisely, ever increasing variable. In Figure 7 it is assumed to be 100 years. In Panel D, the T1 threshold is not crossed at this time limit and the disease does not occur, but in Panels A–C both the T1 crossing takes place and AD develops. As was mentioned above, at the time of Dr. Alois Alzheimer, life expectancy in Germany was only 48 years. At this “defined” lifespan the crossing of the T1 threshold would take place and AD would occur in neither of panels of Figure 7. Inversely, we can extrapolate what would happen with the increase in the “defined” lifespan. The conclusion is straightforward: provided the lifespan is long enough, the T1 threshold would be crossed, and the disease would inevitably occur. Moreover, the “long enough” is not far away. Currently, the incidence of the disease is 5% at sixty-five, 15% at seventy-five, and 35% at eighty-five. By extrapolation, it would approach 100% at about hundred-twenty. In the last hundred years, the lifetime expectancy in industrial countries has increased by 60% (from about fifty to about eighty). Given a comparable rate of increase, the longevity of 120 years and the incidence of AD approaching certainty would be reached within the next five decades. The disease is inevitable, provided the lifespan is sufficiently long, because the first stage of AD, or rather potential first stage of AD, is a physiologically occurring process; given enough time, the potential would be realized and the T1 threshold would, unavoidably, be reached. There are only two approaches to preclude the eventual certitude of developing AD. One is to limit the lifespan. Another is to develop therapies for the prevention and cure of the disease. The second approach is vastly preferable and is discussed below.

## 30. The Critical Role of the Extent of the T1 Threshold in the Incidence and Timing of AD and of Aging-Associated Cognitive Decline (AACD)

**Figure 8:**
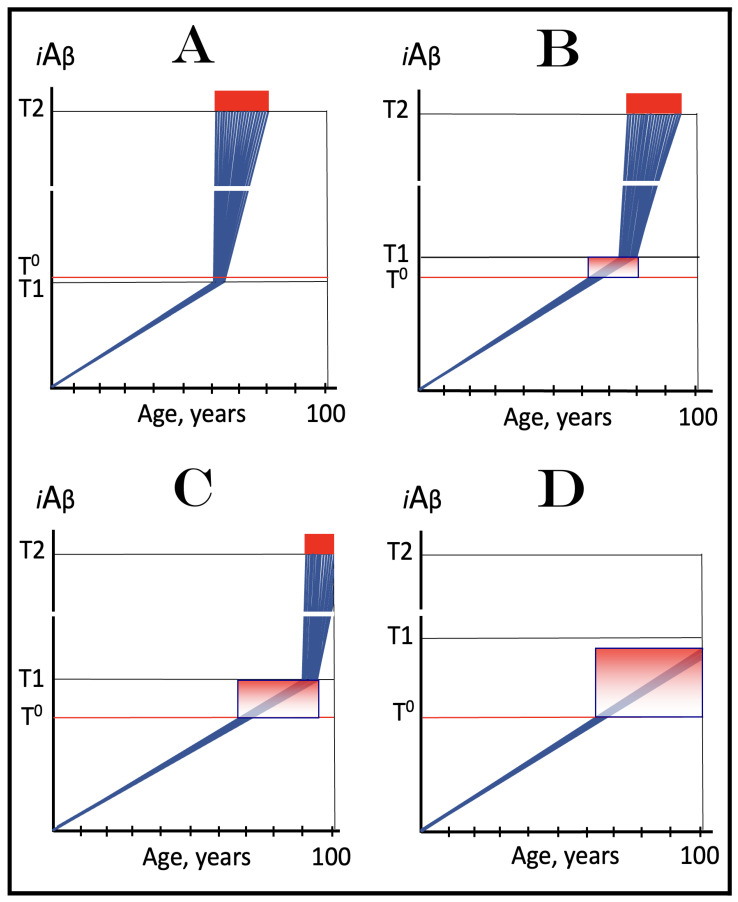
**Role of the extent of the T1 threshold in the incidence and timing of AD and of aging-associated cognitive decline, AACD**. *Blue lines*: Intraneuronal Aβ, iAβ. ***T*^0^**: Threshold of cellular concentration of AβPP-derived iAβ triggering the neuronal damage, which manifests as AACD. ***T1***: Threshold of cellular concentration of iAβ triggering the activation of PKR and/or HRI kinases, phosphorylation of eIF2α, the elicitation of the neuronal ISR, the initiation of the AβPP-independent C99 production pathway, and the commencement of AD; its extent is the only variable in the present figure. ***T2***: Cellular concentration of iAβ triggering neuronal death via apoptosis or necroptosis. *Red box*: Apoptotic zone, defined as a range of cellular concentrations of iAβ within which cells neuronal cells committed apoptosis or necroptosis or are dead. *Pink boxes*: The range of cellular concentrations of AβPP-derived iAβ concentrations between the T^0^ and the highest extent reached by AβPP-derived iAβ or the T1 threshold that support the progression of AACD. The condition commences with the crossing of the T^0^ threshold and morphs into AD with the T1 crossing (if no T1 occurs, AACD persists for the remaining portion of the lifetime). It follows that AACD can occur only if the extent of the T^0^ threshold is smaller than that of the T1. *Panel* (**A**): The T1 threshold is crossed, the neuronal ISR is elicited, the AβPP-independent C99 and iAβ production pathway is activated, and AD commences at about sixty years of age. The extent of the T^0^ threshold is greater than that of the T1; consequently, no AACD occurs. *Panel* (**B**): The extent of the T1 threshold is above that of the T^0^ threshold. When the T^0^ is crossed by AβPP-derived iAβ, AACD commences and morphs into AD following the T1 crossing at about seventy-five years of age. *Panel* (**C**): The T1 threshold is increased further and is crossed at about ninety years of age. Since the extent of the T^0^ does not change, AACD commences at the same time as the preceding panel, but its duration increases. *Panel* (**D**): The extent of the T1 threshold is such that it is not crossed by AβPP-derived iAβ within the lifetime of the individual; no conventional AD occurs. On the other hand, AACD commences with the T^0^ crossing and persists for the remaining lifetime.

As was discussed above (Section 26), the incidence and the timing of conventional AD are defined by the instance when AβPP-derived iAβ crosses the neuronal ISR-eliciting T1 threshold. This crossing, in turn, depends on two factors: the rate of accumulation of AβPP-derived iAβ and the extent of the T1 threshold. The former was discussed in the preceding sections; the latter is addressed in the present section. The extent of the T1 threshold is of special importance. As discussed in the following section below, the extent of the T1 threshold in the subpopulation predisposed to non-familial conventional AD is lower than in the general population. Moreover, this parameter is decisive in the occurrence of aging-associated cognitive decline, AACD. The role of the extent of the T1 threshold in the occurrence and timing of AD is illustrated in Figure 8 (this parameter is the only variable in Figure 8). The logic of this figure is apparent: with the given rate of accumulation of AβPP-derived iAβ, the greater is the extent of the T1 threshold, the later will it be crossed and the later will AD occur (Panels A–C of Figure 8). Eventually, within a defined lifespan, the extent of the T1 threshold could be such that at the given rate of accumulation of AβPP-derived iAβ neither the T1 will be crossed, nor would AD occur (Panel D of Figure 8).

iAβ is prone to aggregate and it is reasonable to assume that above a certain level (different for different Aβ variants) its aggregates are bound to cause a degree of neuronal damage. The threshold demarcating the commencement of such neuronal damage is designated T^0^ in Figure 8. If the extent of the T^0^ threshold were greater than that of its T1 counterpart, this neuronal damage would be a component of AD (Panel A of Figure 8). If, however, the extent of the T^0^ threshold were smaller than that of the T1, the damage would occur but would be insufficient to trigger the elicitation of the neuronal integrated stress response. In terms of the ACH2.0, AACD is defined as the neuronal damage occurring in the range of concentrations of AβPP-derived iAβ between the T^0^ and T1 thresholds. It follows that for AACD to occur, the T^0^ threshold must be lower than the T1. Thus, no AACD occurs in Panel A of Figure 8. In Panel B through D, the crossing of the T^0^ threshold triggers AACD. At the given extent of the T^0^ threshold, its duration increases with the increase in the extent of the T1 threshold. In Panels B and C of Figure 8 the T1 threshold is eventually crossed and AACD morphs into AD. In Panel D of Figure 8, due to the limited lifespan, the T1 threshold is not crossed. In this scenario, no neuronal ISR is elicited, no AβPP-independent C99 production pathway is activated, no AD occurs. AACD, however, commences with the crossing of the T^0^ threshold and progresses until the end of the lifespan.

## 31. “Sporadic AD” Is Not Sporadic at All: The Disease Affects a Well-Defined, Non-Random Subpopulation; “Non-Familial AD” Is a Much Better Designation

“Sporadic” in “sporadic AD” has, uncomfortably, fatalistic connotations. It implies that, starting from a typical age of the onset of the disease, about sixty-five, the disease can strike anyone at random. This, however, is patently not the case. It follows from the preceding sections that individuals with high rate of accumulation of AβPP-derived iAβ and with low extent of the neuronal ISR-eliciting T1 threshold are selectively prone, “predisposed”, to the disease. This is consistent with the findings, discussed above, that carriers of the ApoE4 allele are more likely to develop the disease, and, moreover, carriers of two ApoE4 alleles are much more likely to develop AD than carriers of other ApoE alleles. This is because ApoE4 is significantly (and in a dose-dependent manner, i.e., two versus one alleles) more efficient in facilitation of the cellular uptake of extracellular Aβ than other ApoE variants. The utilization of ApoE4 increases the influx of AβPP-derived iAβ and thus elevates both its rate of accumulation and the degree of predisposition to the disease. ApoE4 is only one example. Doubtless, numerous other factors related to the production, importation, and clearing of both extra- and intraneuronal Aβ influence the rate of AβPP-derived accumulation and, consequently, the degree of predisposition to AD.

As for the extent of the T1 threshold, it is apparently lower in the subpopulation that develops non-familial conventional AD than in the general population. This conclusion is arrived at as follows. The age of onset of AACD is greater than that of non-familial (“sporadic”) AD [208,209]. It follows that in the AACD-developing subpopulation, which is significantly larger than the non-familial AD-developing subpopulation, the extent of the T^0^ and, of course, T1 thresholds are greater than that of the T1 threshold in the subpopulation predisposed to non-familiar AD. The correlation of the low extent of the T1 threshold and the high rate of incidence of non-familial AD is consistent with the conclusion reached in the preceding sections that the former predisposes to the latter. Thus, the subpopulation that is likely to develop non-familial AD can be defined as a category of individuals with the rate of accumulation of AβPP-derived iAβ above the average and with the extent of the T1 threshold below the average seen in the general population. It can be stated with certainty that any factor that contributes to the increase in the rate of accumulation of AβPP-derived iAβ and/or to the lowering of the T1 threshold predisposes to AD.

## 32. Validation (6): ALL FAD Mutations Exert Their Effect by Accelerating the Rate of Accumulation of AβPP-Derived iAβ or Lowering the Extent of the T1 Threshold—Type One FAD Mutations

To validate the concept that conventional AD is triggered when AβPP-derived iAβ crosses the T1 threshold and that, consequently, the rate of its accumulation and the extent of the T1 threshold define the incidence and timing of the disease one does not have to venture far. The analysis of mechanisms of action of the FAD-causing mutation as well as of the mutation that protects from AD and AACD makes a powerful case. Strikingly, all such mutations, both of AβPP and of presenilins, without a single exception, exert their effect either by accelerating or decelerating the rate of accumulation of AβPP-derived iAβ or by lowering the extent of the T1 threshold. The present and the following sections analyze mutations that cause early-onset AD (FAD). They are numerous and, on the basis of their mechanisms of action, can be divided into two types. The present section considers Type One of FAD mutations. This type comprises mutations that increase the influx of AβPP-derived iAβ and thus elevate its rate of accumulation and accelerate the crossing of the T1 threshold and, consequently, the commencement of AD. It is exemplified by the Flemish and Swedish AβPP mutations, as well as by certain presenilin mutations. Flemish A692G mutation affects the physiological activity of BACE2 [210]. The main function of this enzyme is regulation of the production of Aβ and of the levels of iAβ. Its major activity is the cleavage within the iAβ or within the Aβ segment of AβPP or C99. Cleavage within iAβ directly reduces its levels whereas cleavage within the Aβ segment of AβPP or C99 does it indirectly by reducing the production and, consequently, secretion and uptake of Aβ. The Flemish mutation suppresses this activity of BACE2. As an effect, less iAβ is eliminated, more Aβ is secreted, and more extracellular Aβ is imported as iAβ. This increases the influx of AβPP-derived iAβ, elevates its rate of accumulation and accelerates the T1 crossing and commencement of the disease; hence, early-onset AD.

**Figure 9:**
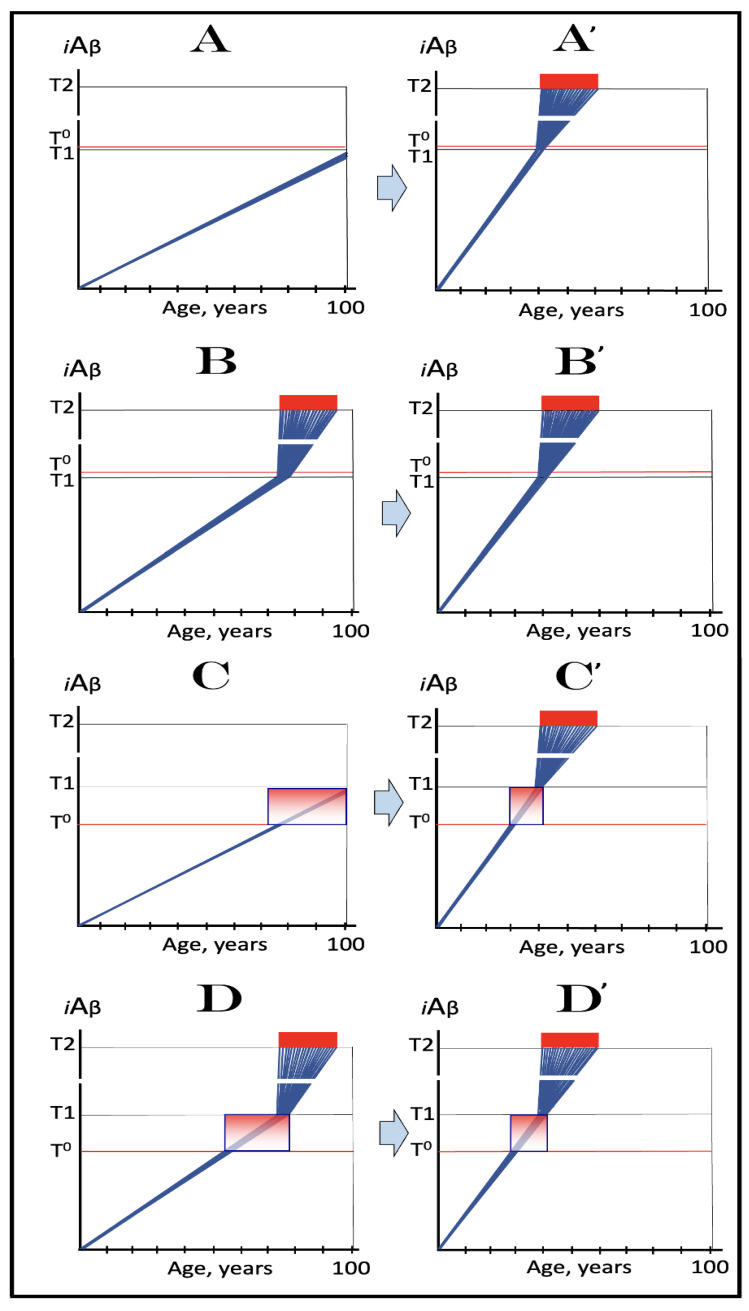
**Familial AD mutations that exert their effect by accelerating the rate of accumulation of AβPP-derived iAβ**. *Blue lines*: Intraneuronal Aβ, iAβ. ***T*^0^**: Threshold of cellular concentration of AβPP-derived iAβ triggering the neuronal damage, which manifests as AACD. ***T1***: Threshold of cellular concentration of iAβ triggering the activation of PKR and/or HRI kinases, phosphorylation of eIF2α, the elicitation of the neuronal ISR, the initiation of the AβPP-independent C99 production pathway, and the commencement of AD; its extent is the only variable in the present figure. ***T2***: Cellular concentration of iAβ triggering neuronal death via apoptosis or necroptosis. *Red box*: Apoptotic zone, defined as a range of cellular concentrations of iAβ within which cells neuronal cells committed apoptosis or necroptosis or are dead. *Pink boxes*: The range of cellular concentrations of AβPP-derived iAβ concentrations between the T^0^ and the highest extent reached by AβPP-derived iAβ or the T1 threshold that support the progression of AACD. The condition commences with the crossing of the T^0^ threshold and morphs into AD with the T1 crossing; it can occur only if the extent of the T^0^ threshold is smaller than that of the T1. *Panels* (**A**–**D**): dynamics of iAβ accumulation in wild-type AβPP carriers. *Panels* (**A**,**B**): the T^0^ levels are above those of T1; no AACD occurs. Panel (**A**): the T1 threshold is not crossed; no disease occurs. *Panel* (**B**): the T1 threshold is crossed; this is followed by late onset AD. *Panels* (**C**,**D**): the T^0^ threshold is lower than T1. *Panel* (**C**): the T1 threshold is not reached; when the T^0^ threshold is crossed, AACD commences and persists for the remaining lifespan. *Panel* (**D**): AACD commences when the T^0^ is crossed and morphs into late onset AD upon the T1 crossing. *Panels* (**A’**–**D’**): Dynamics of iAβ accumulation in carriers of FAD mutations, which elevate the rate of accumulation of AβPP-derived iAβ. As a result, the T1 threshold is crossed sooner, and early-onset AD follows. *Panels* (**A’**,**B’**): the T^0^ levels are above those of T1; no AACD occurs. *Panels* (**C’**,**D’**): the extent of the T^0^ threshold is smaller than that of the T1; AACD commences with the T^0^ crossing and morphs into the early-onset AD when the T1 threshold is reached.

The Swedish AβPP mutation [211] and certain presenilin mutations [212] achieve the same outcome in a different way. They increase the fraction of C99 cleavages occurring on intracellular membranes within various cellular organelles, as described in Section 24 above. Aβ resulting from these cleavages of AβPP-derived C99 is nor secreted but is retained within neuronal cells as iAβ. As a result, the influx of AβPP-derived iAβ is increased, the rate of its accumulation is elevated, the crossing of the T1 threshold and commencement of AD are accelerated, and early-onset AD ensues. The above considerations are illustrated in Figure 9, which compares wild type (Panels A through D) with mutants (Panels A’ through D’) and considers two scenarios. In one, the extent of the T^0^ threshold is greater than that of the T1 threshold; no AACD occurs. In the other scenario, the extents of the T^0^ and T1 thresholds are reversed. In these cases, AD (if it occurs) is preceded by AACD.

## 33. Validation (7): ALL FAD Mutations Exert Their Effect by Accelerating the Rate of iAβ Accumulation or Lowering the Extent of the T1 Threshold—Type Two FAD Mutations

Type One FAD-causing mutations, described in the preceding section, increase the influx of AβPP-derived iAβ, elevate the rate of its accumulation, and accelerate the T1 crossing and commencement of conventional AD. Type Two FAD-causing mutations, both of AβPP and presenilins, do the same. In addition, they also lower the extent of the T1 threshold. Mechanistically, all Type Two FAD-causing mutations result in significantly increased production of Aβ42 [147]. The increase in the Aβ42 fraction of Aβ plays out both inside and outside the neuronal cells. When outside, extracellular Aβ42 is, as discussed above, taken up at the rate twice that of other Aβ variants. The reason for this is, apparently, the propensity of Aβ42 to aggregate. A prerequisite for effective cellular uptake of extracellular Aβ is its oligomerization, which is facilitated by the aggregate-forming potential of Aβ42. The increased rate of importation reproduces the effect of Type One of FAD mutation: the influx of AβPP-derived iAβ is increased, the rate of its accumulation elevated, and the crossing of the T1 threshold and commencement of AD accelerated. When inside the neuronal cells, iAβ42 lowers the extent of the T1 threshold. This is because the T1 is defined as the cellular concentration of iAβ that triggers activation of PKR and/or HRI and consequent elicitation of the neuronal ISR. iAβ42 is more toxic than other variants of iAβ. This is due to the same propensity to aggregate that increases the efficiency of its cellular uptake. Consequently, a cellular concentration of iAβ42 smaller than that of other iAβ variants is required to generate a certain level of cellular stress and/or toxicity to trigger the elicitation of the neuronal ISR, i.e., the T1 threshold for iAβ42 is lower than for other iAβ isoforms.

**Figure 10:**
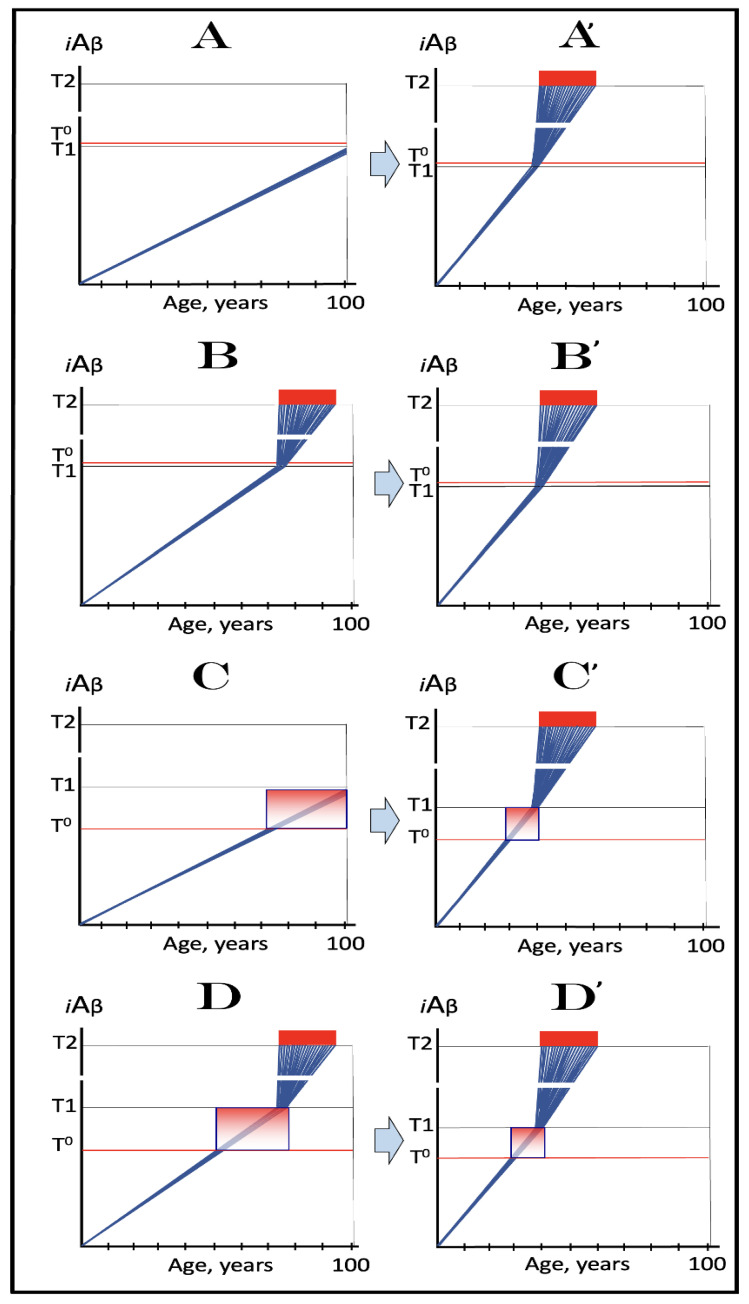
**FAD mutations that exert their effect by accelerating the rate of iAβ Accumulation and lowering the extent of T1.** *Blue lines*: Intraneuronal Aβ, iAβ. ***T*^0^**: Threshold of cellular concentration of AβPP-derived iAβ triggering the neuronal damage, which manifests as AACD. ***T1***: Threshold of cellular concentration of iAβ triggering the activation of PKR and/or HRI kinases, phosphorylation of eIF2α, the elicitation of the neuronal ISR, the initiation of the AβPP-independent C99 production pathway, and the commencement of AD; its extent is the only variable in the present figure. ***T2***: Cellular concentration of iAβ triggering neuronal death via apoptosis or necroptosis. *Red box*: Apoptotic zone, defined as a range of cellular concentrations of iAβ within which cells neuronal cells committed apoptosis or necroptosis or are dead. *Pink boxes*: The range of cellular concentrations of AβPP-derived iAβ concentrations between the T^0^ and the highest extent reached by AβPP-derived iAβ or the T1 threshold that support the progression of AACD. The condition commences with the crossing of the T^0^ threshold and morphs into AD with the T1 crossing; it can occur only if the extent of the T^0^ threshold is smaller than that of the T1. *Panels* (**A**–**D**): dynamics of iAβ accumulation in wild-type AβPP carriers. *Panels* (**A**,**B**): the T^0^ levels are above those of T1; no AACD occurs. *Panel* (**A**): the T1 threshold is not crossed; no disease occurs. Panel (**B**): the T1 threshold is crossed; this is followed by late onset AD. *Panels* (**C**,**D**): the T^0^ threshold is lower than T1. *Panel* (**C**): the T1 threshold is not reached; when the T^0^ threshold is crossed, AACD commences and persists for the remaining lifespan. Panel (D): AACD commences when the T^0^ is crossed and morphs into late onset AD upon the T1 crossing. *Panels* (**A’**–**D’**): Dynamics of iAβ accumulation in carriers of FAD mutations, which not only elevate the rate of accumulation of AβPP-derived iAβ but also reduce the extent of the T1 threshold. As a result, the T1 threshold is crossed sooner, and early-onset AD follows. Panels (**A’,B’**): the T^0^ levels are above those of T1; no AACD occurs. Panels (**C’**,**D’**): the extent of the T^0^ threshold is smaller than that of the T1; AACD commences with the T^0^ crossing and morphs into the early-onset AD when the T1 is reached.

Effects of Type Two FAD-causing mutations are depicted schematically in Figure 10. Panels A through D show wild types whereas Panels A’ through D’ depict Type Two FAD-causing mutants. The effects of mutations are driven by two factors: the increase in the rate of accumulation of the AβPP-derived iAβ and the reduction in the extent of the T1 threshold. Cumulatively, this results in a substantial acceleration of both the T1 crossing and commencement of AD. In cases where the extent of the T^0^ threshold is greater than that of the T1 threshold, no AACD occurs. When the inverse is true, AD (when it occurs) is preceded by AACD. It should be noted that due to a steeper rate of AβPP-derived iAβ accumulation the duration of AACD could be significantly shorter in mutants than in their wild-type counterparts. Also of note is that in Panels A’ through D’ the extent of the T2 thresholds would likely be also lowered due to the increased toxicity of iAβ42.

## 34. Validation (8): The Icelandic AβPP Mutation Protects from AD and AACD by Lowering the Influx of AβPP-Derived iAβ and Decelerating Its Rate of Accumulation

The preceding two sections validate the concept that the incidence and timing of conventional AD are determined by the crossing of the T1 threshold, which, in turn, is defined by the rate of accumulation of AβPP-derived iAβ and by the extent of the T1 threshold. The analysis described above is based on the elucidation of the mechanisms of action of the FAD-causing mutations. The present section provides a complementary validation of the same concept, arriving at it from the opposite direction. It does so by analyzing the mechanism of action of the only known AD-protective mutation. The Icelandic AβPP mutation A673T confers to its carriers protection from both AD and AACD [43,44]. This is a remarkable finding. It confirms that Aβ is involved in AACD to no lesser extent than in AD, a notion articulated by the ACH2.0 and discussed in Section 29 above. The mechanism underlying the protective effect of the Icelandic mutation has been elucidated. At its core is the increase in the rate of BACE1-mediated cleavage within iAβ and Aβ segments of AβPP and C99. As discussed above and as indicated by its designation (beta-site AβPP-cleaving enzyme), BACE1 cleaves primarily at the beta-site, i.e., between the residues 671 and 672 of AβPP; this is its major activity. Its minor activity is the cleavage at the β’-site situated ten amino acid residues downstream from the β-site. Cleavage at the β’-site both degrades iAβ and prevents its formation by the proteolysis of AβPP or C99. The Icelandic mutation increases the efficiency of cleavages at the β’-site. The increase is relatively small, only 30%, but its effects are highly consequential. Cleavage within iAβ degrades the peptide and increases the rate of its efflux. Cleavages within Aβ segments of AβPP and C99 reduce the production and secretion of Aβ and, consequently, the rate of its importation as iAβ. Thus, the rate of the influx of AβPP-derived iAβ is reduced and that of its efflux is increased. As a result, the rate of accumulation of AβPP-derived iAβ is decelerated, and it does not reach the T^0^ and T1 thresholds within the lifespan of the mutation carriers or reaches them much later in life than in the wild type.

**Figure 11:**
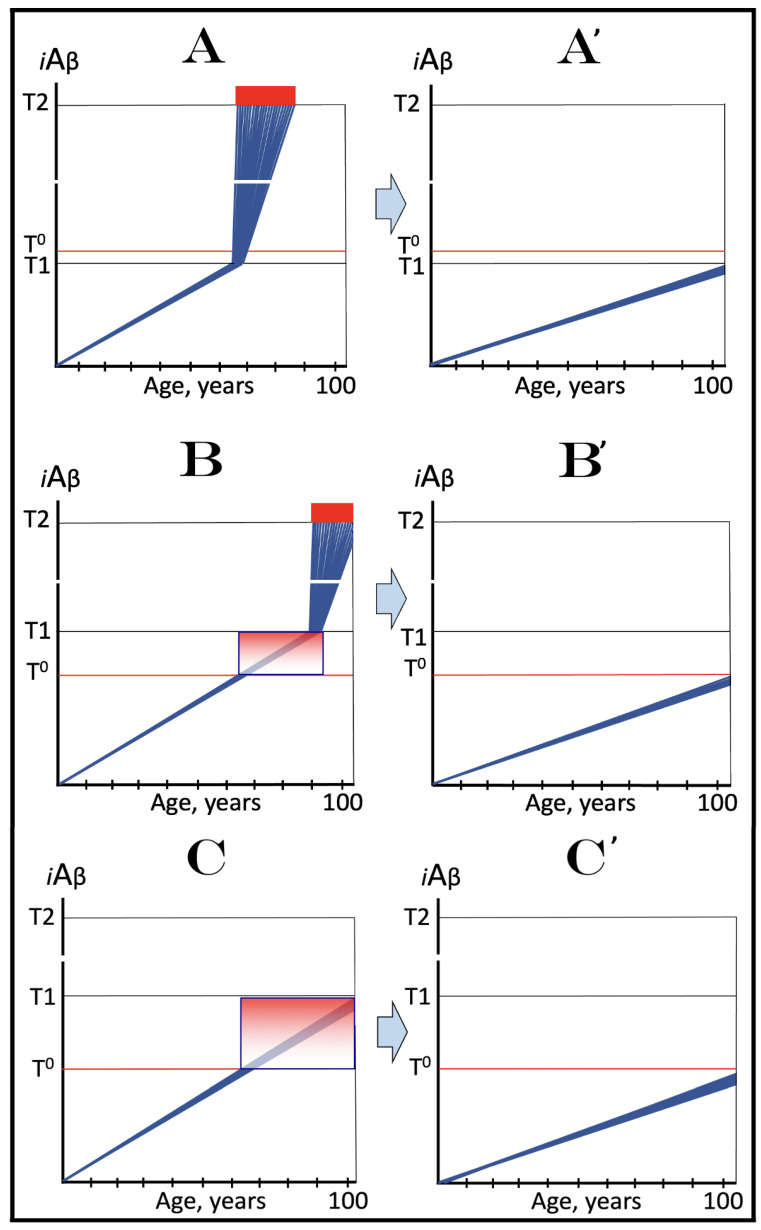
**The Icelandic AβPP mutation protects from AD and AACD by lowering the influx of AβPP-derived iAβ and decelerating its rate of accumulation**. *Blue lines*: Intraneuronal Aβ, iAβ. ***T*^0^**: Threshold of cellular concentration of AβPP-derived iAβ triggering the neuronal damage, which manifests as AACD. ***T1***: Threshold of cellular concentration of iAβ triggering the activation of PKR and/or HRI kinases, phosphorylation of eIF2α, the elicitation of the neuronal ISR, the initiation of the AβPP-independent C99 production pathway, and the commencement of AD; its extent is the only variable in the present figure. ***T2***: Cellular concentration of iAβ triggering neuronal death via apoptosis or necroptosis. *Red box*: Apoptotic zone, defined as a range of cellular concentrations of iAβ within which cells neuronal cells committed apoptosis or necroptosis or are dead. *Pink boxes*: The range of cellular concentrations of AβPP-derived iAβ concentrations between the T^0^ and the highest extent reached by AβPP-derived iAβ or the T1 threshold that support the progression of AACD. The condition commences with the crossing of the T^0^ threshold and morphs into AD with the T1 crossing; it can occur only if the extent of the T^0^ threshold is smaller than that of the T1. *Panels* (**A**–**C**) illustrate principal forms of conventional AD and/or AACD that occur in wild-type AβPP carriers. *Panel* (**A**): The T1 threshold is below the AACD-triggering T^0^ threshold; no AACD occurs. Upon the T1 crossing the neuronal ISR is elicited, the AβPP-independent C99 and iAβ generation pathway activated, and conventional AD commences. *Panel* (**B**): The extent of the T^0^ threshold is smaller than that of the T1. When AβPP-derived iAβ crosses the T^0^, AACD commences and morphs into conventional AD upon the T1 crossing. *Panel* (**C**): The T1 threshold is not reached within the lifetime of the individual. When AβPP-derived iAβ crosses the T^0^ threshold, AACD commences and persists for the remaining lifetime. *Panels* (**A’**–**C’**) illustrate the protective effect of the Icelandic AβPP. In each panel the influx of AβPP-derived iAβ is reduced and its rate of accumulation lowered. *Panel* (**A’**): AβPP-derived iAβ does not reach the T1 threshold within the lifetime of the individual; no AD occurs. *Panels* (**B’**,**C’**): AβPP-derived iAβ reaches neither the T^0^ nor T1 threshold within the lifetime of the individual; neither AACD nor conventional AD occurs.

The above-described effects of the Icelandic mutation are illustrated in Figure 11. Panels A through C depict three principal scenarios of the disease in wild-type individuals. In Panel A, the extent of the T^0^ threshold is greater than that of T1 and AD but no AACD occurs. In Panel B of Figure 11, AD is preceded by AACD. In Panel C the extent of the T1 threshold is such that it is not reached by AβPP-derived iAβ within the lifetime of the individual and no AD occurs. AACD, on the other hand, commences following the T^0^ crossing and persists for the remaining lifetime. Panels A’ through C’ of Figure 11 show effects of the Icelandic mutation in the above-described scenarios. In all, the rate of accumulation of AβPP-derived iAβ is reduced. In none does it reach either the T^0^ or T1 thresholds. Consequently, neither AD nor AACD occur in all three scenarios, hence the Icelandic mutation confers protection from both conditions.

## 35. Icelandic AβPP Mutation as a Guide for AD and AACD Therapies

**Figure 12:**
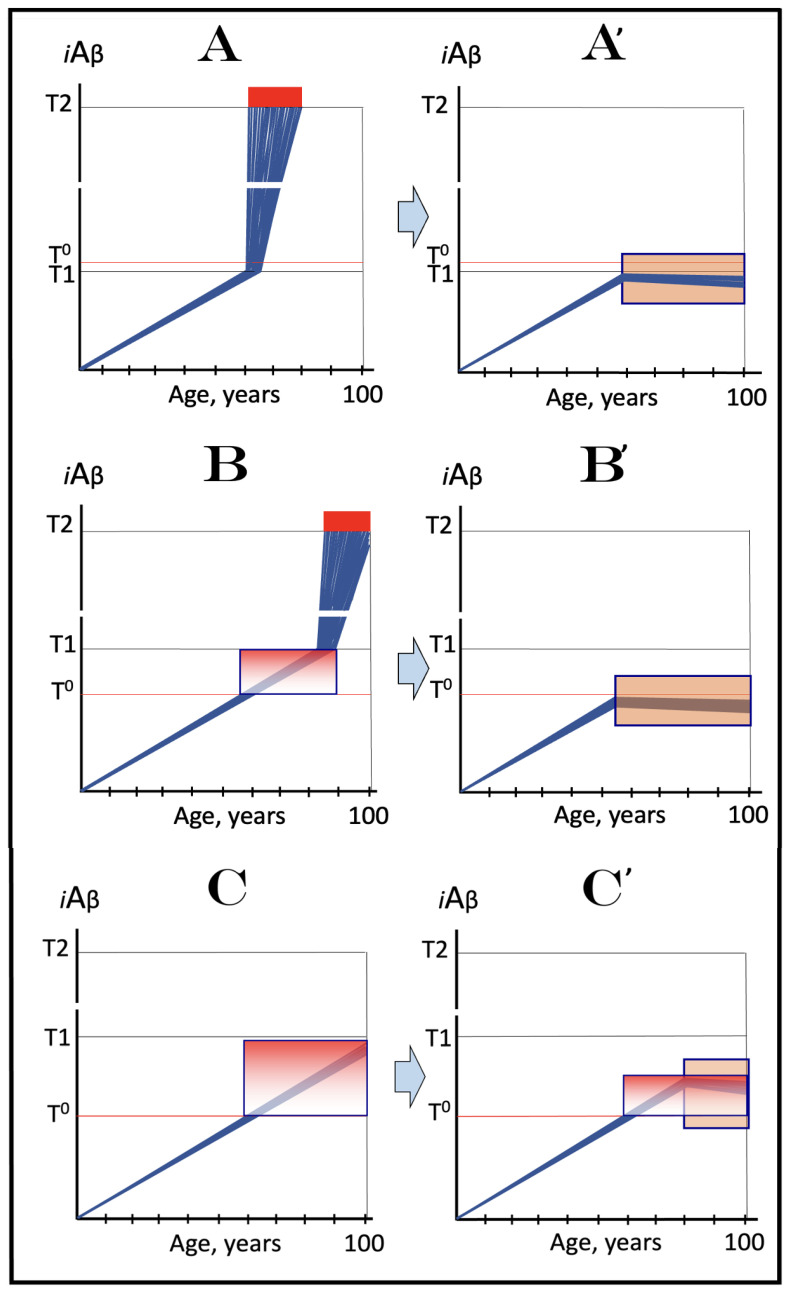
**Therapeutic strategies for conventional AD and AACD suggested by the Icelandic AβPP mutation**. *Blue lines*: Intraneuronal Aβ, iAβ. ***T*^0^**: Threshold of cellular concentration of AβPP-derived iAβ triggering the neuronal damage, which manifests as AACD. ***T1***: Threshold of cellular concentration of iAβ triggering the activation of PKR and/or HRI kinases, phosphorylation of eIF2α, the elicitation of the neuronal ISR, the initiation of the AβPP-independent C99 production pathway, and the commencement of AD; its extent is the only variable in the present figure. ***T2***: Cellular concentration of iAβ triggering neuronal death via apoptosis or necroptosis. *Red box*: Apoptotic zone, defined as a range of cellular concentrations of iAβ within which cells neuronal cells committed apoptosis or necroptosis or are dead. *Pink boxes*: The range of cellular concentrations of AβPP-derived iAβ concentrations between the T^0^ and the highest extent reached by AβPP-derived iAβ or the T1 threshold that support the progression of AACD. The condition commences with the crossing of the T^0^ threshold and morphs into AD with the T1 crossing; it can occur only if the extent of the T^0^ threshold is smaller than that of the T1. *Orange boxes*: Duration of the treatment with the drug that reverses the rate of accumulation of AβPP-derived iAβ. *Panels* (**A**–**C**): Dynamics of iAβ accumulation in AD-affected neurons of the untreated AD and/or AACD patients. *Panel* (***A***): The T1 threshold is below the AACD-triggering T^0^ threshold; no AACD occurs. Upon the T1 crossing, the neuronal ISR is elicited, the AβPP-independent C99 and iAβ generation pathway activated, and conventional AD commences. *Panel* (**B**): The extent of the T^0^ threshold is smaller than that of the T1. When AβPP-derived iAβ crosses the T^0^, AACD commences and morphs into conventional AD upon the T1 crossing. *Panel* (**C**): The T1 threshold is not reached within the lifetime of the individual. When AβPP-derived iAβ crosses the T^0^ threshold, AACD commences and persists for the remaining lifetime. *Panels* (**A’**–**C’**): Dynamics of AβPP-derived iAβ in individuals treated with a drug that suppresses its influx and reveres its rate of accumulation. In Panels (**A’**,**B’**), the drug is administered prior to the crossing of the T^0^ and/or T1 thresholds. Neither threshold is reached for the duration of the treatment, nor does AD or AACD occur. In Panel (**C’**), the drug is administered after the T^0^ crossing but prior to the T1 crossing. The drug reverses the accumulation of AβPP-derived iAβ and stops the progression of AACD. The T1 threshold would not be reached, and conventional AD would not occur for the duration of the treatment.

Protective effects of the Icelandic mutation provide the ultimate guidance for prevention of AD and AACD: suppress the rate of accumulation of AβPP-derived iAβ, prevent it from reaching the T^0^ and T1 thresholds, and you would prevent both conditions. To emulate the Icelandic mutation, the suppression of accumulation of AβPP-derived iAβ should commence in infancy. This, however, is not entirely practical. The later in life the treatment starts, the more challenging it becomes. If at the time of commencement of the treatment levels of AβPP-derived iAβ were relatively close to the T^0^ and/or T1 thresholds, the reduction in the rate of its accumulation would not be sufficient; the rate would have to be actually reversed in order to achieve the protective effect. This strategy is illustrated in Figure 12. Panel A shows the dynamics of iAβ accumulation in the wild-type individual. The extent of the T^0^ threshold is greater than that of the T1, and AD commences upon the crossing of the latter. In Panel A’, the individual is treated prior to the T1 crossing. The rate of accumulation of AβPP-derived iAβ is reversed (or reduced to zero). The T1 threshold would not be crossed, and AD would not occur for the duration of the treatment. In Panel B, AD is preceded by AACD. The latter commences upon the T^0^ crossing and morphs into the latter when the T1 threshold is crossed. In Panel B’, the treatment starts prior to the T^0^ crossing. The rate of accumulation of AβPP-derived iAβ is reversed, the T^0^ and T1 thresholds would not be crossed and AACD and AD would not occur for the duration of the treatment. In Panel C of Figure 12, the T1 threshold is not crossed within the lifespan of the individual. AACD commences with the T^0^ crossing and persists for the remaining lifetime. If treatment were to begin prior to the T^0^ crossing, AACD could be prevented. In Panel C’, the treatment starts after the T^0^ crossing. The rate of accumulation of AβPP-derived iAβ is reversed and the progression of AACD stopped for the duration of the treatment.

## 36. ACH-Based AD Drugs Could Be Effective If Administered Preventively

The preventive strategies discussed in the preceding section can potentially be enacted with ACH-based drugs. ACH-based drugs are agents designed, under the ACH guidance, to deplete extracellular Aβ, the presumed cause of AD (in the ACH). They include drugs that directly interact with extracellular Aβ, such as Aβ-specific antibodies, as well as drugs suppressing the production and, subsequently, secretion of Aβ, such as BACE1 inhibitors. Since, as discussed above, the ACH appears to be unsustainable, it could be reasoned that ACH-based AD drugs would be ineffective. This, however, is not the case. Inadvertently, ACH-based drugs should be effective in the prevention (but not the treatment; as discussed below) of conventional AD in the framework of the ACH2.0. This is because, by impacting levels of extracellular Aβ or the production of Aβ in the AβPP proteolytic pathway, they affect the rate of accumulation of AβPP-derived iAβ. The logic is apparent. The smaller the pool of extracellular Aβ, the lower the rates of its cellular uptake, of the influx of iAβ, and of the accumulation of AβPP-derived iAβ. In cases of the suppression of Aβ production in the AβPP proteolytic pathway, e.g., with BACE1 inhibitors, the impact is even greater: not only would the rate of the importation of extracellular Aβ be lowered due to the reduction of its pool, the retention of newly generated AβPP-derived iAβ would also be reduced. Therefore, if administered preventively, i.e., prior to the crossing of the T1 threshold, ACH-based drugs would either reduce or reverse the rate of accumulation of AβPP-derived iAβ. This would either delay or prevent both the T1 crossing and the commencement of AD. Likewise, if administered prior to the T^0^ crossing, ACH-based drugs could delay or preclude the occurrence of AACD.

**Figure 13:**
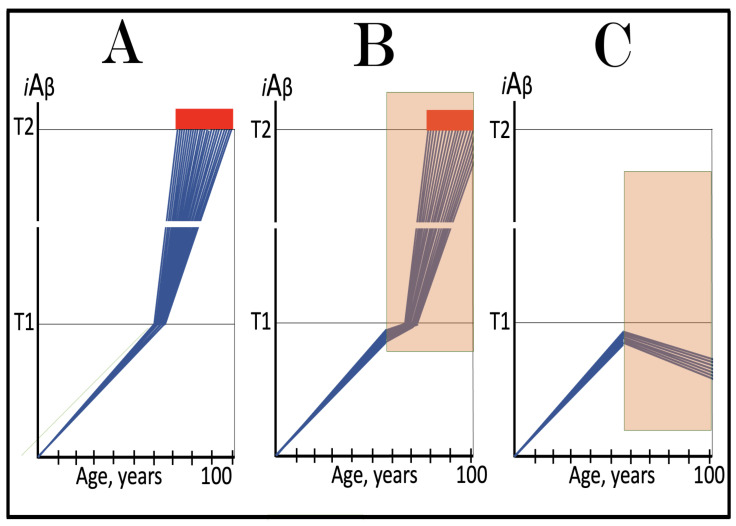
**ACH-based AD drugs could be effective if administered preventively**. *Blue lines*: Intraneuronal Aβ, iAβ. ***T1***: Threshold of cellular concentration of iAβ triggering the activation of PKR and/or HRI kinases, phosphorylation of eIF2α, the elicitation of the neuronal ISR, the initiation of the AβPP-independent C99 production pathway, and the commencement of AD. ***T2***: Cellular concentration of iAβ triggering neuronal death via apoptosis or necroptosis. *Red box*: Apoptotic zone, defined as a range of cellular concentrations of iAβ within which cells neuronal cells committed apoptosis or necroptosis or are dead. *Orange boxes*: Duration of the treatment with the ACH-based drug that suppresses or reverses the rate of accumulation of AβPP-derived iAβ. *Panels* (**A**–**C**): Dynamics of iAβ accumulation in AD-affected neurons of the untreated and treated AD and/or AACD patients. *Panel* (**A**): Dynamics of the accumulation of iAβ in the untreated individual. AβPP-derived iAβ accumulates via two physiological mechanisms described in the main text. When it crosses the T1 threshold, PKR and/or HRI kinases are activated, eIF2α phosphorylated, the neuronal ISR elicited, the AβPP-independent C99 production pathway initiated, and conventional AD commences. *Panel* (**B**): The drug suppresses the influx of AβPP-derived iAβ, but its accumulation continues albeit at a reduced rate. Eventually, subject to sufficient longevity, it would cross the T1 threshold. The neuronal ISR would be elicited, the AβPP-independent C99 production pathway would be activated, and conventional AD would commence. In this scenario the drug delays the occurrence of AD. *Panel* (**C**): The drug suppresses the influx of AβPP-derived iAβ and reverses its rate of accumulation. The T1 threshold would not be reached, and conventional AD would not occur for the duration of the treatment. In this scenario the drug prevents the occurrence of conventional AD.

The potential preventive effects of the ACH-based AD drugs are illustrated in Figure 13. Panel A of Figure 13 depicts the dynamics of accumulation of iAβ and progression of AD in untreated individuals. In the first stage AβPP-derived iAβ accumulates and crosses the T1 threshold. PKR and/or HRI kinases are activated, eIF2α phosphorylated at its Ser51, the neuronal ISR elicited, and the AβPP-independent C99 (and iAβ in this version of the ACH2.0) production pathway initiated, and iAβ reaches the AD pathology-causing range; when it crosses the T2 threshold, neurons commit apoptosis. In Panel B of Figure 13, the ACH-based drug is administered prior to the T1 crossing. The influx of AβPP-derived iAβ is reduced but the reduction in the influx of AβPP-derived iAβ is such that its rate of accumulation is only lowered, not reversed. Its level reaches and crosses the T1 threshold; AβPP-independent C99 and iAβ production pathway is activated and AD commences. In this scenario the treatment does not prevent AD but only delays its occurrence. In Panel C of Figure 13, the administration of the ACH-based drug also commenced prior to the T1 crossing. The influx of AβPP-derived iAβ is reduced and the rate of its accumulation is reversed. The T1 threshold will not be crossed, and conventional AD will not occur for the duration of the treatment.

## 37. Modulators of Gamma-Secretase Could Elevate the T1 Threshold and Thus Be Potentially Effective in the Prevention of Conventional AD

Inhibitors of gamma-secretase are, potentially, ACH-based drugs for the same reason that inhibitors of beta-secretase are; both would reduce the production of Aβ in the AβPP proteolytic pathway and thus decrease its extracellular pool. This approach was indeed attempted [213,214,215] but it produced highly deleterious outcomes and was abandoned. Eventually, the reason for this failure became apparent. Gamma-secretase is a component of the Notch signaling pathway. As such, it has hundreds of targets and therefore cannot be interfered with without adverse effects. Gamma-secretase cleaves at multiple positions within a short segment of the C99 fragment of AβPP, and attempts have been made to develop modulators of gamma-secretase that would shift the position of the cleavage site toward shorter, more benign versions of Aβ. These efforts were met with variable success [215,216,217,218]. Like other ACH-based AD drugs, modulators of gamma-secretase would also be effective in the framework of the ACH2.0, but for different reasons. These modulators are unlikely to change either the size of the extracellular Aβ pool or, apparently, the efficiency of its cellular uptake. What they can do, however, is to elevate the extent of the T1 threshold. The logic of this assertion is the same as in the case iAβ42, which lowers the extent of the T1 because it is relatively (to other iAβ variants) more toxic and less of its needed to achieve a certain level of cellular stress. With shorter, relatively less toxic iAβ species, more of it would be needed to attain the required level of cellular stress, and the extent of the T1 threshold would increase. This is a scenario that was considered in Section 30 above. In the individual treated with such a modulator the crossing of the T1 thresholds and the commencement of conventional AD would be either delayed or prevented.

**Figure 14:**
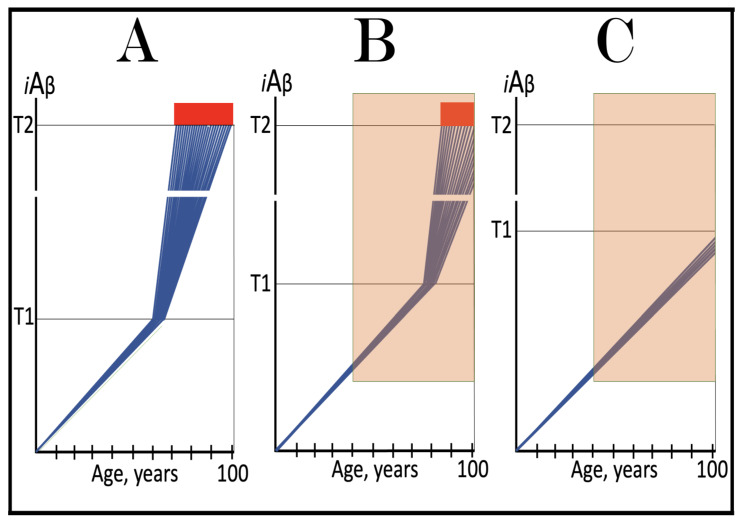
**Modulators of gamma-secretase may potentially protect from conventional AD via the elevation of the T1 threshold**. *Blue lines*: Intraneuronal Aβ, iAβ. ***T1***: Threshold of cellular concentration of iAβ triggering the activation of PKR and/or HRI kinases, phosphorylation of eIF2α, the elicitation of the neuronal ISR, the initiation of the AβPP-independent C99 production pathway, and the commencement of AD. ***T2***: Cellular concentration of iAβ triggering neuronal death via apoptosis or necroptosis. *Red box*: Apoptotic zone, defined as a range of cellular concentrations of iAβ within which cells neuronal cells committed apoptosis or necroptosis or are dead. *Orange boxes*: Duration of the treatment with the gamma-secretase modulating drug that shifts the production of Aβ toward shorter, more benign species. *Panel* (**A**): Dynamics of the accumulation of iAβ in the untreated individual. AβPP-derived iAβ accumulates via two physiological mechanisms described in the main text. When it crosses the T1 threshold, PKR and/or HRI kinases are activated, eIF2α phosphorylated, the neuronal ISR elicited, the AβPP-independent C99 production pathway initiated, and conventional AD commences. *Panel* (**B**): In the presence of the drug shorter, more benign species of Aβ are produced and accumulated as AβPP-derived iAβ. More of it is needed to attain the same level of cellular toxicity and cellular stress than with regular Aβ species. Consequently, the extent of the T1 threshold and the timing of its crossing by AβPP-derived iAβ increase. In this scenario the treatment delays the commencement of conventional AD. *Panel* (**C**): The treatment-caused increase in the extent of the T1 threshold is such that it is not reached, and conventional AD does not occur for the duration of the treatment. In this scenario, the drug prevents the occurrence of AD.

The above scenario is illustrated in Figure 14. Panel A of Figure 14 depicts the accumulation of iAβ and progression of AD in the untreated individual. Panel B of Figure 14 shows the same parameters in the individual treated with a modulator of gamma-secretase. The rate of accumulation of AβPP-derived iAβ is not affected. But because it is less toxic, more of it is needed to trigger the elicitation of the neuronal integrated stress response and the extent of the T1 threshold is elevated. Consequently, it takes longer to reach and cross it. AD commences and progresses, but it is delayed. In Panel C of Figure 14, the elevation of the extent of the T1 threshold in the individual treated with a modulator of gamma-secretase is such that AβPP-derived iAβ does not reach it within the lifetime of the treated individual. Neither the T1 crossing, nor conventional AD would occur in this scenario. It should be noted that if AD does occur in the treated individual (Panel B of Figure 14), the extent of the T2 threshold would likely also be elevated due to the lover toxicity of iAβ produced independently of AβPP. In such a case, gamma-secretase modulators could be effective in symptomatic AD but only in the initial version of the ACH2.0 under discussion; they would be completely ineffective in symptomatic AD in the current Version Three of the ACH2.0 (see Section 66 below).

## 38. ACH-Based Drugs Are Completely Ineffective in Symptomatic AD

Whereas, as described in two preceding sections, ACH-based drugs could potentially be effective in the prevention of the disease, it can be stated categorically that they cannot work, in any way, form, or shape in symptomatic AD. More precisely, they cannot work following the crossing of the T1 threshold. The reason for this is the operation of the AβPP-independent C99 and (in this version of the ACH2.0) iAβ production pathway. From the instance of its activation by the neuronal integrated stress response, this pathway is completely independent from the influx of AβPP-derived iAβ (or from the absence thereof). As discussed in Section 26 above, it is fully autonomous and self-sustainable: its output propagates the neuronal ISR and thus perpetuates its own activity. Moreover, the output of the AβPP-independent C99 and iAβ production pathway is orders of magnitude greater than that of the AβPP proteolytic pathway. Therefore, even if the influx of AβPP-derived iAβ completely ceases, this would affect neither the continuous operation of the AβPP-independent C99 and iAβ production pathway nor the progression of AD.

**Figure 15:**
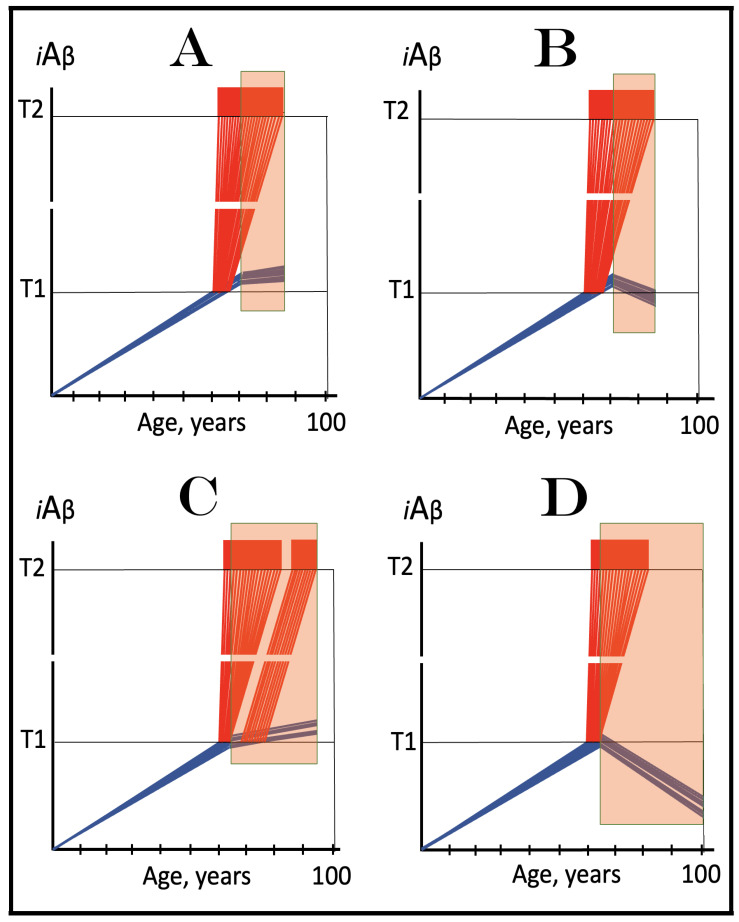
**ACH-based drugs are ineffective when the AβPP-independent C99/iAβ production pathway is operational**. *Blue lines*: AβPP-derived iAβ. *Red lines*: iAβ generated independently of AβPP. ***T1***: Threshold of cellular concentration of iAβ triggering the activation of PKR and/or HRI kinases, phosphorylation of eIF2α, the elicitation of the neuronal ISR, the initiation of the AβPP-independent C99 production pathway, and the commencement of AD. ***T2***: Cellular concentration of iAβ triggering neuronal death via apoptosis or necroptosis. *Red box*: Apoptotic zone, defined as a range of cellular concentrations of iAβ within which cells neuronal cells committed apoptosis or necroptosis or are dead. *Orange boxes*: Duration of the treatment with the ACH-based drug that suppresses or reverses the rate of accumulation of AβPP-derived iAβ. *Panel* (**A**): AβPP-derived iAβ accumulates via two physiological mechanisms described in the main text. When it crosses the T1 threshold PKR and/or HRI kinases are activated, eIF2α phosphorylated, the neuronal ISR elicited, the AβPP-independent C99 production pathway initiated, and conventional AD commences. ACH-based drugs administered at this point reduce significantly the rate of accumulation of AβPP-derived iAβ. They have, however, little, if any, effect on both the production of iAβ in the AβPP-independent pathway and the progression of the disease. *Panel* **(B**): As in the preceding panel, the ACH-based drug is administered when all affected neurons have already crossed the T1 threshold, and the AβPP-independent C99 production pathway has already been activated. The reduction in the influx of AβPP-derived iAβ is such that the rate of its accumulation is reversed. Since the operation of the self-sustainable AβPP-independent C99 production pathway is not affected, this reversal is inconsequential for the progression of the disease. Panels (**C**,**D**): The ACH-based drug is administered when a fraction of the affected neurons has not yet crossed the T1 threshold. In over-T1 neurons, the AβPP-independent C99/iAβ production pathway has been activated, and the drug does not affect its operation. In *Panel* (**C**), the drug reduces the influx of AβPP-derived iAβ, but its accumulation continues. The initially under-T1 neurons eventually cross the T1 threshold, the AβPP-independent C99/iAβ production pathway is activated, and cellular AD pathology commences. In *Panel* (**D**), the drug reverses the rate of accumulation of AβPP-derived iAβ. The initially under-T1 neurons do not cross the T1 threshold and remain AD pathology-free for the duration of the treatment.

The above considerations are illustrated in Figure 15. In this figure, the mode of the production of iAβ is color-coded. AβPP-derived iAβ is presented as blue lines whereas iAβ generated independently of AβPP (in the same neuronal cells) is depicted in red. In Panel A of Figure 15, the administration of the ACH-based drug starts when all affected neurons have already crossed the T1 threshold and AD commenced. The influx of AβPP-derived iAβ is reduced and its rate of accumulation is lowered. This, however, has no effect on the continuous operation of the AβPP-independent C99 and iAβ production pathway and on the progression of AD. The only difference in Panel B of Figure 15 is that the ACH-based drug-mediated reduction in the influx of AβPP-derived iAβ is such that the rate of its accumulation is reversed, and its levels are decreasing. This however does not change the outcome: the AβPP-independent C99 and iAβ production pathway remains fully operative and the progression of AD is unimpeded.

Two conceptually different scenarios are presented in Panels C and D of Figure 15. In both panels, the administration of the ACH-based drug commences at the time when either symptoms of AD have manifested or its biomarker(s) occurred, but a fraction of the affected neurons have not yet crossed the T1 threshold. In both panels, in the neurons that crossed the T1 threshold the activity of the AβPP-independent C99 and iAβ production pathway has commenced and remains unaffected and unimpeded by the drug. The rate of accumulation of AβPP-derived iAβ, however, is affected. In Panel C of Figure 15, it is reduced but accumulation continues. Eventually, levels of AβPP-derived iAβ in the initially under-T1 neurons would reach the T1 threshold. At this point the AβPP-independent C99/iAβ production pathway would be activated and the progression of cellular AD pathology would commence. The drug would be effective in delaying the onset of AD pathology in the initially under-T1 neuronal fraction, but its effect would be only marginal because this neuronal fraction is marginal.

In Panel D of Figure 15, the ACH-based drug-mediated reduction in the influx of AβPP-derived iAβ is such that the rate of its accumulation is reversed. Its levels in the initially under-T1 neurons are decreasing and would not reach the T1 threshold for the duration of the treatment. The drug would be effective in preventing the onset of AD pathology in the initially under-T1 neurons. Its effect, however, would be marginal because the fraction of the initially under-T1 neurons is.

## 39. Validation (9): Effects of Lecanemab and Donanemab in Early AD Are Due to the Timing of Their Administration: They Affect Preventively Only a Marginal Fraction of Neurons That Has Not Yet Crossed the T1 Threshold

The outcomes predicted in scenarios depicted in Panels C and D of Figure 15 in the preceding section were, in fact, verified in clinical trials of two ACH-based drugs, lecanemab and donanemab [29,30,31,32,33]. Both drugs are monoclonal antibodies designed to reduce levels of extracellular Aβ. Both trials produced positive, however marginal, results: cognitive decline of treated patients continued but at a decreased rate. These results were interpreted as an affirmation of the notion that the reduction in levels of extracellular Aβ reduced its adverse cytotoxic effect. However, this interpretation is inconsistent with the outcomes of earlier trials of ACH-based drugs (conducted at more advanced stages of AD) where levels of extracellular Aβ were reduced to no lesser extent without any positive outcomes whatsoever. The interpretation of the results of lecanemab and donanemab clinical trials in terms of the ACH2.0 resolves this controversy.

The selection of subjects in both trials was based on the appearance of a serum biomarker unavailable in earlier clinical trials of ACH-based AD drugs. This biomarker (phosphorylated tau) is currently one of the earliest if not the earliest biomarker of AD. Accordingly, subjects of these trials were at very early stages of the disease, substantially earlier than in any previous clinical AD trial. As argued above, ACH-based drugs are utterly ineffective following the T1 crossing. It follows from their marginal effect that at the time of the commencement of drugs’ administration a fraction of the affected neurons has not yet crossed the T1 threshold and that the observed outcomes resulted from the impact of the drugs on this neuronal fraction (referred to as “initially under-T1”). Moreover, it follows that both drugs acted not curatively (i.e., not on neurons undergoing AD pathology) but only preventively (i.e., preventing the onset of AD pathology).

**Figure 16:**
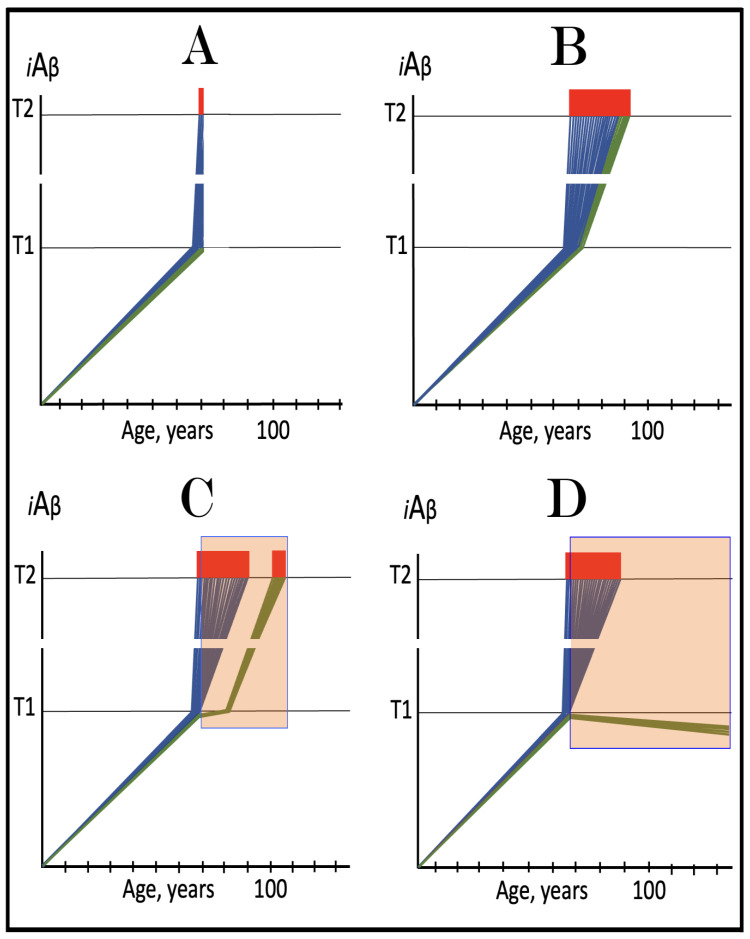
**Effect of Lecanemab and Donanemab in early AD: only a marginal fraction of neurons that have not yet crossed the T1 threshold is affected**. *Blue lines*: Intraneuronal Aβ, iAβ. *Green lines*: Levels of iAβ in the neurons that have not yet crossed the T1 threshold at the commencement of the treatment. ***T1***: Threshold of cellular concentration of iAβ triggering the activation of PKR and/or HRI kinases, phosphorylation of eIF2α, the elicitation of the neuronal ISR, the initiation of the AβPP-independent C99 production pathway, and the commencement of AD. ***T2***: Cellular concentration of iAβ triggering neuronal death via apoptosis or necroptosis. *Red box*: Apoptotic zone, defined as a range of cellular concentrations of iAβ within which cells neuronal cells committed apoptosis or necroptosis or are dead. *Orange boxes*: Duration of the treatment with lecanemab or donanemab. *Panel* (**A):** The initial state of levels of iAβ at the commencement of the drug’s administration. The bulk of the affected neurons have crossed the T1 threshold. The neuronal ISR has been elicited and the AβPP-independent C99/iAβ production pathway has been activated. Some neurons have reached the T2 threshold and AD symptoms have manifested. *Panel* (**B**): The evolution of the initial state in the untreated AD patient. All affected neurons cross the T1 threshold. The disease progresses and reaches the end stage. *Panels* (**C**,**D**): The evolution of the initial state in treated patients. The drug has no effect whatsoever on the operation of the self-sustainable AβPP-independent C99/iAβ production pathway; it affects only the rate of accumulation of AβPP-derived iAβ. *Panel* (**C**): The drug suppresses the influx of AβPP-derived iAβ, but its accumulation continues, albeit at a reduced rate. Eventually, subject to sufficient longevity, it would cross the T1 threshold in the initially under-T1 neurons. The neuronal ISR would be elicited, the AβPP-independent C99 production pathway would be activated, and cellular AD pathology would commence. *Panel* (**D**): The drug suppresses the influx of AβPP-derived iAβ and reverses its rate of accumulation. The T1 threshold would not be reached, and AD pathology would not occur in the initially under-T1 neurons for the duration of the treatment. In both scenarios, however, the effect is only marginal because the fraction of the drug-affected neurons is.

The above interpretation is illustrated in Figure 16. Panel A of Figure 16 depicts the initial (i.e., at the time of the commencement of drug’s administration) state of the levels of iAβ in affected neurons. In most neurons the T1 threshold has been crossed, the AβPP-independent C99/iAβ production pathway activated, and the progression of cellular AD pathology commenced. A fraction of the neurons is, however, still under-T1 at this stage. Panel B of Figure 16 shows how the initial state would evolve in the untreated patient. The initially under-T1 neurons would cross the T1 threshold; cellular AD pathology would progress in all affected neurons, and the disease would reach its end stage. In Panel C of Figure 16, the ACH-based drug is ineffective in the over-T1 neurons where the AβPP-independent C99/iAβ production pathway is operative. In the initially under-T1 neuronal fraction, it reduces the influx of AβPP-derived iAβ and lowers the rate of its accumulation. Eventually, however, this neuronal fraction would cross the T1 threshold, and the progression of AD pathology would commence. In Panel D of Figure 16, the drug is also affecting only the initially under-T1 neuronal fraction. The reduction in the influx of AβPP-derived iAβ in these neurons is such that the rate of its accumulation is reversed, and its levels are declining. The T1 threshold would not be crossed, and AD pathology would not commence in these neurons for the duration of the treatment. In the scenarios depicted in both Panels C and D, the effect would be marginal because the drug affects only a marginal neuronal fraction.

## 40. ANY ACH-Based Drug Has a Proverbial Snowball’s in Hell Chance of Being Effective in Advanced AD

It is important to emphasize that lecanemab and donanemab, discussed in the preceding section, do not act conceptually differently or more efficiently than other ACH-based drugs. They exhibited their marginal effect (and therefore were FDA-approved as AD drugs) only because their makers were fortunate to be able to conduct trials at the early enough stages of AD (guided by phosphorylated tau serum biomarker not available previously) when a marginal neuronal subpopulation did not yet cross the T1 threshold and could be treated preventively. It can be predicted with considerable certainty that most, if not all, ACH-based drugs would yield similar results if tested at similarly early stages of AD.

The bottom line is that ACH-based drugs may have an effect only in situations where the AβPP-independent C99/iAβ production pathway is inoperative. This is why they performed brilliantly in transgenic mouse models overexpressing human AβPP. In these models, for the reasons explained above, the AβPP-independent C99/iAβ generation pathway is inoperative, and they do not develop AD. Neurodegeneration and cognitive impairment exhibited by these models are the consequence of the neuronal ISR elicited (via PKR and/or HRI activation) by AβPP-derived iAβ accumulated over the T1 threshold. ACH-based drugs reduce the levels of iAβ to those below the T1 threshold and thus reverse the neuronal ISR, hence the effect.

This is why ACH-based drugs are legitimate AD and AACD preventive agents. Moreover, they may be effective in the treatment of AACD (where by definition, levels of iAβ are below the T1 threshold). What they principally cannot do, regardless of their effectiveness in depleting the extracellular Aβ pool or suppressing the production of Aβ in the AβPP proteolytic pathway, is be effective in settings where the AD Engine, i.e., the self-sustainable AβPP-independent C99/iAβ generation pathway is active. This is an unadulterated impossibility in terms of the ACH2.0; hence there is a “snowball in hell” chance of the ACH drugs being effective in symptomatic AD.

## 41. ACH2.0-Based AD Drugs: A Definition

ACH2.0-base AD drugs are the agents capable of the prevention and treatment of AD (and AACD) in terms of the ACH2.0 theory of the disease. Mechanistically, they are defined by their targets and on this basis can be divided into three categories. One comprises agents capable of direct interference with the operation of the AβPP-independent C99/iAβ production pathway. This pathway is the active core of the disease, its engine, and, therefore, constitutes the major therapeutic target. Presuming that the AβPP-independent C99/iAβ production pathway is underpinned by RNA-dependent amplification of AβPP mRNA, in authors’ opinion by far the most plausible scenario, targeting it is challenging. As described above (see Section 10), RNA-dependent mRNA amplification is a major physiological process crucial, for example, in erythropoiesis and in the deposition of extracellular matrix proteins, and therefore, it cannot be interfered with systemically without deleterious consequences. The only way to interfere with it is specifically, i.e., to target its specific substrate, in this case AβPP mRNA or the antisense AβPP RNA. This is feasible, and promising possibilities are discussed in detail in Section 79 below.

Another category includes drugs targeting the enabler and sustainer of the AβPP-independent C99/iAβ production pathway, namely the neuronal integrated stress response. The latter provides components essential for the operation of the former. Inhibition of the neuronal ISR would cease the production of these components and disable the AD-driving pathway. ISR inhibitors are available and their effect in the prevention and treatment of conventional AD are analyzed in the two following sections. The ISR is a major physiological survival tool and its systemic long-term inhibition is bound to be detrimental. On the other hand, transient administration of ISR inhibitors in concert with other therapies could be crucially important in successful treatment of AD, as described in Section 48 below.

The two categories above discuss approaches that would affect AD but not AACD. The third category of ACH2.0-based drugs would affect both. It includes the agents that deplete the trigger and the actual driver of the disease, iAβ (in this version of the ACH2.0, but the concept holds up in other ACH2.0 versions as well, as discussed below). Preventively, the depletion of iAβ (in this case AβPP-derived iAβ) would preclude the crossing of the T^0^ and T1 threshold and the occurrence of AACD and AD. With the AβPP-independent C99/iAβ production pathway operational, the depletion of iAβ (in this case mostly iAβ produced independently of AβPP) would deprive the disease of its driver and stop its progression. Importantly, the sufficient, below the T1 threshold, depletion of iAβ would also cease the propagation of the neuronal ISR and cause its reversal. This, in turn, would deprive the AβPP-independent C99/iAβ production pathway of its essential components and thus disable it. This opens up a possibility that is indeed remarkable. Following the transient depletion, and provided it is deep enough, the accumulation of iAβ would resume from a low baseline, supported only by the AβPP proteolytic pathway, and it may not reach the T1 threshold within the remaining lifetime of the treated individual. In such a case, a single, one-time-only iAβ depletion treatment may prevent the occurrence of AD (and AACD) in healthy individuals and stop the progression of the disease in AD patients. This possibility is further discussed in Section 44, Section 45, Section 46, Section 47 and Section 48 below.

## 42. Suppression of the Neuronal Integrated Stress Response in the Prevention of Conventional Alzheimer’s Disease

**Figure 17:**
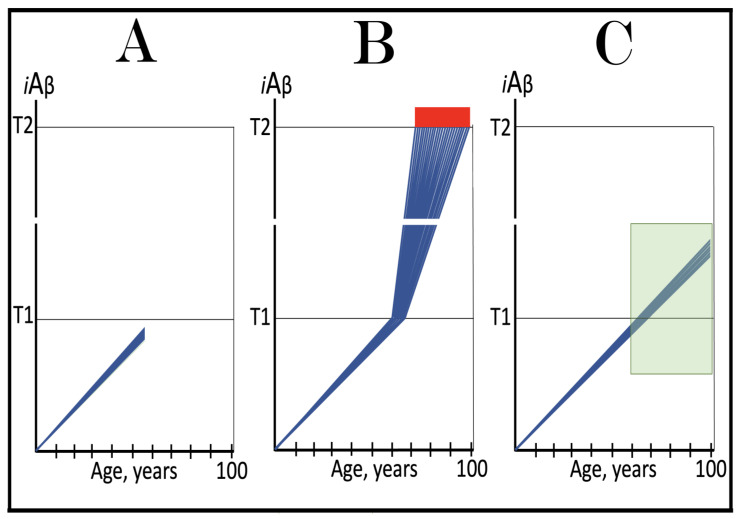
**Suppression of the neuronal integrated stress response in the prevention of conventional AD.** *Blue lines*: Intraneuronal Aβ, iAβ. ***T1***: Threshold of cellular concentration of iAβ triggering the activation of PKR and/or HRI kinases, phosphorylation of eIF2α, the elicitation of the neuronal ISR, the initiation of the AβPP-independent C99 production pathway, and the commencement of AD. ***T2***: Cellular concentration of iAβ triggering neuronal death via apoptosis or necroptosis. *Red box*: Apoptotic zone, defined as a range of cellular concentrations of iAβ within which cells neuronal cells committed apoptosis or necroptosis or are dead. *Green box*: Duration of the treatment with the ISR-inhibiting drug. *Panel* (**A**): The initial state of the levels of AβPP-derived iAβ in the neurons of the healthy individual who would develop AD if untreated. AβPP-derived iAβ has been accumulating but its levels are below the T1 threshold *Panel* (**B**): The evolution of the initial state in the untreated individual. AβPP-derived iAβ accumulates unimpeded and crosses the T1 threshold. This triggers activation of PKR and/or HRI, phosphorylation of eIF2α, elicitation of the neuronal ISR, and initiation of the AβPP-independent C99/iAβ production pathway. AD commences and progresses until it reaches its end stage. *Panel* (**C**): The evolution of the initial state in the individual treated with the ISR-inhibiting drug. AβPP-derived iAβ reaches and crosses the T1 threshold but the neuronal ISR cannot be elicited. The influx of iAβ is supported solely by the AβPP proteolysis and it continues to accumulate at a slow pre-T1 crossing rate. Its level would not reach the AD pathology-causing range for the duration of the treatment.

Suppression of the neuronal ISR in order to prevent AD is an intuitive approach. Indeed, the AβPP-independent C99/iAβ production pathway cannot be activated and therefore AD cannot occur in the absence of the neuronal ISR. It follows that the prevention of the elicitation of the neuronal ISR would prevent the disease. This approach is illustrated in Figure 17. Panel A of Figure 17 shows the initial state of the levels of AβPP-derived iAβ in the neurons of the healthy individual who would develop AD if left untreated. Panel B of Figure 17 depicts the evolution of the initial state in the untreated individual. AβPP-derived iAβ accumulates unimpeded and reaches the T1 threshold. This triggers activation of PKR and/or HRI, phosphorylation of eIF2α, elicitation of the neuronal ISR, and initiation of the AβPP-independent C99/iAβ production pathway. AD commences and progresses until it reaches its end stage. Panel C of Figure 17 presents the evolution of the initial state in the individual treated with the ISR-inhibiting drug. AβPP-derived iAβ reaches and crosses the T1 threshold but the neuronal ISR cannot be elicited. The influx of iAβ is supported solely by the AβPP proteolysis and it continues to accumulate at a slow pre-T1 crossing rate. Its level would not reach the AD pathology-causing range for the duration of the treatment.

## 43. Suppression of the Neuronal Integrated Stress Response in the Treatment of Conventional Alzheimer’s Disease

**Figure 18:**
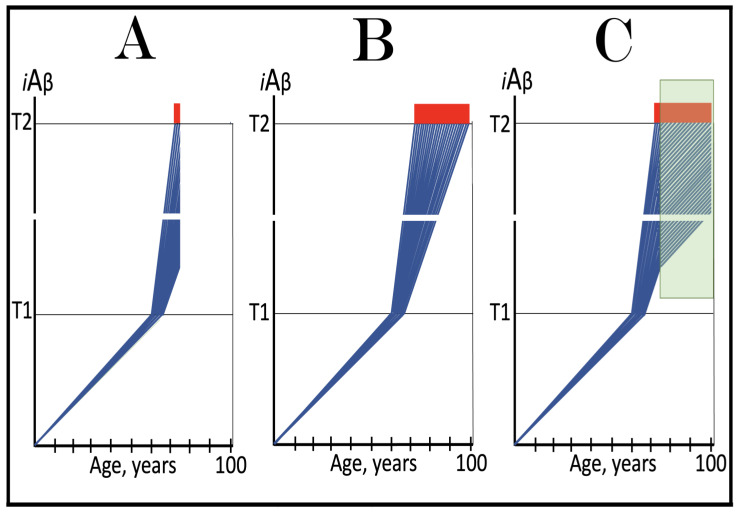
**Suppression of the neuronal integrated stress response in the treatment of conventional AD.** *Blue lines*: Intraneuronal Aβ, iAβ. ***T1***: Threshold of cellular concentration of iAβ triggering the activation of PKR and/or HRI kinases, phosphorylation of eIF2α, the elicitation of the neuronal ISR, the initiation of the AβPP-independent C99 production pathway, and the commencement of AD. ***T2***: Cellular concentration of iAβ triggering neuronal death via apoptosis or necroptosis. *Red box*: Apoptotic zone, defined as a range of cellular concentrations of iAβ within which cells neuronal cells committed apoptosis or necroptosis or are dead. *Green box*: Duration of the treatment with the ISR-inhibiting drug. *Panel* (**A**): The initial state of the levels of AβPP-derived iAβ in the neurons of the AD patient. AβPP-derived iAβ has crossed the T1 threshold in all affected neurons, the neuronal ISR has been elicited, the AβPP-independent C99/iAβ production pathway activated, the T2 threshold crossed in a neuronal fraction, and AD symptoms have manifested. *Panel* (**B**): The disease progresses and reaches its end stage. *Panel* (**C**): The evolution of the initial state in the individual treated with the ISR-inhibiting drug. The supply of components essential for the activity of the AβPP-independent C99/iAβ production pathway stops and it operation ceases. The accumulation of iAβ, however, continues but at a slow pre-T1 crossing rate. The rate of the progression of the disease is reduced for the duration of the treatment.

In the treatment of conventional AD, the administration of the ISR-inhibiting drug commences when the AβPP-independent C99/iAβ production pathway is operational and the disease progresses. This approach is illustrated in Figure 18. Panel A of Figure 18 depicts the initial state of the levels of iAβ in the affected neurons of the AD patient. At this point AβPP-derived iAβ has crossed the T1 threshold in all affected neurons, the neuronal ISR has been elicited, AβPP-independent C99/iAβ production pathway activated, and AD symptoms have manifested. Left untreated, the disease would progress until it reaches the end stage, as shown in Panel B of Figure 18. Panel C of Figure 18 shows the evolution of the initial state in the presence of the ISR-inhibiting drug. The neuronal ISR is suppressed and no longer supplies the components required for the operation of the AβPP-independent C99/iAβ production pathway. The operation of the pathway ceases. However, the accumulation of iAβ, supported now only by the AβPP proteolysis, continues, albeit at a slow pre-T1 crossing rate. The progression of AD does not stop; it continues but at a significantly lower rate for the duration of the treatment.

Importantly, in both the present and the preceding sections the beneficial effect of the suppression of the neuronal ISR requires a continuous long-term administration of the drug. However, the long-term systemic inhibition of the ISR is, apparently, not feasible. The ISR is a major physiological cellular survival tool, and its systemic inhibition is likely to cause severe adverse effects. Such an outcome is strongly indicated by studies of eIF2α Ser51Ala KI mice [219]. Ser51 of aIF2α is the amino acid residue, which is phosphorylated in the integrated stress response. If the phosphorylation in this position were prevented, there would be no ISR. This is precisely what occurs when Ser51 is replaced by alanine. Ala51 of eIF2α cannot be phosphorylated and, consequently, the integrated stress response cannot be elicited in the carriers of this mutation. It is a predictive equivalent of the long-term systemic inhibition of the ISR, and the outcome is highly deleterious: mice with a homozygous mutation eIF2α Ser51Ala survived only up to eighteen hours after their birth [219]. These findings suggest that other types of drugs and alternative approaches are required for the prevention and treatment of AD.

## 44. BACE1 and BACE2 Activators Are Potential ACH2.0-Based iAβ Depletion Drugs

Any agent capable of specific degradation of iAβ is a potential ACH2.0-based drug. For example, directly acting iAβ-specific degradation agents such as appropriately designed proteolysis-targeting chimeras (PROTACs) and molecular-glue degraders (MGDs) would constitute ACH2.0-based AD drugs. Fortunately, we do not have to venture far afield to find two outstanding candidates capable of the targeted degradation of iAβ, they are front and center: BACE1 and BACE2, or, more precisely, intra-iAβ cleaving activities of BACE1 and BACE2. Both enzymes are capable of cleaving at the beta-site of AβPP, as indicated by their designations. In BACE1 cleavage at the β-site is its major activity. In addition, BACE1 also cleaves at the β’-site, ten amino acids downstream from the β-site [43,44] and between amino acid residues 34 and 35 of Aβ, the latter generating an intermediate in the iAβ clearance process [220,221,222,223]. Potential beneficial outcomes of these cleavages were demonstrated in transgenic mouse model and in neuronal cells overexpressing BACE1. This substantially increased both the efficiency and the proportion of cleavages at the β’ and Aβ34/35 sites [220,221,222,223,224,225,226,227].

In BACE2 cleavages within iAβ, or within the Aβ segment of AβPP and C99, are the major activity of the enzyme. These cleavages, occurring physiologically, take place at positions Aβ19 and Aβ20 [228,229]. The therapeutic potential of these cleavages is underscored by the observation that when BACE2 activity was suppressed in transgenic models, the production of Aβ as well as the extent of its extracellular deposition significantly increased. The notion that the increased activity of BACE2 confers benefits and its reduced activity is detrimental is supported by the observations made in human pluripotent cells-derived brain organoids, where increased activity of BACE2 protected from the iAβ-mediated toxicity and neuronal death whereas the reduction in BACE2 activity correlated with the increased neurodegeneration [230]. Thus, the activation of intra-iAβ cleaving potential of BACE1 and, especially, BACE2 (because intra-iAβ cleavages are its major activity) is, apparently, a plausible therapeutic strategy. Moreover, simultaneous activation of both BACE1 and BACE2 can yield synergetic benefits: these enzymes not only target different positions within iAβ and the Aβ segment of AβPP and C99 but are also localized in different intracellular compartments [231].

## 45. Validation (10): Augmentation of the Efficiency of the BACE1-Mediated Intra-iAβ Cleavage Protects from AD and AACD Whereas Suppression of the BACE2-Mediated Intra-iAβ Cleavage Causes AD

How to test the proposition that the activation of BACE1 and BACE2 is a valid therapeutic strategy? The answer is simple: develop suitable and efficient activators of both enzymes (more precisely, of their intra-iAβ cleaving activities) and test them in human clinical trials. If the proposition is valid, activated BACE enzymes would protect from AD and AACD. In a decisive complementary trial, unfeasible for the ethical reasons, BACE intra-iAβ cleaving activities would be suppressed. If the proposition were valid, this would cause AD. Both trials, in fact, were already carried out by nature (which is, apparently, not concerned by the ethical considerations), in the form of Aβ mutations; the results of these “natural” trials soundly validate the proposition.

One of the mutations referred to above is the Icelandic Aβ mutation. It is in the proximity of the β’-site and, consequently, affects, more specifically, increases the efficiency of cleavage at this position (in iAβ as well as in Aβ segments of AβPP and C99) by BACE1. This increase has a two-pronged effect. First, it reduces (by cleavages within Aβ segments of AβPP and C99) the rate of production of Aβ. This decreases the rate of secretion of Aβ, reduces the extracellular Aβ pool, lowers the rate of the uptake of extracellular Aβ, and thus diminishes the rate of influx of AβPP-derived iAβ. Second, it increases, by the cleavage within iAβ, the rate of its efflux. Consequently, with the decreased influx and increased efflux, the rate of accumulation of AβPP-derived iAβ decreases and the timing of the T1 crossing increases. The extent of the Icelandic mutation-mediated increase in the efficiency of cleavage at the β’-site is relatively small, only 30%. It is sufficient, however, to confer on its carriers protection from both AD and AACD [43,44].

Another mutation under discussion in the present section is the Flemish Aβ mutation. It substitutes the amino acid residue at the position Aβ21, a position in the contiguous proximity to BACE2 intra-iAβ cleavage sites (Aβ19 and Aβ29). This affects, more specifically, decreases to below the physiological the efficiency of cleavages at the positions Aβ19 and Aβ20 in iAβ as well as in Aβ segments of AβPP and C99. Since fewer cleavages occur within Aβ segments of AβPP and C99, the rates of production and secretion of Aβ increase. This results in an increase in the extracellular Aβ pool and elevated rates of its uptake, leading to an influx of AβPP-derived iAβ accordingly. Concurrently, the reduction in the rate of cleavages within iAβ lowers the rate of its efflux. With the increased influx and decreased efflux, the rate of accumulation of AβPP-derived iAβ increases. The crossing of the T1 threshold and, consequently, AD would occur sooner than in the wild type. Indeed, in the carriers of the Flemish mutation the onset of AD begins as early as thirty-five years of age [232,233,234,235,236,237,238].

The above results leave little if any doubt that effective activators of intra-iAβ cleaving capabilities of BACE1 and BACE2 would constitute efficient AD and AACD drugs. The following three sections analyze their anticipated effects in the prevention and treatment of conventional Alzheimer’s disease.

## 46. Activators of BACE1 and/or BACE2 in the Prevention of Conventional AD and the Prevention and Treatment of AACD: A Single Transient Therapy Can Potentially Protect for Life

As discussed in the preceding sections, activators of the intra-iAβ cleaving capabilities of BACE1 and BACE2 (referred to henceforth as “activators of BACE1 and BACE2” and their effect designated as “activation of BACE1 and BACE2”) are potential AD and AACD drugs. The present section considers their anticipated effect in the prevention of conventional AD and the prevention and treatment of AACD. In all three scenarios the treatment is administered prior to the crossing of the T1 threshold by AβPP-derived iAβ, i.e., prior to the activation of the AβPP-independent C99/iAβ production pathway. In such a setting, it can be anticipated that activated BACE1 and/or BACE2 would be very effective in depleting iAβ. Indeed, only a 30% increase in the efficiency of cleavage at only the β’-site (the Icelandic mutation) prevents the accumulation of AβPP-derived iAβ to the T1 level. Adequate activators can potentially increase the efficiency of cleavages not only at the β’-site but also at the position Aβ34 (by BACE1) and at the positions Aβ19 and Aβ20 (by BACE2) to the extent much greater than 30%. The required duration of the treatment would be defined by its efficiency. The aim is to deplete iAβ close to its initial baseline. With the anticipated activity of sufficiently activated BACE1 and/or BACE2, the desired depletion can be accomplished in a relatively short time. The treatment, in such a case, would be transient. When the drug is withdrawn, the de novo accumulation of AβPP-derived iAβ, supported only by the AβPP proteolysis, would commence from a low baseline at the pre-treatment rate. The accumulation of AβPP-derived iAβ is a very slow process. It does not reach the T1 threshold within the lifetime of most individuals, and in those who develop non-familial AD, it takes over six decades for the T1 crossing to occur. This implies that if the transient iAβ depletion treatment via the activation of BACE1 and/or BACE2 is administered at midlife, around fifties, and if the depletion is sufficiently deep, iAβ levels would reach neither the T^0^ nor T1 thresholds within the lifetime of the treated individual, and neither AACD nor AD would occur. Thus, a single treatment, timely administered, can potentially protect from both AD and AACD for life.

**Figure 19:**
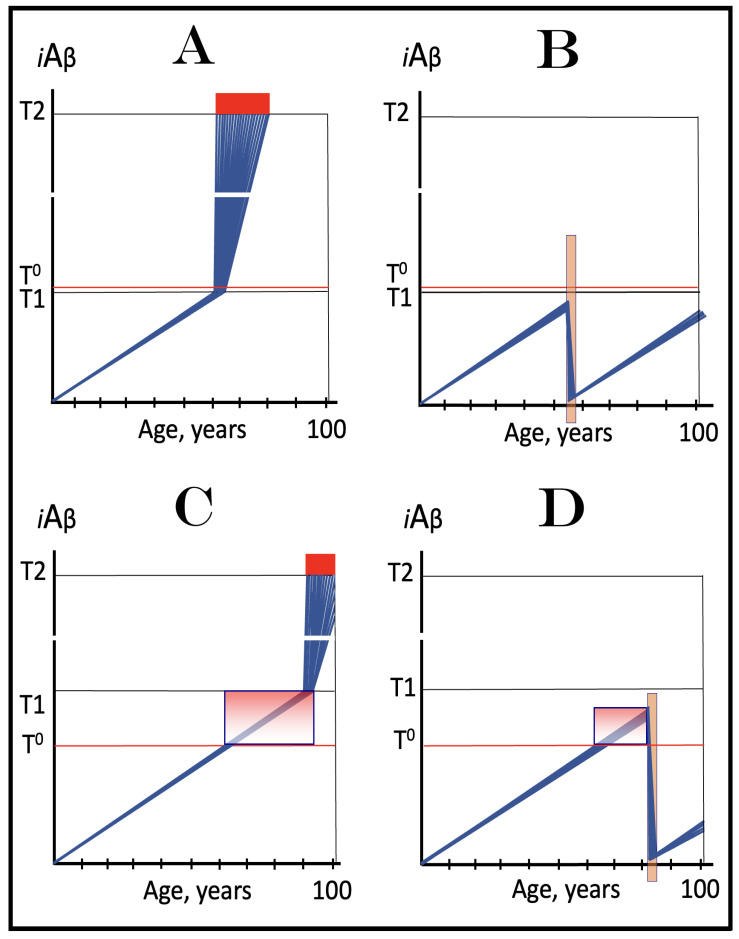
**Activators of BACE1 and/or BACE2 in the prevention of conventional AD and the prevention and treatment of AACD.** *Blue lines*: Intraneuronal Aβ, iAβ. ***T*^0^**: Threshold of cellular concentration of AβPP-derived iAβ triggering the neuronal damage, which manifests as AACD. ***T1***: Threshold of cellular concentration of iAβ triggering the activation of PKR and/or HRI kinases, phosphorylation of eIF2α, the elicitation of the neuronal ISR, the initiation of the AβPP-independent C99 production pathway, and the commencement of AD; its extent is the only variable in the present figure. ***T2***: Cellular concentration of iAβ triggering neuronal death via apoptosis or necroptosis. *Red box*: Apoptotic zone, defined as a range of cellular concentrations of iAβ within which cells neuronal cells committed apoptosis or necroptosis or are dead. *Pink boxes*: The range of cellular concentrations of AβPP-derived iAβ concentrations between the T^0^ and the highest extent reached by AβPP-derived iAβ or the T1 threshold that support the progression of AACD. The condition commences with the crossing of the T^0^ threshold and morphs into AD with the T1 crossing; it can occur only if the extent of the T^0^ threshold is smaller than that of the T1. *Orange boxes*: Duration of the treatment with the BACE1- and/or BAEC2-activating drug. *Panel* (**A**): Dynamics of the accumulation of iAβ in the untreated AD patient. AβPP-derived iAβ accumulates via two physiological mechanisms described in the main text. When it crosses the T1 threshold PKR and/or HRI kinases are activated, eIF2α phosphorylated, the neuronal ISR elicited, the AβPP-independent C99 production pathway initiated, and conventional AD commences. The T^0^ threshold is above the T1; there is no AACD. *Panel* (**B**): Activators of BACE1 and/or BACE2 are administered transiently prior to the T1 crossing. As a result, AβPP-derived iAβ is substantially depleted. Following the withdrawal of the drug, the de novo accumulation commences from low baseline and proceeds at the pre-treatment rate. Neither the T1 threshold would be crossed, nor AD would occur within the lifetime of the treated individual. *Panel* (**C**): Dynamics of the accumulation of iAβ in the untreated AACD/AD patient. When AβPP-derived iAβ reaches the T^0^ threshold, AACD commences and persists until the T1 crossing, the activation of the AβPP-independent C99/iAβ production pathway, and the commencement of AD. At this point AACD morphs into AD. *Panel* (**D**): Activators of BACE1 and/or BACE2 are administered transiently following the T^0^ crossing and the commencement of AACD but prior to the T1 crossing. As a result, iAβ is substantially depleted well below the T^0^ threshold. At this point AACD is cured. The accumulation of AβPP-derived iAβ starts de novo from low baseline and proceeds at the pre-treatment rate. It would not reach the T^0^ threshold within the lifetime of the treated individual and neither AACD would recur nor AD would occur.

The above strategy is illustrated in Figure 19. Panel A of Figure 19 depicts the accumulation of iAβ and the progression of AD in the untreated individual. In the first stage the accumulation of iAβ is supported solely by the AβPP proteolysis. The extent of the T^0^ threshold is greater than that of the T1 threshold; no AACD occurs in this scenario. When the T1 threshold is crossed, PKR and/or HRI are activated, eIF2α phosphorylated, the neuronal integrated stress response elicited and the AβPP-independent production of C99/iAβ initiated. Levels of the latter rapidly increase and reach the AD pathology-causing range. When they reach the T2 threshold, neuronal loss ensues. Panel B of Figure 19 shows the outcome of the transient treatment with activators of BACE1 and/or BACE2. The treatment is administered prior to the T1 crossing, and AβPP-derived iAβ is substantially depleted. Following the withdrawal of the drug, the de novo accumulation commences from low baseline and proceeds at the pre-treatment rate. Neither the T1 threshold would be crossed, nor AD would occur within the lifetime of the treated individual.

Panel C of Figure 19 presents the dynamics of accumulation of iAβ and the progression of AACD and AD in a scenario where the extent of the T^0^ threshold is smaller than that of the T1 threshold. When AβPP-derived iAβ reaches the T^0^ threshold, AACD commences and persists until the T1 crossing and the activation of the AβPP-independent C99/iAβ production pathway. At this point AACD morphs into AD. If activators of BACE1 and/or BACE2 were administered transiently prior to the T^0^ crossing, and if the depletion of iAβ were deep enough, iAβ, accumulating de novo following the treatment, would not reach the T^0^ threshold within the remaining lifetime of the treated individual and no AACD (and neither AD) would occur. If, as shown in Panel D of Figure 19, the treatment was administered post-T^0^ crossing, but prior to the T1 crossing, iAβ would be substantially depleted well below the T^0^ threshold. At this point AACD would be cured. The accumulation of AβPP-derived iAβ would start de novo from a low baseline and proceed at the pre-treatment rate. It would not reach the T^0^ threshold within the lifetime of the treated individual and neither AACD would recur nor AD would occur.

## 47. Problem with the Utilization of Activators of BACE1 and/or BACE2 in the Treatment of Conventional AD: With the AβPP-Independent C99 Generation Pathway Operational the Rate of Degradation of iAβ Is Unlikely to Match or Exceed That of Its Influx

The depletion of iAβ to a low baseline would be as effective therapeutically in the treatment of symptomatic conventional AD as in the prevention of the disease provided it could be accomplished. However, the problem with the activators of BACE1 and/or BACE2 is that, on their own, they are, apparently, incapable of such a feat. The source of this problem is the operation of the AβPP-independent C99/iAβ production pathway. As described above, the efficiency of this pathway is apparently comparable to that of a massive gene amplification, with the rate of iAβ generation potentially orders of magnitude greater than the rate of iAβ production in the AβPP proteolytic pathway. In such a case, it is inconceivable that the degradation of iAβ by activated BACE1 and/or BACE2, however efficient, could match the influx of iAβ generated independently of AβPP. In such a case, even if employed long-term, the activation of BACE1 and/or BACE2 would only reduce the rate of iAβ accumulation and, at best, slow down the progression of AD.

**Figure 20:**
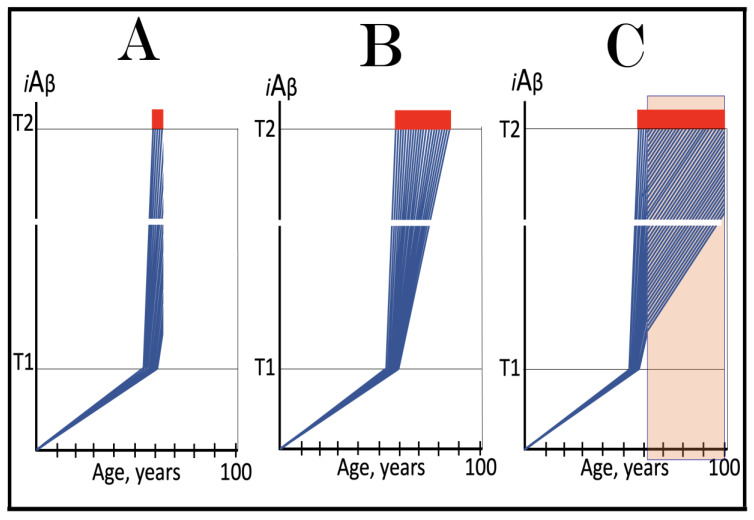
**With the AβPP-independent C99/iAβ production pathway operational, the rate of degradation of iAβ by activated BACE1 and/or BACE2 cannot match or exceed that of its influx**. *Blue lines*: Intraneuronal Aβ, iAβ. ***T1***: Threshold of cellular concentration of iAβ triggering the activation of PKR and/or HRI kinases, phosphorylation of eIF2α, the elicitation of the neuronal ISR, the initiation of the AβPP-independent C99 production pathway, and the commencement of AD. ***T2***: Cellular concentration of iAβ triggering neuronal death via apoptosis or necroptosis. *Red box*: Apoptotic zone, defined as a range of cellular concentrations of iAβ within which cells neuronal cells committed apoptosis or necroptosis or are dead. *Orange box*: Duration of the treatment with the BACE1- and/or BACE2-activating drug. *Panel* (**A**): The initial state of the levels of AβPP-derived iAβ in the neurons of the AD patient. AβPP-derived iAβ has crossed the T1 threshold in all affected neurons, the neuronal ISR has been elicited, the AβPP-independent C99/iAβ production pathway activated, the T2 threshold crossed in a neuronal fraction, and AD symptoms have manifested. *Panel* (**B**): The disease progresses and reaches its end stage. *Panel* (**C**): The evolution of the initial state in the individual treated with the BACE1- and/or BACE2-activating drug. The rate of degradation of iAβ via its internal cleavages increases but it cannot match the rate of the influx of iAβ produced independently of AβPP. The rate of the accumulation of iAβ decreases and the progression of AD slows down for the duration of the treatment, but the disease persists.

The anticipated outcome of the utilization of activators of BACE1 and BACE2 in the treatment of conventional AD in the version of the ACH2.0 under discussion is illustrated in Figure 20. Panel A of Figure 20 shows the initial state of the levels of iAβ in the affected neurons of the AD patient at the commencement of the treatment. AβPP-derived iAβ has crossed the T1 threshold in all affected neurons, the neuronal integrated stress response has been elicited and the AβPP-independent C99/iAβ production pathway activated; a fraction of the neurons has crossed the T2 threshold and AD symptoms have manifested. Panel B depicts the evolution of the initial state in the untreated AD patient. The accumulation of iAβ, mainly in the AβPP-independent C99/iAβ production pathway, continues and AD pathology progresses. When a sufficient neuronal fraction reaches the T2 threshold and commits apoptosis, the disease reaches its end stage. Panel C presents the evolution of the initial state in the presence of the activators of BACE1 and/or BACE2. The rate of degradation of iAβ via its internal cleavages increases but it cannot match, not to say exceed, the rate of the influx of iAβ produced independently of AβPP. The rate of the accumulation of iAβ decreases and the progression of AD slows down for the duration of the treatment, but the disease persists. This is better than nothing but not good enough. Fortunately, the solution to this problem exists and is presented in the following section.

## 48. Solution: Cessation of the Influx of iAβ Produced Independently of AβPP by the Transient Inhibition of the Neuronal ISR in the Presence of Activators of BACE1 and/or BACE2

As discussed in the preceding section, the problem with the employment of the activators of BACE1 and BACE2 in the treatment of conventional AD is that the rate of the influx of iAβ produced independently of AβPP is too great to be matched by the rate of degradation of iAβ by activated BACE1 and/or BACE2. The solution is simple: suppress the influx of iAβ produced independently of AβPP for the duration of the depletion treatment or even better, stop it altogether. If this was accomplished, the influx of iAβ would be supported only by the AβPP proteolysis, and the rate of its depletion by activated BACE1 and/or BACE2 would be comparable to that anticipated in the prevention of the disease. This is doable and can be accomplished by transient inhibition of the neuronal integrated stress response executed concurrently with the treatment by the activators of BACE1 and/or BACE2.

It was argued above (see Section 43) that the long-term systemic inhibition of the integrated stress response is, apparently, not feasible. This is correct. But in the application under discussion the transient suppression of the ISR would suffice (and, apparently, would not be deleterious; indeed, transient inhibition of ISR did not cause adverse effects in mouse models [119,120,121,122,123,124,125,126,127]). The reversal of the neuronal ISR would deprive the AβPP-independent C99/iAβ generation pathway of its essential components; its operation would stop, and the influx of its product would cease. The situation in such a case would be similar to that encountered in the prevention of AD: the influx of iAβ would be supported solely by the AβPP proteolysis. Activators of BACE1 and/or BACE2, administered concurrently with ISR inhibitors, would rapidly and efficiently deplete iAβ. The administration of ISR inhibitors in this application is transient. Their presence is essential only for the depletion of iAβ to levels below the T1 thresholds. This would ensure that, when they are withdrawn, the neuronal ISR would not be re-elicited and the AβPP-independent C99/iAβ production pathway reactivated. There are no obvious limitations for the duration of the presence of activators of BACE1 and BACE2, but they also are needed only transiently, until the levels of iAβ are reduced to sufficiently low extents. Following the withdrawal of BACE1 and/or BACE2 activators, the accumulation of AβPP-derived iAβ would resume. However, its levels would not reach the T1 thresholds and AD would not recur.

**Figure 21:**
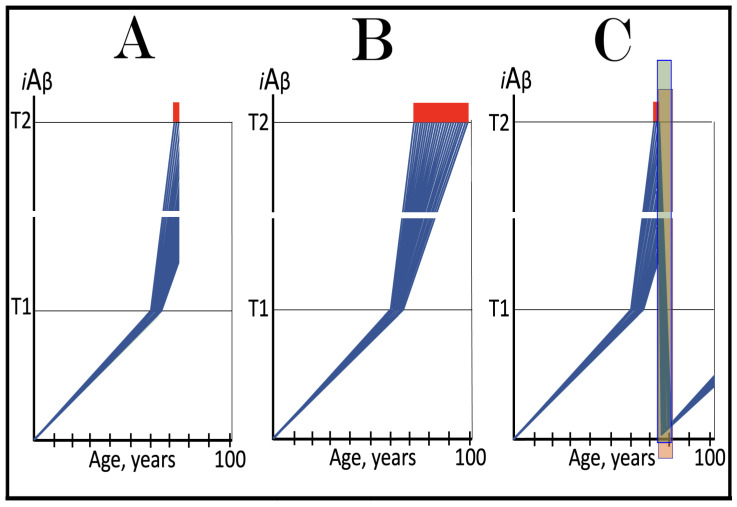
**Concurrent transient BACE1 and/or BACE2 activation and ISR inhibition in the treatment of conventional AD**. *Blue lines*: Intraneuronal Aβ, iAβ. ***T1***: Threshold of cellular concentration of iAβ triggering the activation of PKR and/or HRI kinases, phosphorylation of eIF2α, the elicitation of the neuronal ISR, the initiation of the AβPP-independent C99 production pathway, and the commencement of AD. ***T2***: Cellular concentration of iAβ triggering neuronal death via apoptosis or necroptosis. *Red box*: Apoptotic zone, defined as a range of cellular concentrations of iAβ within which cells neuronal cells committed apoptosis or necroptosis or are dead. *Green box*: Duration of the treatment with the ISR-inhibiting drug. *Orange box*: Duration of the treatment with the BACE1- and/or BACE2-activating drug. *Panel* (**A**): The initial state of the levels of AβPP-derived iAβ in the neurons of the AD patient. AβPP-derived iAβ has crossed the T1 threshold in all affected neurons, the neuronal ISR has been elicited, the AβPP-independent C99/iAβ production pathway activated, the T2 threshold crossed in a neuronal fraction, and AD symptoms have manifested. *Panel* (**B**): The disease progresses and reaches its end stage. *Panel* (**C**): The evolution of the initial state in the treated patient. The ISR-inhibiting drug is administered transiently and concurrently with the activator of BACE1 and/or BACE2. Suppression of the neuronal ISR disables the operation of the AβPP-independent C99/iAβ production pathway and the influx of its product stops. This allows activated BACE1 and/or BACE2 to efficiently deplete iAβ. Following the withdrawal of both ISR inhibitors and BACE activators, the AβPP-independent C99/iAβ production pathway remains inoperative, and the progression of AD ceases. De novo accumulation of iAβ commences from a low baseline supported solely by the AβPP proteolysis. It would not reach the T1 threshold, the AβPP-independent C99/iAβ production pathway would not be reactivated, and the disease would not recur within the remaining lifetime of the treated AD patient.

The strategy under discussion is illustrated in Figure 21. Panels A and B of Figure 21 are similar, in fact, identical to the corresponding panels of Figure 20. Panel C of Figure 21 is drastically different from its counterpart in Figure 20. It depicts a composite therapy. Inhibitor(s) of the ISR are administered transiently and concurrently with activator(s) of BACE1 and/or BACE2. Suppression of the neuronal ISR disables the operation of the AβPP-independent C99/iAβ production pathway and the influx of its product stops. This allows activated BACE1 and/or BACE2 to efficiently deplete iAβ. With the AβPP-independent C99/iAβ production pathway inoperative, the progression of AD ceases. Following the withdrawal of both ISR inhibitors and BACE activators, de novo accumulation of iAβ commences from a low baseline; it is supported solely by the AβPP proteolysis. It would not reach the T1 threshold, the AβPP-independent C99/iAβ production pathway would not be reactivated, and the disease would not recur within the remaining lifetime of the treated AD patient.

## 49. Progression of AD Is Defined by the Rate of the Production of iAβ in the AβPP-Independent Pathway and by the Extent of the T2 Threshold: Sequential Occurrence of AD Pathology in Defined Brain Compartments

AβPP-derived iAβ reaches and crosses the T1 threshold in the affected neurons within a narrow temporal window [1,4], and this crossing denotes the commencement of conventional AD. Therefore, the rate of accumulation of AβPP-derived iAβ and the extent of the T1 threshold decide the occurrence of conventional AD (or the lack thereof) and determine its timing. Once the disase occurs, its progression is also defined by two major parameters. One is the dynamics of accumulation of iAβ produced independently of AβPP (in this variant of the ACH2.0), which, in turn, is defined by the efficiency of the AβPP-independent C99/iAβ generation pathway. Another is the extent of the T2 threshold. The greater the rate of accumulation of iAβ produced independently of AβPP and the smaller the extent of the T2 threshold, the faster would be the progression of the disease and the shorter its duration. Conversely, the smaller the rate of accumulation of iAβ produced independently of AβPP and the greater the extent of the T2 threshold, the slower the progression of AD and the longer its duration.

Variations in these two parameters, the rate of accumulation of iAβ produced independently of AβPP and the extent of the T2 threshold, can explain one of the principal features of Alzheimer’s disease, namely the sequential occurrence of AD pathology in defined compartments of the brain [239,240,241]. Indeed, it appears that the AD-associated neurodegeneration commences within the defined layer of the entorhinal cortex. This is followed, sequentially, by its occurrence in the hippocampus, temporal cortex, frontoparietal cortex, and subcortical nuclei [239]. The sequential development of AD pathology is observed even within defined areas of a specific brain region. Thus, in the hippocampus, AD pathology spreads sequentially from the CA1 area to CA2, CA3, and, finally, DG areas [240]. One explanation of such a temporal order is that, at the given rate of accumulation of iAβ produced independently of AβPP, the extent of the T2 threshold differs in different brain compartments and even sub-compartments. The greater it is, the later the neurodegeneration manifests. Another explanation is that, at the given extent of the T2 threshold, the rate of accumulation of iAβ produced independently of AβPP varies in distinct brain compartments and sub-compartments. The greater it is, the sooner the neurodegeneration manifests. Finally, of course, these two variables can modulate concurrently.

**Figure 22:**
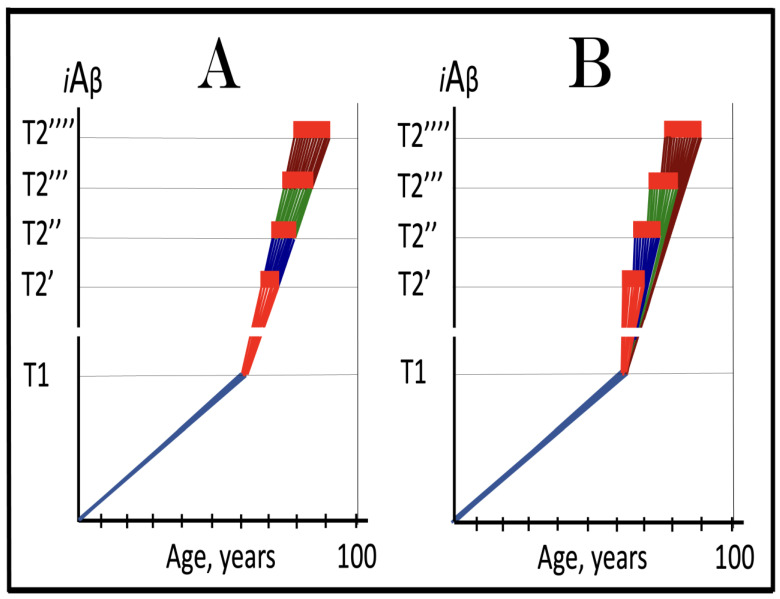
**Sequential occurrence of AD pathology in the defined brain compartments as the consequence of the variability in the rates of AβPP-independent iAβ accumulation and the extents of the T2 threshold**. *Blue lines*: Intraneuronal Aβ, iAβ. ***T1***: Threshold of cellular concentration of iAβ triggering the activation of PKR and/or HRI kinases, phosphorylation of eIF2α, the elicitation of the neuronal ISR, the initiation of the AβPP-independent C99 production pathway, and the commencement of AD. ***T2***: Cellular concentration of iAβ triggering neuronal death via apoptosis or necroptosis. ***T2′*, *T2’’*, *T2’’’*, *T’’’’***: Variable T2 thresholds in the defined regions of the brain. *Red*, *blue*, *green*, *and brown lines above the T1 threshold*: iAβ accumulation in the AD-affected neurons in various regions of the brain. *Red box*: Apoptotic zone, defined as a range of cellular concentrations of iAβ within which cells neuronal cells committed apoptosis or necroptosis or are dead. *Panel* (**A**): Effects of the variable extent of the T2 threshold at the background of the given rate of accumulation of iAβ produced independently of AβPP on the sequential occurrence of AD pathology in the defined regions of the brain. The greater the former, the later the neurodegeneration manifests. *Panel* (**B**): the combined effect of modulation of both the extent of the T1 threshold and the rate of accumulation of iAβ produced independently of AβPP on the sequential occurrence of AD pathology in the defined brain compartment.

The variability discussed above is illustrated in Figure 22 where different brain compartments are schematically denoted by different colors. Panel A of Figure 22 depicts the effects of the variable extent of the T2 threshold at the background of the given rate of accumulation of iAβ produced independently of AβPP. The greater the former, the later the neurodegeneration manifests. Panel B of Figure 22 shows the combined effect of modulation of both the extent of the T1 threshold and the rate of accumulation of iAβ produced independently of AβPP.

Importantly, the above interpretation of the sequential occurrence of AD pathology in the defined compartments of the brain has consequential therapeutic implications and underscores the significance of the earliest possible intervention in AD. Indeed, if the composite therapy described in Section 48 above is successful, the progression of the disease in the selectively affected brain compartments will cease and not recur. The depletion of iAβ to low baselines would take place also in brain compartments not yet affected (or affected insignificantly) by the disease. Therefore, in the treated patients, these compartments would be protected by the treatment and would stay AD pathology-free for the remaining lifespan.

## 50. The ACH2.0, Version Two: Alzheimer’s Is a Disease of the Neuronal ISR; Unconventional AD Is Triggered by Stressors Distinct from AβPP-Derived iAβ and Driven by iAβ Generated Independently of AβPP

The initial version of the ACH2.0, discussed above, defines conventional AD. In this version, by far the most important player is iAβ generated differentially in two distinct pathways. iAβ derived in the AβPP proteolytic pathway and accumulated over the T1 threshold triggers the activation of the AβPP-independent C99/iAβ generation pathway, and it is iAβ produced in the latter, which drives the disease. The importance of the neuron-specific component, iAβ, produced in two distinct ways, in the neuron-specific disease, AD, overshadowed the role of a non-neuron-specific intermediate component in transition from AβPP-derived iAβ to iAβ generated independently of AβPP, namely the integrated stress response. But it is the neuronal integrated stress response, which supplies essential constituents of the AβPP-independent production of C99/iAβ. Moreover, it not just “switches on” the process: the neuronal ISR maintains the activity of the AβPP-independent C99/iAβ production pathway, thus enabling it; if the ISR conditions are reversed, the operation of the AβPP-independent C99/iAβ generation pathway ceases. AD was defined, in terms of the ACH, as the disease of Aβ, and the notion remained ingrained and, therefore, hard to overcome. But it is, in fact, the neuronal ISR, which triggers the disease by delivering its essential components, and it is not only necessary but also sufficient for the disease to occur [8,10]. AD is, therefore, not a disease of Aβ but a disorder of the neuronal integrated stress response: elicit, IN ANY WAY, the sustained neuronal ISR and Alzheimer’s disease will ensue.

It follows that the sustained elicitation of the neuronal ISR, even by stressors other than AβPP-derived iAβ and even at the levels of AβPP-derived iAβ far below the T1 threshold, would trigger the activation of the AβPP-independent C99/iAβ production pathway and, consequently, the commencement of AD pathology. The category of Alzheimer’s disease caused by sustained neuronal ISR elicited by stressors distinct from AβPP-derived iAβ has been designated “unconventional AD” [8,9,10] to distinguish it from ”conventional AD”, which is defined as the disease caused by the neuronal ISR elicited solely by AβPP-derived iAβ. This separation into two categories seems ostentatiously asymmetrical: the only difference appears to be one specific ISR-eliciting stressor (AβPP-derived iAβ) versus a multitude of potential ISR-eliciting stressors. This, however, is not the case. First, conventional AD is the prevalent category of the disease, accounting for about 60% of all cases of dementia. Second, all unconventional AD cases can be grouped together (and separately from conventional cases) on the basis of the following criteria. Whereas in conventional AD the AβPP-independent C99/iAβ production pathway is always self-sustainable from the instance of its activation, in unconventional AD it takes some time for it to become self-sustainable: if the initial ISR-eliciting unconventional stressor were withdrawn too early, the activity of the AβPP-independent C99/iAβ generation pathway would be only transient. This is why, to trigger AD, the unconventionally elicited neuronal ISR state should be sufficiently prolonged in order to ensure that the AβPP-independent C99/iAβ production pathway becomes self-sustainable. This aspect of unconventional AD is further discussed in Section 52 below.

Thus, beside AβPP-derived iAβ (designated “conventional stressor”) that triggers conventional AD, any “unconventional” (i.e., distinct from AβPP-derived iAβ) stressor capable of sustainably activating one or more of the four kinases of eIF2α can potentially initiate unconventional AD. Such stressors are abundant and, therefore, so are their sources, i.e., conditions potentially resulting in unconventional AD. They include brain trauma (traumatic brain injury, TBI) [242,243,244] and chronic traumatic encephalopathy, CTE [245]. These conditions can stem from viral as well as from bacterial infections [246,247,248,249,250,251,252,253,254]. Thus, an infection of viral encephalitis elevates more than thirtyfold the probability of developing unconventional AD [246,247,248,249,250] whereas various bacterial infections make the incidence of AD more than tenfold likely [251,252,253,254]. Notable conditions that have strong association with AD encompass neuroinflammation, and chronic systemic and even localized inflammations including, for example, rheumatoid- and osteoarthritis [255,256,257,258,259,260,261,262,263,264,265,266]. Cases of dementia related to the above conditions are often categorized as AD-like dementia, ADLD or AD-related dementia, ADRD. In the framework of the ACH2.0 version under discussion, the majority, if not all, cases of ADRD and ADLD probably belong to the category of unconventional AD.

Recently, it has been shown that distincly different sources of unconventional stressors can interact with and even stimulate one another. Thus, herpes simplex virus in general and HSV-1 in particular were shown in multiple studies to be associated with Alzheimer’s disease [267,268,269,270]. HSV-1 is known to be neurotropic and capable of establishing dormant state in nerve tissues, e.g., in the sensory root ganglia of the trigeminal nerve [268]. In a recent study employing human brain organoid tissue model harboring dormant HSV-1 it was demonstrated that repetitive mechanical injury (i.e., a simulation of CTE) activates dormant HSV-1 and induces phenotypes associated with AD [271].

**Figure 23:**
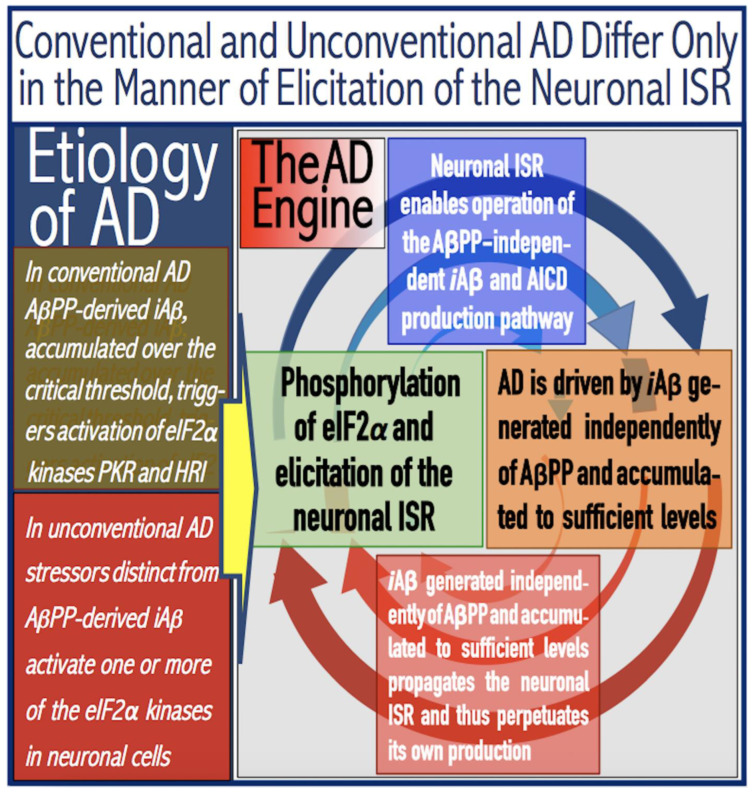
**Alzheimer’s is a multiform disease of the neuronal ISR: Conventional AD is triggered by AβPP-derived iAβ whereas neuronal ISR-eliciting stressors distinct from AβPP-derived iAβ initiate unconventional AD**. *eIF2α*: eukaryotic translation initiation factor 2*α*. *PKR and HRI*: kinases that phosphorylate eIF2α at its Ser51 residue. *PACT*: PKR activator. *TNFα*: tumor necrosis factor *α*. *OMA1*: mitochondrial protease activated during mitochondrial dysfunction. *DELE1*: substrate of OMA1 protease. Cleavage of DELE1 leads to the activation of HRI. Phosphorylation of eIF2α elicits the integrated stress response, which, in turn, enables the generation of components essential for the activity of the AβPP-independent pathway of C99 and iAβ production. iAβ: intraneuronal Aβ. The accumulation of iAβ produced in both the AβPP proteolytic and AβPP-independent pathways is accompanied by a proportional accumulation of AICD, AβPP Intracellular Domain. AICD was shown to be capable of interfering with numerous processes involved in AD but its contribution to the disease remains to be elucidated. AβPP-derived iAβ accumulates physiologically via the importation of extracellular Aβ and the retention of iAβ produced by gamma-cleavages on intracellular membranes in an exceedingly slow process. In most individuals it does not reach the PKR- and/or HRI-activating, the neuronal ISR-eliciting threshold within their lifetimes and no conventional AD occurs. When this threshold is crossed, the neuronal ISR is elicited and the AβPP-independent C99 (and, subsequently, iAβ) production pathway activated, and conventional AD ensues. In unconventional AD stressors that are distinct from AβPP-derived iAβ and capable of activating one or more of eIF2α kinases trigger the elicitation of the neuronal ISR. In both conventional and unconventional forms of AD the iAβ product of the AβPP-independent pathway of C99 and iAβ production drives AD pathology. It also propagates the neuronal ISR and thus perpetuates its own production in the AβPP-independent pathway. Thus, conventional and unconventional AD differ only in the manner of the elicitation of the neuronal integrated stress response.

Specific signaling pathways connecting the above discussed conditions with the activation of one or more of eIF2α kinases remain to be elucidated. However, there are apparently at least two features that are common to all conditions listed above. One such feature is compromised blood–brain barrier, BBB [272]. BBB defects, in turn, allow the neuronal penetration of stressors potentially capable of eliciting the unconventional neuronal ISR. Another common feature is the suppression of the cerebral blood flow, CBE [273,274,275,276,277,278,279]. There are, potentially, numerous ways for CBE to trigger the elicitation of the neuronal ISR but one stands out because of its involvement in conventional AD. CBE is known to trigger mitochondrial dysfunction in neuronal cells [280,281]. This is sufficient to activate, via the OMA1 to DELE1 to HRI signaling pathway, the HRI [185,186], and thus to trigger the elicitation of the neuronal ISR and, consequently, unconventional AD [8]. The bottom line of the above considerations is that conventional and unconventional forms of AD differ only in the manner of elicitation of the neuronal ISR. Once the AβPP-independent C99/iAβ production pathway, activated by the neuronal ISR, becomes self-sustainable, conventional and unconventional forms of AD are mechanistically indistinguishable. This relationship between two forms of the disease is illustrated in Figure 23.

## 51. Validation (11): Sustained Elicitation of the ISR by Unconventional Stressors Should Activate the AβPP-Independent C99 Production Pathway and Trigger Cellular AD Pathology, Including NFT Formation, in Human Neuronal Cells

The concept that sustained elicitation of the neuronal integrated stress response is necessary and sufficient to cause AD, more precisely unconventional AD, can be validated with human neuronal cells. Above, in Section 20, we argued that, as experiments by Choi and co-workers demonstrated [128], human neuronal cells constitute an adequate model of AD. They possess functional machinery underlying the operation of the AβPP-independent C99/iAβ production pathway. In the approach described in [128] this pathway is activated by the neuronal ISR elicited by exogenously expressed human iAβ accumulated over the T1 threshold. Its operation drives cellular AD pathology, which manifests in the formation of neurofibrillary tangles [128]. If sustained neuronal ISR is all it takes (i.e., is necessary and sufficient) to activate the AβPP-independent C99/iAβ generation pathway, the same outcome can be attained via the utilization of unconventional ISR-eliciting stressors (i.e., stressors distinct from iAβ) such as, for example, those causing mitochondrial dysfunction (and, consequently activating, via the OMA1 to DELE1 to HRI signaling pathway, the HRI kinase). If the concept of unconventional AD were valid, cellular AD pathology would develop and manifest as NFTs. Another anticipated reporter is N-terminal Met-C99 (C100); it would, as discussed in Section 23 above, report the activity of the AβPP-independent C99/iAβ production pathway. As a control, several unrelated stressors could be employed; this would rule out that the outcome is a side effect of a stressor rather than of the neuronal ISR. The same confirmation can be obtained by performing the experiment in the presence of an ISR inhibitor; this should abrogate the outcome (i.e., the occurrence of NFTs and of C100). Another control option is to perform the same experiment in mouse neuronal cells. As discussed above (Section 17), the AβPP-independent C99/iAβ production pathway is inoperative in mice. Therefore, neither NFTs nor C100 should be observed in mouse neuronal cells under the sustained neuronal ISR conditions. In Section 21 above we described the utilization of human neuronal cells as a model system for conventional AD. Positive outcomes of the experiment proposed in the present section would not only validate the concept formulated above but also establish human neuronal cells as an adequate model system for unconventional AD.

## 52. Unconventionally Activated AβPP-Independent C99/iAβ Production Pathway Becomes Self-Sustainable Only After a Lag Period

Two principal features differentiate unconventional AD from its conventional counterpart. One such feature was discussed above: in unconventional AD the elicitation of the neuronal ISR is triggered not by AβPP-derived iAβ (as in conventional AD) but rather by a variety of stressors that are explicitly not iAβ. Another distinguishing feature is that whereas in conventional AD the activation of the AβPP-independent C99/iAβ production pathway equates with the commencement of the disease, the unconventional activation, in fact, even multiple unconventional activations of this pathway could be transient and do not necessarily result in AD. This is because, in contrast to its conventionally activated counterpart, the unconventionally activated AβPP-independent C99/iAβ production pathway is initially not self-sustainable.

In conventional AD, at the instance of the activation of the AβPP-independent C99/iAβ production pathway levels of AβPP-derived iAβ have crossed the ISR-eliciting T1 threshold rendering the pathway self-sustainable. Indeed, iAβ produced independently of AβPP significantly elevates the total iAβ levels thus ensuring that it remains well over-T1. This maintains the activity of PKR and/or HRI kinase, propagates the neuronal ISR conditions and thus sustains the activity of the AβPP-independent C99/iAβ production pathway. The pathway is, therefore, self-sustainable from the instance of its activation. The withdrawal of the initial ISR-eliciting stressor (in this case AβPP-derived iAβ) would have no effect whatsoever on the operation of the pathway (this is the reason why ACH-based AD drugs are inapplicable in symptomatic AD).

On the other hand, the unconventional activation of the AβPP-independent C99/iAβ production pathway occurs always at the levels of AβPP-derived iAβ below the T1 threshold (otherwise the activation would occur conventionally). iAβ produced independently of AβPP would rapidly accumulate and eventually reach and cross the T1 threshold. At this point the AβPP-independent C99/Aβ production pathway would be rendered self-sustainable, i.e., sustained by its own product, which propagates the neuronal ISR state and thus maintains the activity of the pathway, and unconventional AD would commence. The withdrawal of the initial (unconventional) ISR-eliciting stress at this stage would have no effect on the continuous operation of the AβPP-independent C99/iAβ production pathway. If, however, the initial unconventional stressor would be withdrawn before the T1 threshold is reached, the ISR conditions would be reversed and the activity of the AβPP-independent C99/iAβ production pathway would cease; its operation would be only transient, and the disease would not commence. This aspect of the unconventional activation of the AβPP-independent C99/iAβ production pathway is further discussed in the following sections below.

## 53. Unconventional Activation of the AβPP-Independent C99/iAβ Production Pathway: Effect of the Long-Term Presence of Unconventional ISR-Eliciting Stressors

**Figure 24:**
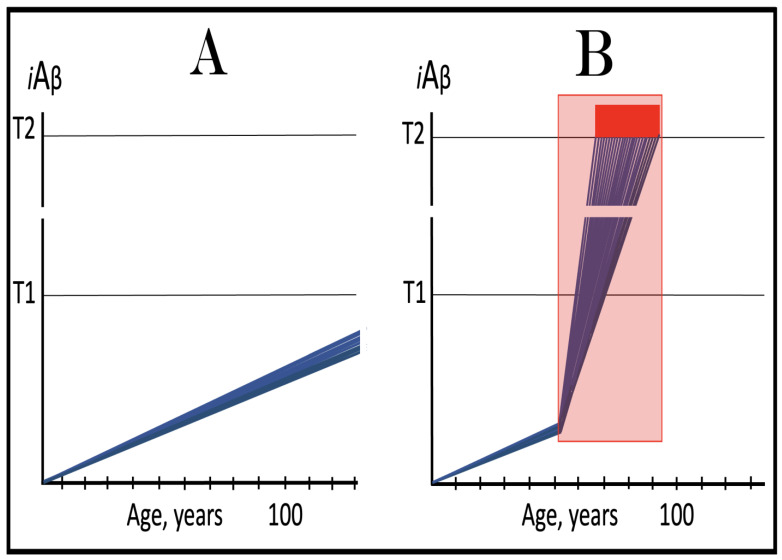
**Unconventional activation of the AβPP-independent C99/iAβ production pathway: Effect of the long-term presence of unconventional ISR-eliciting stressors**. *Blue lines*: Intraneuronal Aβ, iAβ. ***T1***: Threshold of cellular concentration of iAβ triggering the activation of PKR and/or HRI kinases, phosphorylation of eIF2α, the elicitation of the neuronal ISR, the initiation of the AβPP-independent C99 production pathway, and the commencement of AD. ***T2***: Cellular concentration of iAβ triggering neuronal death via apoptosis or necroptosis. *Red box*: Apoptotic zone, defined as a range of cellular concentrations of iAβ within which cells neuronal cells committed apoptosis or necroptosis or are dead. *Pink box*: Duration of the occurrence of the unconventional stressor capable of activating one or more of eIF2α kinases and triggering the elicitation of the neuronal ISR. *Panel* (**A**): Kinetics of the accumulation of AβPP-derived iAβ in the healthy individual. The rate of accumulation of iAβ is such that it does not reach the T1 threshold. eIF2α kinases are not activated, eIF2α is not phosphorylated, the neuronal ISR is not elicited, the AβPP-independent C99/iAβ production pathway is not initiated, and AD does not occur. *Panel* (**B**): Kinetics of the iAβ accumulation in the presence of the unconventional stressor. The unconventional (i.e., distinct from AβPP-derived iAβ) stressor occurs when the levels of AβPP-derived iAβ are well below the T1 threshold and persists for the remaining portion of the lifetime. The neuronal ISR is unconventionally elicited, and the AβPP-independent C99/iAβ production pathway is unconventionally activated. iAβ generated independently of AβPP rapidly accumulates. When its levels cross the T1 threshold, the AβPP-independent C99/iAβ production pathway is rendered self-sustainable and unconventional AD commences.

Figure 24 illustrates the effect of the long-term presence of an unconventional stressor capable of eliciting the integrated stress response in neuronal cells. Panel A of Figure 24 depicts the kinetics of accumulation of AβPP-derived iAβ in the healthy individual. The rate of the accumulation of iAβ is such that it does not reach the T1 threshold. eIF2α kinases are not activated, eIF2α is not phosphorylated, the neuronal ISR is not elicited, the AβPP-independent C99/iAβ production pathway is not initiated, and AD does not occur. In Panel B of Figure 24, the unconventional (i.e., distinct from AβPP-derived iAβ) stressor occurs when the levels of AβPP-derived iAβ are well below the T1 threshold and persists for the remaining portion of the lifetime. The neuronal ISR is unconventionally elicited, and the AβPP-independent C99/iAβ production pathway is unconventionally activated. iAβ generated independently of AβPP rapidly accumulates. When its levels cross the T1 threshold, the AβPP-independent C99/iAβ production pathway is rendered self-sustainable and unconventional AD commences.

## 54. Unconventional Activation of the AβPP-Independent C99/iAβ Production Pathway: Effects of the Transient Presence of Unconventional ISR-Eliciting Stressors

**Figure 25:**
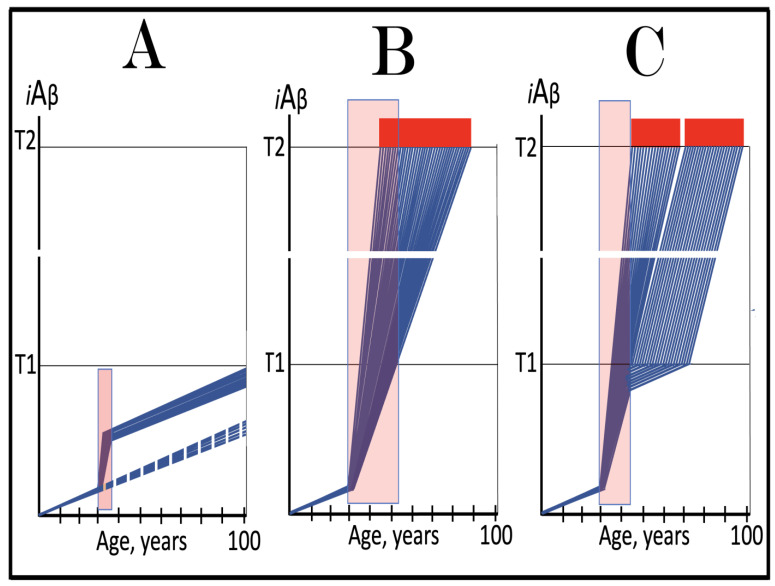
**Unconventional activation of the AβPP-independent C99/iAβ production pathway: effects of the transient presence of unconventional ISR-eliciting stressors**. *Blue lines*: Intraneuronal Aβ, iAβ. ***T1***: Threshold of cellular concentration of iAβ triggering the activation of PKR and/or HRI kinases, phosphorylation of eIF2α, the elicitation of the neuronal ISR, the initiation of the AβPP-independent C99 production pathway, and the commencement of AD. ***T2***: Cellular concentration of iAβ triggering neuronal death via apoptosis or necroptosis. *Red box*: Apoptotic zone, defined as a range of cellular concentrations of iAβ within which cells neuronal cells committed apoptosis or necroptosis or are dead. *Pink boxes*: Duration of the occurrence of the unconventional stressor capable of activating one or more of eIF2α kinases and triggering the elicitation of the neuronal ISR. *Broken lines*: The projected dynamics of the accumulation of AβPP-derived iAβ in the absence of the episodes of unconventional activity of the AβPP-independent C99 production pathway. *Panel* (**A**): The unconventional stressor occurs when AβPP-derived iAβ is well below the T1 threshold and accumulates at such a rate that it would not reach the T1 threshold within the individual’s lifetime (projected accumulation of AβPP-derived iAβ in the absence of the unconventional stressor is shown as broken lines). Following the occurrence of the unconventional stressor, the neuronal ISR is unconventionally elicited and the AβPP-independent C99/iAβ production pathway unconventionally activated. The levels of iAβ produced independently of AβPP rapidly increase. But before they reach the T1 threshold, the unconventional stressor is withdrawn. The ISR conditions are reversed, and the operation of the AβPP-independent C99/iAβ production pathway ceases. The accumulation of AβPP-derived iAβ resumes from an elevated baseline but proceeds at a low rate, supported only by the AβPP proteolysis. It does not reach the T1 threshold within the lifetime of the individual and no AD occurs. *Panel* (**B**): The unconventional stressor persists for a considerable duration. The neuronal ISR is unconventionally elicited, the AβPP-independent C99/iAβ production pathway is unconventionally activated, and iAβ produced independently of AβPP rapidly accumulates. When the unconventional stressor is withdrawn, iAβ has crossed the T1 threshold in all affected neurons. The AβPP-independent C99/iAβ production pathway has attained self-sustainability and the withdrawal of the initial unconventional stressor has no effect whatsoever on its continuous operation. *Panel* (**C**): The unconventional stressor is withdrawn when iAβ produced independently of AβPP has crossed the T1 threshold in only a fraction of affected neurons. In these neurons the AβPP-independent C99/iAβ production pathway has attained self-sustainability and its continuous operation is not affected by the withdrawal of the initial unconventional stressor. In the neurons that have not yet crossed the T1 threshold, the withdrawal of the unconventional stressor terminates the operation of the AβPP-independent C99/iAβ production pathway. The accumulation of AβPP-derived iAβ resumes and when it crosses the T1 threshold the neuronal ISR is elicited, the AβPP-independent C99/iAβ production pathway is activated, and the progression of AD pathology in these neurons commences.

Figure 25 considers the effects of the transient presence of the unconventional stressor capable of eliciting the neuronal ISR. The outcomes of the transient occurrence of unconventional stressors are multiple and defined by whether or not the levels of iAβ (produced mainly independently of AβPP) have crossed the T1 threshold at the time of withdrawal of the unconventional stressor. In Panel A of Figure 25, the unconventional stressor occurs when the levels of AβPP-derived iAβ are well below the T1 threshold. The rate of accumulation of the latter is such that it would not reach the T1 threshold within the individual’s lifetime, and no AD would develop (projected accumulation of AβPP-derived iAβ in the absence of the unconventional stressor is shown as broken lines). Following the occurrence of the unconventional stressor, the neuronal ISR is unconventionally elicited and the AβPP-independent C99/iAβ production pathway unconventionally activated. The levels of iAβ produced independently of AβPP rapidly increase. But before they reach the T1 threshold, the unconventional stressor is withdrawn. The ISR conditions are reversed, and without the supply of its essential components the operation of the AβPP-independent C99/iAβ production pathway ceases. The accumulation of AβPP-derived iAβ resumes from an elevated baseline but proceeds at a low rate, supported only by the AβPP proteolysis. It does not reach the T1 threshold within the lifetime of the individual and no AD occurs.

In Panel B of Figure 25, the unconventional stressor occurs at the low levels of AβPP-derived iAβ and persists for a considerable duration. Following its occurrence, the neuronal ISR is unconventionally elicited, the AβPP-independent C99/iAβ production pathway is unconventionally activated, and iAβ produced independently of AβPP rapidly accumulates. When the unconventional stressor is withdrawn, the levels of iAβ have crossed the T1 threshold in all affected neurons. The AβPP-independent C99/iAβ production pathway has attained self-sustainability and the withdrawal of the initial unconventional stressor has no effect whatsoever on its continuous operation.

In Panel C of Figure 25, the unconventional stressor is withdrawn when iAβ produced independently of AβPP has crossed the T1 threshold in only a fraction of affected neurons. In these neurons the AβPP-independent C99/iAβ production pathway has attained self-sustainability and its continuous operation is not affected by the withdrawal of the initial unconventional stressor. In the neurons that have not yet crossed the T1 threshold, the withdrawal of the unconventional stressor terminates the operation of the AβPP-independent C99/iAβ production pathway. The accumulation of AβPP-derived iAβ resumes and when it crosses the T1 threshold the neuronal ISR is elicited, the AβPP-independent C99/iAβ production pathway is activated, and the progression of AD pathology in these neurons commences.

## 55. Numerous Transient Microbursts of Unconventional Activity of the AβPP-Independent C99/iAβ Production Pathway Potentially Contribute to the “Normal” Under-T1 Accumulation of iAβ; Conventional AD Always Contains an Unconventional Component

The present section develops further the concept illustrated in Panel A of Figure 25, namely that a burst of the unconventional activity of the AβPP-independent C99/iAβ production pathway occurring at AβPP-derived iAβ levels below the T1 threshold may have benign consequences and does not necessarily result in AD. Conceivably, such bursts, more appropriately “microbursts”, of unconventional activity of the AβPP-independent C99/iAβ production pathway may occur dozens of times during the lifetime of an individual, reflecting numerous episodes of viral or bacterial infection and of transient inflammation events, occurring without causing AD (because levels of iAβ remain below the T1 threshold and the AβPP-independent C99/iAβ production pathway does not attain self-sustainability) and thus remaining undetected and unconsequential. Indeed, many, if not most, of the transient viral and bacterial infections may be accompanied by the transient microbursts of unconventional activity of the AβPP-independent C99/iAβ production pathway. When infection ends, the unconventional neuronal ISR-eliciting stressor is withdrawn and the activity of the AβPP-independent C99/iAβ production pathway ceases. It follows that the “normal” kinetics of accumulation of iAβ in the healthy individual are not uniformly linear (as depicted in Panel A of Figure 24) but composed of the linear portions of AβPP-derived iAβ accumulation interspersed by dozens of micro-steps, each resulting from the transient unconventional activation (“microburst”) of the AβPP-independent C99/iAβ production pathway; as long as the levels of iAβ do not cross the T1 threshold, no AD occurs.

Above, in Section 24, it was stated that prior to reaching the T1 threshold iAβ accumulates physiologically in two ways. One is the retention of Aβ generated by gamma-cleavages occurring on intraneuronal membranes within various cellular organelles. Another way is the importation of secreted extracellular AβPP-derived Aβ. In view of the above considerations, we must add yet another way: the production of iAβ in microbursts of the activity of the AβPP-independent C99/iAβ generation pathway. This is not a physiological mechanism, but it is “normal” in that it potentially occurs routinely and repeatedly in every individual; it either remains uncosequential (if the T1 threshold is not crossed) or contributes to the occurrence of AD (if the T1 threshold is crossed).

**Figure 26:**
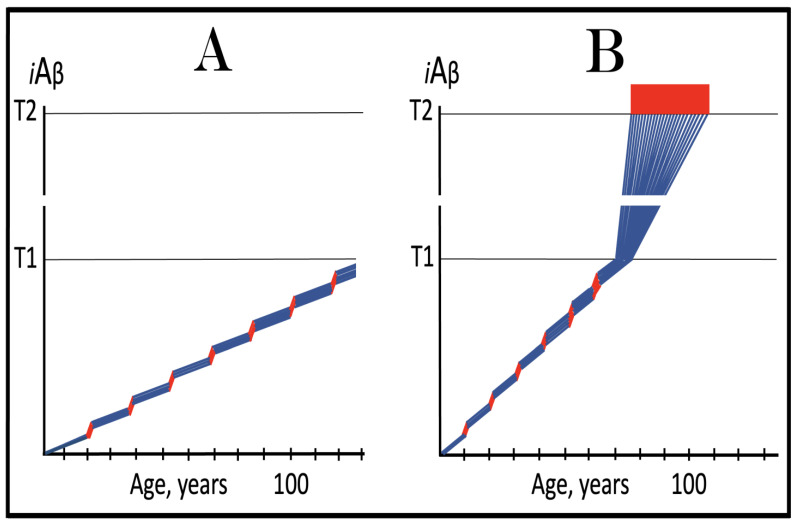
**Microbursts of unconventional activity of the AβPP-independent C99/iAβ production pathway contribute to the normal Under-T1 accumulation of iAβ**. *Blue lines*: Intraneuronal Aβ, iAβ. iAβ produced in the microbursts of the unconventional activity of the AβPP-independent C99/iAβ generation pathway. ***T1***: Threshold of cellular concentration of iAβ triggering the activation of PKR and/or HRI kinases, phosphorylation of eIF2α, the elicitation of the neuronal ISR, the initiation of the AβPP-independent C99 production pathway, and the commencement of AD. ***T2***: Cellular concentration of iAβ triggering neuronal death via apoptosis or necroptosis. *Red box*: Apoptotic zone, defined as a range of cellular concentrations of iAβ within which cells neuronal cells committed apoptosis or necroptosis or are dead. *Panel* (**A**): The accumulation of iAβ in neurons of the healthy individual. Unconventional neuronal ISR-eliciting stressors appear for only a short duration but on numerous occasions. Every time their occurrence causes a microburst of the activity of the AβPP-independent C99/iAβ generation pathway and the levels of iAβ rapidly increase. Each burst concludes with the withdrawal of the unconventional stressor and the resumption of the slow accumulation of AβPP-derived iAβ from the elevated baseline. Despite numerous microbursts, the levels of iAβ do not reach the T1 threshold within the lifetime of the individual and no AD occurs. *Panel* (**B**): The rate of accumulation of AβPP-derived iAβ combined with the extents and number of microbursts generating iAβ independently of AβPP are such that the T1 threshold is reached and crossed. The neuronal ISR is sustainably elicited, the self-sustainable AβPP-independent C99/iAβ generation pathway is activated, and conventional AD commences and progresses. Thus, numerous microbursts of the unconventional activity of the AβPP-independent C99/iAβ production pathway potentially occur “normally”, without adverse effects, in healthy individuals. On the other hand, conventional AD potentially always contains an unconventional component in the form of the microbursts of the unconventional activity of the AβPP-independent C99/iAβ generation pathway.

The above notion is illustrated in Figure 26. Panel A of Figure 26 shows the under-T1 accumulation of iAβ in neurons of the healthy individual. Blue lines depict iAβ derived from AβPP whereas red lines show iAβ produced in microbursts independently of AβPP. Every time the unconventional ISR-eliciting stressor appears, and the burst occurs, the levels of iAβ are rapidly increased. Each burst concludes with the withdrawal of the unconventional stressor and the resumption of the slow accumulation of AβPP-derived iAβ from the elevated baseline. Despite numerous microbursts, the levels of iAβ do not reach the T1 threshold within the lifetime of the individual and no AD occurs.

In Panel B of Figure 26, the rate of accumulation of AβPP-derived iAβ combined with the extents and number of microbursts generating iAβ independently of AβPP is such that the T1 threshold is reached and crossed. The neuronal ISR is sustainably elicited, the self-sustainable AβPP-independent C99/iAβ generation pathway is activated, and conventional AD commences and progresses. Thus, numerous microbursts of the unconventional activity of the AβPP-independent C99/iAβ production pathway potentially occur “normally”, without adverse effects, in healthy individuals. On the other hand, conventional AD potentially always contains an unconventional component in the form of the contribution of iAβ generated in numerous microbursts of the unconventional activity of the AβPP-independent C99/iAβ generation pathway to reaching and crossing the T1 threshold.

## 56. Traumatic Brain Injury (TBI) and Chronic Traumatic Encephalopathy (CTE) as Causes of Unconventional AD

In Panel A of Figure 25, shown above, the duration of the operation of the unconventionally activated AβPP-independent C99/iAβ generation pathway is substantial yet AβPP-derived iAβ, accumulating from an elevated baseline following the withdrawal of the unconventional stressor, does not reach the T1 threshold within the lifetime of the individual, and no AD occurs. However, with a sufficient increase in the duration of the operation of the unconventionally activated AβPP-independent C99/iAβ generation pathway and consequent elevation of the baseline of the resumed accumulation of AβPP-derived iAβ (to levels still below the T1) (following the withdrawal of an unconventional stressor), the latter would inevitably cross the T1 threshold and AD would occur. In such a case the timing of the T1 crossing by AβPP-derived iAβ and of the commencement of AD would be inversely proportional to the duration of the operation of the unconventionally activated AβPP-independent C99/iAβ generation pathway.

**Figure 27:**
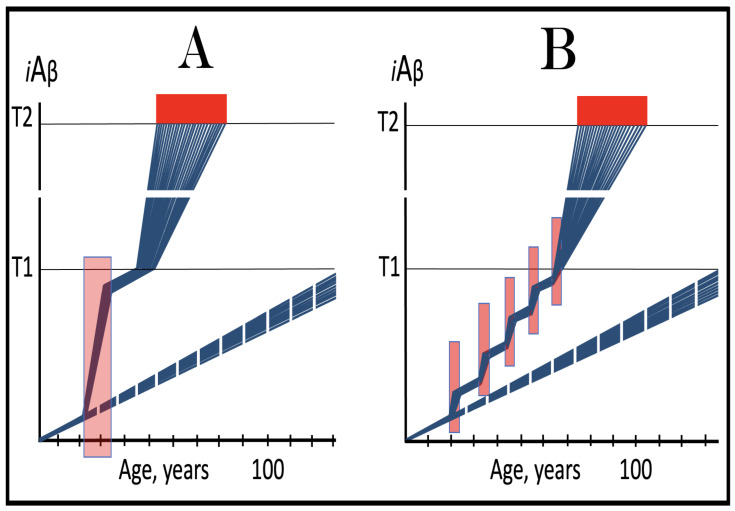
**TBI and CTE cause unconventional AD via the unconventional transient activation of the AβPP-independent C99/iAβ generation pathway**. *Blue lines*: Intraneuronal Aβ, iAβ. ***T1***: Threshold of cellular concentration of iAβ triggering the activation of PKR and/or HRI kinases, phosphorylation of eIF2α, the elicitation of the neuronal ISR, the initiation of the AβPP-independent C99 production pathway, and the commencement of AD. ***T2***: Cellular concentration of iAβ triggering neuronal death via apoptosis or necroptosis. *Red box*: Apoptotic zone, defined as a range of cellular concentrations of iAβ within which cells neuronal cells committed apoptosis or necroptosis or are dead. *Pink boxes*: Duration of the occurrence of the unconventional stressor capable of activating one or more of eIF2α kinases and triggering the elicitation of the neuronal ISR. *Broken lines*: The projected dynamics of the accumulation of AβPP-derived iAβ in the absence of the episodes of unconventional activity of the AβPP-independent C99 production pathway; no T1 would be crossed within the lifetime of the individual, no AD would occur. *Panel* (**A**): TBI provides, probably via the suppression of CBF, the unconventional neuronal ISR-eliciting stressor and thus enables the unconventional activation and transient operation of the AβPP-independent C99/iAβ generation pathway. Upon withdrawal of the unconventional stressor, AβPP-derived iAβ resumes its accumulation from the elevated (but still under-T1) baseline. When the T1 is crossed, PKR and/or HRI are activated, eIF2α is phosphorylated, the neuronal ISR is elicited, the self-sustainable AβPP-independent C99/iAβ generation pathway is initiated, and AD commences. The time period between the TBI event and the commencement of AD is defined by the severity of TBI and, consequently, by the duration of the presence of the unconventional neuronal ISR-eliciting stressor. *Panel* (**B**): In CTE, the severity of trauma is substantially smaller than in TBI, but it does provide, potentially through the suppression of CBF, an unconventional neuronal ISR-eliciting stressor. The duration of the transient operation of the unconventionally activated AβPP-independent C99/iAβ generation pathway is shorter and the extent of the elevation of the baseline of the resumed accumulation of AβPP-derived iAβ is smaller in CTE than in TBI. But in the former traumatic events occur repeatedly, and after each event the baseline of the resumed accumulation of AβPP-derived iAβ is elevated further until, eventually, the T1 threshold is crossed, the self-sustainable AβPP-independent C99/iAβ generation pathway is activated, and AD commences.

The above scenario is illustrated in Panel A of Figure 27. The duration of the operation of the unconventionally activated AβPP-independent C99/iAβ generation pathway is such that upon its termination the levels of iAβ produced independently of AβPP are relatively close to but still below the T1 threshold. The accumulation of AβPP-derived iAβ resumes from this elevated baseline and relatively soon reaches and crosses the T1 threshold. PKR and/or HRI are activated, eIF2α is phosphorylated, the neuronal ISR is elicited, the self-sustainable AβPP-independent C99/iAβ generation pathway is initiated, and AD commences. In terms of the ACH2.0 this scenario describes how traumatic brain injury causes AD. TBI provides the unconventional neuronal ISR-eliciting stressor (probably via the suppression of CBF) and thus enables the unconventional activation and transient operation of the AβPP-independent C99/iAβ generation pathway. Upon withdrawal of the unconventional stressor, AβPP-derived iAβ resumes its accumulation from the elevated (but still under-T1) baseline and when the T1 is crossed, the disease commences. The time period between the TBI event and the commencement of AD is defined by the severity of TBI and, consequently, by the duration of the presence of the unconventional neuronal ISR-eliciting stressor.

Panel B of Figure 27 illustrates how chronic traumatic encephalopathy leads to AD. In CTE, the severity of trauma is substantially smaller than in TBI, but it does provide an unconventional neuronal ISR-eliciting stressor (probably by the same mechanism as TBI, namely the suppression of CBF). Consequently, the duration of the transient operation of the unconventionally activated AβPP-independent C99/iAβ generation pathway is shorter and the extent of the elevation of the baseline of the resumed accumulation of AβPP-derived iAβ is smaller. But in CTE traumatic events occur repeatedly (e.g., during boxing, football, hockey matches, or any other contact events), and after each event the baseline of the resumed accumulation of AβPP-derived iAβ is elevated further until, eventually and inevitably, the T1 threshold is crossed, the self-sustainable AβPP-independent C99/iAβ generation pathway is activated, and AD commences. Importantly, in both panels the disease is due solely to TBI and CTE and consequent episodes of the unconventional activity of the AβPP-independent C99 generation pathway; in their absence AβPP-derived iAβ would not reach (broken blue lines) the T1 threshold within the lifetime of the individuals and no AD would occur.

## 57. ACH-Based Drugs Would Have No Effect Whatsoever in Unconventional AD

As discussed above (see Section 36 and Section 37), in terms of the ACH2.0, the ACH-based AD drugs exert their potentially preventive effect (on conventional AD) by reducing the rate of accumulation of AβPP-derived iAβ or elevating the extent of the T1 threshold. This translates into a delay or prevention of the crossing of the T1 threshold by AβPP-derived iAβ. Consequently, no ISR is elicited, the AβPP-independent C99/iAβ production pathway is not activated, and no AD occurs. ACH-based drugs, however, have no effect on the AβPP-independent C99/iAβ production pathway and thus are completely ineffective when this pathway is operational. It follows that ACH-based drugs can have no effect whatsoever in unconventional AD. Indeed, the occurrence of unconventional ISR-eliciting stressors does not depend in any way on and can take place at any level of AβPP-derived iAβ. Modulating the latter would have no conceivable effect on the former. Moreover, when the AβPP-independent C99/iAβ production pathway is unconventionally activated, ACH-based drugs cannot interfere in any way with its operation. On the other hand, ACH2.0-based drugs can be effective in unconventional AD. Their anticipated effects in both the prevention and treatment of the disease are discussed in the following sections below.

## 58. ISR Inhibitors in the Prevention and Treatment of Unconventional AD

**Figure 28:**
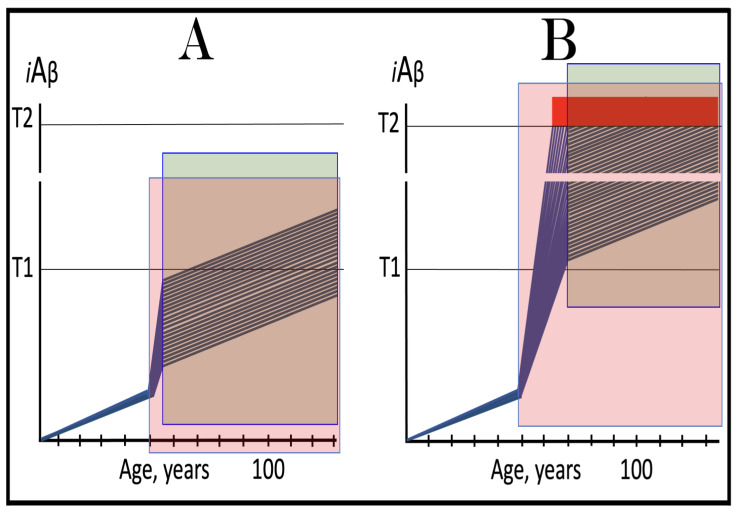
**Inhibitors of the neuronal integrated stress response in the prevention and treatment of unconventional AD.** *Blue lines*: Intraneuronal Aβ, iAβ. ***T1***: Threshold of cellular concentration of iAβ triggering the activation of PKR and/or HRI kinases, phosphorylation of eIF2α, the elicitation of the neuronal ISR, the initiation of the AβPP-independent C99 production pathway, and the commencement of AD. ***T2***: Cellular concentration of iAβ triggering neuronal death via apoptosis or necroptosis. *Red box*: Apoptotic zone, defined as a range of cellular concentrations of iAβ within which cells neuronal cells committed apoptosis or necroptosis or are dead. *Pink boxes*: The duration of the presence of the unconventional stressor capable of activating one or more of eIF2α kinases and triggering the elicitation of the neuronal ISR. *Green box*: The duration of the treatment with the ISR-inhibiting drug. *Panel* (**A**): ISR inhibitors in the prevention of unconventional AD. Following the occurrence of the unconventional stressor, the neuronal ISR has been unconventionally elicited and the AβPP-independent C99/iAβ production pathway unconventionally activated. iAβ levels have rapidly increased but have not yet reached the T1 threshold. The administration of ISR inhibitors at this point reverse the ISR conditions, deprive the AβPP-independent C99/iAβ production pathway of its essential components and render it inoperative. The accumulation of iAβ continues, but at a low rate, supported only by the AβPP proteolysis. The crossing of the T1 threshold would be inconsequential since no ISR can be elicited in the presence of the drug. The levels of iAβ would not reach the AD pathology-causing range and AD would not occur for the duration of the treatment. *Panel* (**B**): ISR inhibitors in the treatment of unconventional AD. Inhibition of the neuronal ISR deprives the AβPP-independent C99/iAβ production pathway of its essential components and its operation ceases. The rate of accumulation of iAβ, produced at this point only by the AβPP proteolysis, significantly decreases. The progression of the disease continues but at a much slower rate for the duration of the treatment.

The present and following sections are concerned with the therapeutic strategies for unconventional AD. In these considerations, it is assumed that unconventional stressors capable of the elicitation of the neuronal ISR, once they appear, persist for the remaining lifetime (their withdrawal prior to the T1 crossing would render the disease, provided it eventually occurs, conventional, and their removal following the T1 crossing is inconsequential since the AβPP-independent C99/iAβ production pathway is self-sustainable at this point). Unconventional AD is driven by the self-sustainable AβPP-independent C99/iAβ production pathway, which is activated by the sustained neuronal ISR. The suppression of the neuronal integrated stress response is, therefore, an intuitive therapeutic strategy in both the prevention and treatment of the disease. Such therapeutic strategies are presented in Figure 28.

Panel A of Figure 28 depicts schematically the effects of inhibition of the neuronal integrated stress response in the prevention of unconventional AD. In this panel, the unconventional stressor has occurred at the levels of AβPP-derived iAβ well below the T1 threshold. The neuronal ISR has been unconventionally elicited and the AβPP-independent C99/iAβ production pathway unconventionally activated. iAβ levels have rapidly increased but have not yet reached the T1 threshold. The administration of ISR inhibitors at this point would reverse the ISR conditions, deprive the AβPP-independent C99/iAβ production pathway of its essential components and render it inoperative. The accumulation of iAβ would continue at a low rate, supported only by the AβPP proteolysis. The crossing of the T1 threshold would be inconsequential since no ISR can be elicited in the presence of the drug. The levels of iAβ would not reach the AD pathology-causing range and AD would not occur in treated individuals.

In Panel B of Figure 28, the administration of the ISR suppressing drug commences when the T1 threshold has been crossed in all affected neurons; a fraction of the neurons has also crossed the T2 threshold, and AD symptoms have manifested. The administration of the ISR-inhibiting drug deprives the AβPP-independent C99/iAβ production pathway of its essential components, and its operation ceases. The rate of accumulation of iAβ, produced at this point only by the AβPP proteolysis, significantly decreases. Accordingly, the progression of the disease continues but at a much slower rate. Thus, both strategies, preventive and curative, would be beneficial. However, for the reasons discussed above (Section 43) they are, apparently, unfeasible due to anticipated adverse effects of systemically long-term administered ISR inhibitors. A solution would be either the development of neuron-specific ISR inhibitors or selective delivery of the drug to the neurons alone.

## 59. Activation of BACE1 and/or BACE2 in the Prevention of Unconventional Alzheimer’s Disease

As discussed above (Section 46), it can be anticipated that transient activation of BACE1 and/or BACE2 would prevent conventional AD and AACD for the remaining portion of the lifetime of the treated individual. This is because, in the absence of the activity of the AβPP-independent C99/iAβ production pathway, iAβ is substantially depleted by activated BACE1 and/or BACE2. Following the withdrawal of the drug, the accumulation of AβPP-derived iAβ resumes from a low baseline and would reach neither T^0^ nor T1 thresholds within the lifetime of the treated person. The situation in unconventional AD is fundamentally different in that the AβPP-independent C99/iAβ production pathway is operational even prior to the T1 crossing. Consequently, the rate of degradation of iAβ by activated BACE1 and/or BACE2 cannot match the rate of influx of iAβ produced independently of AβPP. The best possible outcome, therefore, is the reduction in the rate of accumulation of iAβ generated in the AβPP-independent C99/iAβ production pathway.

**Figure 29:**
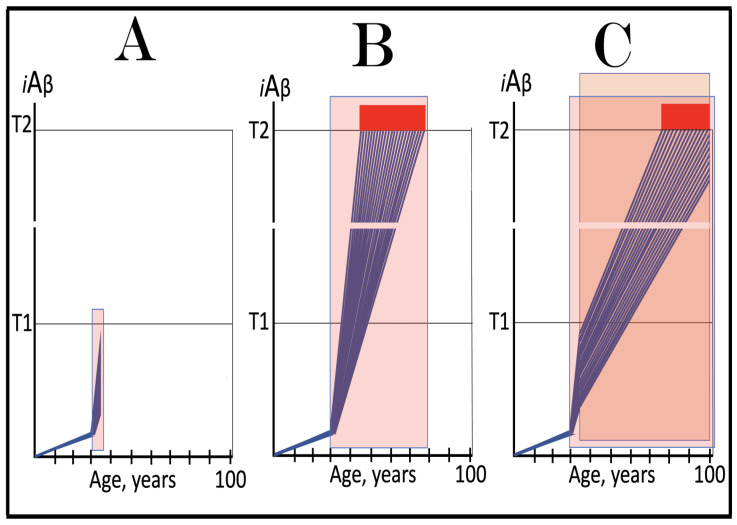
**Activation of BACE1 and/or BACE2 in the prevention of unconventional AD**. *Blue lines*: Intraneuronal Aβ, iAβ. ***T1***: Threshold of cellular concentration of iAβ triggering the activation of PKR and/or HRI kinases, phosphorylation of eIF2α, the elicitation of the neuronal ISR, the initiation of the AβPP-independent C99 production pathway, and the commencement of AD. ***T2***: Cellular concentration of iAβ triggering neuronal death via apoptosis or necroptosis. *Red box*: Apoptotic zone, defined as a range of cellular concentrations of iAβ within which cells neuronal cells committed apoptosis or necroptosis or are dead. *Pink boxes*: The duration of the presence of the unconventional stressor capable of activating one or more of eIF2α kinases and triggering the elicitation of the neuronal ISR. *Orange box*: The duration of the treatment with the BACE1 and/or BACE2-activating drug. *Panel* (**A**): The initial state of the levels of iAβ in individual neurons at the time of the commencement of the drug’s administration. In these neurons, the occurrence of the unconventional stressor elicited the neuronal ISR, which, in turn, provided components essential for the operation of the AβPP-independent C99/iAβ production pathway and thus activated it. iAβ generated independently of AβPP rapidly accumulated but is still below the T1 threshold. *Panel* (**B**): The evolution of the initial state in the untreated individual. The accumulation of iAβ produced independently of AβPP continues unchecked. It crosses the T1 threshold and the AβPP-independent C99/iAβ production pathway is rendered self-sustainable. AD commences and progresses until it reaches its end stage. *Panel* (**C**): The evolution of the initial state in the individual treated with the BACE1 and/or BACE2-activating drug. In the presence of the drug, the rate of the efflux of iAβ increases but it cannot match that of the influx of iAβ produced independently of AβPP. However, the rate of the accumulation of iAβ is reduced and the T1 crossing is delayed. The commencement of AD is also delayed, and its progression is slowed down for the duration of the treatment.

The above considerations are illustrated in Figure 29. Panel A of Figure 29 depicts the initial state of the levels of iAβ in individual neurons at the time of the commencement of the drug’s administration. In these neurons, the occurrence of the unconventional stressor elicited the neuronal integrated stress response, which, in turn, provided components essential for the operation of the AβPP-independent C99/iAβ production pathway and thus activated the pathway. iAβ generated independently of AβPP rapidly accumulated but is still below the T1 threshold. Panel B of Figure 29 shows the evolution of the initial state in the untreated individual. The accumulation of iAβ produced independently of AβPP continues unhindered. It crosses the T1 threshold and the AβPP-independent C99/iAβ production pathway is rendered self-sustainable. AD commences and progresses until it reaches its end stage. Panel C of Figure 29 presents the evolution of the initial state in the individual treated with the BACE1 and/or BACE2-activating drug. In the presence of the drug the rate of the efflux of iAβ increases but it cannot match that of the influx of iAβ produced independently of AβPP. However, the rate of the accumulation of iAβ is reduced and the T1 crossing is delayed. Accordingly, the commencement of AD is also delayed, and its progression is slowed down for the duration of the treatment.

## 60. Activation of BACE1 and/or BACE2 in the Treatment of Unconventional Alzheimer’s Disease

**Figure 30:**
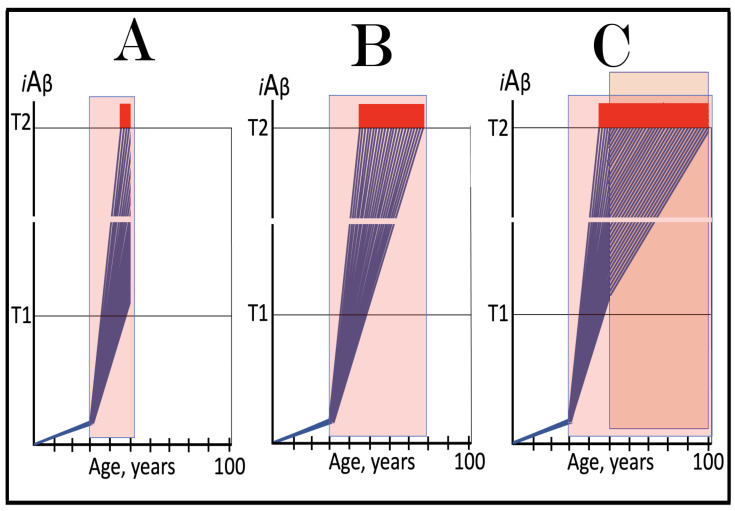
**Activation of BACE1 and/or BACE2 in the treatment of unconventional AD**. *Blue lines*: Intraneuronal Aβ, iAβ. ***T1***: Threshold of cellular concentration of iAβ triggering the activation of PKR and/or HRI kinases, phosphorylation of eIF2α, the elicitation of the neuronal ISR, the initiation of the AβPP-independent C99 production pathway, and the commencement of AD. ***T2***: Cellular concentration of iAβ triggering neuronal death via apoptosis or necroptosis. *Red box*: Apoptotic zone, defined as a range of cellular concentrations of iAβ within which cells neuronal cells committed apoptosis or necroptosis or are dead. *Pink boxes*: The duration of the presence of the unconventional stressor capable of activating one or more of eIF2α kinases and triggering the elicitation of the neuronal ISR. *Orange box*: The duration of the treatment with the BACE1 and/or BACE2-activating drug. *Panel* (**A**): The initial state of the levels of iAβ in individual neurons at the time of the commencement of the drug’s administration. The neuronal ISR has been unconventionally elicited and the AβPP-independent C99/iAβ production pathway unconventionally activated at the levels of AβPP-derived iAβ well below the T1 threshold. iAβ produced independently of AβPP rapidly accumulated and crossed the T1 threshold in all affected neurons, and the AβPP-independent C99/iAβ production pathway was rendered self-sustainable. AD commenced and progressed; a fraction of the neurons crossed the T2 threshold, and AD symptoms manifested. *Panel* (**B**): The evolution of the initial state in the untreated individual. iAβ produced independently of AβPP continues to accumulate unhindered, additional neurons cross the T2 threshold, and the disease enters its end stage. *Panel* (**C**): The evolution of the initial state in the individual treated with the BACE1 and/or BACE2-activating drug. iAβ is being degraded by the intra-iAβ cleaving activities of BACE1 and/or BACE2 but the rate of its efflux is significantly smaller than that of its influx. Its accumulation continues and the disease progresses, although at a decreased rate, for the duration of the treatment.

Whereas the outcomes of the preventive application of activators of BACE1 and/or BACE2 differ radically in conventional and unconventional AD, they are conceptually similar in the treatment of both forms of the disease. This is because in both, at the time of treatment, the AβPP-independent C99/iAβ production pathway is operational (in the preventive application of BACE1 and/or BACE2-activating drugs this pathway is operational in unconventional AD but inoperative in the conventional form of the disease). Effects of BACE1 and/or BACE2-activating drugs in symptomatic unconventional AD are considered in Figure 30. Panel A of Figure 30 shows the initial state of the levels of iAβ at the commencement of drug’s administration. At this stage, the neuronal integrated stress response has been unconventionally elicited and the AβPP-independent C99/iAβ production pathway unconventionally activated at the levels of AβPP-derived iAβ well below the T1 threshold. iAβ produced independently of AβPP has rapidly accumulated, crossed the T1 threshold in all affected neurons, and the AβPP-independent C99/iAβ production pathway was rendered self-sustainable. AD commenced and progressed; a fraction of the neurons has crossed the T2 threshold, and AD symptoms manifested. Panel B of Figure 30 depicts the evolution of the initial state in the untreated individual. iAβ produced independently of AβPP continues to accumulate unhindered, further neurons cross the T2 threshold, and the disease enters its end stage. Panel C of Figure 30 presents the evolution of the initial state in the presence of the BACE1 and/or BACE2-activating drug. iAβ is being degraded by the intra-iAβ cleaving activities of BACE1 and/or BACE2 but the rate of its efflux is significantly smaller than that of its influx. Its accumulation continues and the disease progresses, although at a decreased rate.

## 61. Composite Transient ISR Inhibition/Long-Term BACE Activation Therapy in the Prevention and Treatment of Unconventional AD

The BACE1 and/or BACE2 activation therapy in the prevention and treatment of unconventional AD, considered in two preceding sections, is beneficial. However, it neither prevents the disease nor does it stop the progression of AD. On the other hand, this therapy can be significantly improved upon. One of the main reasons for its inefficiency is that it commences at the high rates of iAβ production (in the AβPP-independent pathway) and no depletion of iAβ takes place during the therapy. A solution for this is the composite therapy described above for the treatment of conventional AD (see Section 48). In this approach, inhibitors of the ISR are administered transiently and concurrently with activators of BACE1 and/or BACE2, also administered transiently. The former disable the AβPP-independent C99/iAβ production pathway and interrupt the influx of iAβ generated independently of AβPP. Consequently, the latter are capable of depleting iAβ substantially below the T1 threshold. When drugs are withdrawn, the de novo accumulation of iAβ commences from a low baseline and (since it is conventional AD) is supported only by the AβPP proteolysis. Its levels do not reach the T1 threshold, and the disease does not recur within the lifetime of the treated patient.

**Figure 31:**
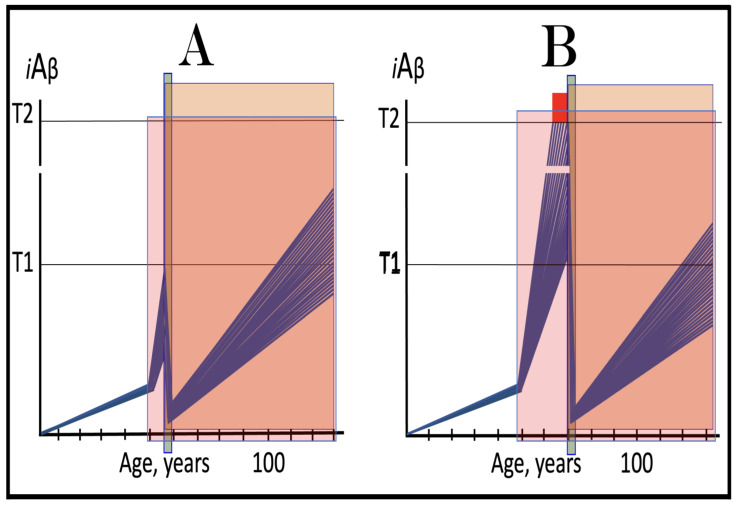
**Composite transient ISR inhibition/long-term BACE activation therapy in the prevention and treatment of unconventional AD**. *Blue lines*: Intraneuronal Aβ, iAβ. *T1*: Threshold of cellular concentration of iAβ triggering the activation of PKR and/or HRI kinases, phosphorylation of eIF2α, the elicitation of the neuronal ISR, the initiation of the AβPP-independent C99 production pathway, and the commencement of AD. *T2*: Cellular concentration of iAβ triggering neuronal death via apoptosis or necroptosis. *Red box*: Apoptotic zone, defined as a range of cellular concentrations of iAβ within which cells neuronal cells committed apoptosis or necroptosis or are dead. *Pink boxes*: The duration of the presence of the unconventional stressor capable of activating one or more of eIF2α kinases and triggering the elicitation of the neuronal ISR. *Green box*: The duration of the treatment with the ISR-inhibiting drug. *Orange box*: The duration of the treatment with the BACE1 and/or BACE2-activating drug. *Panel* (**A**): The neuronal ISR is unconventionally elicited and the AβPP-independent C99/iAβ production pathway unconventionally activated at the levels of AβPP-derived iAβ well below the T1 threshold. The levels of iAβ generated independently of AβPP rapidly increase but at the time of the commencement of the composite therapy do not yet reach the T1 threshold. The ISR inhibitor disables the AβPP-independent C99/iAβ production pathway and stops the influx of iAβ generated independently of AβPP. The concurrent activation of BACE1 and/or BACE2 substantially depletes iAβ. Following the withdrawal of the ISR inhibitor, the neuronal ISR is re-elicited and the AβPP-independent C99/iAβ production pathway reactivated. The continuous presence of the BACE activator reduces the rate of accumulation of iAβ produced independently of AβPP and its levels increase relatively slowly. They do not reach the AD pathology-causing range and AD does not occur for the duration of the treatment. *Panel* (**B**): iAβ produced independently of AβPP has crossed the T1 threshold in all affected neurons and the AβPP-independent C99/iAβ production pathway has been rendered self-sustainable. At the time of the commencement of the composite therapy a fraction of the neurons had crossed the T2 threshold and AD symptoms manifested. The presence of the ISR inhibitor abrogates the influx of iAβ produced independently of AβPP and allows the activator of BACE1 and/or BACE2 to substantially reduce iAβ levels. Following the withdrawal of the ISR inhibitor, the neuronal ISR is unconventionally re-elicited and the AβPP-independent C99/iAβ production pathway unconventionally reactivated. However, the continuous presence of the BACE1 and/or BACE2 activator maintains a relatively low rate of accumulation of iAβ. It does not reach the AD pathology-causing levels and AD does not recur for the duration of the treatment.

The same composite therapy can be applied in the prevention and treatment of unconventional AD. In this case, however, following the withdrawal of the ISR inhibitor (which can be applied only transiently to avoid adverse effects), the neuronal ISR would be re-elicited and the AβPP-independent C99/iAβ production pathway reactivated. Therefore, to reduce the rate of accumulation of iAβ generated independently of AβPP, it is advantageous to continue the activation of BACE1 and/or BACE2 long-term. Effects of such a strategy in the prevention and treatment of unconventional AD are illustrated in Figure 31. In Panel A of Figure 31, the neuronal ISR is unconventionally elicited and the AβPP-independent C99/iAβ production pathway unconventionally activated at the levels of AβPP-derived iAβ well below the T1 threshold. The levels of iAβ generated independently of AβPP rapidly increase but at the time of the commencement of the composite therapy do not yet reach the T1 threshold. The ISR inhibitor disables the AβPP-independent C99/iAβ production pathway and stops the influx of iAβ generated independently of AβPP. The concurrent activation of BACE1 and/or BACE2 substantially depletes iAβ. Following the withdrawal of the ISR inhibitor, the neuronal ISR is re-elicited and the AβPP-independent C99/iAβ production pathway reactivated. The continuous presence of the BACE activator reduces the rate of accumulation of iAβ produced independently of AβPP and its levels increase relatively slowly. As shown, they do not reach the AD pathology-causing range and AD does not occur for the duration of the treatment.

In Panel B of Figure 31, iAβ produced independently of AβPP has crossed the T1 threshold in all affected neurons. The AβPP-independent C99/iAβ production pathway has been rendered self-sustainable and AD has commenced and progressed. At the time of the commencement of the composite therapy a fraction of the neurons had crossed the T2 threshold and AD symptoms manifested. The presence of the ISR inhibitor abrogates the influx of iAβ produced independently of AβPP and allows the activator of BACE1 and/or BACE2 to substantially reduce iAβ levels. Following the withdrawal of the ISR inhibitor, the neuronal ISR is unconventionally re-elicited and the AβPP-independent C99/iAβ production pathway unconventionally reactivated. However, the continuous presence of the BACE1 and/or BACE2 activator maintains a relatively low rate of accumulation of iAβ. As depicted in Panel B, it does not reach the AD pathology-causing levels and AD does not recur for the duration of the treatment.

## 62. Transient Composite ISR Inhibition/BACE Activation Therapy in the Prevention and Treatment of Unconventional AD

Life-long activation of the intra-iAβ cleavage at the β’-site confers only benefits and no adverse effects in the carriers of the Icelandic AβPP mutations. This indicates that the long-term activation of BACE1 and/or BACE2 would likely not be harmful. On the other hand, a relatively long-term inhibition of BACE1 in AD patients resulted in substantial adverse effects [27,28]. However, if the long-term utilization of BACE1 and/or BACE2 activators turns out to be problematic, their transient application as a part of the composite therapy would also be beneficial and would offer a viable therapeutic option. Therefore, the present and following sections consider effects of the composite transient ISR inhibition/BACE activation therapy in the prevention and treatment of unconventional AD.

**Figure 32:**
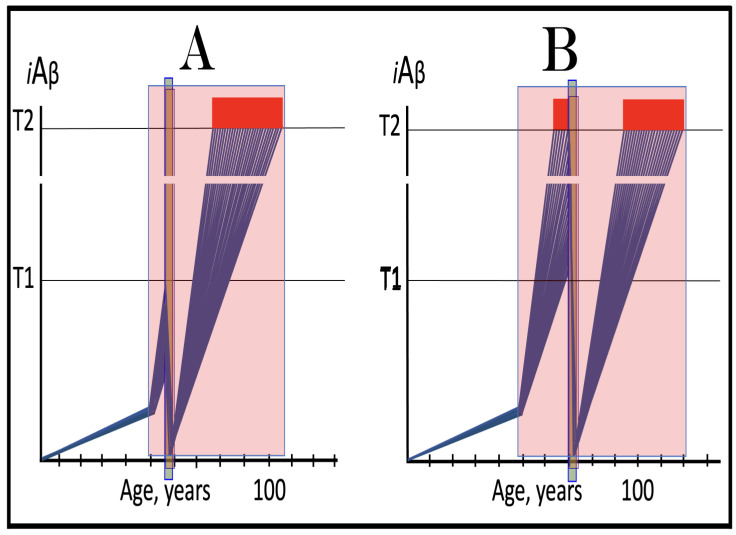
**Transient composite ISR inhibition/BACE activation therapy in the prevention and treatment of unconventional AD**. *Blue lines*: Intraneuronal Aβ, iAβ. ***T1***: Threshold of cellular concentration of iAβ triggering the activation of PKR and/or HRI kinases, phosphorylation of eIF2α, the elicitation of the neuronal ISR, the initiation of the AβPP-independent C99 production pathway, and the commencement of AD. ***T2***: Cellular concentration of iAβ triggering neuronal death via apoptosis or necroptosis. *Red box*: Apoptotic zone, defined as a range of cellular concentrations of iAβ within which cells neuronal cells committed apoptosis or necroptosis or are dead. *Pink boxes*: The duration of the presence of the unconventional stressor capable of activating one or more of eIF2α kinases and triggering the elicitation of the neuronal ISR. *Green box*: The duration of the treatment with the ISR-inhibiting drug. *Orange box*: The duration of the treatment with the BACE1 and/or BACE2-activating drug. *Panel* (**A**): The effect of the transient concurrent ISR inhibition and BACE1 and/or BACE2 activation in the prevention of unconventional AD. The neuronal ISR has been unconventionally elicited and the AβPP-independent C99/iAβ production pathway unconventionally activated at the levels of AβPP-derived iAβ well below the T1 threshold. iAβ generated independently of AβPP has rapidly accumulated but at the time of the implementation of the composite therapy is still below the T1 threshold. The treatment with the ISR inhibitor disables the AβPP-independent C99/iAβ production pathway and terminates the influx of its iAβ product. This enables a substantial depletion of iAβ by concurrently activated BACE. When both drugs are withdrawn, the neuronal IST is unconventionally re-elicited and the AβPP-independent C99/iAβ production pathway unconventionally reactivated. The accumulation of iAβ resumes from a low baseline but proceeds rapidly. When it crosses the T1 threshold the AβPP-independent C99/iAβ production pathway is rendered self-sustainable and AD commences. The composite therapy does not prevent the disease but provides a short reprieve. *Panel* (**B**): The effect of the transient concurrent ISR inhibition and BACE1 and/or BACE2 activation in the treatment of unconventional AD. At the time of the implementation of the composite therapy all affected neurons have crossed the T1 threshold, a fraction of the neurons has crossed the T2 threshold, and AD symptoms manifested. Inhibition of the neuronal ISR deprives the AβPP-independent C99/iAβ production pathway of its essential components, disables it, and stops the influx of iAβ generated independently of AβPP. The concurrent activation of BACE1 and/or BACE2 substantially depletes iAβ. Upon the completion of the treatment and the withdrawal of the drugs the neuronal ISR is re-elicited and the AβPP-independent C99/iAβ production pathway reactivated. De novo accumulation of iAβ commences from a low baseline but proceeds rapidly. When it reaches the T1 threshold, the disease recurs.

These effects are illustrated schematically in Figure 32. Panel A of Figure 32 depicts the anticipated effect of the concurrent transient administration of both, the ISR inhibitor and BACE1 and/or BACE2 activator in the prevention of unconventional AD. In this panel, the neuronal integrated stress response has been unconventionally elicited and the AβPP-independent C99/iAβ production pathway unconventionally activated at the levels of AβPP-derived iAβ well below the T1 threshold. iAβ generated independently of AβPP has rapidly accumulated but at the time of the implementation of the composite therapy is still below the T1 threshold. The treatment with the ISR inhibitor disables the AβPP-independent C99/iAβ production pathway and terminates the influx of its iAβ product. This empowers a substantial depletion of iAβ by concurrently activated BACE1 and/or BACE2. When both drugs, the ISR inhibitor and BACE activator, are withdrawn, the neuronal IST is unconventionally re-elicited and the AβPP-independent C99/iAβ production pathway unconventionally reactivated. The accumulation of iAβ resumes from a low baseline but is unimpeded and proceeds rapidly. When it crosses the T1 threshold the AβPP-independent C99/iAβ production pathway is rendered self-sustainable, AD commences and progresses, eventually reaching its end stage. The composite therapy does not prevent the disease but provides a reprieve of several AD-free years.

Panel B of Figure 32 depicts the anticipated effect of the concurrent transient administration of the ISR inhibitor and BACE1 and/or BACE2 activator in the treatment of unconventional AD. In this panel, the neuronal integrated stress response has been unconventionally elicited and the AβPP-independent C99/iAβ production pathway unconventionally activated at the low levels of AβPP-derived iAβ. iAβ generated independently of AβPP has rapidly accumulated and crossed the T1 threshold in all affected neurons. The AβPP-independent C99/iAβ production pathway has been rendered self-sustainable and AD commenced. At the time of the implementation of the composite therapy a fraction of the neurons has crossed the T2 threshold and AD symptoms manifested. Inhibition of the neuronal ISR deprives the AβPP-independent C99/iAβ production pathway of its essential components, disables it, and stops the influx of iAβ generated independently of AβPP. The concurrent activation of BACE1 and/or BACE2 substantially depletes iAβ. Upon the completion of the treatment and the withdrawal of the drugs the neuronal ISR is re-elicited and the AβPP-independent C99/iAβ production pathway reactivated. De novo accumulation of iAβ commences from a low baseline but proceeds unimpeded and rapidly. When it reaches the T1 threshold, the disease recurs. Thus, the transient composite therapy does not prevent the recurrence of AD; it buys, however, a few disease-free years.

## 63. Recurrent Transient Composite ISR Inhibition/BACE Activation Therapy in the Prevention and Treatment of Unconventional AD

As described in the preceding section, the transient composite therapy consisting of the concurrent administration of the inhibitors of the integrated stress response and activators of BACE1 and/or BACE2 can neither prevent the occurrence of unconventional AD nor preclude its recurrence following the treatment. In both cases, however, the implementation of this therapy provides a temporary reprieve of several disease-free years. It follows that if, at strategically timed intervals defined by suitable biomarkers, the therapy would be repeated, the duration of the reprieve would be multiplied proportionally to the number of repeats. Potentially, the reprieve could be extended for the remaining portion of the lifetime of the treated individual.

**Figure 33:**
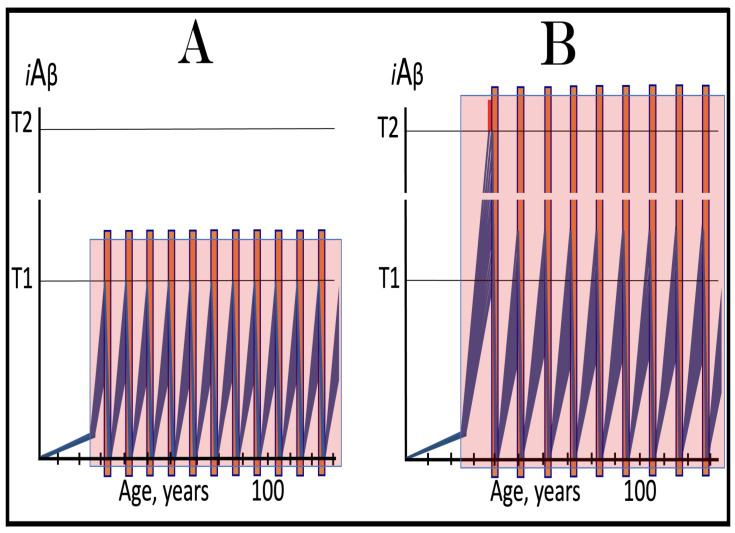
**Recurrent transient composite ISR inhibition/BACE activation therapy in the prevention and treatment of unconventional AD**. *Blue lines*: Intraneuronal Aβ, iAβ. ***T1***: Threshold of cellular concentration of iAβ triggering the activation of PKR and/or HRI kinases, phosphorylation of eIF2α, the elicitation of the neuronal ISR, the initiation of the AβPP-independent C99 production pathway, and the commencement of AD. ***T2***: Cellular concentration of iAβ triggering neuronal death via apoptosis or necroptosis. *Red box*: Apoptotic zone, defined as a range of cellular concentrations of iAβ within which cells neuronal cells committed apoptosis or necroptosis or are dead. *Pink boxes*: The duration of the presence of the unconventional stressor capable of activating one or more of eIF2α kinases and triggering the elicitation of the neuronal ISR. *Brown boxes*: The duration of the composite treatment with concurrently administered ISR inhibitors and BACE activators. *Panel* (**A**): Recurrent administration of the composite therapy in the prevention of unconventional AD. At the time of the implementation of the therapy the neuronal ISR has been unconventionally elicited and the AβPP-independent C99/iAβ production pathway unconventionally activated at the low levels of AβPP-derived iAβ. iAβ generated independently of AβPP has rapidly accumulated but has not yet reached the T1 threshold. The concurrent administration of ISR inhibitors and BACE activators disables the AβPP-independent C99/iAβ production pathway and substantially depletes iAβ. Upon the completion of the treatment, the neuronal ISR is unconventionally re-elicited and the AβPP-independent C99/iAβ production pathway unconventionally reactivated. The accumulation of iAβ produced independently of AβPP resumes but from a low baseline. Before it reaches the T1 threshold, at a point indicated by suitable biomarkers, the composite therapy is repeated recurrently. The T1 threshold is not crossed, and the disease does not occur for the duration of the treatments. *Panel* (**B**): Recurrent administration of the composite therapy in the prevention of unconventional AD. The first round of the transient composite therapy is administered when iAβ produced independently of AβPP have already crossed the T1 threshold in all affected neurons, the AβPP-independent C99/iAβ production pathway has been rendered self-sustainable, some neurons have crossed the T2 threshold, and AD symptoms have already manifested. The concurrent transient inhibition of the neuronal ISR and activation of BACE1 and/or BACE2 disable the AβPP-independent C99/iAβ production pathway, stop the influx of iAβ generated independently of AβPP, and substantially deplete iAβ. When drugs are withdrawn, the neuronal ISR is unconventionally re-elicited and the AβPP-independent C99/iAβ production pathway unconventionally reactivated. The accumulation of iAβ resumes from a low baseline. Before it reaches the AD pathology-causing levels, at a point defined by the appropriate biomarkers, the composite therapy is re-administered and repeated recurrently as needed. The AD pathology-causing range of iAβ concentrations is not reached and the disease does not recur for the duration of the treatment.

The above strategy is illustrated in Figure 33. Panel A of Figure 33 depicts the implementation of the recurrent transient composite ISR inhibition/BACE activation therapy in the prevention of unconventional AD. In this panel, the neuronal integrated stress response has been unconventionally elicited and the AβPP-independent C99/iAβ production pathway unconventionally activated at the low levels of AβPP-derived iAβ. iAβ generated independently of AβPP has rapidly accumulated but at the time of the implementation of the therapy has not yet reached the T1 threshold. The concurrent administration of ISR inhibitors and BACE activators disables the AβPP-independent C99/iAβ production pathway and substantially depletes iAβ. Upon the completion of the treatment the neuronal ISR is unconventionally re-elicited and the AβPP-independent C99/iAβ production pathway unconventionally reactivated. The accumulation of iAβ produced independently of AβPP resumes but from a low baseline. Before it reaches the T1 threshold, at a point indicated by suitable biomarkers, the composite therapy is repeated recurrently. The T1 threshold is not crossed, and the disease does not occur for the duration of the treatments.

Panel B of Figure 33 depicts the implementation of the recurrent transient composite ISR inhibition/BACE activation therapy in the treatment of unconventional AD. The first round of the transient composite therapy is administered when iAβ produced independently of AβPP have already crossed the T1 threshold in all affected neurons, the AβPP-independent C99/iAβ production pathway has been rendered self-sustainable, some neurons have crossed the T2 threshold, and AD symptoms have already manifested. The concurrent transient inhibition of the neuronal ISR and activation of BACE1 and/or BACE2 disable the AβPP-independent C99/iAβ production pathway, stop the influx of iAβ generated independently of AβPP, and substantially deplete iAβ. When drugs are withdrawn, the neuronal ISR is unconventionally re-elicited and the AβPP-independent C99/iAβ production pathway unconventionally reactivated. The accumulation of iAβ resumes from a low baseline. Before it reaches the AD pathology-causing levels, at a point defined by the appropriate biomarkers, the composite therapy is re-administered and repeated recurrently as needed. The AD pathology-causing range of iAβ concentrations is not reached and the disease does not recur for the duration of the treatments. On the alternative approach, effectively converting unconventional AD into the conventional form of the disease, see Section 78 below.

## 64. On the Difficulties of Overcoming a Dogma: Ingrained Notions Are “Set in Stone”; Quantum Leaps as Breaks Through the Inertia of Preexisting Assumptions

The following sections outline the third version of the ACH2.0 theory of AD. The transition from the initial version of ACH2.0 to its second version was conceptually fundamental but logically trivial. Yet it took considerable time to make the leap from one to another. Initially, the ACH2.0 posited that AβPP-derived iAβ triggers AD by eliciting the neuronal integrated stress response. The ISR, in turn, enables the AβPP-independent C99/iAβ production pathway, and it is iAβ generated independently of AβPP, which drives AD pathology. Thus, the neuronal ISR initiates AD. It follows trivially that ANY stressor (other than AβPP-derived iAβ) capable of eliciting the neuronal ISR would trigger the disease (i.e., unconventional AD). The difficulty in formulating this concept was the ingrained notion that AD is triggered and driven by Aβ. An ingrained notion forms, apparently, a durable neural circuit, an equivalent of being “set in stone”, and the inertia of preexisting assumptions is very difficult to overcome; it involves the necessity to disassemble and replace a durable neural circuit. This is why quantum leaps, at least some of them, can be regarded as breaking through the inertia of the ingrained notion. This is also why newcomers to a field are disproportionately successful in executing quantum jumps: they are blessed by ignorance.

The breakthrough from Version Two to Version Three of the ACH2.0, which we are about to discuss in the following sections, is neither conceptually less fundamental nor less trivial than the shift between the first two versions. Moreover, the nature of the challenge of formulating it is the same as encountered in the first shift: the presumed role of Aβ in AD. The second shift stems from our understanding of effects of the integrated stress response. The ISR fundamentally restructures the translational (and transcriptional) landscape of the cell. This entails the global suppression of the cellular protein synthesis. Translation of the decisive bulk of cellular protein species is inhibited and there is no reason to assume that the constituents of the AβPP proteolytic pathway, i.e., AβPP, BACE enzymes, components of the gamma-secretase complex, and, consequently, Aβ are exempted from this process. Moreover, as discussed in the following sections below, this notion is supported by the empirical data. The difficulty in making this leap and the reason that it (again) took a considerable time to implement it is that it (again) downsizes the role of Aβ in AD, in fact, eliminates Aβ, which epitomizes AD in the previously ingrained notion, as the driver of the disease. However, both shifts, from the first version of the ACH2.0 to the second and from the second to the third are dictated by simple logic, and it is the logic, which we must follow because in the end it is always right.

## 65. Lessons from Transgenic Mouse Models: Production of Aβ Is Suppressed Following the Elicitation of the Neuronal ISR

As discussed above, mouse transgenic models overexpressing human AβPP develop the neuronal integrated stress response. It is elicited by AβPP-derived iAβ accumulated over the T1 threshold. The accumulation of iAβ in mice occurs via the same mechanisms that operate in humans, namely, the retention of intraneuronal Aβ produced by gamma cleavages on the intracellular membranes and the uptake of secreted extracellular Aβ. In transgenic mouse models both mechanisms are very efficient for the following reasons. First, these models express human AβPP mRNA (which is, however, ineligible for RNA-dependent amplification due to its modified 5′ terminus) from numerous, in some cases over a hundred, transgenes. Consequently, the rate of the exogenous production of Aβ is very high. Accordingly, the intraneuronally retained fraction of Aβ (produced by gamma cleavages on the intracellular membranes) translates into a greatly increased cellular concentration of iAβ. The increased rate of exogenous production of Aβ also elevates its extracellular pool; this significantly increases the rate of its cellular uptake. The rate of accumulation of exogenous AβPP-derived iAβ in mouse transgenic models is further augmented by the utilization of the FAD-causing mutations and AD-predisposing factors. Thus, human AβPP genes used in these models usually contain a combination of FAD-causing mutations. Transgenes encoding PSENs harboring FAD mutations and those encoding the ApoE4 isoform are also utilized in mouse models.

Cumulatively, these mutations significantly augment the rate of accumulation of exogenously produced AβPP-derived iAβ. Indeed, the mutations employed shift the production of Aβ toward its Aβ42 isoform, which, following its secretion is taken up twice as efficiently as other Aβ isoforms. Moreover, exogenously expressed ApoE4 is much more efficient in facilitating the cellular uptake of Aβ than other ApoE variants. In addition, some AβPP mutations usually utilized in human transgenes (such as Swedish AβPP mutation) as well as certain PSEN mutations substantially increase the fraction of gamma-cleavages occurring on the intraneuronal membranes; this increases the rate of the retention of exogenous AβPP-derived iAβ. The overall rate of accumulation of exogenous AβPP-derived iAβ is, therefore, considerable. Moreover, the utilization of exogenous Aβ42 reduces the extent of the T1 threshold, as discussed above. Consequently, iAβ reaches the T1 threshold and elicits the neuronal integrated stress response early in life. This is reflected in the occurrence of eIF2α phosphorylated at its Ser51, the elicitation of the neuronal ISR, and in the neuronal ISR-mediated mild neurodegeneration and cognitive impairments (caused by the global suppression of cellular protein synthesis) observed in these models. If exogenously produced iAβ were to continue accumulating at the same rate following the T1 crossing and elicitation of the neuronal ISR, a massive neurodegeneration and neuronal loss, caused by sheer volume of iAβ and its unavoidable aggregation, would be inevitable. This, however, does not happen. The most plausible explanation is that, following the elicitation of the neuronal ISR, the AβPP proteolytic pathway is suppressed as a part of the global suppression of cellular protein synthesis [10]. Since iAβ cannot be produced independently of AβPP in these models, its rate of accumulation is substantially reduced or even reversed, and the extents of both neurodegeneration and cognitive impairment do not increase significantly with time.

**Figure 34:**
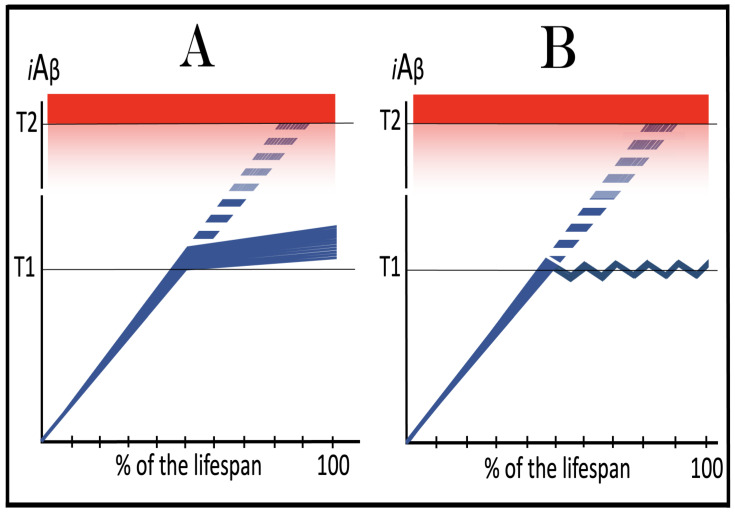
**The neuronal ISR inhibits the production of Aβ and suppresses the rate of iAβ accumulation: lessons from transgenic mouse models**. *Blue lines*: Intraneuronal Aβ, iAβ. ***T1***: Threshold of cellular concentration of iAβ triggering the activation of PKR and/or HRI kinases, phosphorylation of eIF2α, the elicitation of the neuronal ISR, the initiation of the AβPP-independent C99 production pathway, and the commencement of AD. ***T2***: Cellular concentration of iAβ triggering neuronal death via apoptosis or necroptosis. *Red box*: Apoptotic zone, defined as a range of cellular concentrations of iAβ within which cells neuronal cells committed apoptosis or necroptosis or are dead. *Pink box*: Range of AD pathology-causing concentrations of iAβ. *Broken blue lines*: the projected dynamics of accumulation of AβPP-derived iAβ occurring at the pre-T1 crossing rate; it would cause massive neurodegeneration and neuronal loss. *Panel* (**A**): Exogenously produced AβPP-derived iAβ rapidly accumulates and crosses the T1 threshold. PKR and/or HRI are activated, eIF2α is phosphorylated at its Ser51 residue, and the neuronal ISR is elicited. ISR-mediated suppression of cellular protein synthesis causes mild neurodegeneration and cognitive impairment. Were the accumulation of exogenous AβPP-derived iAβ to continue at the pre-T1 crossing rate (shown by broken blue lines), it would inevitably cause massive neuronal loss. Instead, the production of constituents of the AβPP proteolytic pathway is suppressed in the framework of the neuronal ISR together with that of the decisive bulk of cellular proteins. Consequently, the rate of accumulation of AβPP-derived iAβ is substantially reduced and the degree of the neuronal damage and of cognitive impairment does not change significantly with time. *Panel* (**B**): The rate of accumulation of AβPP-derived iAβ is reversed under ISR conditions, and its levels are decreasing. When iAβ levels reverse-cross the T1 threshold the ISR state is also reversed. This restores cellular protein synthesis and enables the production of the components of the AβPP proteolytic pathway and, consequently, the generation of iAβ. With its rate of accumulation restored, levels of iAβ increase. When they cross the T1 threshold, the neuronal ISR is re-elicited, the AβPP proteolytic pathway and the production of iAβ are suppressed, and the rate of accumulation of the latter is again reversed. The cycle repeats, and the levels of iAβ oscillate around the T1 threshold.

The above interpretation is illustrated in Figure 34. In Panel A of Figure 34, exogenously produced AβPP-derived iAβ accumulates at a considerable rate and reaches the T1 threshold relatively early in life. PKR and/or HRI are activated, eIF2α is phosphorylated at its Ser51 residue, and the neuronal integrated stress response is elicited. This causes mild neurodegeneration and cognitive impairment (both due to the ISR-mediated suppression of cellular protein synthesis). Were the accumulation of exogenous AβPP-derived iAβ to continue at the pre-T1 crossing rate (shown by broken blue lines), it would inevitably cause massive neurodegeneration, increasingly severe cognitive impairment and, upon reaching the T2 threshold, massive neuronal loss. Instead, the production of constituents of the AβPP proteolytic pathway is suppressed in the framework of the neuronal ISR together with that of the decisive bulk of cellular proteins. Consequently, the rate of accumulation of AβPP-derived iAβ is substantially reduced and the degree of the neuronal damage and of cognitive impairment does not change significantly with time.

In Panel B of Figure 34, following the T1 crossing and the elicitation of the neuronal integrated stress response, the rate of accumulation of AβPP-derived iAβ is reversed, and its levels are decreasing. This creates an interesting situation. When iAβ levels reverse-cross the T1 threshold the ISR state is abrogated and is no longer in effect. This restores a “normal” cellular protein synthesis and enables the production of the components of the AβPP proteolytic pathway and, consequently, the generation of iAβ. With its rate of accumulation restored, levels of iAβ increase. When they cross the T1 threshold, the neuronal integrated stress response is re-elicited, the AβPP proteolytic pathway and the production of iAβ are suppressed, and the rate of accumulation of the latter is again reversed. The cycle repeats, and the levels of iAβ oscillate around the T1 threshold.

## 66. The ACH2.0, Version Three: The Neuronal ISR Suppresses AβPP Proteolytic Pathway but Tau Is Translated from IRES; AD, in Its Both Conventional and Unconventional Forms, Is Driven by C99 Generated Independently of AβPP

**Figure 35:**
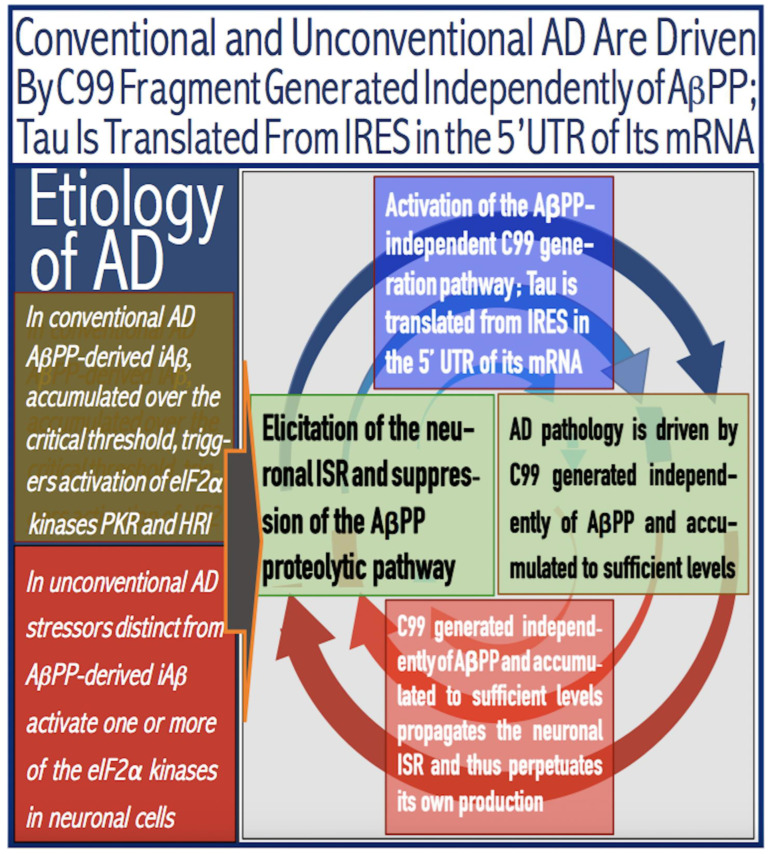
**Conventional and unconventional AD is driven by C99 generated independently of AβPP; Tau is translated from IRES in the 5′UTR of its mRNA**. *eIF2α*: eukaryotic translation initiation factor 2*α*. *PKR and HRI*: kinases that phosphorylate eIF2α at its Ser51 residue. *PACT*: PKR activator. *TNFα*: tumor necrosis factor *α*. *OMA1*: mitochondrial protease activated during mitochondrial dysfunction. *DELE1*: substrate of OMA1 protease. Cleavage of DELE1 leads to the activation of HRI. Phosphorylation of eIF2α elicits the integrated stress response, which, in turn, enables the generation of components essential for the activity of the AβPP-independent pathway of C99 and iAβ production. iAβ: intraneuronal Aβ. AβPP-derived iAβ accumulates physiologically via the importation of extracellular Aβ and the retention of iAβ produced by gamma-cleavages on intracellular membranes in an exceedingly slow process. In most individuals it does not reach the PKR- and/or HRI-activating, the neuronal ISR-eliciting threshold within their lifetimes and no conventional AD occurs. When this threshold is crossed, the neuronal ISR is elicited, and the AβPP-independent C99 production pathway activated, and conventional AD ensues. In unconventional AD stressors that are distinct from AβPP-derived iAβ and capable of activating one or more of eIF2α kinases trigger the elicitation of the neuronal ISR. In both cases the neuronal ISR provides components essential for the operation of the AβPP-independent C99 production pathway. Concurrently, under the neuronal ISR conditions the global cellular protein synthesis, including that of all components of the AβPP proteolytic pathway, is suppressed. Consequently, the accumulation of AβPP-derived iAβ is also suppressed. The production of C99 in the AβPP-independent pathway, on the other hand, proceeds unimpeded; due to the deficiency of gamma secretase it is processed no further. It rapidly accumulates, and it is C99 generated independently of AβPP, which drives AD pathology. Translation of tau protein, on the other hand, persists under ISR conditions because its mRNA contains an internal ribosomal entry site in the 5′UTR.

Version Three of the ACH2.0 posits that in both, conventional and unconventional forms of Alzheimer’s disease the neuronal integrated stress response-caused suppression of the global cellular protein synthesis includes all constituents of the AβPP proteolytic pathway, namely AβPP, BACE enzymes, components of the gamma-secretase complex, and, consequently, Aβ and iAβ. The picture that emerges in this paradigm is as follows. The neuronal integrated stress response is elicited either by AβPP-derived iAβ accumulated over the T1 threshold (conventional AD) or by a stressor distinct from iAβ at the levels of AβPP-derived iAβ well below the T1 threshold (unconventional AD). The neuronal ISR provides components essential for the operation of the AβPP-independent C99 generation pathway and this pathway is activated. Concurrently, under the neuronal ISR conditions the global cellular protein synthesis, including that of all components of the AβPP proteolytic pathway, is suppressed. The accumulation of AβPP-derived iAβ is either suppressed or reversed. The production of C99 in the AβPP-independent pathway, on the other hand, proceeds unimpeded. However, since the production of the components of the gamma-secretase complex is suppressed under the neuronal ISR conditions, C99 produced independently of AβPP is processed no further. It rapidly accumulates, and it is C99 generated independently of AβPP, which drives AD pathology. Accordingly, in this version of the ACH2.0 the pathway underlying AD pathology is designated as AβPP-independent C99 (not C99/iAβ) production pathway. The notion that C99 generated independently of AβPP drives AD pathology is strongly supported by the observation that in AD patients C99 accumulates in the disease-affected neurons but not in the regions of the brain not affected by the disease, and that the extent of its accumulation correlates with the severity of cognitive impairment [282,283]. The qualifications of C99 generated independently of AβPP to serve as the driver of AD pathology are discussed in the following section below. The present Version Three of the ACH 2.0 is illustrated schematically in Figure 35. It should be emphasized that in Version Three of the ACH2.0 the neuronal integrated stress response is sustained in the disease by C99 (generated independently of AβPP) at the levels above the ISR-eliciting T1 threshold. In the preceding versions of the ACH2.0 AβPP-derived iAβ was the only “conventional” neuronal ISR-eliciting stressor; in the present Version Three C99 is also the conventional ISR-eliciting stressor; not only does it drive AD pathology, it also propagates the neuronal ISR state.

Thus, in AD-affected neurons the driver of AD pathology, C99, is produced in a neuronal ISR-compatible process. But what happens with the other major constituent of AD pathology, namely tau protein? It is, evidently, produced and accumulates in the disease. This implies that its production is not suppressed by the neuronal ISR and, therefore, must occur in an ISR-compatible manner. This is indeed the case. Whereas the production of Aβ (and of the bulk of cellular proteins) is suppressed in AD-affected neurons under the ISR conditions, the production of tau protein continues because the human tau mRNA contains an internal ribosomal entry site (IRES) in its 5′ untranslated region [284]. Translation from this site is unaffected by the ISR and results in the complete polypeptide.

## 67. C99 Generated Independently of AβPP Meets Qualification Criteria for the Driver of AD: C99 as “Substance X” Predicted in [2]

A possibility that an agent other than Aβ or iAβ, dubbed “substance X”, can drive AD pathology was entertained in our previous study [2]. The study [2] considered it a given that conventional AD is triggered by iAβ accumulated over the PKR and/or HRI activating threshold. Consequently, eIF2α is phosphorylated and the neuronal integrated stress response elicited. A small subset of cellular proteins, newly expressed under the ISR conditions, contains components essential for the operation of the substance X-generating pathway. The pathway is activated, and its product, substance X, accumulates and drives the disease. This study [2] concluded that in order to qualify as the driver of AD, such an agent (substance X) must meet two requirements. First, it has to be sufficiently toxic to be capable of promoting AD pathology. Second, it has to be capable of maintaining the neuronal integrated response state and, consequently, of sustaining the operation of the pathway of its own production. By positing that the disease is driven by C99 generated independently of AβPP, the present Perspective equates the C99 fragment with substance X. But does C99 meet the stated above qualification criteria for the driver of AD?

The answer to the above question is, apparently, affirmative. Indeed, the C99 fragment is capable of triggering the neuronal integrated stress response in multiple ways, including, for example, the ER stress [285] or causing mitochondrial distress [286]. The former entails the activation of the PERK kinase and the latter results, via the OMA1 to DELE1 to HRI signaling pathway, in the activation of HRI kinase. In both cases, eIF2α is phosphorylated at its Ser51 residue and the neuronal ISR is elicited. With regard to its toxicity, C99 was shown to interfere with lipid metabolism and to cause the loss of lipid homeostasis. This occurs through the interaction of C99 with MAM (mitochondria-associated ER membranes) [287]. Intraneuronal accumulation of C99 was also shown to trigger endosomal/autophagic/lysosomal (EAL) dysfunction, which is a recognized neuropathological feature of AD [288]. In this case, the deleterious effect of the C99 fragment apparently results from its aggregation within EAL-vesicle membranes; this leads to disrupted lysosomal proteolysis and to autophagic impairment. Both of these conditions, if sustained, are capable of initiating and maintaining cascades of cellular pathology. Thus, the C99 fragment apparently possesses the toxicity required to drive AD pathology and is also capable of propagating the neuronal ISR state, consequently perpetuating the AβPP-independent pathway of its own production. Thus, it meets qualification criteria for the driver of AD.

## 68. Validation (12): Inhibition of Gamma-Secretase in Human Neuronal Cell-Based AD Model Would Not Prevent or Abrogate Cellular AD Pathology

The notion that AD is driven by C99 generated independently from AβPP can potentially be validated in a human neuronal cell-based AD model. In this application, the sustained neuronal integrated stress response can be elicited unconventionally, by a variety of stressors other than iAβ (e.g., mitochondrial distress). The utilization of unconventional, rather than conventional (iAβ-triggered) AD model is important in order to exclude the possibility that cellular AD pathology, if observed, is caused by exogenously produced iAβ. It can be anticipated that the neuronal ISR would activate the AβPP-independent C99 generation pathway and that, with a sufficient duration of the presence of the unconventional stressor, this pathway would be rendered self-sustainable. It is expected that, as described above, this would lead to the development of cellular AD pathology and manifest in the formation of NFTs. If cellular AD pathology in such an experiment is indeed driven by C99, the inclusion of gamma-secretase inhibitors would not prevent AD pathology, and the addition of inhibitors when NFTs have already formed would not abrogate it. This is in contrast to a possibility that C99 produced independently of AβPP is processed into iAβ, which actually drives AD pathology. In such a case, the inclusion of gamma-secretase inhibitors would prevent AD pathology (and the formation of NFTs) or, at least, modulate it.

## 69. Dynamics of Conventional and Unconventional AD in the ACH2.0 Version Three Perspective

From the perspective of the ACH2.0, Version Three, the dynamics of AD in its conventional form is defined by two sets of factors. One is AβPP-derived iAβ, more precisely, the rate of its accumulation, and the extent of the T1 threshold. Its role in the version under discussion is, in fact, identical to that in the preceding versions of ACH2.0. The occurrence of AD is defined by AβPP-derived iAβ reaching and crossing the T1 threshold; if the latter is not crossed within the lifetime of an individual, the former does not occur. If the disease does occur, the timing of its commencement is directly proportional to the extent of the T1 threshold and inversely proportional to the rate of accumulation of AβPP-derived iAβ. The relationships between the rate of accumulation of AβPP-derived iAβ, the extent of the T1 threshold, and the occurrence and timing of conventional AD are discussed in-depth in Section 27, Section 28, Section 29, Section 30 and Section 31 above. All conclusions reached in those sections apply fully in Version Three of the ACH2.0.

Another set of factors determining the dynamics of conventional AD is the rate of accumulation of C99 produced independently of AβPP and the extent of the T2 threshold. In this respect, in Version Three of the ACH2.0, C99 generated in the AβPP-independent pathway plays the same role as iAβ, presumably produced independently of AβPP, does in the preceding versions of ACH2.0. The rate of the progression of AD is directly proportional to the rate of accumulation of C99 produced independently of AβPP, whereas the duration of the disease is inversely proportional to the rate of accumulation of C99 generated in the AβPP-independent pathway and directly proportional to the extent of the T2 threshold. This dynamic is examined (for iAβ produced in the AβPP-independent pathway as the driver of AD) in Section 49 above. The conclusions arrived at in that section are conceptually fully applicable in Version Three of the ACH2.0 as well.

**Figure 36:**
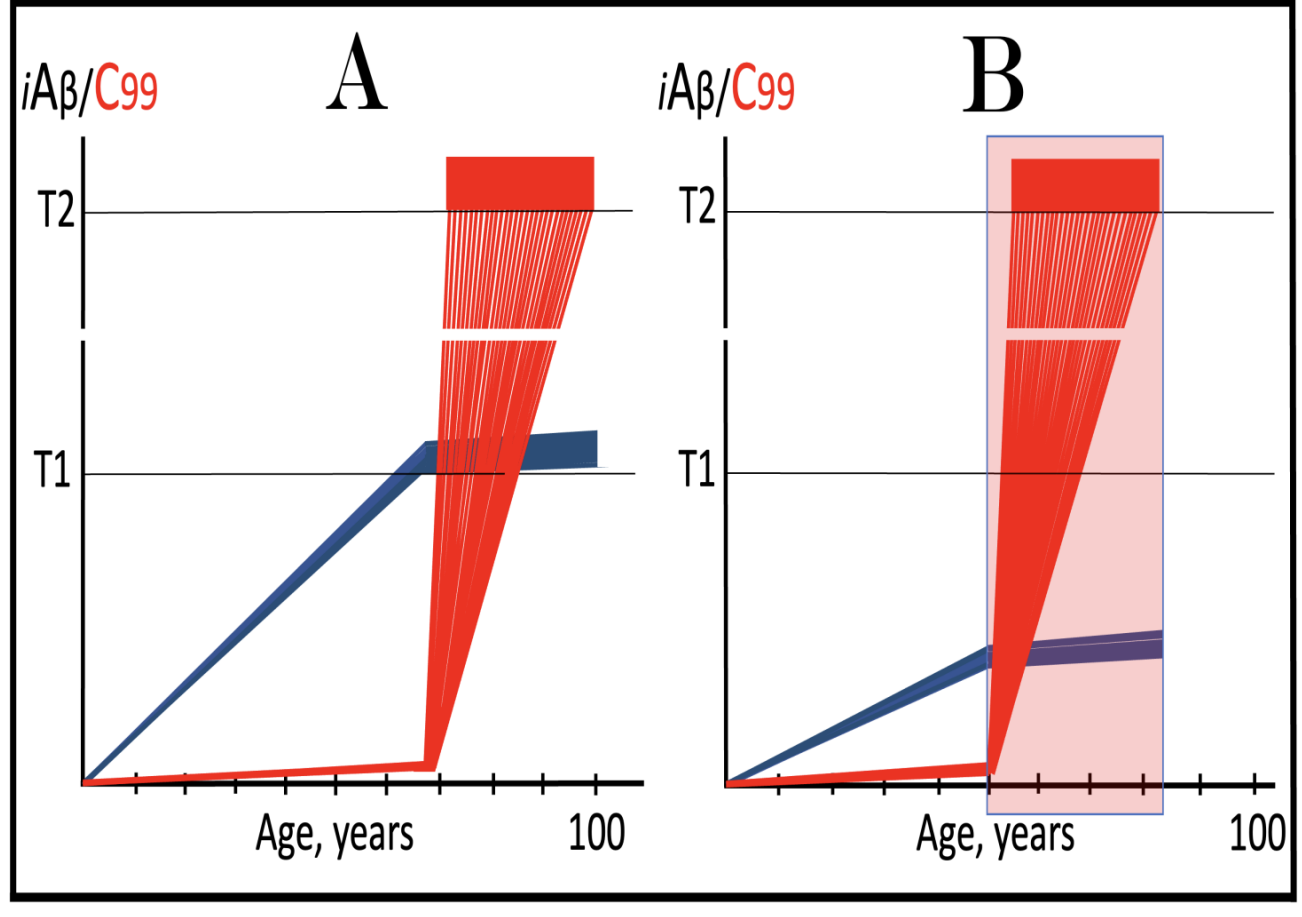
**Dynamics of conventional and unconventional AD in the ACH2.0 Version Three perspective**. *Blue lines*: Intraneuronal Aβ, iAβ. *Red lines*: The C99 fragment of AβPP. ***T1***: Threshold of cellular concentration of iAβ triggering the activation of PKR and/or HRI kinases, phosphorylation of eIF2α, the elicitation of the neuronal ISR, the initiation of the AβPP-independent C99 production pathway, and the commencement of AD. ***T2***: Cellular concentration of iAβ triggering neuronal death via apoptosis or necroptosis. *Red box*: Apoptotic zone, defined as a range of cellular concentrations of iAβ within which cells neuronal cells committed apoptosis or necroptosis or are dead. *Pink box*: The duration of the presence of the unconventional stressor capable of activating one or more of eIF2α kinases and triggering the elicitation of the neuronal ISR. *Panel* (**A**): Dynamics of conventional AD. AβPP-derived iAβ accumulates physiologically via mechanisms discussed in the main text. When it crosses the T1 threshold, PKR and/or HRI are activated, eIF2α is phosphorylated at its Ser51 residue, and the neuronal ISR is elicited. Global cellular protein synthesis, including all constituents of the AβPP proteolytic pathway: AβPP, BACE enzymes, components of the gamma-secretase complex, and, consequently Aβ, is severely suppressed. The influx of AβPP-derived iAβ is inhibited, and the rate of its accumulation is reduced. Concurrently, the neuronal ISR enables the production of components essential for the operation of the AβPP-independent C99 generation pathway. The pathway is self-sustainable from the instance of its activation because the ISR state is initially sustained by over-T1 AβPP-derived iAβ and eventually by C99 after it crosses the T1 threshold. C99 produced independently of AβPP drives AD pathology; when it crosses the T2 threshold the disease enters its end stage. *Panel* (**B**): Dynamics of unconventional AD. The neuronal ISR is unconventionally elicited, and the AβPP-independent C99 generation pathway is unconventionally activated at the levels of AβPP-derived iAβ well below the T1 threshold. Under the neuronal ISR conditions the AβPP proteolytic pathway is suppressed and the rate of accumulation of AβPP-derived iAβ is substantially reduced. On the other hand, C99 generated independently of AβPP rapidly accumulates and crosses the T1 threshold (provided the unconventional stressor persists for a sufficient duration). Following the T1 crossing, the AβPP-independent C99 production pathway is rendered self-sustainable, and AD commences and progresses. When the T2 threshold is reached in a sufficient neuronal fraction, the disease enters its end stage.

The dynamics of conventional AD in terms of the accumulation of AβPP-derived iAβ and of C99 generated independently of AβPP is illustrated concisely (it shows neither the effects of the variable rates of accumulation of both nor the outcomes of the variable extents of the T1 and T2 thresholds) in Panel A of Figure 36. In the first phase of AD AβPP-derived iAβ accumulates via the retention of a fraction of Aβ resulting from gamma-cleavages occurring on the intracellular membranes within various cellular organelles and the cellular uptake of a fraction of secreted extracellular Aβ (discussed in Section 24 above). When its levels reach and cross the T1 threshold, PKR and/or HRI are activated, eIF2α is phosphorylated at its Ser51 residue, and the neuronal integrated stress response is elicited. This results in two concurrently occurring developments. First, global cellular protein synthesis is severely suppressed. This includes all constituents of the AβPP proteolytic pathway: AβPP, BACE enzymes, components of the gamma-secretase complex, and, consequently, Aβ. The influx of AβPP-derived iAβ is inhibited at both sources (suppressed production of Aβ, suppressed retention; diminishing extracellular pool of Aβ, diminishing uptake), and the rate of its accumulation is reduced. In the second, and concurrent, development, the neuronal ISR enables the production of components essential for the operation of the AβPP-independent C99 generation pathway. The pathway is activated and is self-sustainable from the instance of its activation because the ISR state is initially sustained by over-T1 AβPP-derived iAβ and eventually by C99 after it crosses the T1 threshold. C99 produced independently of AβPP drives AD pathology; when it crosses the T2 threshold the disease enters its end stage. Thus, in Version Three of the ACH2.0 the role of Aβ, more precisely of iAβ, is limited to triggering the neuronal integrated stress response, and even this limited function is restricted to the conventional form of AD; it plays little or no role in the progression of AD. It also retains its causative role in AACD. If the extent of the T^0^ threshold were below that of the T1 threshold, AACD would commence upon the crossing of the former and morph into AD when the latter is crossed.

The dynamics of unconventional AD is different. It is less complex as far as iAβ is concerned. The disease is triggered at the levels of AβPP-derived iAβ below the T1 threshold, and therefore iAβ plays no substantive role in unconventional AD. The dynamics is, however, more complex due to the potential variability of the timing and, especially, the duration of the unconventionally elicited neuronal integrated stress response. The former (timing) is defined by the occurrence of an unconventional stressor capable of eliciting the neuronal ISR (any stressor distinct from iAβ and C99). The latter, on the other hand, is determined by the duration of the presence of the unconventional stressor. In unconventional AD the neuronal ISR is unconventionally elicited and, accordingly, the AβPP-independent C99 generation pathway is unconventionally activated. C99, produced independently of AβPP, rapidly accumulates. If the duration of the activity of the AβPP-independent C99 generation pathway were insufficient for the T1 crossing by C99, the former would be only transient and not necessarily result in AD (further discussed below). If and when C99 crosses the T1 threshold, the pathway becomes self-sustainable, and AD commences and progresses; the T2 crossing denotes the entrance of the disease into the end stage. The rate of its progression is directly proportional to the rate of accumulation of C99 produced independently of AβPP, and its duration is directly proportional to the extent of the T2 threshold and inversely proportional to the rate of accumulation of C99 generated in the AβPP-independent pathway.

As stated above, the above-invoked complexity is due in part to the possibility that the presence of the unconventional stressor can be transient. Its withdrawal after the T1 crossing by C99 would be inconsequential: the neuronal ISR state would be sustained by the product of the AβPP-independent C99 production pathway, i.e., the pathway would be self-sustainable, and the disease would proceed unimpeded. On the other hand, if the unconventional stressor is withdrawn prior to the crossing of the T1 threshold by C99, the ISR state would be reversed, and the AβPP-independent C99 generation pathway would be rendered inoperative. Thus, transient unconventional activation of the AβPP-independent C99 production pathway could be benign. If, however, it occurs recurrently, every time the accumulation of C99 would resume from an elevated threshold until, eventually it crosses the T1 threshold, the AβPP-independent C99 generation pathway becomes self-sustainable, and the disease commences. The possible outcomes of transient unconventional activation of the AβPP-independent C99 production pathway were discussed above (Section 52 and Section 53) with an assumption that AD is driven by iAβ generated independently of AβPP. Nevertheless, conceptually, the conclusions reached above apply fully in Version Three of the ACH2.0 as well.

The dynamics of unconventional AD in terms of the accumulation of C99 generated independently of AβPP is presented in Panel B of Figure 36. In this panel, the neuronal integrated stress response is unconventionally elicited, and the AβPP-independent C99 generation pathway is unconventionally activated at the levels of AβPP-derived iAβ well below the T1 threshold. Under the neuronal ISR conditions the AβPP proteolytic pathway is suppressed and the rate of accumulation of AβPP-derived iAβ is substantially reduced. On the other hand, C99 generated independently of AβPP rapidly accumulates and crosses the T1 threshold (it is assumed in this panel that the duration of the presence of the unconventional stressor is sufficient for the T1 crossing to occur in all affected neurons). Following the T1 crossing, the AβPP-independent C99 production pathway is rendered self-sustainable, and AD commences and progresses. When the T2 threshold is reached in a sufficient neuronal fraction, the disease enters its end stage. Thus, in unconventional AD, Aβ and iAβ play little or no role at all in both triggering the disease and driving AD pathology.

## 70. ACH-Based AD Drugs from the Perspective of the ACH2.0, Version Three

ACH-based AD drugs are agents designed under the ACH guidance with the purpose of reducing the levels of extracellular Aβ. This group of drugs includes Aβ-specific antibodies designed to sequester extracellular Aβ and BACE1 inhibitors intended to suppress the production of Aβ and, consequently, its secretion and extracellular concentration. Initially, gamma-secretase inhibitors were also part of this group of drugs, but their utilization was ruled out due to severe adverse effects. Instead, the effort switched to the development of gamma-secretase modulators, agents that shift the position of gamma-cleavage within the C99 fragment toward shorter, more benign Aβ species. Above (Section 36 and Section 37), we reasoned that, in the initial version of the ACH2.0, ACH-based drugs are legitimate (albeit not very efficient) conventional AD-preventive agents. This is because by reducing the extracellular pool of Aβ they reduce the rate of its cellular uptake, and by suppressing its production (with BACE1 inhibitors) they reduce both the cellular uptake of Aβ (due to its reduced extracellular pool) and its retention (less produced, less retained). As a result, the rate of accumulation of AβPP-derived iAβ is decreased, the T1 crossing is either delayed or prevented, and the occurrence of AD is either delayed or precluded. As for gamma-secretase modulators, the more benign iAβ species would elevate the T1 threshold (by the same logic that more toxic Aβ42 would lower it; see Section 33 above), and the T1 threshold crossing also be either delayed or prevented.

The above conclusions remain fully in effect in Version Three of the ACH2.0 but only for the conventional form of AD and only for its prevention but not the treatment. In conventional AD the neuronal integrated stress response can be elicited, and the disease triggered only by AβPP-derived iAβ accumulated above the T1 threshold. If no T1 crossing occurs, neither does the disease; if the T1 crossing is delayed, the occurrence of AD is also delayed. The preventive prospect of ACH-based drugs in conventional AD, however, is their only potential utility in Version Three of the ACH2.0. Indeed, in unconventional AD these drugs are inapplicable preventively because stressors other than AβPP-derived iAβ trigger the disease. As for the treatment of AD, ACH-based drugs are inapplicable in both, conventional and unconventional forms of the disease because in both AD pathology is driven by self-sustainable AβPP-independent C99 production pathway. Aβ and iAβ play no role at this stage and applying ACH-based drugs would amount to attacking an imaginary target. This applies also to modulators of gamma-secretase. In the initial version of the ACH2.0, such agents could potentially confer benefits in the treatment of AD by elevating the T2 threshold and thus slowing down the progression of the disease. This is principally not the case in Version Three of ACH2.0, where the AβPP proteolytic pathway is suppressed in symptomatic AD and there is little or no gamma-secretase to modulate. On the other hand, as discussed in the following sections below, ACH2.0-based drugs retain their therapeutic potential in both the prevention and treatment of both conventional and unconventional forms of the disease in terms of the ACH2.0, Version Three.

## 71. ISR Inhibitors in the Prevention of Conventional AD: The ACH2.0, Version Three Perspective

**Figure 37:**
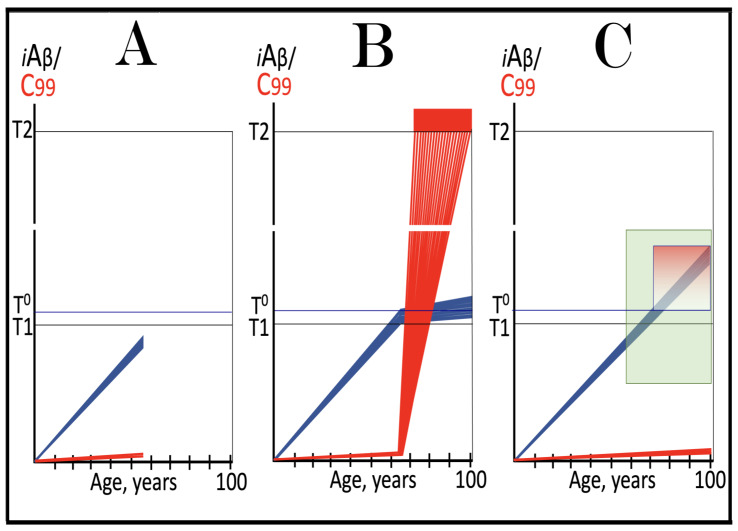
**ISR inhibitors in the prevention of conventional AD: the ACH2.0**, **Version Three perspective**. *Blue lines*: Intraneuronal Aβ, iAβ. *Red lines*: The C99 fragment of AβPP. ***T*^0^**: Threshold of cellular concentration of AβPP-derived iAβ triggering the neuronal damage, which manifests as AACD. ***T1***: Threshold of cellular concentration of iAβ triggering the activation of PKR and/or HRI kinases, phosphorylation of eIF2α, the elicitation of the neuronal ISR, the initiation of the AβPP-independent C99 production pathway, and the commencement of AD; its extent is the only variable in the present figure. ***T2***: Cellular concentration of iAβ triggering neuronal death via apoptosis or necroptosis. *Red box*: Apoptotic zone, defined as a range of cellular concentrations of iAβ within which cells neuronal cells committed apoptosis or necroptosis or are dead. *Pink box*: The range of cellular concentrations of AβPP-derived iAβ concentrations between the T^0^ and the highest extent reached by AβPP-derived iAβ or the T1 threshold that support the progression of AACD. The condition commences with the crossing of the T^0^ threshold and morphs into AD with the T1 crossing and elicitation of the ISR; usually it can occur only if the extent of the T^0^ threshold is smaller than that of the T1. *Green box*: Duration of the treatment with the ISR-inhibiting drug. *Panel* (**A**): The initial state of the levels of AβPP-derived iAβ in the neurons of the healthy individual who would develop AD if untreated. AβPP-derived iAβ has been accumulating but its levels are below the T1 threshold. *Panel* (**B**): AβPP-derived iAβ crosses the T1 threshold, PKR and/or HRI are activated, eIF2α is phosphorylated at its Ser51 residue, and the neuronal ISR is elicited. Under the ISR conditions the global cellular protein synthesis, including that of the constituents of the AβPP proteolytic pathway, is suppressed and the rate of accumulation of AβPP-derived iAβ is substantially reduced. Concurrently, the neuronal ISR enables the production of components essential for the operation of the AβPP-independent C99 production pathway, and the pathway is activated. C99 generated independently of AβPP rapidly accumulates and drives the progression of AD pathology. *Panel* (**C**): The evolution of the initial state in the individual treated with the ISR-inhibiting drug. AβPP-derived iAβ accumulates and crosses the T1 threshold. The drug precludes the elicitation of the neuronal ISR, and the AβPP-independent C99 production pathway remains inoperative. The accumulation of AβPP-derived iAβ continues at the pre-T1 crossing rate; it does not reach the AD pathology-causing levels for the duration of the treatment. On the other hand, since the ISR is suppressed, if AβPP-derived iAβ crosses the T^0^ threshold, even if it is above the T1 threshold, AACD would commence and persist for the remaining portion of the lifetime; it would be unaffected by the treatment.

The elicitation of the neuronal integrated stress response remains the AD-triggering event in Version Three of the ACH2.0. It follows that the prevention of the neuronal ISR by a therapeutic intervention would potentially prevent the disease for the duration of the treatment. This notion, in its application to conventional AD, is considered in Figure 37. Panel A of Figure 37 depicts the initial state of the levels of AβPP-derived iAβ at the time of the commencement of the treatment. It has been accumulating via both the cellular uptake of a fraction of secreted extracellular Aβ and the retention of iAβ produced by gamma-cleavages of C99 on the intraneuronal membranes within various cellular organelles, but its levels have not yet reached the T1 threshold. Panel B of Figure 37 illustrates the evolution of the initial state in the absence of the ISR-inhibiting treatment. The accumulation of AβPP-derived iAβ continues unhindered and it crosses the T1 threshold. PKR and/or HRI are activated, eIF2α is phosphorylated at its Ser51 residue, and the neuronal integrated stress response is elicited. Under the ISR conditions the global cellular protein synthesis, including that of the constituents of the AβPP proteolytic pathway, is suppressed and the rate of accumulation of AβPP-derived iAβ is substantially reduced. Concurrently, the neuronal ISR enables the production of components essential for the operation of the AβPP-independent C99 production pathway, and the pathway is activated. C99 generated independently of AβPP rapidly accumulates and drives the progression of AD pathology. Panel C of Figure 37 shows the evolution of the initial state in the individual treated with the ISR-inhibiting drug. AβPP-derived iAβ continues to accumulate and crosses the T1 threshold. The drug precludes the elicitation of the neuronal integrated stress response, and the AβPP-independent C99 production pathway remains inoperative. The accumulation of AβPP-derived iAβ continues at the pre-T1 crossing rate; it does not, however, reach the AD pathology-causing levels. Thus, ISR inhibitors administered prior to the T1 crossing by AβPP-derived iAβ can prevent the occurrence of conventional AD for the duration of the treatment. On the other hand, if AβPP-derived iAβ crosses the T^0^ threshold, even if it is above the T1 threshold, as shown in Figure 37, AACD would commence and persist for the remaining portion of the lifetime; it would be unaffected by the treatment. Importantly, the treatment has to be administered for the lifetime. If the drug were withdrawn, the neuronal ISR would be elicited, and conventional AD would commence.

## 72. ISR Inhibitors in the Treatment of Conventional AD: The ACH2.0, Version Three Perspective

**Figure 38:**
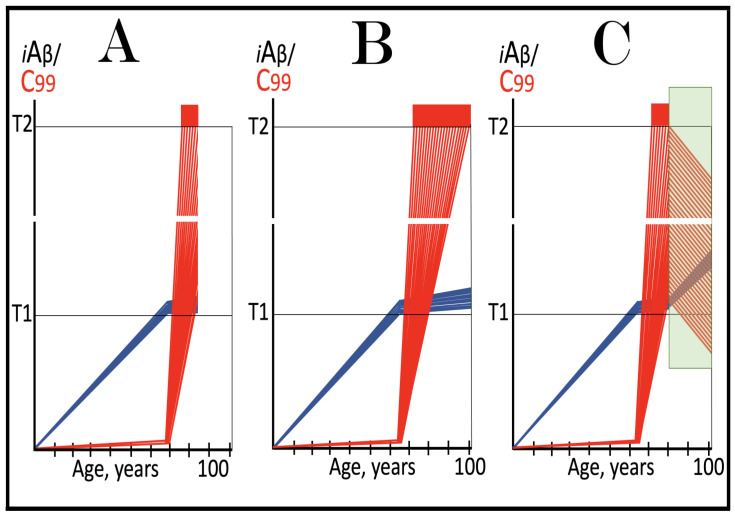
**ISR inhibitors in the treatment of conventional AD: the ACH2.0**, **Version Three perspective**. *Blue lines*: Intraneuronal Aβ, iAβ. *Red lines*: The C99 fragment of AβPP. ***T1***: Threshold of cellular concentration of iAβ triggering the activation of PKR and/or HRI kinases, phosphorylation of eIF2α, the elicitation of the neuronal ISR, the initiation of the AβPP-independent C99 production pathway, and the commencement of AD; its extent is the only variable in the present figure. ***T2***: Cellular concentration of iAβ triggering neuronal death via apoptosis or necroptosis. *Red box*: Apoptotic zone, defined as a range of cellular concentrations of iAβ within which cells neuronal cells committed apoptosis or necroptosis or are dead. *Green box*: Duration of the treatment with the ISR-inhibiting drug. *Panel* (**A**): The initial state of the levels of AβPP-derived iAβ and C99 in the neurons of the AD patient. AβPP-derived iAβ has crossed the T1 threshold in all affected neurons, and the neuronal ISR has been elicited. The AβPP proteolytic pathway has been suppressed and the rate of accumulation of AβPP-derived iAβ substantially reduced. Concurrently, the AβPP-independent C99 generation pathway has been activated. C99 accumulated and reached the T2 threshold in a fraction of the neurons, and AD symptoms manifested. *Panel* (**B**): The evolution of the initial state in the untreated patient. C99 produced independently of AβPP continues to accumulate and reaches the T2 threshold in a sufficient fraction of the neurons, and the disease enters its end stage. *Panel* (**C**): The evolution of the initial state in the AD patient treated with the ISR-inhibiting drug. The ISR state is reversed. The activity of the AβPP proteolytic pathway is restored, and the accumulation of AβPP-derived iAβ resumes at the pre-T1 crossing rate. It would not, however, reach the AD pathology-causing levels. On the other hand, the operation of the AβPP-independent C99 generation pathway ceases. Levels of C99, processed by now available gamma-secretase decline, and the progression of AD is arrested for the duration of the treatment.

In the ACH2.0, Version Three, AD is driven by C99 generated in the AβPP-independent pathway. The operation of this pathway is contingent on the sustained occurrence of the neuronal ISR state. The inhibition of the latter would deprive the former of its essential components; its operation would cease, and the progression of AD potentially arrested. This scenario is considered in Figure 38. Panel A of Figure 38 shows the initial state of the levels of AβPP-derived iAβ and C99 generated independently of AβPP at the commencement of the treatment. In this panel, the levels of AβPP-derived iAβ have crossed the T1 threshold in all affected neurons of the AD patient, and the neuronal integrated stress response has been elicited. The AβPP proteolytic pathway has been suppressed and the rate of accumulation of AβPP-derived iAβ substantially reduced. Concurrently, the AβPP-independent C99 generation pathway has been activated. C99 accumulated, reached the T2 threshold in a fraction of the neurons, and AD symptoms manifested. Panel B of Figure 38 depicts the evolution of the initial state in the untreated patient. C99 produced independently of AβPP continues to accumulate, reaches the T2 threshold in a sufficient fraction of the neurons, and the disease enters its end stage. Panel C of Figure 38 presents the evolution of the initial state in the AD patient treated with the ISR-inhibiting drug. The ISR state is reversed. The activity of the AβPP proteolytic pathway is restored, and the accumulation of AβPP-derived iAβ resumes at the pre-T1 crossing rate. It would not, however, reach the AD pathology-causing levels. On the other hand, the operation of the AβPP-independent C99 generation pathway ceases. Levels of C99, processed by now available gamma-secretase decline, and the progression of AD is arrested for the duration of the treatment. The processing of C99 generated in the AβPP-independent pathway by gamma-secretase would produce a temporary peak of Aβ, but without the influx of C99 produced independently of AβPP this peak would dissipate. Thus, the long-term treatment with ISR inhibitors can stop the progression of conventional AD.

## 73. ISR Inhibitors in the Prevention and Treatment of Unconventional AD: The ACH2.0, Version Three Perspective

In unconventional AD the neuronal integrated stress response is elicited by a variety of stressors distinct from iAβ or C99. This occurs at the levels of AβPP-derived iAβ below the T1 threshold (the T1 crossing triggers conventionally the neuronal ISR, and the appearance of unconventional ISR-eliciting stressors at this point would be redundant and inconsequential). The unconventionally elicited neuronal ISR suppresses the AβPP proteolytic pathway and substantially reduces the rate of accumulation of AβPP-derived iAβ. At the same time, it activates the AβPP-independent C99 generation pathway. C99 produced independently of AβPP rapidly accumulates, and AD commences when it crosses the T1 threshold. This is because prior to the T1 crossing, the AβPP-independent C99 production pathway is not self-sustainable, i.e., if the unconventional stressor were withdrawn, the operation of the pathway would cease. Thus, if the neuronal ISR is inhibited prior to the T1 crossing by C99 produced independently of AβPP, the AβPP-independent C99 generation pathway would be rendered inoperative, C99 produced independently of AβPP would not cross the T1 threshold, and AD would be prevented.

**Figure 39:**
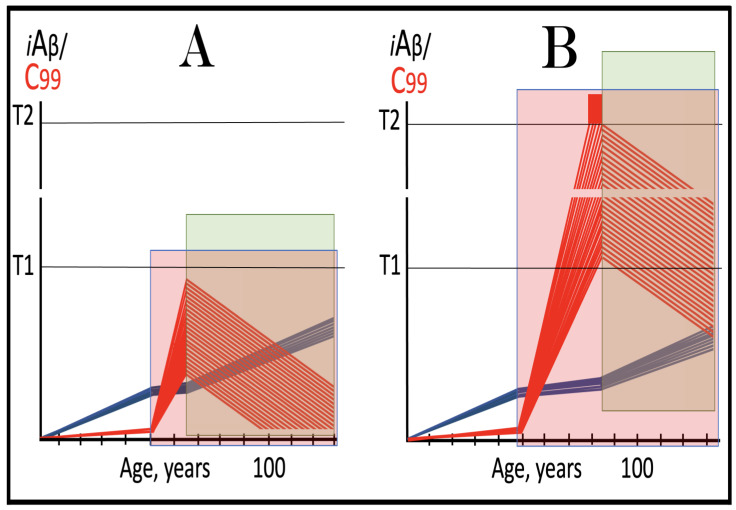
**ISR inhibitors in the prevention and treatment of unconventional AD: the ACH2.0**, **Version Three perspective**. *Blue lines*: Intraneuronal Aβ, iAβ. *Red lines*: The C99 fragment of AβPP. ***T1***: Threshold of cellular concentration of iAβ triggering the activation of PKR and/or HRI kinases, phosphorylation of eIF2α, the elicitation of the neuronal ISR, the initiation of the AβPP-independent C99 production pathway, and the commencement of AD; its extent is the only variable in the present figure. ***T2***: Cellular concentration of iAβ triggering neuronal death via apoptosis or necroptosis. *Red box*: Apoptotic zone, defined as a range of cellular concentrations of iAβ within which cells neuronal cells committed apoptosis or necroptosis or are dead. *Pink boxes*: The duration of the presence of the unconventional stressor capable of activating one or more of eIF2α kinases and triggering the elicitation of the neuronal ISR. *Green box*: Duration of the treatment with the ISR-inhibiting drug. *Panel* (**A**): ISR inhibitors in the prevention of unconventional AD. Following the occurrence of the unconventional stressor, the neuronal ISR has been unconventionally elicited and the AβPP-independent C99 production pathway unconventionally activated. The neuronal ISR suppresses the AβPP proteolytic pathway and reduces the rate of accumulation of AβPP-derived iAβ. Concurrently, C99 produced independently of AβPP rapidly accumulates but is still below the T1 threshold. The drug reverses the ISR state, restores the activity of the AβPP proteolytic pathway, and the accumulation of the AβPP-derived iAβ resumes at its pre-ISR rate. It also disables the production of C99 in the AβPP-independent pathway, and its levels decline for the duration of the treatment. *Panel* (**B**): ISR inhibitors in the treatment of unconventional AD. At the time of the drug’s administration, C99 produced independently of AβPP has crossed the T2 threshold in a fraction of the neurons, and AD symptoms have manifested. The drug reverses the neuronal ISR state, enables the activity of the AβPP proteolytic pathway, and the accumulation of AβPP-derived iAβ resumes at the pre-ISR rate. It also disables the AβPP-independent C99 production pathway. The levels of C99 generated independently of AβPP are declining and the progression of the disease is stopped for the duration of the treatment.

This scenario is illustrated in Panel A of Figure 39. In this panel, the neuronal integrated stress response has been unconventionally elicited and the AβPP-independent C99 generation pathway unconventionally activated at the levels of AβPP-derived iAβ well below the T1 threshold. The neuronal ISR suppresses the AβPP proteolytic pathway and reduces the rate of accumulation of AβPP-derived iAβ. At the same time C99 produced independently of AβPP rapidly accumulates but at the time of the commencement of treatment with an ISR-inhibiting drug it is still below the T1 threshold. The drug reverses the ISR state, restores the activity of the AβPP proteolytic pathway, and the accumulation of the AβPP-derived iAβ resumes at its initial (pre-ISR) rate. It also deprives the AβPP-independent C99 generation pathway of its essential components, and the production of C99 in the AβPP-independent manner ceases. Its levels decline and would not reach the T1 threshold and AD would be prevented for the duration of the treatment. AβPP-derived iAβ may cross the T1 threshold, but in the presence of the ISR inhibitor this would be inconsequential, as described in the preceding sections.

Panel B of Figure 39 illustrates another scenario where the unconventional elicitation of the neuronal integrated stress response suppressed the AβPP proteolytic pathway, reduced the rate of accumulation of AβPP-derived iAβ, and activated the AβPP-independent C99 production pathway. In this scenario C99 generated in the unconventionally activated AβPP-independent pathway accumulates over the T1 threshold. The pathway is rendered self-sustainable, and AD commences. At the time of the commencement of treatment with the ISR inhibitor, C99 produced independently of AβPP has crossed the T2 threshold in a fraction of the neurons, and AD symptoms have manifested. The reversal of the neuronal ISR state enables the activity of the AβPP proteolytic pathway, and the accumulation of AβPP-derived iAβ resumes at the pre-ISR rate. It also disables the AβPP-independent C99 production pathway. The levels of C99 generated independently of AβPP are declining and the progression of the disease is arrested for the duration of the treatment. AβPP-derived iAβ may potentially cross the T1 threshold but no AD would be triggered in the presence of the drug. Thus, the long-term inhibition of the neuronal ISR may stop the progression of unconventional AD.

## 74. BACE1 and/or BACE2 Activators in the Prevention of Conventional AD and in the Prevention and Treatment of AACD: The Perspective of the ACH2.0, Version Three

The present section considers a scenario where the levels of AβPP-derived iAβ in the neurons of an individual are either below or above the AACD-triggering T^0^ threshold, but in both cases below the T1 threshold. In this scenario, since the T1 threshold has not been crossed, the neuronal integrated stress response has not yet been elicited, and the AβPP-independent C99 production pathway remains inoperative. Above (Section 46) and elsewhere [9,10] it has been established that with the influx of iAβ supported solely by the AβPP proteolytic pathway, the transient activation of BACE1 and/or BACE2 would apparently be highly effective in substantially depleting AβPP-derived iAβ. The de novo accumulation of iAβ would commence from a low baseline and proceed at the pre-treatment rate. If the treatment is implemented in mid-life of an individual, accumulating AβPP-derived iAβ would reach neither the T^0^ nor T1 thresholds within the remaining portion of the lifetime. Therefore, if the treatment were carried out prior to the T^0^ crossing (in this scenario the extent of the T^0^ threshold is smaller than that of the T1), it would prevent both conventional AD and AACD (which commences upon the T^0^ crossing). On the other hand, if the treatment were implemented at the levels of AβPP-derived iAβ above the T^0^ but below the T1 thresholds, it would cure AACD and prevent for life the occurrence of conventional AD and recurrence of AACD.

**Figure 40:**
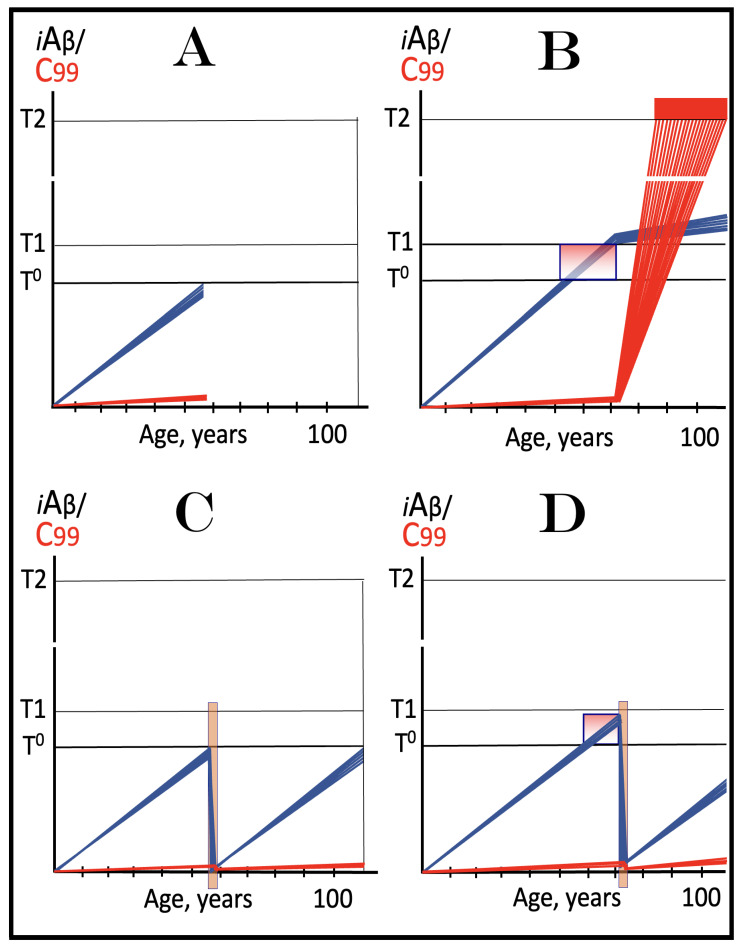
**BACE1 and/or BACE2 activators in the prevention of conventional AD and in the prevention and treatment of AACD: the ACH2.0, Version Three perspective**. *Blue lines*: Intraneuronal Aβ, iAβ. *Red lines*: The C99 fragment of AβPP. ***T*^0^**: Threshold of cellular concentration of AβPP-derived iAβ triggering the neuronal damage, which manifests as AACD. ***T1***: Threshold of cellular concentration of iAβ triggering the activation of PKR and/or HRI kinases, phosphorylation of eIF2α, the elicitation of the neuronal ISR, the initiation of the AβPP-independent C99 production pathway, and the commencement of AD. ***T2***: Cellular concentration of iAβ triggering neuronal death via apoptosis or necroptosis. *Red box*: Apoptotic zone, defined as a range of cellular concentrations of iAβ within which cells neuronal cells committed apoptosis or necroptosis or are dead. *Pink box*: The range of cellular concentrations of AβPP-derived iAβ concentrations between the T^0^ and the highest extent reached by AβPP-derived iAβ or the T1 threshold that support the progression of AACD. The condition commences with the crossing of the T^0^ threshold and morphs into AD with the T1 crossing and elicitation of the ISR; it can occur only if the extent of the T^0^ threshold is smaller than that of the T1. *Orange box*: Duration of the treatment with the BACE1 and/or BACE2-activating drug. *Panel* (**A**): The initial state of the levels of AβPP-derived iAβ and C99 in the neurons of an individual. At the time of the drug’s administration these levels are below the T^0^ threshold. *Panel* (**B**): The evolution of the initial state in the untreated individual. AβPP-derived iAβ continues to accumulate. When it crosses the T^0^ threshold, AACD commences and morphs into AD upon the T1 crossing. At this point PKR and/or HRI kinases are activated, eIF2α is phosphorylated at its Ser51 residue, and the neuronal integrated stress response is elicited. Under the ISR conditions the AβPP proteolytic pathway is suppressed and the rate of accumulation of AβPP-derived iAβ is substantially reduced. Concurrently, the neuronal ISR enables the operation of the AβPP-independent C99 generation pathway. C99 produced independently of AβPP rapidly accumulates and drives the progression of AD pathology. When the T2 threshold is crossed in a sufficient fraction of the neurons, the disease reaches its end stage. *Panel* (**C**): The evolution of the initial state following the transient administration of the BACE1 and/or BACE2-activating drug. The treatment substantially depletes AβPP-derived iAβ and its accumulation resumes from low baseline and proceeds at the pre-treatment rate. Neither the T^0^ or T1 thresholds are reached nor AACD or conventional AD occurs within the remaining lifetime of the treated individual. *Panel* (**D**): The outcome of the transient treatment with the BACE1 and/or BACE2 activating drug administered to the AACD patient. At the time of the implementation of the treatment AβPP-derived iAβ has already crossed the T^0^ threshold and triggered the commencement of AACD. The transient administration of BACE1 and/or BACE2 activators depletes AβPP-derived iAβ well below the T^0^ threshold. When the T1 is reverse-crossed by AβPP-derived iAβ, AACD is cured. The de novo accumulation of AβPP-derived iAβ commences from a low baseline. It reaches neither T^0^ nor T1 thresholds within the remaining lifetime of the treated individual. Thus, a single transient iAβ depletion treatment via the activation of BACE1 and/or BACE2 prevents both the occurrence of conventional AD and recurrence of AACD.

The above considerations are illustrated in Figure 40. Panel A of Figure 40 depicts the initial state of the levels of AβPP-derived iAβ in the neurons of an individual. At the time of the administration of the transient iAβ depletion treatment they are below the T^0^ threshold. Panel B of Figure 40 shows the evolution of the initial state in the untreated individual. AβPP-derived iAβ continues to accumulate. When it crosses the T^0^ threshold, AACD commences and progresses. When AβPP-derived iAβ crosses the T1 threshold, PKR and/or HRI kinases are activated, eIF2α is phosphorylated at its Ser51 residue, and the neuronal integrated stress response is elicited. Under the ISR conditions the AβPP proteolytic pathway is suppressed and the rate of accumulation of AβPP-derived iAβ is substantially reduced. Concurrently, the neuronal ISR enables the production of components essential for the operation of the AβPP-independent C99 generation pathway. As a result, the pathway is activated, and C99 produced independently of AβPP rapidly accumulates and drives the progression of AD pathology. When the T2 threshold is crossed in a sufficient fraction of the neurons, the disease reaches its end stage.

Panel C of Figure 40 illustrates the evolution of the initial state following the transient iAβ depletion treatment via the activation of BACE1 and/or BACE2. The treatment substantially depletes AβPP-derived iAβ and its accumulation resumes from low baseline and proceeds at the pre-treatment rate. Neither the T^0^ or T1 thresholds are reached, nor do AACD or conventional AD occur within the remaining lifetime of the treated individual. Panel D of Figure 40 presents the outcome of the transient treatment with the BACE1 and/or BACE2 activating drug administered to the AACD patient. At the time of the implementation of the treatment AβPP-derived iAβ has already crossed the T^0^ threshold and triggered the commencement of AACD. The transient treatment with activators of BACE1 and/or BACE2 depletes AβPP-derived iAβ well below the T^0^ threshold. When this threshold is reverse-crossed by AβPP-derived iAβ, AACD is cured. The de novo accumulation of AβPP-derived iAβ commences from a low baseline. It reaches neither T^0^ nor T1 thresholds within the remaining lifetime of the treated individual. Thus, a single transient iAβ depletion treatment via the activation of BACE1 and/or BACE2 prevents both the occurrence of conventional AD and recurrence of AACD. It should be emphasized that treatment with directly acting iAβ-specific degradation agents such as appropriately designed proteolysis-targeting chimeras (PROTACs) and molecular-glue degraders (MGDs) would also achieve the outcomes described in the present section above.

## 75. BACE1 and/or BACE2 Activators Can Be Effective in the Treatment of Conventional AD Only if Administered Concurrently with ISR Inhibitors; A Single Transient Composite Therapy Could Prevent for Life the Recurrence of the Disease: The ACH2.0 Version Three Perspective

In the preceding two versions of the ACH2.0, the purpose of the activation of BACE1 and/or BACE2 as a therapeutic strategy in the treatment of conventional AD and the prevention and treatment of unconventional AD was to deplete iAβ (produced overwhelmingly in the AβPP-independent manner). The rationale for such a strategy is simple. By depleting iAβ we remove the driver of AD pathology (a presumption of versions One and Two of the ACH2.0). By depleting iAβ we presumably (in the first two versions of the ACH2.0) also remove the propagator of the neuronal integrated stress response and the sustainer of the activity of the AβPP-independent C99 (and iAβ in versions One and Two) production pathway. Without its driver, the progression of AD pathology ceases, and it takes time (in case of conventional AD, potentially in excess of the remaining lifetime) for iAβ to accumulate to the neuronal ISR-eliciting concentrations.

Whereas the efficiencies of intra-iAβ cleavages by activated BACE1 and, especially, BACE2 are, apparently, sufficient to effectively deplete iAβ produced solely by the AβPP proteolysis, they cannot match the influx of C99 (and iAβ in versions One and Two) generated independently of AβPP. Therefore, to enable the depletion of iAβ (rather than only a reduction in the rate of its accumulation), its production in the AβPP-independent pathway must be stopped for the duration of the administration of BACE1 and/or BACE2 activators. In the composite strategy described above for versions One and Two of the ACH2.0 this is achieved by the concurrent administration of ISR inhibitors. The suppression of the neuronal ISR deprives the AβPP-independent C99 production pathway of its essential components and terminates its operation. With the influx of iAβ produced independently of AβPP abrogated, activated BACE1 and BACE2 can effectively deplete total iAβ.

In Version Three of the ACH2.0 the driver of AD pathology is the C99 fragment generated independently of AβPP; it assumes the role of iAβ produced in the AβPP-independent pathway formulated in the preceding versions of the ACH2.0 in both driving the disease and propagating the neuronal integrated stress response and thus sustaining the activity of the AβPP-independent C99 generation pathway (thus rendering it self-sustainable). Therefore, to attain the objectives of the therapeutic strategy described above, C99 has to be depleted. This is in addition to the depletion of the accumulated AβPP-derived iAβ, so that its de novo accumulation would commence from a low baseline. Fortunately, the degradation and depletion of C99 can be accomplished in precisely the same way as those of iAβ, namely via the activation of intra-iAβ cleaving capabilities of BACE1 and/or BACE2. Indeed, C99 contains the Aβ segment as its integral part. The toxicity of C99 apparently emanates from that of its Aβ segment. Thus, when the Aβ segment is cleaved off by gamma-secretase, the remaining AICD loses the toxicity of C99. Moreover, when only sixteen N-terminal residues are removed from C99, the remaining C83 fragment is rendered non-toxic. It can be firmly anticipated, therefore, that intra-iAβ cleavages by activated BACE1and/or BACE2 would degrade C99 and detoxify its resulting fragments. However, there are two problems. One, the production of BACE enzymes has been suppressed under the neuronal ISR conditions. Another, just as with iAβ in the preceding versions of the ACH2.0, in its version Three the influx of C99 generated independently of AβPP is so great that to deplete it via its degradation by activated BACE1 and/or BACE2, its production in the AβPP-independent pathway must be stopped. Both problems are solved by the transient inhibition of the neuronal ISR concurrently with the activation of BACE1 and/or BACE2.

It should be emphasized that there is a principal distinction of the purpose of the inclusion of ISR inhibitors in the composite C99/iAβ depletion therapy in Version Three of the ACH2.0 versus two preceding ACH2.0 versions. In the preceding versions of the ACH2.0 the sole purpose of ISR inhibitors was to disable the AβPP-independent C99 production pathway and thus to abrogate the influx of iAβ generated independently of AβPP. This purpose remains in effect in Version Three as well: ISR inhibitors would stop the influx of C99 generated independently of AβPP. But in this version of the ACH2.0 there is a second, no less, and arguably more important purpose: to enable the production of BACE1 and BACE2. Indeed, in the ACH2.0, Version Three the neuronal ISR state suppresses all components of the AβPP proteolytic pathway, including the production of BACE enzymes. The reversal of the neuronal ISR state not only disables the AβPP-independent C99 production pathway but also ensures the availability of BACE enzymes. Since, for the reasons described above (Section 43), systemic ISR inhibitors can be administered only transiently, so BACE1 and BACE2 activators can (because BACE enzymes are both unavailable and would be inefficient under the neuronal ISR conditions). The transient composite therapy, nevertheless, would be sufficient to prevent the recurrence of conventional AD for the remaining lifetime of the treated patient.

Thus, the transient composite BACE1 and or BACE2 activation administered concurrently with the ISR inhibition is fully valid as a therapeutic strategy in ACH2.0, Version Three. It would result in the depletion of not only C99, but also of the accumulated AβPP-derived iAβ. Following the completion of the treatment of the AD patient, the accumulation of AβPP-derived iAβ would resume from a low baseline at the pre-T1 crossing rate. It would not reach the T1 threshold, and conventional AD would not recur for the duration of the treatment.

**Figure 41:**
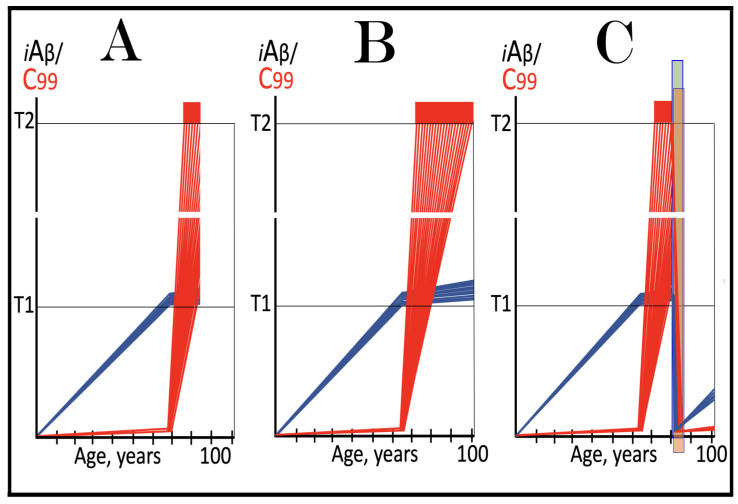
**A single transient composite ISR inhibition/BACE activation therapy could prevent for life the recurrence of conventional AD: the ACH2.0 Version Three perspective**. *Blue lines*: Intraneuronal Aβ, iAβ. *Red lines*: The C99 fragment of AβPP. ***T1***: Threshold of cellular concentration of iAβ triggering the activation of PKR and/or HRI kinases, phosphorylation of eIF2α, the elicitation of the neuronal ISR, the initiation of the AβPP-independent C99 production pathway, and the commencement of AD; its extent is the only variable in the present figure. ***T2***: Cellular concentration of iAβ triggering neuronal death via apoptosis or necroptosis. *Red box*: Apoptotic zone, defined as a range of cellular concentrations of iAβ within which cells neuronal cells committed apoptosis or necroptosis or are dead. *Green box*: Duration of the treatment with the ISR-inhibiting drug. *Orange box*: Duration of the treatment with the BACE1 and/or BACE2-activating drug. *Panel* (**A**): The initial state of the levels of AβPP-derived iAβ and C99, produced mainly in the AβPP-independent pathway, at the time of the administration of the composite BACE activation/ISR inhibition therapy. AβPP-derived iAβ has crossed the T1 threshold in all affected neurons. PKR and/or HRI have been activated, eIF2α phosphorylated at its Ser51 residue, and the neuronal ISR elicited. As a result, the AβPP proteolytic pathway has been suppressed and the rate of accumulation of AβPP-derived iAβ substantially reduced. Concurrently, the production of C99 in the AβPP-independent pathway has been activated, and AD commenced. It rapidly accumulated, crossed the T2 threshold in a fraction of the neurons, and AD symptoms manifested. *Panel* (**B**): The evolution of the initial state in the untreated patient. The accumulation of C99 generated independently of AβPP continues, more neurons cross the T2 threshold, and the disease enters its end stage. *Panel* (**C**): The evolution of the initial state following the composite BACE activation/ISR inhibition therapy. The reversal of the neuronal ISR disables the AβPP-independent C99 generation pathway and ceases the influx of its C99 product. It also restores the production of BACE enzymes and ensures their availability. Activated BACE1 and/or BACE2 efficiently and substantially deplete both C99 and iAβ. When drugs are withdrawn, the progression of AD is arrested; the accumulation of AβPP-derived iAβ resumes from low baseline and proceeds at a slow pre-T1 crossing rate. It would not reach the T1 threshold and conventional AD would not recur in the treated individual. Thus, a single composite BACE activation/ISR inhibition treatment can prevent for life the recurrence of conventional AD.

The considerations described above are illustrated in Figure 41. Panel A of Figure 41 depicts the initial state of the levels of AβPP-derived iAβ and C99, produced mainly in the AβPP-independent pathway, at the time of the administration of the composite BACE activation/ISR inhibition therapy. In this panel, AβPP-derived iAβ, accumulating via the cellular uptake of secreted extracellular Aβ and the retention of a fraction of Aβ resulting from gamma-cleavages on the intraneuronal membranes, has crossed the T1 threshold in all affected neurons. PKR and/or HRI have been activated, eIF2α phosphorylated at its Ser51 residue, and the neuronal ISR elicited. As a result, the AβPP proteolytic pathway has been suppressed and the rate of accumulation of AβPP-derived iAβ substantially reduced. Concurrently, the AβPP-independent C99 generation pathway has been activated, and AD commenced. C99 produced independently of AβPP has rapidly accumulated, crossed the T2 threshold in a fraction of the neurons, and AD symptoms manifested. Panel B of Figure 41 shows the evolution of the initial state in the untreated patient. The accumulation of C99 generated independently of AβPP continues unimpeded, more neurons cross the T2 threshold, and the disease enters its end stage. Panel C of Figure 41 presents the evolution of the initial state following the composite BACE activation/ISR inhibition therapy. The reversal of the neuronal ISR disables the AβPP-independent C99 generation pathway and ceases the influx of C99 produced independently of AβPP. It also restores the production of BACE enzymes and ensures their availability. With AβPP-independent C99 production pathway inoperative, activated BACE1 and/or BACE2 efficiently and substantially deplete both C99 and iAβ. When drugs are withdrawn, the progression of AD is arrested; the accumulation of AβPP-derived iAβ resumes from low baseline and proceeds at a slow pre-T1 crossing rate. It would not reach the T1 threshold and conventional AD would not recur in the treated individual. Thus, a single composite BACE activation/ISR inhibition treatment can prevent for life the recurrence of conventional AD. It should be emphasized that in this strategy BACE1 and/or BACE2-activating drugs can be replaced, with the same effect, with directly acting iAβ-specific degradation agents such as appropriately designed proteolysis-targeting chimeras (PROTACs) and molecular-glue degraders (MGDs).

## 76. Transient Composite BACE Activation/ISR Inhibition Therapy in the Prevention and Treatment of Unconventional AD: The ACH2.0 Version Three Perspective

The present section considers the potential outcomes of the transient composite BACE activation/ISR inhibition therapy in the prevention and treatment of unconventional AD. In this form of the disease AβPP-derived iAβ has little, if any, relevance. The disease is triggered by the neuronal integrated stress response elicited unconventionally, i.e., by a stressor distinct from AβPP-derived iAβ, when the levels of the latter are below the T1 threshold. The neuronal ISR activates, in turn, the AβPP-independent C99 generation pathway, and C99 produced independently of AβPP rapidly accumulates. If the duration of the occurrence of unconventional stressors and, consequently, of the ISR state is sufficient, C99 levels would cross the T1 threshold, the AβPP-independent C99 generation pathway would become self-sustainable, and the disease would commence. In the present and the following sections, we consider the scenarios where once the unconventional stressor capable of eliciting the neuronal integrated stress response appeared, it persists for the remaining lifetime.

In unconventional AD, as in its conventional counterpart, the objective of the therapeutic strategy is to substantially deplete the driver of AD pathology, in this case C99 generated independently of AβPP. Inhibiting the neuronal ISR and, concurrently activating BACE1 and/or BACE2 can accomplish this. The inhibition of the ISR enables the production of BACE enzymes. It also disables the AβPP-independent C99 generation pathway, stops the influx of C99 produced independently of AβPP, and empowers the efficient depletion of C99. BACE activators can be used only in the absence of the ISR state. Therefore, since ISR inhibitors can be employed only transiently, BACE activators would be effective for no longer duration. It is expected that at the completion of the transient composite BACE activation/ISR inhibition treatment C99 would be substantially depleted. However, unlike in conventional AD, in the unconventional form of the disease the depletion of C99 does not result in the cessation of the activity of the AβPP-independent C99 generation pathway. This is because, as soon as drugs are withdrawn, the neuronal ISR would be unconventionally re-elicited (due to the persistent presence of unconventional stressors), and AβPP-independent C99 production pathway unconventionally activated. The accumulation of C99 produced independently of AβPP would resume from a low baseline but as soon as it crosses the T1 threshold, AD would commence (or re-commence). The delay in the occurrence of the disease (in preventive cases) or in its recurrence (in the treatment scenario) would be relatively short, but still probably measured in years.

**Figure 42:**
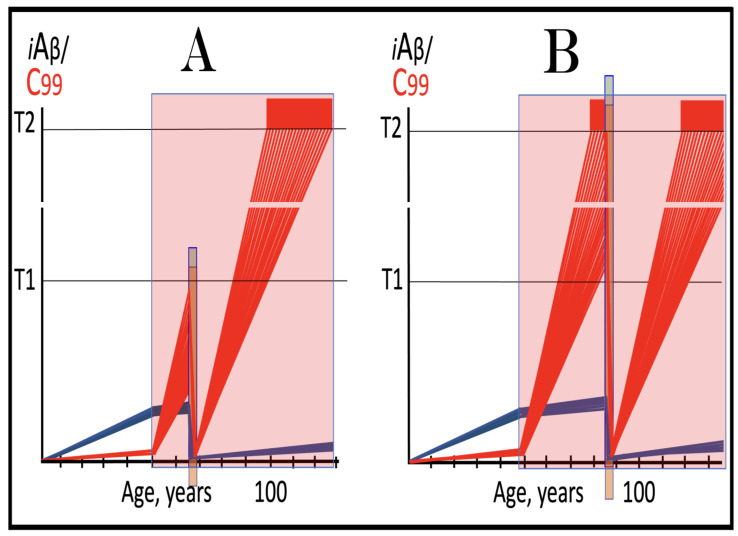
**Transient composite ISR inhibition/BACE activation therapy in the prevention and treatment of unconventional AD: The ACH2.0 Version Three perspective**. *Blue lines*: Intraneuronal Aβ, iAβ. *Red lines*: The C99 fragment of AβPP. ***T1***: Threshold of cellular concentration of iAβ triggering the activation of PKR and/or HRI kinases, phosphorylation of eIF2α, the elicitation of the neuronal ISR, the initiation of the AβPP-independent C99 production pathway, and the commencement of AD; its extent is the only variable in the present figure. ***T2***: Cellular concentration of iAβ triggering neuronal death via apoptosis or necroptosis. *Red box*: Apoptotic zone, defined as a range of cellular concentrations of iAβ within which cells neuronal cells committed apoptosis or necroptosis or are dead. *Green box*: Duration of the treatment with the ISR-inhibiting drug. *Orange box*: Duration of the treatment with the BACE1 and/or BACE2-activating drug. *Pink boxes*: The duration of the presence of the unconventional stressor capable of activating one or more of eIF2α kinases and triggering the elicitation of the neuronal ISR. *Panel* (**A**): The anticipated outcome of the transient composite BACE activation/ISR inhibition therapy in the prevention of unconventional AD. At the time of the administration of the therapy the neuronal ISR has been unconventionally elicited at the levels of AβPP-derived iAβ below the T1 thresholds. Consequently, the AβPP proteolytic pathway and the accumulation of AβPP-derived iAβ have been suppressed, and the AβPP-independent C99 generation pathway unconventionally activated. C99 produced independently of AβPP has rapidly accumulated but is still below the T1 threshold; the disease does not commence. The ISR inhibition enables the production of BACE enzymes, disables the production of C99 in the AβPP-independent C99 pathway, and stops its influx. In this setting, activated BACE1 and/or BACE2 efficiently deplete C99 and iAβ. When drugs are withdrawn, the neuronal ISR is unconventionally re-elicited, and the AβPP-independent C99 generation pathway unconventionally reactivated. C99 produced independently of AβPP rapidly accumulates. When it crosses the T1 threshold the disease commences. *Panel* (**B**): Anticipated effects of the transient composite BACE activation/ISR inhibition therapy in the treatment of unconventional AD. At the time of the treatment the neuronal ISR has been unconventionally elicited at levels of AβPP-derived iAβ below the T1 threshold. The AβPP proteolytic pathway has been suppressed and the AβPP-independent C99 production pathway unconventionally activated. C99 generated independently of AβPP has rapidly accumulated and crossed the T1 threshold. The AβPP-independent C99 production pathway was rendered self-sustainable, and AD commenced. C99 has continued to accumulate, crossed the T2 threshold in a fraction of the neurons, and AD symptoms manifested. The composite treatment resulted in a substantial depletion of C99 and iAβ. However, upon the withdrawal of the drugs, the neuronal ISR is unconventionally re-elicited, the AβPP-independent C99 production pathway unconventionally reactivated; C99 rapidly accumulates, re-crosses the T1 threshold, and the disease recurs.

The above considerations are illustrated in Figure 42. Panel A of Figure 42 depicts the anticipated outcome of the implementation of the transient composite BACE activation/ISR inhibition therapy in the prevention of unconventional AD. In this panel, at the time of the administration of the therapy the neuronal integrated stress response has been unconventionally elicited at the levels of AβPP-derived iAβ below the T1 thresholds. Consequently, the AβPP proteolytic pathway has been suppressed and the AβPP-independent C99 generation pathway unconventionally activated. C99 produced independently of AβPP has rapidly accumulated but at the commencement of the composite therapy is still below the T1 threshold; the disease, therefore, has not yet commenced. The ISR inhibition enables the production of BACE enzymes, disables the AβPP-independent C99 generation pathway, and stops the influx of C99 produced independently of AβPP. In this setting, activated BACE1 and/or BACE2 efficiently deplete C99. When drugs are withdrawn, the neuronal ISR is unconventionally re-elicited, and the AβPP-independent C99 generation pathway unconventionally reactivated. C99 produced independently of AβPP rapidly accumulates. When it crosses the T1 threshold the disease commences and progresses.

Panel B of Figure 42 shows anticipated effects of the concurrent administration of BACE activators and ISR inhibitors in the treatment of unconventional AD. In this panel, the neuronal ISR has been unconventionally elicited at levels of AβPP-derived iAβ below the T1 threshold. The AβPP proteolytic pathway has been suppressed and the AβPP-independent C99 production pathway unconventionally activated. C99 generated independently of AβPP has rapidly accumulated and crossed the T1 threshold. The AβPP-independent C99 production pathway was rendered self-sustainable, and AD commenced. C99 has continued to accumulate, crossed the T2 threshold in a fraction of the neurons, and AD symptoms manifested. The transient concurrent administration of ISR inhibitors and BACE activators led to substantial depletion of C99 in exactly the same way as described in the preceding paragraph. However, upon the withdrawal of the drugs, the neuronal ISR is unconventionally re-elicited, the AβPP-independent C99 production pathway unconventionally reactivated; C99 rapidly accumulates, re-crosses the T1 threshold, and the disease recurs. Thus, in both scenarios, preventive and curative, the transient administration of the composite BACE activation/ISR inhibition therapy is anticipated to provide a temporary reprieve from either the occurrence or recurrence of unconventional AD; the duration of such a reprieve is probably measured in years.

If, in the above-described strategy, BACE-activating drugs were substituted by directly acting iAβ-specific degradation agents such as appropriately designed proteolysis-targeting chimeras (PROTACs) and molecular-glue degraders (MGDs), similar outcomes would be attained. Importantly, utilization of directly acting iAβ-specific degradation agents instead of BACE activators in the above strategy provides an additional option of a long-term administration because, unlike BACE enzymes, once introduced, their cellular presence is not affected by the ISR state; the anticipated outcomes of this approach are analogous to those discussed (for BACE activators) in Section 61 and shown in Figure 31.

## 77. Recurrent Transient Composite BACE1 and/or BACE2 Activation/ISR Inhibition Therapy in the Prevention and Treatment of Unconventional AD: The ACH2.0 Version Three Perspective

As discussed in the preceding section, the activated BACE-mediated degradation of C99/iAβ is feasible only in the presence of ISR inhibitors, and since the latter can be used only transiently, so the former can. However, the transient concurrent administration of BACE activators and ISR inhibitors provides only a temporary reprieve, both preventive and curative: following the treatment, either the occurrence or the recurrence of unconventional AD is only delayed. The benefit, therefore, is transient, lasting, apparently, a few years. On the other hand, this limitation, both in the prevention and treatment of unconventional AD can be circumvented. Indeed, the duration of benefits conferred by both preventive and curative applications of the transient composite BACE activation/ISR inhibition therapy could be multiplied by repeating the treatment at strategically timed intervals. Conceptually, the treatments could continue recurrently for the remaining lifetime, ensuring that unconventional AD neither occurs in the preventively treated individual nor recurs in the treated patient.

**Figure 43:**
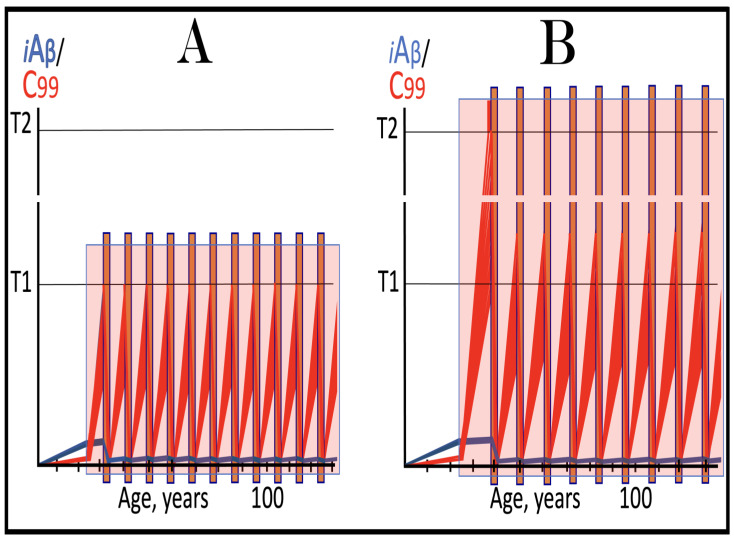
**Recurrent transient composite BACE1 and/or BACE2 activation/ISR inhibition therapy in the prevention and treatment of unconventional AD: The ACH2.0 Version Three perspective**. *Blue lines*: Intraneuronal Aβ, iAβ. *Red lines*: The C99 fragment of AβPP. ***T1***: Threshold of cellular concentration of iAβ triggering the activation of PKR and/or HRI kinases, phosphorylation of eIF2α, the elicitation of the neuronal ISR, the initiation of the AβPP-independent C99 production pathway, and the commencement of AD; its extent is the only variable in the present figure. ***T2***: Cellular concentration of iAβ triggering neuronal death via apoptosis or necroptosis. *Red box*: Apoptotic zone, defined as a range of cellular concentrations of iAβ within which cells neuronal cells committed apoptosis or necroptosis or are dead. *Pink boxes*: The duration of the presence of the unconventional stressor capable of activating one or more of eIF2α kinases and triggering the elicitation of the neuronal ISR. *Brown boxes*: The duration of the composite treatment with concurrently administered ISR inhibitors and BACE activators. *Panel* (**A**): Effects of the recurrent transient composite BACE activation/ISR inhibition therapy in the prevention of unconventional AD. At the time of the initial treatment the neuronal ISR has been unconventionally elicited. Consequently, the AβPP proteolytic pathway has been suppressed and the rate of accumulation of AβPP-derived iAβ substantially reduced. Concurrently, the production of C99 in the AβPP-independent pathway has been activated. It has rapidly accumulated but has not yet reached the T1 threshold. The inhibition of the neuronal ISR enables the production of BACE enzymes, disables the AβPP-independent C99 generation pathway, stops the influx of its C99 product, and empowers the efficient depletion of the latter by activated BACE1 and/or BACE2. When drugs are withdrawn, the neuronal ISR is unconventionally re-elicited, the AβPP-independent C99 generation pathway unconventionally reactivated, and C99 resumes its accumulation from a low baseline. Before it reaches the T1 threshold, at the time points determined by appropriate biomarkers, the treatment is repeated recurrently for the remaining portion of the lifetime; neither the T1 threshold would be crossed, nor AD would occur in the treated individual. *Panel* (**B**): Effects of the recurrent transient composite BACE activation/ISR inhibition therapy in the treatment of unconventional AD. At the time of the initial treatment, the neuronal ISR has been unconventionally elicited, the AβPP proteolytic pathway suppressed, AβPP-independent C99 generation pathway unconventionally activated, the C99 product of the latter has crossed the T1 threshold, the pathway was rendered self-sustainable, and the disease commenced. In a fraction of the neurons C99 has crossed the T2 threshold, and AD symptoms have manifested. The concurrent administration of ISR-inhibiting and BACE-activating drugs enable the production of BACE enzymes, cease the operation of the AβPP-independent C99 generation pathway, and block the influx of its C99 product. In this setting, activated BACE1 and/or BACE2 efficiently deplete C99 and iAβ. Upon the completion of the treatment, the neuronal ISR is unconventionally re-elicited, AβPP-independent C99 production pathway unconventionally reactivated, and the accumulation of its C99 product resumes from a low baseline. It crosses the T1 threshold, but before it reaches AD-pathology-causing levels, at time points defined by suitable biomarkers, the transient composite therapy is re-implemented recurrently for the remaining lifetime. The AD-pathology-causing range of C99 concentrations would not be reached, and the disease would not recur in the treated patient.

The above strategy is illustrated schematically In Figure 43. Panel A of Figure 43 depicts the effects of the recurrent transient composite BACE activation/ISR inhibition therapy in the prevention of unconventional AD. In this panel, the neuronal integrated stress response has been unconventionally elicited at the levels of AβPP-derived iAβ below the T1 threshold. Consequently, the AβPP proteolytic pathway has been suppressed and the rate of accumulation of AβPP-derived iAβ substantially reduced. Concurrently, the neuronal ISR has activated the AβPP-independent C99 generation pathway. C99 produced independently of AβPP has rapidly accumulated but at the time of the initial administration of the transient composite BACE activation/ISR inhibition therapy has not yet reached the T1 threshold. The inhibition of the neuronal ISR enables the production of BACE enzymes, disables the AβPP-independent C99 generation pathway, stops the influx of its C99 product, and empowers the efficient depletion of the latter by activated BACE1 and/or BACE2. When drugs are withdrawn, the neuronal ISR is unconventionally re-elicited, the AβPP-independent C99 generation pathway unconventionally reactivated, and C99 produced independently of AβPP resumes its accumulation from a low baseline. Before it reaches the T1 threshold, at the time points determined by appropriate biomarkers, the treatment is repeated recurrently for the remaining portion of the lifetime; neither the T1 threshold would be crossed, nor AD would occur in the treated individual.

Panel B of Figure 43 shows the effects of the recurrent transient composite BACE activation/ISR inhibition therapy in the treatment of unconventional AD. In this panel, at the time of the initial administration of the transient composite therapy, the neuronal ISR has been unconventionally elicited, the AβPP proteolytic pathway suppressed, AβPP-independent C99 generation pathway unconventionally activated, the C99 product of the latter has crossed the T1 threshold, the pathway was rendered self-sustainable, and the disease commenced. In a fraction of the neurons C99 has crossed the T2 threshold, and AD symptoms have manifested. The concurrent administration of ISR-inhibiting and BACE-activating drugs enables the production of BACE enzymes, ceases the operation of the AβPP-independent C99 generation pathway, and blocks the influx of its C99 product. In this setting, activated BACE1 and/or BACE2 efficiently deplete C99 (and iAβ). Upon the completion of the treatment, the neuronal ISR is unconventionally re-elicited, AβPP-independent C99 production pathway unconventionally reactivated, and the accumulation of its C99 product resumes from a low baseline. It crosses the T1 threshold but before it reaches AD-pathology-causing levels, at time points defined by suitable biomarkers, the transient composite therapy is re-implemented recurrently for the remaining lifetime. The AD-pathology-causing range of C99 concentrations would not be reached, and the disease would not recur in the treated patient.

## 78. Sustained Reduction/Removal of Unconventional Stressors Would Convert Unconventional AD into Conventional AD, Preventable and Treatable by a Single Transient Administration of the Composite BACE Activation/ISR Inhibition Therapy

The preceding sections demonstrate the feasibility of preventing and treating unconventional AD by recurrent applications of transient composite BACE activation/ISR inhibition therapy administered every few years for the remaining lifetime. There is, however, a better way to do it. Indeed, it is of the utmost importance to emphasize that the best way to approach the prevention and treatment of unconventional AD is to perform the transient composite BACE activation/ISR inhibition therapy (or utilize directly acting iAβ degradation agents instead of BACE activators in this approach) in conjunction with the reduction or removal of unconventional stressors capable of eliciting the neuronal integrated stress response via the elimination of their source. The unconventional stressors form the foundation of unconventional AD. By eliciting the neuronal integrated stress response, they enable the operation of the AβPP-independent C99 generation pathway and sustain it until C99 produced independently of AβPP crosses the T1 threshold and the pathway is rendered self-sustainable. If the unconventional stressors were removed (or their levels reduced below those required for the elicitation of the neuronal ISR), the outcomes of the transient composite BACE activation/ISR inhibition therapy would be as follows. The transient composite BACE activation/ISR inhibition treatment would substantially deplete C99 and AβPP-derived iAβ. When drugs are withdrawn both C99 and AβPP-derived iAβ are well below the neuronal ISR-eliciting T1 threshold. Unconventional stressors are either sustainably removed or their levels are below those needed for the elicitation of the neuronal ISR. Therefore, the neuronal ISR is not re-elicited, and the AβPP-independent C99 generation pathway remains inoperative. Under these conditions the de novo accumulation of AβPP-derived iAβ would commence from a low threshold and proceed at a slow rate. It would not cross the T1 threshold, and no AD would occur or recur within the remaining lifetime of the treated individual. The removal of unconventional neuronal ISR-eliciting stressor effectively converts unconventional AD (or perspective unconventional AD) into conventional one. One-time-only C99/iAβ depletion treatment, therefore, would be sufficient to either prevent the disease or preclude its recurrence for life.

**Figure 44:**
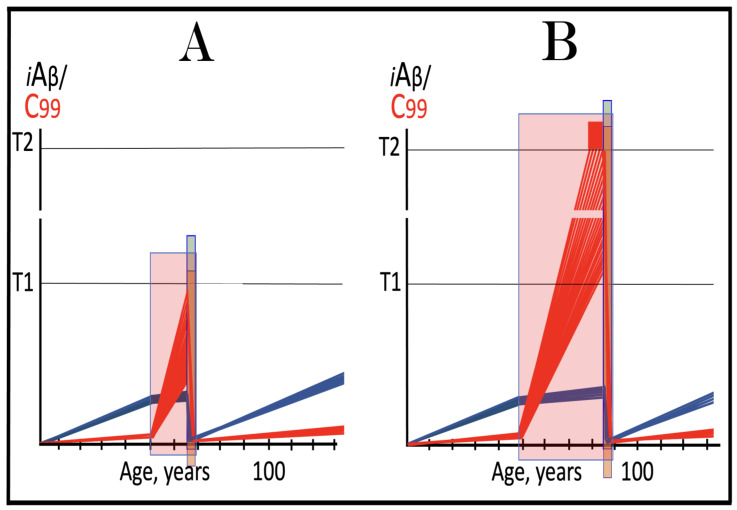
**Sustained removal of unconventional stressors converts unconventional AD into conventional one, preventable and treatable by a single transient administration of the composite BACE activation/ISR inhibition therapy**. *Blue lines*: Intraneuronal Aβ, iAβ. *Red lines*: The C99 fragment of AβPP. ***T1***: Threshold of cellular concentration of iAβ triggering the activation of PKR and/or HRI kinases, phosphorylation of eIF2α, the elicitation of the neuronal ISR, the initiation of the AβPP-independent C99 production pathway, and the commencement of AD; its extent is the only variable in the present figure. ***T2***: Cellular concentration of iAβ triggering neuronal death via apoptosis or necroptosis. *Red box*: Apoptotic zone, defined as a range of cellular concentrations of iAβ within which cells neuronal cells committed apoptosis or necroptosis or are dead. *Green box*: Duration of the treatment with the ISR-inhibiting drug. *Orange box*: Duration of the treatment with the BACE1 and/or BACE2-activating drug. *Pink boxes*: The duration of the presence of the unconventional stressor capable of activating one or more of eIF2α kinases and triggering the elicitation of the neuronal ISR. *Panel* (**A**): The anticipated outcome of the transient composite BACE activation/ISR inhibition therapy administered in concert with the sustained removal of unconventional stressors in the prevention of unconventional AD. At the time of the treatment the neuronal ISR has been unconventionally elicited, the AβPP proteolytic pathway has been suppressed and the rate of accumulation of AβPP-derived iAβ reduced. Concurrently, the AβPP-independent C99 production pathway has been unconventionally activated; its C99 product has accumulated but is still below the T1 threshold. The transient administration of the composite BACE activation/ISR inhibition therapy is carried out in concert with the sustained removal of unconventional stressors (or their depletion below levels below the neuronal ISR-eliciting levels). When the transient BACE activation/ISR inhibition treatment is completed, both C99 and AβPP-derived iAβ are substantially depleted and so are unconventional stressors. The neuronal ISR is not re-elicited, and the AβPP-independent C99 production pathway remains inoperative. The accumulation of AβPP-derived iAβ resumes from a low baseline and proceed at a slow rate. It does not reach the T1 threshold and AD does not occur within the lifetime of the treated individual. *Panel* (**B**): The anticipated outcome of the transient composite BACE activation/ISR inhibition therapy administered in concert with the sustained removal of unconventional stressors in the treatment of unconventional AD. At the time of the treatment C99 produced in the unconventionally activated AβPP-independent pathway has crossed the T1 threshold, the pathway became self-sustainable, and AD commenced. C99 has continued to accumulate, crossed the T2 threshold in a fraction of the neurons, and AD symptoms manifested. The transient administration of the composite BACE activation/ISR inhibition therapy is carried out in concert with the sustained removal of unconventional stressors. When BACE-activating and ISR-inhibiting drugs are withdrawn, C99 and AβPP-derived iAβ are substantially depleted. The progression of the disease ceases, and the AβPP-independent C99 generation pathway remains inoperative. The de novo accumulation of AβPP-derived iAβ commences from low baseline and proceeds at a slow rate. It would not reach the T1 threshold, and the disease would not recur within the lifetime of the treated patient. Thus, the sustained removal/depletion of unconventional stressors capable of eliciting the neuronal ISR effectively converts unconventional AD into conventional one, preventable and treatable by a single transient composite ISR inhibition/BACE activation treatment.

The above considerations are illustrated in Figure 44. In Panel A of Figure 44 the neuronal ISR has been unconventionally elicited at levels of AβPP-derived iAβ below the T1 threshold. The AβPP proteolytic pathway has been suppressed and the rate of accumulation of AβPP-derived iAβ reduced. Concurrently, the AβPP-independent C99 production pathway has been unconventionally activated; its C99 product has accumulated but is still below the T1 threshold. The transient application of the composite BACE activation/ISR inhibition therapy is carried out in conjunction with the sustained removal of unconventional stressors or the reduction in their levels below those required for the elicitation of the neuronal ISR. As detailed above, when the transient BACE activation/ISR inhibition treatment is completed, both C99 and AβPP-derived iAβ are substantially depleted. Importantly, there are no (or there are insufficient) unconventional stressors, and upon the withdrawal of BACE activators and ISR inhibitors the neuronal ISR is not re-elicited, and the AβPP-independent C99 production pathway remains inoperative. In such a setting, the accumulation of AβPP-derived iAβ would resume from a low baseline and proceed at a slow rate. It would not reach the T1 threshold and AD would not occur within the lifetime of the treated individual.

In Panel B of Figure 44, C99 produced in the unconventionally activated AβPP-independent pathway has crossed the T1 threshold, the pathway became self-sustainable, and AD commenced. C99 has continued to accumulate, crossed the T2 threshold in a fraction of the neurons, and AD symptoms manifested. At this point the transient composite treatment with activators of BACE1 and/or BACE2 and inhibitors of the ISR is performed in concert with the sustained removal of unconventional stressor or their sustained depletion below the neuronal ISR-eliciting levels. When BACE-activating and ISR-inhibiting drugs are withdrawn, C99 and AβPP-derived iAβ are substantially depleted. The progression of the disease ceases. With unconventional stressors sustainably removed and C99 and AβPP-derived iAβ well below the T1 threshold, the AβPP-independent C99 generation pathway remains inoperative. The de novo accumulation of AβPP-derived iAβ commences from low baseline and proceeds at a slow rate. It would not reach the T1 threshold, and the disease would not recur within the lifetime of the treated patient.

## 79. Phase Two of the RNA-Dependent Mammalian mRNA Amplification: AβPP Is Potentially Produced in AD-Affected Neurons Unconventionally in an ISR-Driven and ISR-Compatible Process

Section 10, Section 11, Section 12 and Section 13 above discussed the “chimeric” phase of RNA-dependent mammalian mRNA amplification in general and that of human AβPP mRNA in particular. It is a powerful process akin to massive gene amplification. Indeed, every molecule of the amplification-eligible mRNA species potentially serves repeatedly as a template (dubbed “progenitor mRNA”) for the production of additional mRNA molecules. The first stage in the chimeric phase of mRNA amplification is synthesis of the antisense RNA strand initiating at the 3′poly(A) segment of the mRNA molecule. The presence of the poly(A) segment is, apparently, the only eligibility requirement for serving as a template for antisense RNA. The specificity of the amplification process is introduced at the following stage, which mandates the occurrence of two complementary segments within the antisense strand (the 3′ Terminal Complementary Element, TCE, and the Internal Complementary Element, ICE) that are mutually accessible in the folded configuration of the antisense RNA strand. Consequently, the antisense strand folds into a self-priming structure and is extended into a sense-orientation RNA. This is followed by separation of strands and cleavage of the chimeric RNA intermediate. One of the end products of the chimeric phase of mRNA amplification is a chimeric mRNA molecule consisting of 5′-truncated mRNA containing an antisense segment at its 5′ terminus. It encodes either the original polypeptide or, as in the case of human AβPP mRNA amplification, only a C-terminal fragment, CTF, of the original polypeptide. Another end product of the chimeric pathway is the 3′-truncated antisense RNA molecule. Initially, it was assumed to be a non-functional by-product. However, subsequent observations by Kapranov and coworkers of a class of modified mRNA molecules truncated at the 5′ end and containing 5′-terminal poly(U) segment [289] indicated that 3′-truncated antisense RNA end product of the chimeric phase of mRNA amplification can be utilized as a progenitor in the second, PCR-like phase of the mRNA amplification process.

**Figure 45:**
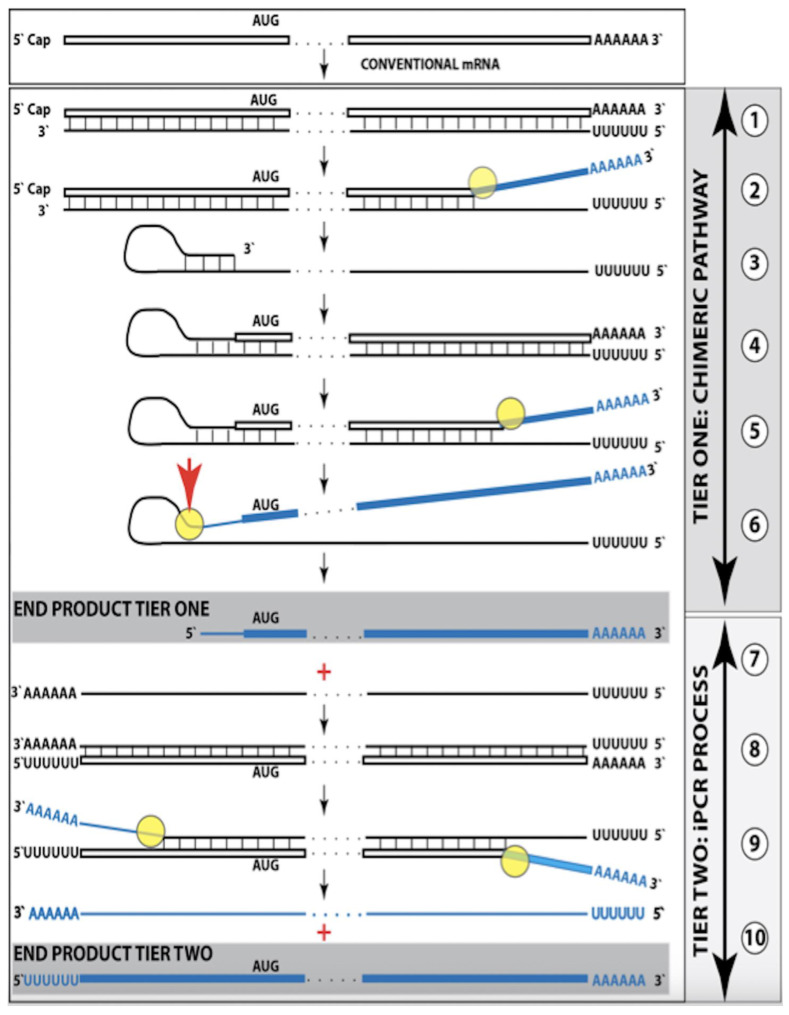
**Mammalian RNA-dependent mRNA amplification occurs in two phases: Principal stages**. *Single lines*: Antisense RNA. *Boxed lines*: Sense RNA. *Blue boxed lines*: Single-stranded RNA separated from its complementary RNA strand by helicase. *Yellow circles*: Helicase complex containing helicase strand-separating activity, nucleotide modifying activity and RNA cleaving activity. *Red arrows*: positions of the cleavage of the intermediate generating RNA end products of the amplification process. *RdRp*: RNA-dependent RNA polymerase. *AUG*: Translation initiation codon. *TCE*: 3′ Terminal Complementary Element of the antisense RNA strands. *ICE*: Internal Complementary Element of the antisense RNA strands. ***Top panel***: Conventionally transcribed mRNA molecule referred to as progenitor mRNA. ***Bottom panel***: Stages of the chimeric pathway of the RNA-dependent mRNA amplification; the ICE is located within a portion of antisense RNA corresponding to the 5′ UTR of the progenitor mRNA molecule. (**1**): The progenitor mRNA is transcribed by RdRp into antisense RNA. (**2**): Complementary RNA strands are separated by the helicase activity. Helicase complex mounts the 3′ poly(A) segment and moves along the sense RNA strand modifying, on average, every fifth nucleotide. (**3**): Folding of the antisense RNA into self-priming configuration is guided by the interaction of its TCE and ICE elements. (**4**): RdRp extends the 3′ terminus of self-primed antisense RNA. This produces a hairpin-like molecule containing both sense and antisense RNA components, referred to as the chimeric RNA intermediate. (**5**): Complementary strands of the chimeric RNA intermediate are separated by the helicase activity. Nucleotide modifications introduced during the separation prevent the re-annealing of the sense and antisense RNA strands. (**6**): When the helicase reaches the single-stranded portion of the chimeric intermediate (either the 5′ end of the TCE or a TCE/ICE mismatch) it cleaves the RNA molecule; this cleavage is coupled with polyadenylation of the 3′ terminus of the truncated antisense RNA molecule. (**7**): End products of RNA-dependent mRNA amplification. Antisense RNA is truncated and polyadenylated at its 3′ end and sense portion of the chimeric RNA is truncated at the 5′ end and acquires the cleaved-off antisense RNA fragment. The polyadenylation of the antisense RNA strand denotes the commencement of the second phase of mRNA amplification. (**8**): 3′poly(A)-containing antisense RNA is the initial template (progenitor) in the intracellular PCR (iPCR) amplification process; it is transcribed into the sense RNA strand, initiating at the 3′poly (A), by RdRp. (**9**): The helicase complex separates RNA strands. (**10**): End products of the first iPCR cycle. Each contains 3′poly (A) and 5′poly(U) and each can serve as a PCR template in the RdRp-driven process.

Both phases of mRNA amplification are illustrated schematically in Figure 45. Stages 1 through 6 (designated “Tier One” in Figure 45) are identical to those discussed in Section 10 and shown in Figure 1. Briefly, the antisense RNA is transcribed from the mRNA progenitor by RNA-dependent RNA polymerase, RdRp. After strands are separated by a helicase complex, antisense RNA, guided by the interaction of its TCE and ICE elements, folds into a self-priming configuration, and its 3′ terminus (TCE) is extended into a sense-orientation molecule. This results in a hairpin-like structure (designated “chimeric RNA intermediate”). Complementary regions of the hairpin structure are then separated by the helicase complex, which mounts the 3′ poly(A) segment and proceeds in the 5′ direction. When it reaches the single-stranded portion of the hairpin structure, it cleaves it. The end product of this phase of mRNA amplification (designated “End Product Tier One” in Figure 45) is the chimeric mRNA described above. In the human AβPP mRNA amplification process it encodes the C100 fragment of AβPP (eventually processed into C99, see Section 23 above).

The cleavage of the chimeric RNA intermediate denotes the end of the first phase and the beginning of the second phase of the mRNA amplification process (designated “Tier Two” in Figure 45) [58,65,66]. In conjunction with the cleavage, the helicase complex or its associated activity polyadenylates the newly generated 3′ terminus of the antisense RNA strand. The resulting antisense RNA molecule contains, therefore, the 3′-terminal poly(A) segment and the 5′-terminal poly(U) segment (transcribed from the 3′ poly(A) of the mRNA progenitor molecule); it constitutes the progenitor molecule in the second phase of mRNA amplification. The presence of the 3′-terminal poly(A) segment makes the antisense RNA molecule an eligible RdRp template. The product of its transcription is the sense-orientation mRNA containing both 3′-terminal polyA) and 5′-terminal poly(U); it is, upon separation of strands by the helicase complex, also an eligible RdRp template. Since both strands are eligible RdRp templates, the entire process constitutes a physiologically occurring intracellular polymerase chain reaction, iPCR (for intracellular PCR).

The sense-orientation RNA product of the iPCR phase of mRNA amplification is always the mRNA molecule with the intact protein coding capacity of the progenitor mRNA, even if the chimeric phase of amplification produces mRMA encoding only a CTF of the original polypeptide. This may seem paradoxical but the explanation is simple. Indeed, the protein coding capacity of the chimeric mRNA end product of the first phase of amplification depends on the position of the ICE. If it were located in the region of antisense RNA corresponding to the 5′UTR of the mRNA molecule, the chimeric mRNA end product would contain the entire coding region of the mRNA progenitor. If, however, the ICE is positioned within the antisense RNA segment corresponding to the coding region of the progenitor mRNA molecule, the chimeric RNA end product would be 5′-truncated within the coding region of mRNA progenitor, as occurs during the amplification of human AβPP mRNA (see Section 11, Section 12 and Section 13 above). In both cases, however, the mRNA end product of the iPCR phase of amplification would contain the entire coding region of the mRNA progenitor. This is because the 5′ truncation of the chimeric mRNA end product is defined by the position of the ICE, which can be anywhere. In contrast, the 5′ truncation of the mRNA produced in the iPCR phase of amplification is no longer than the length of the TCE (because the cleavage of the chimeric RNA intermediate occurs either within the TCE or at its 5′ end), which is much shorter than an average 5′UTR. In human AβPP mRNA 5′UTR is 149 nucleotides long whereas the TCE of its antisense strand is only 33 nucleotides long. Thus, the mRNA end product of the iPCR phase of human AβPP mRNA amplification can be truncated by no more than 33 nucleotides (in contrast, the chimeric mRNA end product of the first phase of human AβPP mRNA amplification is truncated by nearly two thousand nucleotides). Therefore, its translation would result in the entire AβPP.

Both phases of the amplification of human AβPP mRNA can occur only in AD-affected neurons under the integrated stress response conditions, which provides components essential for the operation of RNA-dependent mRNA amplification machinery. Hence, in another apparent paradox, the neuronal ISR suppresses the AβPP proteolytic pathway, including the conventional production of AβPP, but enables the production of the latter in an ISR-driven and ISR-compatible process. Thus, the product of the first, chimeric, phase of human AβPP mRNA amplification, namely C99, apparently drives AD pathology, whereas the product of the second, iPCR, phase of AβPP mRNA amplification, namely AβPP (which cannot be processed proteolytically under the neuronal ISR conditions), potentially facilitates the viability and functionality of the disease-affected neurons.

## 80. Validation (13): Detection of Predictably 5′-Truncated, 5′ Poly(U)-Containing AβPP mRNA in Human Neuronal Cell-Based AD Models

The notion of the second phase of RNA-dependent mammalian mRNA amplification, as outlined in the preceding section, plausibly explains the origin of a class of human mRNA species that are truncated (in comparison with the original transcription products of their corresponding genes) within their 5′UTRs and possess poly(U) segments appended to their 5′ termini [289]. In this class of mRNAs, all truncations cluster around 8-17 nucleotides, conceivably a typical size of the TCE element (and/or a reflection of the position of a mismatch within the TCE/ICE complex) and are, thus, consistent with the proposed explanation. Moreover, the occurrence of the second phase of the RNA-dependent mRNA amplification was validated in the case of the amplification of alpha-globin and beta-globin mRNA [66]. The occurrence of the first, chimeric, phase of amplification for these mRNA species was proven by the detection of nucleotide sequences containing their “chimeric junctions”, i.e., regions containing the antisense RNA segment extended into sense-orientation RNA. The reason that the detection of the chimeric RNA intermediate rather than of chimeric RNA end product (which is much more abundant) was used for validation is that the latter is undetectable by hybridization and, therefore, by cDNA-based sequencing techniques. This is because when the helicase complex moves along the 3′-terminal poly(A)-containing RNA strand, it modifies on average every fifth nucleotide (apparently to preclude the re-annealing of the strands and potentially to enable translation of amplified mRNA under the ISR conditions). The completed chimeric RNA end product is modified throughout. This is not the case for the chimeric intermediate. The chimeric junction region is generated (by extension of the TCE) unmodified and remains such until the extension is completed, and the helicase complex traverses the distance between the junction and the 3′-terminal poly(A). Thus, the pool of chimeric RNA intermediate with yet unmodified chimeric junction region should occur while the amplification process is operational and would provide the detection opportunities.

Such chimeric junction regions were indeed detected for both alpha-globin and beta-globin mRNAs [65,66]. This indicated the positions of anticipated cleavage sites of chimeric RNA intermediates, which, in turn, provided opportunities to validate the occurrence of the second phase of the amplification process. Indeed, the occurrence of mRNA 5′-truncated at the predicted position and containing 5′-terminal poly(U) segments would validate the proposed mechanism. Such a validation was indeed obtained for both alpha and beta-globin mRNAs [65,66]. The same type of validation for the occurrence of the second phase (iPCR) of AβPP mRNA amplification can be obtained in human neuronal cell-based AD models. It would be much easier to detect the predictably 5′-truncated, 5′-terminal poly(U)-containing mRNA segment then 3′-truncated, 3′-terminal poly(A)-containing segment of the antisense RNA. This is because, as shown in Figure 45, the latter would not stay unmodified for long since this is where the strands separation (and modification of the poly(A)-containing strand) begins, whereas the former would remain unmodified for much greater duration of the traverse of the helicase complex along the entire length of the mRNA molecule. Therefore, with the RNA-dependent human AβPP mRNA amplification process operational, the pool of not yet modified 5′ polyuridylated sense RNA molecules (or their 5′ portions) would be much greater than that of 3′ polyadenylated antisense RNA molecules. In Section 15, we identified potential cleavage sites of the chimeric RNA intermediate of the human AβPP mRNA amplification process. This enables us to predict the following validation targets for the occurrence of the second phase of the human AβPP mRNA amplification process in human neuronal cell-based AD models (based on the anticipated cleavage sites of chimeric RNA intermediates shown in Section 15, Figure 3 above):

(1) …UUUUUUUUUUUUUUUUUUUUUUUUUUUUUUCCUCGGCAGCGGUAGGCGAGAGCACGCGGAGGAGCGUGCGCGGGGGCCCCGGGAGACGGCGGCGGUGGCGGCGCGG…
(2) …UUUUUUUUUUUUUUUUUUUUUUUUUUUUUGUAGGCGAGAGCACGCGGAGGAGCGUGCGCGGGGGCCCCGGGAGACGGCGGCGGUGGCGGCGCGGGCAGAGCAAGGA…
(3) …UUUUUUUUUUUUUUUUUUUUUUUUUUUUUAGGCGAGAGCACGCGGAGGAGCGUGCGCGGGGGCCCCGGGAGACGGCGGCGGUGGCGGCGCGGGCAGAGCAAGGACG…

## 81. Alternative RNA-Based Therapeutic Strategies for AD Targeting AβPP Antisense RNA and 5′ Terminus of AβPP mRNA via: (1) TSS Shift, (2) AβPP Gene Editing, and (3) Anti-Antisense AβPP Oligonucleotides

In terms of the ACH2.0 theory of AD, the C99 fragment of AβPP generated independently of AβPP drives the Alzheimer’s disease. Therefore, all therapeutic strategies for AD considered in the present *Perspective* can be distilled to one common point: the prevention or cessation of the activity of the AβPP-independent C99 production pathway. This is, for example, exactly what the reversal of the neuronal integrated stress response (either by direct inhibition or via the removal of stressors capable of its elicitation) does. We do not know yet how to intervene with the AβPP-independent C99 production pathway directly, but long-term systemic interference with this pathway could be no less deleterious than that with integrated stress response. This is definitely the case if this pathway is, in fact, RNA-dependent amplification of mRNA, a scenario we consider overwhelmingly plausible. However, whereas we cannot interfere with the enzymatic machinery of this pathway in general, due to its physiological significance, we certainly can interfere with the amplification of human AβPP mRNA in particular. More specifically, undermining the eligibility of AβPP mRNA to serve as the amplification template is, apparently, a valid therapeutic strategy for AD (in its both, conventional and unconventional forms; this strategy, however, would not impact AACD). This can be accomplished in three ways.

### 81.1. Shift of Transcription Start Sites, TSSs, of Human AβPP mRNA

**Figure 46:**
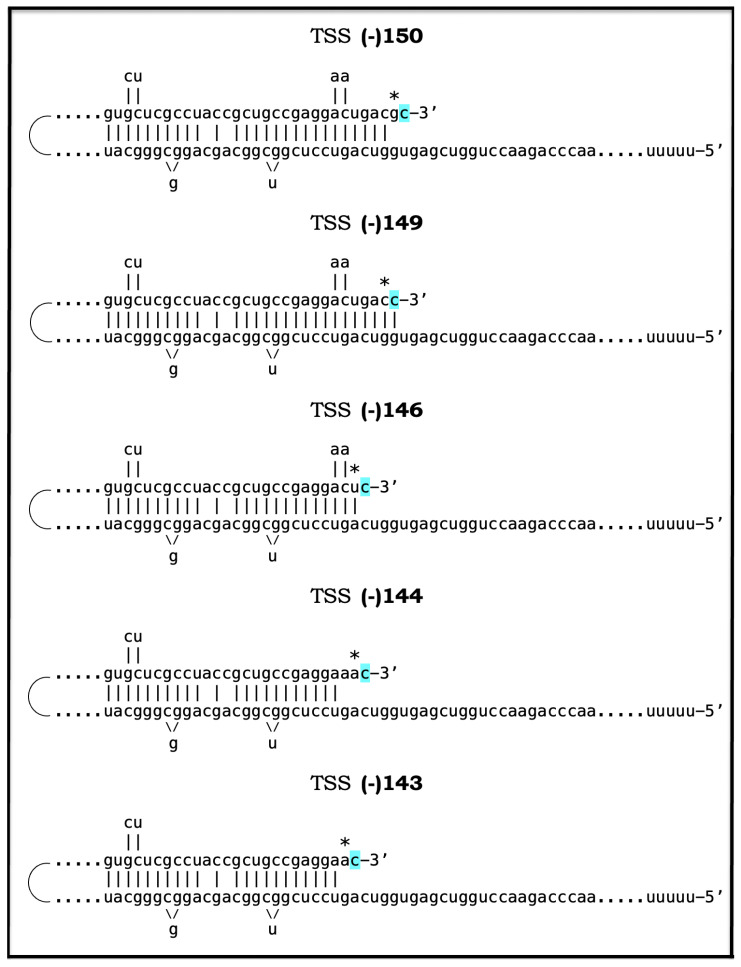
**Folding of the antisense RNA molecules corresponding to human AβPP mRNAs initiated at the known transcription start sites**. *TSS*: Transcription start site. *(-)150*, *(-)149*, *(-)146*, *(-)144*, *(-)143*: Known transcription start sites of human AβPP mRNA. Distances are given in numbers of nucleotides upstream from the translation-initiating AUG codon. *Asterisk*: The nucleotide of the antisense RNA molecule corresponding to the transcription-initiating nucleotide of human AβPP mRNA. Highlighted *in blue*: “C”, which is transcribed from the 5′-terminal cap “G” of AβPP mRNA. Only the antisense RNA strand originating from human AβPP mRNA transcribed from the TSS (-)149 is capable of forming self-primed structure without the 3′-overhang.

The AβPP gene belongs to the category of housekeeping genes that lack the promoter component known as TATA box. The TATA box is typically situated 30 nucleotides upstream from the TSS and defines its position. In genes lacking the TATA box transcription can and usually does initiate at multiple sites, i.e., these genes possess multiple TSSs. This is definitely the case with the human AβPP gene, which has five demonstrated transcription start sites [63]. They are situated at the following positions (in number of nucleotides upstream from the “A” of the AUG translation initiation codon): (-)150, (-)149, (-)146, (-)144, (-)143. Since nucleotides at these sites are in penultimate 5′-terminal positions within AβPP mRNA (the ultimate 5′-terminal position is occupied by the capG), they are of special importance for the structure of the TCE element of the antisense strand and its ability to form a stable complex with the ICE, without any 3′ overhangs. Of the five TSSs listed above only one, namely (-)149, yields AβPP antisense RNA that satisfies these requirements. The situation is exacerbated by the observation that capG of mRNA is transcribed into 3′-terminal “C” of the antisense RNA molecule and has to be accommodated into the TCE/ICE structure. The TCE/ICE complexes formed by antisense complements of human AβPP mRNA molecules initiated from different TSSs are depicted in Figure 46 (asterisks denote nucleotides corresponding to TSS of mRNA). As stated above, the only AβPP antisense RNA capable of forming stable, 3′ overhang-free TCE/ICE complex is that corresponding to AβPP mRNA initiated from the TSS (-)149. It follows that the frequency of the usage of the TSS (-)149 potentially determines the rate of accumulation of C99 produced independently of AβPP and thus defines the rate of the progression of AD. It also follows that the shift of the TSSs of AβPP mRNA could be a powerful therapeutic tool. Indeed, the modulation of the usage of the known TSSs away from the TSS (-)149 would be beneficial: it would lower the rate of the production of C99 independently of AβPP and reduce the rate of the progression of the disease. Shifting TSSs of human AβPP mRNA more extensively could be far more beneficial. Indeed, shifting it upstream from “regular” TSSs would introduce a substantial 3′ overhang into the TCE/ICE complex; it would, in fact, render the 3′TCE non-terminal and disable its utility in the extension of the 3′ terminus of the antisense RNA molecule. If the TSS is shifted substantially downstream from regularly used sites (but still within the 5′UTR of AβPP mRNA) so that the segment corresponding to the TCE of the antisense molecule is either significantly shortened or eliminated altogether, the TCE/ICE complex would be either destabilized or eliminated. AβPP mRNA amplification would be disabled, and the progression of AD stopped. Importantly, with all manipulations described above, AβPP mRNA would retain its protein coding capacity.

### 81.2. Limited AβPP Gene Editing as Therapeutic Strategy for AD

The objective of destabilizing the TCE/ICE complex formed by the antisense AβPP RNA molecule or eliminating it altogether can be accomplished also by a limited editing of the AβPP gene in human neurons. Indeed, the removal of a sufficiently large portion of the gene’s segment corresponding to the TCE element of AβPP antisense RNA would destabilize the TCE/ICE complex or even prevent its formation. Alternatively, a portion, if not the entire segment of the AβPP gene corresponding to the TCE element of AβPP antisense RNA can be replaced by a “neutral” nucleotide sequence, i.e., by a sequence, which gives rise to a segment of AβPP antisense RNA (replacing its TCE) that is not complementary to its ICE element. This also would prevent the formation of the TCE/ICE complex. In addition, potentially, changes in the segment of the AβPP gene corresponding to the 5′UTR of AβPP mRNA could be sufficient to alter the folding of AβPP antisense RNA in such a way as to render the TCE and ICE elements mutually inaccessible and thus prevent the formation of the TCE/ICE complex. Such modifications would preclude the RNA-dependent amplification of human AβPP mRNA and, consequently, prevent AD in it both conventional and unconventional forms. Importantly, the proposed alterations would not interfere with the protein coding potential of AβPP mRNA. It should be emphasized that the feasibility of these two strategies (TSS shift and 5′ gene editing) has been validated by the observations that human AβPP mRNA expressed from 5′-modified transgenes in mouse models cannot be amplified (discussed in Section 18).

### 81.3. Therapeutic Utilization of Anti-Antisense Oligonucleotides Targeting AβPP Antisense RNA in General and Its TCE and ICE Elements in Particular

The two therapeutic strategies discussed above, namely the shift of TSSs of human AβPP mRNA and the alteration of a segment of the AβPP gene encoding 5′UTR of AβPP mRNA, impact AβPP antisense RNA and the interaction of its TCE and ICE elements indirectly, by affecting the structure of either the AβPP gene or AβPP mRNA. This mandates certain restrictions. For example, any alterations must be limited to the 5′UTR of AβPP mRNA (or corresponding portion of the gene) in order not to interfere with the protein coding potential of AβPP mRNA. Even among those, some alterations could interfere significantly with the efficiency of transcription and/or translation of AβPP and thus would be unusable. These limitations are immaterial in the therapeutic strategy utilizing anti-antisense oligonucleotides. In this strategy only AβPP antisense RNA is targeted and affected, and, therefore, there are no restrictions in the choice of particular targets within the RNA molecule. The primary targets are, of course, the TCE and ICE elements of AβPP antisense RNA. The interference with either would affect, possibly prevent, the formation of the TCE/ICE complex. Moreover, potentially, interference with any portion of AβPP antisense RNA may affect the mutual accessibility of the TCE and ICE elements within folded AβPP antisense RNA. Such interference would potentially disable the AβPP mRNA amplification process, and either prevent AD or arrest its progression. Since neither the AβPP gene nor AβPP mRNA are impacted in this approach, translation of the latter would not be hindered in any way. This approach is feasible in light of the recent development of nucleic acid-carrying lipid vehicles capable of crossing the blood–brain barrier [290,291,292,293].

## 82. Validation (14): Prevention of Cellular AD Pathology in Human Neuronal Cell-Based AD Models by Limited Editing of the 5′ Terminus of AβPP mRNA or via the Employment of Anti-Antisense Oligonucleotides

One of the therapeutic approaches described in the preceding section, namely the shift of TSSs of human AβPP mRNA, cannot be currently tested experimentally because techniques to execute such a shift have yet to be developed. In contrast, two other RNA-based therapeutic strategies discussed above can be readily validated in human neuronal cell-based AD models. In these models, cellular AD pathology, including the formation of neurofibrillary tangles, can be induced either conventionally, via the exogenous production of iAβ (which elicits the neuronal integrated stress response when accumulated over the T1 threshold), or unconventionally, through the sustained elicitation of the neuronal integrated stress response by stressors other than iAβ. In both cases, endogenous self-sustainable AβPP-independent production of C99, presumably via RNA-dependent amplification of AβPP mRNA, is activated, and AD pathology is driven by C99 generated independently of AβPP. Such a model, therefore, presents two measurable variables: (1) the extent of AD pathology in general and the formation of NFTs in particular, and (2) the occurrence of C100, i.e., N-terminal Met-C99 (see Section 23 above).

The validation of the therapeutic strategies discussed above can be carried out exactly as proposed in the preceding section. In case of the AβPP gene editing, a segment of the gene corresponding to the TCE element of AβPP antisense RNA can be either removed or replaced by a neutral nucleotide sequence in such a way that in the antisense RNA molecule the TCE is substituted by a nucleotide sequence, which is not complementary to the ICE element. In case of anti-antisense oligonucleotides, they should target either the TCE or the ICE elements of the antisense AβPP RNA. In both cases, gene editing and anti-antisense oligonucleotides, the interference, if successful, would prevent the formation of the TCE/ICE complex. Consequently, the amplification of AβPP mRNA would be precluded. Neither would NFTs be formed nor C100 generated in human neuronal cells following the sustained elicitation of the neuronal ISR.

## 83. Conclusions: Hope Is in the Air

### 83.1. The Initial Version of the ACH2.0: Principal Tenets

The present *Perspective* analyses the evolution of the Amyloid Cascade Hypothesis 2.0 (ACH2.0) theory of Alzheimer’s disease. The central thread of this evolution is the diminishing role of Aβ in AD. The initial version of the theory marginalized the role of extracellular Aβ, previously believed to be both the causative agent and the driver of the disease and posited that the disease is triggered by intraneuronal Aβ (iAβ) derived from AβPP by the proteolysis of the latter and accumulated over the critical threshold, and is driven by iAβ produced by gamma-secretase cleavage of the C99 fragment of AβPP generated independently from AβPP. In this paradigm, the bridge between differentially produced iAβ is the neuronal integrated stress response. Evidently, AβPP-derived iAβ acts as a neuronal ISR-eliciting stressor. Upon reaching certain cellular concentrations, it activates PKR and/or HRI, both eIF2α kinases. Consequently, eIF2α is phosphorylated at its Ser51 residue, and the elicitation of the neuronal ISR ensues. A pivotal role of the neuronal ISR in AD is enabling the production of components essential for the operation of the AβPP-independent C99 generation pathway. Apparently, iAβ produced by the AβPP proteolysis alone cannot reach the AD pathology-causing range of cellular concentrations. On the other hand, the entire iAβ output of the AβPP-independent C99 production pathway is retained within the neurons, rapidly accumulates, and drives AD pathology. It also propagates the neuronal ISR state, making it independent from the influx of AβPP-derived iAβ, and thus perpetuates the activity of the AβPP-independent C99 generation pathway.

### 83.2. ACH2.0, Version Two: Conventional and Unconventional AD

A major step in the evolution of the ACH2.0 was the realization that the AβPP-derived iAβ-triggered form of AD is only a special, albeit prevalent, category of the disease, and that Alzheimer’s is not a disease of AβPP-derived iAβ but rather a disease of the neuronal integrated stress response. In this paradigm, the sustained elicitation of the neuronal ISR by ANY stressor results in AD. To distinguish between the two categories of AD and their respective triggers, the disease initiated by AβPP-derived iAβ has been referred to as “conventional AD” and its trigger, AβPP-derived iAβ, as the “conventional stressor”, whereas the disease triggered by stressors (any stressors) distinct from AβPP-derived iAβ was designated as “unconventional AD” and stressors triggering it have been referred to as “unconventional stressors”. Sources of unconventional neuronal ISR-eliciting and, consequently, unconventional AD-triggering stressors are numerous. They can be of mechanical nature, such as traumatic brain injury or chronic traumatic encephalopathy, or can take the form of viral and bacterial infections as well as of various types of inflammation. The common features of all such sources include a decreased cerebral blood flow and compromised blood–brain barrier. The latter may allow unconventional stressors to penetrate the brain and, subsequently, the neurons whereas the former was shown to trigger mitochondrial dysfunction and, consequently, activate the ISR-eliciting HRI kinase in neuronal cells. Multiple forms of dementia classified until now as AD-like dementia (ADLD) and AD-related dementia (ADRD) all, apparently, belong to the unconventional AD category. In unconventional AD the role of Aβ is significantly diminished. AβPP-derived iAβ has little, if any, relevance to the disease but AD pathology is still driven (in the second variant of the ACH2.0) by iAβ produced by gamma-secretase cleavages of C99 generated independently of AβPP.

### 83.3. ACH2.0, Version Three: AβPP Proteolytic Pathway Is Suppressed in AD-Affected Neurons; The Disease Is Driven by C99 Generated Independently of AβPP

The next milestone in the evolution of the ACH2.0 was the formulation of its Version Three. In this version, the AβPP proteolytic pathway is suppressed in AD-affected neurons following the elicitation of the neuronal ISR. This suppression encompasses the production of AβPP, of BACE enzymes, of components of the gamma-secretase complex, and, consequently, of Aβ and iAβ. Due to the deficiency of gamma-secretase, the processing of C99 generated independently of AβPP into iAβ is also suppressed. Because of the persisting aura of Aβ as the principal player in AD, it took both time and effort to formulate this notion, but this is where the logic and empirical data lead, and it is only prudent for us to follow. This conclusion was derived from a detailed analysis of the current transgenic mouse models of AD. They are rather “attempted models of AD”, because, despite vast exogenous overproduction of human AβPP, none of them develops the full spectrum of AD pathology but exhibit only the effects of the neuronal ISR elicited by exogenous AβPP-derived iAβ. The AβPP-independent C99 generation pathway is inoperative in mouse models. Therefore, the analysis left only one plausible explanation for the lack of the massive neurodegeneration, namely that the influx of AβPP-derived iAβ is discontinued or severely suppressed following the elicitation of the neuronal ISR. In retrospect, this is rather a trivial assumption. The global cellular protein synthesis is severely suppressed by the integrated stress response, this is the epitome of the ISR state, and there is no reason why constituents of the AβPP proteolytic pathway would be exempted.

Thus, constituents of the AβPP proteolytic pathway are not exempted but the C99 fragment is produced in the ISR-compatible process. It is both interesting and fitting that tau, a protein forming the major hallmark of AD, neurofibrillary tangles, is also produced under the neuronal ISR conditions (if it were not, the theory would be inconsistent with the data). But in the tau case, it is exempted from the ISR-exerted suppression of cellular protein synthesis. This is because the human tau mRNA contains an internal ribosomal entry site (IRES) [284]. The IRES is positioned within the 5′UTR of tau mRNA; translation, therefore, proceeds in the ISR-compatible manner and yields the complete tau protein

**Figure 47:**
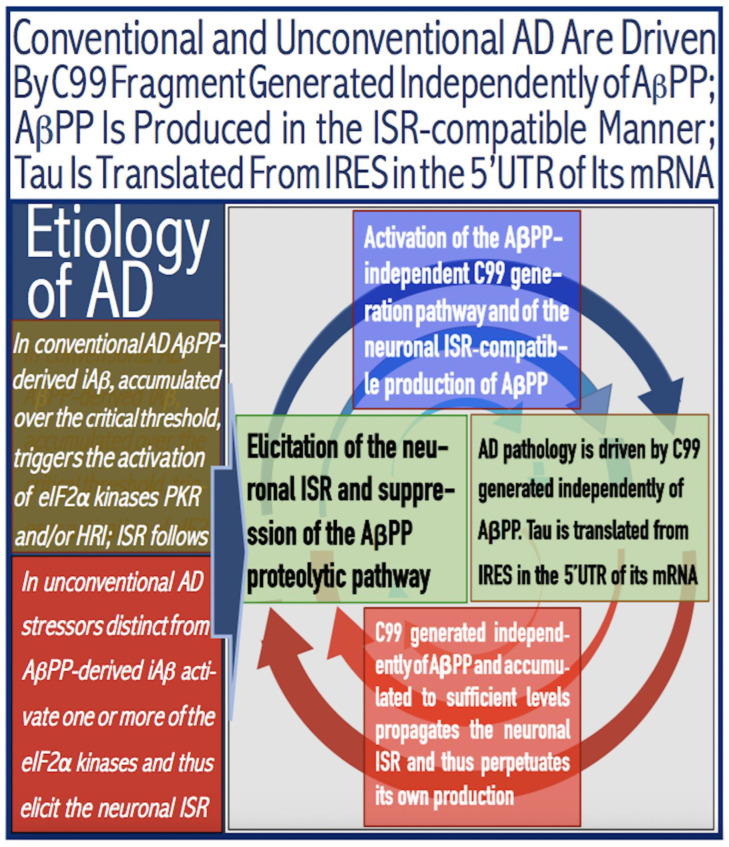
**Current understanding of AD: The neuronal ISR suppresses the AβPP proteolytic pathway but enables the AβPP-independent generation of C99, which drives the disease; AβPP is produced in the ISR-compatible process but not processed; Tau is translated from IRES in the 5′UTR of its mRNA**. *eIF2α*: eukaryotic translation initiation factor 2*α*. *PKR and HRI*: kinases that phosphorylate eIF2α at its Ser51 residue. *PACT*: PKR activator. *TNFα*: tumor necrosis factor *α. OMA1*: mitochondrial protease activated during mitochondrial dysfunction. *DELE1*: substrate of OMA1 protease. Cleavage of DELE1 leads to the activation of HRI. Phosphorylation of eIF2α elicits the integrated stress response, which, in turn, enables the generation of components essential for the activity of the AβPP-independent pathway of C99 and iAβ production. iAβ: intraneuronal Aβ. AβPP-derived iAβ accumulates physiologically via the importation of extracellular Aβ and the retention of iAβ produced by gamma-cleavages on intracellular membranes in an exceedingly slow process. In most individuals it does not reach the PKR- and/or HRI-activating, the neuronal ISR-eliciting threshold within their lifetimes and no conventional AD occurs. When this threshold is crossed, the neuronal ISR is elicited and the disease ensues. In unconventional AD, stressors that are distinct from AβPP-derived iAβ and capable of activating one or more of eIF2α kinases trigger the elicitation of the neuronal ISR. Under the ISR conditions the AβPP proteolytic pathway is suppressed; this happens within the framework of the ISR-mediated inhibition of the global cellular protein synthesis and includes AβPP, BACE enzymes, components of the gamma-secretase complex and, consequently, Aβ and iAβ. Concurrently, the neuronal ISR provides the components essential for, and thus enables, the operation of the AβPP-independent C99 generation pathway, and it is this C99, which drives AD pathology. Simultaneously, in the second phase of the same mechanism that underlies AβPP-independent generation of C99, AβPP is produced in the ISR-compatible process but is not processed further. In general, in the AD-affected neurons, any new protein synthesis has to occur in an ISR-compatible manner. In cases of C99 and AβPP this is achieved, apparently, via the nucleotide modifications of their mRNAs. Translation of tau protein, on the other hand, persists under the ISR conditions because its mRNA contains an internal ribosomal entry site in the 5′UTR.

This alteration of the ACH2.0 deprives Aβ from its formerly major role, namely driving AD pathology, thus leaving it with only one function in the disease: triggering the neuronal ISR in the conventional form of AD. It also establishes C99 generated independently of AβPP as the driver of AD pathology and the sustainer of the activity of the AβPP-independent C99 generation pathway. Due to its demonstrated cellular toxicity and the ability to trigger the elicitation of the neuronal ISR, C99 is fully qualified for such a role, which is consistent with the observation of substantial accumulation of C99 in AD-affected neurons but not in the disease-resistant regions of the human brain. The final (to-date) alteration to the ACH2.0 is a notion that, after all, AβPP is produced (though it cannot be processed proteolytically due to the deficiency of gamma-secretase) in the AD-affected neurons not conventionally, but rather in a neuronal ISR-driven and ISR-compatible process. In fact, it is the second phase of the same pathway, which generates C99 independently of AβPP. Thus, interestingly, the same process (although in two distinct phases) produces both the driver of AD pathology (C99) and the substance (AβPP) that potentially facilitates the viability and functionality of AD-affected neurons. The current understanding of AD, in its both conventional and unconventional forms, in the ACH2.0 perspective is summarized graphically in Figure 47.

### 83.4. ACH-Based AD Drugs in the ACH2.0 Perspective

The current interpretation of the disease in the ACH2.0 perspective allows the evaluation of the potential of currently available as well as future AD drugs. All presently FDA-approved AD drugs belong to the ACH-based category of AD drugs. ACH-based drugs are named so because their design and function have been guided by the ACH. Accordingly, their stated aim is to deplete extracellular Aβ. They achieve this goal by either sequestering their target (e.g., Aβ-specific antibodies) or by reducing its production and thus secretion (e.g., BACE1 inhibitors). Also in this category are gamma-secretase modulators, which shift the production of AβPP-derived Aβ toward shorter, more benign species. ACH-based drugs are legitimate (albeit inefficient) conventional AD-preventive agents in the ACH2.0 perspective. This is because by reducing the pool of extracellular Aβ they also lower the rate of its cellular uptake and, consequently, decrease the rate of iAβ accumulation. In case of BACE inhibitors, the retention of iAβ resulting from gamma-cleavages on intraneuronal membranes is also reduced. The decreased rate of iAβ accumulation translates into delayed or prevented T1 crossing and thus delayed or precluded occurrence of conventional AD. As for gamma-secretase modulators, they elevate the extent of the T1 threshold. As a result, its crossing is delayed or prevented and so is the occurrence of the conventional disease. All these drugs, however, can have no positive effect whatsoever when the self-sustainable AβPP-independent C99 generating pathway is operational. Effects of lecanemab and donanemab in very early AD were marginal because they acted only preventively and only on a marginal neuronal subpopulation that did not yet cross the T1 threshold and where the AβPP-independent C99 generating pathway was not yet activated. It can be fully expected that any ACH-based drug would elicit the same preventive response if administered at similarly early stages of AD. These drugs would have no effect (neither preventive nor curative) of any sort in unconventional AD.

### 83.5. ACH2.0-Based Drugs in the Prevention of Conventional AD and AACD and in the Treatment of AACD: BACE1 and/or BACE2 Activators

In sharp contrast, the ACH2.0-based drugs hold extraordinary promise for the prevention and treatment of both conventional and unconventional forms of AD, as well as for the prevention and treatment of AACD. By definition, the ACH2.0-based drugs are the agents that either deplete iAβ (and thus remove the trigger of conventional AD when applied preventively) and/or C99 (and thus abolish both the driver of the disease and the sustainer of its own AβPP-independent production) via their selective degradation or terminate the activity of the AβPP-independent C99 generation pathway in any other way. As discussed above, in the current version (Version Three) of ACH2.0, iAβ plays a central role only in conventional AD and in aging-associated cognitive decline, AACD. Its accumulation over the T^0^ threshold denotes the commencement of AACD (provided T^0^ < T1). When iAβ crosses the T1 threshold, it triggers the elicitation of the neuronal integrated response, activates the self-sustainable AβPP-independent C99 generation pathway and initiates conventional AD; AACD morphs into AD at this point. No AACD commences until the T^0^ threshold is crossed and no AD occurs until the T1 threshold is reached. This opens up tantalizing preventive opportunities. If iAβ is depleted at mid-life by transient treatment with activators of BACE1 and/or BACE2 (or by any other iAβ–degrading agent), and if the depletion is “deep” enough, de novo accumulating iAβ would not reach the T^0^ and T1 thresholds within the lifetime of the individual and neither AACD nor AD would occur. If the treatment is administered following the T^0^ but prior to the T1 crossing, AACD would be cured, and neither would it recur nor would AD occur within the individual’s lifespan. Thus, a single, one time only transient treatment with BACE1 and/or BACE2 activating drugs can prevent both conventional AD and AACD and cure the latter.

### 83.6. ACH2.0-Based Drugs in the Treatment of Conventional AD: Concurrent BACE1 and/or BACE2 Activation and Neuronal ISR Inhibition

No less spectacular outcomes can be attained in the treatment of conventional AD. In this case, however, the AβPP-independent C99 generation pathway is operational and, due to the high rate of the influx of C99 produced independently of AβPP, activated BACE1 and/or BACE2 can only reduce but not reverse its accumulation and thus can only slow down but not stop the progression of the disease. The solution is the transient composite C99/iAβ depletion therapy. BACE1 and/or BACE2 activators (or any other iAβ–degrading agent) are administered transiently and concurrently with ISR inhibitors. The latter reverse the neuronal ISR state, restore the production of BACE enzymes, disable the AβPP-independent C99 generation pathway and abrogate the influx of C99 whereas the former activate BACE enzymes, which efficiently deplete both C99 and AβPP-derived iAβ. The progression of the disease ceases and the accumulation of iAβ resumes from a low baseline at a slow pre-T1 crossing rate. It does not reach the T1 threshold, and the disease does not recur within the lifetime of the treated patient. Thus, a single, once in a lifetime transient composite therapy with concurrently administered BACE1 and/or BACE 2 activators and ISR inhibitors can stop for life the progression of conventional Alzheimer’s disease and preclude its recurrence.

### 83.7. ACH2.0-Based Drugs in the Prevention and Treatment of Unconventional AD: Recurrent Composite BACE Activation/ISR Inhibition Therapy; Sustained Removal of Unconventional Stressors as the Ultimate Goal

The prevention and treatment of unconventional presents significantly more challenges. This is because the elicitation of the neuronal ISR and subsequent activation of the AβPP-independent C99 generation pathway (and suppression of AβPP proteolytic pathway) are triggered by unconventional stressors occurring at the levels of AβPP-derived iAβ below the T1 threshold and persistently present thereafter. The disease would commence only when C99 crosses the T1 threshold, and the AβPP-independent pathway of its production becomes self-sustainable. To prevent the disease is to prevent the T1 crossing. C99 (and AβPP-derived iAβ) can be depleted by the transient composite treatment with BACE1 and /or BACE2 activators and ISR inhibitors (transient because long-term administration of ISR inhibitors would be deleterious, and BACE occurrence and, consequently, activation is feasible only under ISR inhibition). The problem is that as soon as drugs are withdrawn, the neuronal ISR would be unconventionally re-elicited, AβPP-independent C99 generation pathway reactivated, and its C99 product would rapidly accumulate. This would only delay the occurrence of AD, possibly for a few years.

Conceptually the same process would happen in the treatment of unconventional AD. Following the composite C99 depletion treatment, the progression of the disease would cease but the AβPP-independent pathway of C99 production would be reactivated; it would rapidly accumulate and cross the T1 threshold, and the disease would recur. A solution, in both the prevention and treatment of unconventional AD, is the recurrent application of the transient BACE activation/ISR inhibition treatment, administered at strategically timed intervals under the guidance of the appropriate biomarkers.

On the other hand, a more fundamental solution, in both the prevention and treatment of unconventional AD, is to administer a single transient composite BACE activation/ISR inhibition treatment but in conjunction with sustained removal (or depletion below neuronal ISR-eliciting levels) of unconventional stressors via the elimination of their sources. Indeed, the removal of unconventional stressors would convert unconventional AD (or perspective unconventional AD) into conventional one. In such a setting, the one time only implementation of the composite treatment with BACE1 and/or BACE2 activators and ISR inhibitors would prevent the occurrence of the disease or its recurrence for the remaining lifetime of the treated individual. In addition, the replacement of BACE-activating drugs with directly acting iAβ-specific degradation agents such as appropriately designed proteolysis-targeting chimeras (PROTACs) and molecular-glue degraders (MGDs) allows long-term treatment and opens further therapeutic opportunities for unconventional AD.

### 83.8. RNA-Based Approaches: Highly Promising and Eminently Feasible Therapeutic Strategies for AD

The present *Perspective* also introduces a conceptually distinct set of RNA-based therapeutic strategies for AD. These strategies are founded on the overwhelmingly plausible assumption that the AβPP-independent C99 generation pathway is, in fact, the asymmetric RNA-dependent human AβPP mRNA amplification process (asymmetric because only a 3′-terminal portion of AβPP mRNA, encoding only the C99 fragment of AβPP, is amplified in this process). This process occurs, possibly, exclusively in humans, which explains why Alzheimer’s disease is, apparently, the exclusively human disorder. The reason for such an exclusivity is that whereas human AβPP mRNA is an eligible template for RNA-dependent amplification, its counterparts in non-human mammals are not; no amplification-eligible AβPP mRNA, no production of C99 independently of AβPP, no disease. The objective of the RNA-based therapeutic strategies for AD is, therefore, to render human AβPP mRNA ineligible for amplification or to interfere on the molecular level at defined stages of the amplification process. The eligibility to serve as amplification template is conferred on human AβPP mRNA by the occurrence on the corresponding antisense RNA strand of two complementary element, one 3′-terminal (the terminal complementary element, TCE) another internal (the internal complementary element, ICE), which are mutually accessible within the folded RNA molecule. These elements are the prime interference targets. Of the two, the TCE is more feasible to interfere with because the necessary modifications of the AβPP gene or AβPP mRNA would occur in the 5′ untranslated region, thus preserving their protein-coding potential. One therapeutic strategy, centered on the AβPP gene, is to shift its transcription start site, TSS. The upstream shift would render the TCE non-terminal whereas the downstream shift would remove either a portion of or the entire TCE; in either case, the amplification process would be disrupted.

Another AβPP gene-centered approach is to remove or to replace, via gene editing in neuronal cells, gene segments encoding a portion of or the entire TCE element. The outcome would be identical to that of a TSS shift: the disruption of the amplification process. The feasibility of these approaches is validated by the observations that human AβPP mRNA expressed from 5′-modified transgenes in mouse models cannot be amplified (discussed in Section 18 above).

The third RNA-based strategy centers on antisense AβPP RNA. In this approach, anti-antisense oligonucleotides targeting either the TCE or the ICE may block their interaction and thus disrupt the amplification process. Moreover, anti-antisense oligonucleotides targeting other regions of AβPP antisense RNA may potentially render the TCE and ICE elements mutually inaccessible within the folded RNA molecule. For two reasons this strategy is more feasible and practical than the other two. First, since the preservation of the coding capacity is not an issue, antisense RNA presents many more potential target sites. Second reason is the feasibility of delivery of oligonucleotides: lipids vehicles have been recently developed that are capable of carrying nucleic acid loads through the blood–brain barrier [290,291,292,293]. All three strategies would, depending on the timing of their implementation, either prevent or disrupt the AβPP mRNA amplification process. Accordingly, they would either preclude the occurrence of AD or arrest the progression of the disease equally efficiently in its both conventional and unconventional forms.

In conclusion, the fundamental principles of the ACH2.0 in general and of its latest version in particular are, apparently, solid. Since its inception the theory underwent a remarkable evolution. There is little doubt that it will continue to evolve. However, there is also a firm conviction that the current level of understanding is sufficient to inform and guide the development of potent therapies for AD and AACD. Hope is in the air!
